# Supplementary material for: Associations of menstrual health with school absenteeism and examination performance among Ugandan secondary school students: A prospective study
Source: PLoS One. 2026 Jan 23;21(1):e0326549. doi: 10.1371/journal.pone.0326549 (PMC12829969; doi:10.1371/journal.pone.0326549)
Supplement: S1 Table — (DOCX) [file pone.0326549.s001.docx]

**Supplementary Table 1. Baseline menstrual-related exposures by endline school absenteeism for any reason**

| **Variable** | **Number of participants** | **Baseline days of school missed overall** | | **Endline days of school missed overall (n= 1192)** | |
| --- | --- | --- | --- | --- | --- |
|  | **Frequency (%)** | **Mean days (SE)** | **aIRR**  **(95%CI)^1^** | **Standardized**  **mean exam score (SE)** | **aSMD (95%CI)^1^** |
| **Level 2 – Menstrual support** | | | | | |
| **Social support for menstruation** | | | P=0.10 |  | P=0.61 |
| Yes | 1010 (84.7) | 1.20 (0.04) | 1 | 2.12 (0.07) | 1 |
| No | 182 (15.3) | 1.43 (0.10) | 1.11 (0.98-1.26) | 2.16 (0.20) | 1.05 (0.87, 1.26) |
| **Level 3 – Menstrual knowledge, attitudes and adequate product use** | | | | | |
| **Adequate product use** | | | P=0.11 |  | P=0.04 |
| Yes | 1106 (92.8) | 1.21 (0.04) | 1 | 2.08 (0.70) | 1 |
| No | 86 (7.2) | 1.43 (0.12) | 1.15 (0.97-1.35) | 2.71 (0.29) | 1.29 (1.01, 1.66) |
| **Attitudes towards menstruation** | | | P=0.24 |  | P=0.50 |
| Positive (2-3) | 614 (51.5) | 1.21 (0.05) | 1 | 2.11 (0.09) | 1 |
| Negative (0-1) | 578 (48.5) | 1.27 (0.05) | 1.06 (0.95-1.16) | 2.14 (0.10) | 1.05 (0.92, 1.20) |
| **Knowledge of puberty and menstruation** | | | P=0.06 |  | P=0.21 |
| High (7-9) | 198 (16.6) | 1.28 (0.09) | 1 | 2.22 (0.18) | 1 |
| Medium (4-6) | 839 (70.4) | 1.24 (0.04) | 0.89 (0.78-1.02) | 2.06 (0.08) | 0.86 (0.72, 1.03) |
| Low (0-3) | 155 (13.0) | 1.17 (0.09) | 0.81 (0.67-0.97) | 2.30 (0.20) | 0.96 (0.74, 1.23) |
| **Level 4 – Menstrual pain, pain management and experience of teasing** | | | | | |
| **Menstrual pain at LMP** | | | P<0.001 |  | P=0.007 |
| No pain | 305 (25.6) | 0.88 (0.05) | 1 | 1.64 (0.12) | 1 |
| Any pain | 887 (74.4) | 1.36 (0.04) | 1.41 (1.26-1.57) | 2.29 (0.08) | 1.24 (1.06, 1.45) |
| **Effective pain management strategy** |  |  | P<0.001 |  | P=0.01 |
| No pain | 305 (25.6) | 0.88 (0.05) | 1 | 1.63 (0.12) | 1 |
| Pain and used at least one effective management strategy | 538 (45.1) | 1.32 (0.05) | 1.39 (1.24-1.56) | 2.23 (0.10) | 1.19 (1.01, 1.41) |
| Pain and did not use at least one effective strategy | 349 (29.3) | 1.43 (0.07) | 1.44 (1.26-1.64) | 2.39 (0.12) | 1.32 (1.10, 1.58) |
| **Menstrual pain relief** | |  | P<0.001 |  | P=0.03 |
| No pain | 305 (25.6) | 0.88 (0.05) | 1 | 1.64 (0.12) | 1 |
| All/most pain relieved | 371 (31.1) | 1.43 (0.07) | 1.50 (1.32-1.70) | 2.30 (0.13) | 1.25 (1.04, 1.46) |
| Some/none of pain relieved | 516 (43.3) | 1.32 (0.05) | 1.34 (1.20-1.51) | 2.28 (0.10) | 1.24 (1.05, 1.49) |
| **Menstrual Practice Needs Score (MPNS score)** | |  | P<0.001 |  | P=0.03 |
| High - few unmet needs (2.38-3.00] | 445 (37.6) | 0.95 (0.05) | 1 | 1.83 (0.11) | 1 |
| Medium (1.88-2.38] | 372 (31.4) | 1.21 (0.06) | 1.25 (1.11-1.40) | 2.11 (0.12) | 1.10 (0.93, 1.29) |
| Low - many unmet needs (0.00-1.88] | 366 (30.9) | 1.56 (0.07) | 1.46 (1.29-1.65) | 2.48 (0.14) | 1.23 (1.03, 1.47) |
| **Experience of teasing by boys** | |  | P=0.06 |  | P=0.18 |
| No | 1098 (92.1) | 1.20 (0.03) | 1 | 2.07 (0.07) | 1 |
| Yes | 94 (7.9) | 1.67 (0.15) | 1.19 (0.99-1.42) | 2.73 (0.32) | 1.19 (0.92, 1.53) |
| **Experience of teasing by girls** | |  | P=0.62 |  | P=0.58 |
| No | 1087 (91.2) | 1.22 (0.04) | 1 | 2.08 (0.07) | 1 |
| Yes | 105 (8.8) | 1.43 (0.11) | 1.04 (0.88-1.24) | 2.52 (0.24) | 1.07 (0.84, 1.37) |
| **Level 5 – Menstrual care confidence** | | | | | |
| **Menstrual care confidence (SAMNS score)** | |  | P=0.003 |  | P-trend=0.44 |
| High self-efficacy (69.62-100) | 420 (35.2) | 1.12 (0.06) | 1 | 1.97 (0.11) | 1 |
| Medium (52.31-69.62) | 397 (33.3) | 1.10 (0.05) | 0.93 (0.83-1.04) | 2.20 (0.13) | 1.11 (0.94, 1.31) |
| Low (0-52.31) | 375 (31.5) | 1.50 (0.07) | 1.17 (1.04-1.32) | 2.21 (0.12) | 1.03 (0.87, 1.23) |
| Trend |  |  |  |  |  |
| **Level 6 – Class participation at LMP** | | | | | |
| **Trouble participating in class during LMP** | |  | P<0.001 |  | P=0.12 |
| No | 678 (56.9) | 1.04 (0.04) | 1 | 1.93 (0.89) | 1 |
| Yes | 514 (43.1) | 1.50 (0.06) | 1.20 (1.08-1.33) | 2.37 (0.11) | 1.12 (0.97, 1.29) |
| ^1^ Adjusted for school level clustering and baseline variables at Level 1 (socio-demographic) and at the same or more distal levels. | | | | | |
