# Supplementary material for: Associations of menstrual health with school absenteeism and examination performance among Ugandan secondary school students: A prospective study
Source: PLoS One. 2026 Jan 23;21(1):e0326549. doi: 10.1371/journal.pone.0326549 (PMC12829969; doi:10.1371/journal.pone.0326549)
Supplement: S1 File — (DOCX) [file pone.0326549.s002.docx]

MENISCUS

Menstrual health interventions, schooling and mental health symptoms among Ugandan students (MENISCUS): a school-based cluster-randomised trial

ISRCTN 45461276

Version 7.0 February 2024

SPONSOR: London School of Hygiene & Tropical Medicine

FUNDERS: UK Joint Global Health Trials (Medical Research Council-Department for International Development

-Wellcome Trust) Grant # MR/V005634/1

UK Medical Research Council Grant APP11866

STUDY COORDINATION CENTRE: MRC/UVRI Uganda

LSHTM ethics reference: 22952

UVRI-REC ethics reference: GG/127/21/05/819

UNCST reference: HS1525ES

**Amendment history**

| **Version** | **Changes** |
| --- | --- |
| v1.0 (5 Jan 2021) -  v1.2 (1 Feb 2021) | First version and initial revisions following feedback from TSC and UVRI Science Committee |
| v1.3 (21 May 2021) | - Increased number of schools from 48 to 60 and decreased estimated number of participants from ~4,800 to ~3,600, reflecting change in expected school enrolment. Change also reflected in sample size for process evaluation. (p9, 15, 16, 20-21, 31-2, 34) - Changed participants to S2 students to reflect changes in school terms following COVID-19 closures; references to terms changed to calendar years. (p9, 14, 16, 20-21, 25, 28, 34, 37) - Removed urogenital sub-study procedures from baseline survey and expanded endline urogenital sub-study procedures to include all female participants. Nested cohort therefore removed; diary component still conducted with sample of ~25 girls/school (p9-10, 14, 17, 23, 28-29, 33, 37) - Change to e-assent methods for student participants instead of traditional paper based procedure (p38) - Added detail on contact information in case of suspected TSS (p27) - Added note of MoES member on TSC (p39) - Added section on efforts to avoid contamination between trial arms (p21) - Change in UNFPA representative on TSC (p14) - All ICFs updated to reflect increased number of schools and changes to the school calendar - Main ICFs for girls (1 and 2) updated to include information on urogenital sub-study and diary completion procedures as these are no longer limited to the nested cohort. - ICFs 1-3 updated to include mention of UNEB exam in line with protocol |
| v2.0 (13 Aug 2021) | - Added amendment history - Named Kate Nelson as trial statistician (p4) - Updated WASH inclusion criteria following rapid assessment (p20) - Updated exclusion criteria to specify MH studies or programmes; added language exclusion criteria (p21) - Specified that randomisation will take place within strata (p21) - Added engagement with community advisory boards (p24) - Updated information on COVID-19 school closures (p26) - Clarified school-level approval for UNEB assessments (p39) - Added option for parental consent by phone if necessary (p39-40) - Updated list of TSC members (Annex 1) - Updated data management plan and risk assessment to reflect previous change to sample size and nested cohort procedures (Annex 2, Annex 3) - Updated COVID risk management plan based on continued restrictions (Annex 4) - Updated pain killer use guide to improve clarity and formatting (Annex 6c) - Updates to all ICFs for formatting and clarity (to version 1.2) |
| v3.0 (Jan 2022) | - Updated trial staff to include new trial statistician and clinical officer (p4, 28) - Addition of secondary outcomes on self-confidence in maths and science (p9, 10, 15, 19, 30) and exploratory outcomes for each subject of UNEB exam (separately) (p10, 15, 18) - Addition of procedures for pre-test workshops with teachers in two schools not selected for the main trial (p24-5, ICF18, CRF20) - Corrected date regarding curriculum material the baseline UNEB exam will cover to 2020 (p18) - Clarified that members of the MH Action Group and those participating in the ‘training of trainers’ will be eligible to receive a MH kit with consent/assent (p23) and addition of ICFs for them to receive kit and optional cup (ICF9, ICF12-16) - Specified that there will be structured observations of puberty education and menstrual health trainings (p23 and Annex 23a) - Updated funder representative on TSC to Ms Caroline Harris (p42, Annex 1) - Updated girls’ baseline and endline surveys to add new secondary outcomes and improve skips and clarity on other items (CRF 1 to version 1.0) - Addition of ICF for Head Teacher’s consent to school participation (ICF17) - Updates to Data Management Plan in line with protocol changes (Annex 2) |
| v4.0 (Nov 2022) | - Add Dr Nambusi Kyegombe as principal coinvestigator (p4)  - Removal of testing vaginal swabs for bacterial vaginosis from the urogenital sub-study (p11,12,14,17,20,21,23,28 33, removal of Annex 7)  - Clarification that the diary sub-study will be amongst post-menarche girls only (p22)  - Amend ‘MH Leadership group’ to ‘MH Action group’ (p24)  - Addition of the social norms sub study (p34, Annex 12, 13 & 51)  - Amend number of girls and boys in each focus group from 4 to 6-8 (p36, Annex 20 & 21)  - Requesting a waiver for parental consent of the networking tool (p42, Annex 51) |
| v5.0 (May 2023) | - Added Stephen Lagony as Trial Manager and co-investigator (p5) - Added Rebecca Prah as co-investigator (p5) - Included Quality of life measurement and valuation as a sub-study (p 36-40,43-44, 46-48) - Requesting a waiver for parental consent for the CHU9D piloting and valuation exercise (pg46-47) - Included 60 parents to participate in adult CHU9D valuation exercise (p 37 and 40). - Amended name and contact information of new trial manager in ICF 1, ICF 4, ICF 9, ICF 10, ICF 12, ICF 13, ICF 14, ICF 15, ICF 16, ICF 19, ICF 20, ICF 24). - Clarification that the endline survey for boys that have left school since baseline will be followed up by phone |
| v6.0 (August 2023) | - Increased the number of parents for the quality of life sub-study from 60 (5 parents per each of the 12 schools) to ~120 parents (p38, 39 & 41) |
| V7.0 (February 2024) | - Contact parents of female trial participants to request consent to contact their daughters in future to complete further questionnaires on menstrual health and related outcomes included in the original trial (page 47 ICF 28) - Conduct a quantitative survey and qualitative interviews with stakeholders in two Wakiso secondary schools to understand the context of sleep and it’s relation to menstrual health and mental health (page 42-45 ICF 29-33) - Conduct a feasibility study of a school-based intervention focused on improving sleep and menstrual health in two Wakiso secondary schools (page 42-45) |

**SIGNATURE PAGE**

**The undersigned confirm that the following protocol has been agreed and accepted and that the Chief Investigator agrees to conduct the trial in compliance with the approved protocol and will adhere to the principles outlined in the International Council for Harmonisation Good Clinical Practice (ICH GCP) and/or Medicines for Human Use (Clinical Trials) Regulations 2004 (SI2004/1031), amended regulations (SI 2006/1928) and any subsequent amendments of the clinical trial regulations, the Sponsor’s (and any other relevant) Policies and Standard Operating Procedures (SOPs), and other regulatory requirements as amended.**

**I agree to ensure that the confidential information contained in this document will not be used for any other purpose other than the evaluation or conduct of the clinical investigation without the prior written consent of the sponsor.**

**I also confirm that I will make the findings of the trial publicly available through publication or other dissemination tools without any unnecessary delay and that an honest accurate and transparent account of the trial will be given; and that any discrepancies and serious breaches of GCP from the trial as planned in this protocol will be explained.**

**For and on behalf of the Trial Sponsor:**

**Name: Role: Research Governance and Integrity Office, LSHTM**

**Signature: Date:**

**Chief Investigator:**

| **Name:** | **Helen Weiss** | **Date:** | 23^rd^ February 2024 |
| --- | --- | --- | --- |
| **Signature:** |  |  |  |
|  |  |  |  |

**Main Contacts**

**Chief Investigator**

Name: Professor Helen Weiss

Address: LSHTM, Keppel Street, London, WC1E 7HT, United Kingdom

Email: Helen.Weiss@lshtm.ac.uk

Phone: 0207 927 2087

**Trial Management Group**

Chief Investigator (as above)

Co-investigators:

1. Dr Nambusi Kyegombe, Associate Professor in Social Science of Adolescent and Global Health and Head Of The Social Aspects Of Health Across The Life Course Programme MRC/UVRI And LSHTM Uganda Research Unit, Email: [Nambusi.Kyegombe@lshtm.ac.uk](mailto:Nambusi.Kyegombe@lshtm.ac.uk)
2. Prof Janet Seeley, Professor of Anthropology and Health , LSHTM, UK and Head of Social Science Programme, MRC/UVRI and LSHTM Email: janet.seeley@lshtm.ac.uk
3. Dr Stella Neema, Senior Lecturer in Anthropology, Makerere University, Email: sheisim@yahoo.com
4. Prof Chris Bonell, Professor of Public Health Sociology, LSHTM, UK, Email: chris.bonell@lshtm.ac.uk
5. Dr Fred Matovu, Senior Lecturer and Director, PADRI, Makerere University, Kampala Uganda Email: frmatov2000@yahoo.co.uk
6. Prof John Jerrim, Professor of Education and Social Statistics, UCL Institute of Education, University College London, UK, Email: j.jerrim@ucl.ac.uk
7. Dr Belen Torondel, Assistant Professor, Faculty of Infectious and Tropical Diseases, LSHTM, UK. Email: belen.torondel@lshtm.ac.uk
8. Dr Suzanna Francis, Associate Professor, LSHTM, UK, Tel. +44 (0)20 7927 2245, Email: Suzanna.Francis@lshtm.ac.uk
9. Dr Clare Tanton, Assistant Professor in Epidemiology, LSHTM, UK, Tel. +44 20 7958 8393, Email: Clare.Tanton@lshtm.ac.uk
10. Dr Giulia Greco, Assistant Professor and MRC Fellow (Health Economics), LSHTM, UK, Email: Giulia.Greco@lshtm.ac.uk
11. Dr Catherine Kansiime, MRC/UVRI and LSHTM P.O.Box 49, Entebbe-Uganda. Plot 51-59 Nakiwogo Road, Email: Catherine.Kansiime@mrcuganda.org
12. Ms Kate Nelson, Research Fellow, LSHTM, UK: Email [kate.nelson@lshtm.ac.uk](mailto:kate.nelson@lshtm.ac.uk);
13. Mr Stephen Lagony, MRC/UVRI and LSHTM P.O.Box 49, Entebbe-Uganda. Plot 51-59 Nakiwogo Road, stephen.lagony@mrcuganda.org
14. Rebecca Prah, Ph.D. student, LSHTM, UK: Email: [rebecca.prah@lshtm.ac.uk](mailto:rebecca.prah@lshtm.ac.uk);

Trial Statistician: Dr Levicatus Mugenyi

Trial Manager: Mr Stephen Lagony MRC/UVRI and LSHTM P.O.Box 49, Entebbe-Uganda. Plot 51-59 Nakiwogo Road, Email: stephen.lagony @mrcuganda.org

**Trial Coordination Centre**

For general queries, supply of trial documentation, and collection of data, please contact:

Trial Manager and/or Data Manager: Mr Stephen Lagony

Address: Plot 51-59 Nakiwogo Road, Entebbe

Tel: +256782386558

**Clinical Queries**

Clinical queries should be directed to Mr. Stephen Lagony and Clinical Officer Dr Denis Ssenyondwa.

**Sponsor**

London School of Hygiene & Tropical Medicine is the main research sponsor for this study. For further information regarding the sponsorship conditions, please contact the Research Governance and Integrity Office:

London School of Hygiene & Tropical Medicine

Keppel Street

London WC1E 7HT

Tel: +44 207 927 2626

Email: rgio@lshtm.ac.uk

**Funder**

Department of Health and Social Care (DHSC) through the National Institute for Health Research (NIHR), Foreign, Commonwealth and Development Office (FCDO), the Medical Research Council (MRC) and the Wellcome Trust through the Joint Global Health Trials scheme

This protocol describes the “Menstrual health interventions, schooling and mental health symptoms among Ugandan students (MENISCUS): a school-based cluster-randomised trial study” and provides information about procedures for entering participants. The protocol should not be used as a guide for the treatment of other participants; every care was taken in its drafting, but corrections or amendments may be necessary. These will be circulated to investigators in the study, but centres entering participants for the first time are advised to contact the trials centre to confirm they have the most recent version.

Problems relating to this trial should be referred, in the first instance, to the study coordination centre.

This trial will adhere to the principles outlined in the International Conference on Harmonisation Good Clinical Practice (ICH GCP) guidelines, protocol and all applicable local regulations.

**TABLE OF CONTENTS**

[1. INTRODUCTION 14](#_Toc159563074)

[1.1. BACKGROUND 14](#_Toc159563075)

[1.2. RATIONALE FOR CURRENT STUDY 16](#_Toc159563076)

[**1.2.1.** Hypothesis 16](#_Toc159563077)

[**1.2.2.** Principal research questions 16](#_Toc159563078)

[**1.2.3.** How this trial complements other trials 16](#_Toc159563079)

[**1.2.4.** Generalisability of results 17](#_Toc159563080)

[2. STUDY OBJECTIVES 17](#_Toc159563081)

[2.1. PRIMARY OBJECTIVES 17](#_Toc159563082)

[2.2. SECONDARY OBJECTIVES 17](#_Toc159563083)

[3. STUDY DESIGN 18](#_Toc159563084)

[3.1. STUDY OUTCOME MEASURES 19](#_Toc159563085)

[**3.1.1.** Primary outcomes measures 19](#_Toc159563086)

[**3.1.2.** Secondary outcomes 20](#_Toc159563087)

[**3.1.3.** Exploratory outcome 20](#_Toc159563088)

[4. Selection and withdrawal of PARTICIPANTs 23](#_Toc159563089)

[4.1. Pre-randomisation or pre-registration evaluations 23](#_Toc159563090)

[4.2. Inclusion Criteria 23](#_Toc159563091)

[4.3. EXCLUSION CRITERIA 23](#_Toc159563092)

[5. RANDOMISATION, masking AND ENROLMENT PROCEDUREs 24](#_Toc159563093)

[5.1. ENROllment / Registration PRACTICALITIES 24](#_Toc159563094)

[**5.1.1.** Recruitment of schools 24](#_Toc159563095)

[**5.1.2.** Recruitment of trial participants 24](#_Toc159563096)

[5.2. Randomisation 24](#_Toc159563097)

[5.3. MASKING 24](#_Toc159563098)

[5.4 CONTAMINATION 25](#_Toc159563099)

[6. Intervention 25](#_Toc159563100)

[6.1. Optimised usual care 25](#_Toc159563101)

[6.2. Intervention components 25](#_Toc159563102)

[6.3. PRETEST OF DELIVERY APPROACH 27](#_Toc159563103)

[6.4. Stakeholder’s engagement 27](#_Toc159563104)

[6.5. Adherence to study intervention protocol and assessment of compliance 28](#_Toc159563105)

[6.6. Name and description of each non-investigation medicinal product (NIMP) 28](#_Toc159563106)

[7. RIsks and BENEFITS 28](#_Toc159563107)

[7.1. Risks 28](#_Toc159563108)

[**7.1.1.** Participation in the research data collection 28](#_Toc159563109)

[**7.1.2.** Participation in the intervention 28](#_Toc159563110)

[**7.1.3.** COVID-19 related risks 29](#_Toc159563111)

[7.2. Benefits 29](#_Toc159563112)

[8. safety reporting for non-drug trials 30](#_Toc159563113)

[8.1. Definitions 30](#_Toc159563114)

[8.2. Intensity 30](#_Toc159563115)

[8.3. Reporting Procedures 31](#_Toc159563116)

[**8.3.1.** Non-serious AEs 31](#_Toc159563117)

[**8.3.2.** Serious AEs 31](#_Toc159563118)

[9. ASSESSMENT AND FOLLOW-UP 32](#_Toc159563119)

[9.1. INTERVENTION IMPACT ASSESSMENT 32](#_Toc159563120)

[**9.1.1.** Baseline assessment of self-reported outcomes 32](#_Toc159563121)

[**9.1.2.** Baseline assessment of educational performance 32](#_Toc159563122)

[**9.1.3.** Endline assessment of self-reported outcomes 32](#_Toc159563123)

[**9.1.4.** Endline assessment of educational performance 32](#_Toc159563124)

[9.2. EDUCATIONAL OUTCOMES SUB-STUDY 33](#_Toc159563125)

[9.3. Urinary Tract Infection SUB-STUDY 33](#_Toc159563126)

[9.4 SOCIAL NORMS SUB-STUDY 34](#_Toc159563127)

[9.5 Process Evaluation 34](#_Toc159563128)

[9.6 Economic Evaluation 37](#_Toc159563129)

[9.7. QUALITY OF LIFE MEASUREMENT AND VALUATION SUB-STUDY 37](#_Toc159563130)

[9.8 SLEEP sub-study 42](#_Toc159563131)

[9.8 Policy Analysis 50](#_Toc159563132)

[9.9 loss to follow-up 50](#_Toc159563133)

[9.10 trial closure 50](#_Toc159563134)

[9.11 CONSENTING FOR FURTHER SURVEYS 50](#_Toc159563135)

[10. data management and analysis 51](#_Toc159563136)

[10.1. Sample size justification 51](#_Toc159563137)

[10.2. Statistical Analyses 51](#_Toc159563138)

[10.3. Proposed frequency of analyses 53](#_Toc159563139)

[11. monitoring 54](#_Toc159563140)

[11.1. Risk assessment 54](#_Toc159563141)

[11.2. Data Monitoring committee 54](#_Toc159563142)

[11.3. MoNITORING AT STUDY COORDINATION CEntre/data management 54](#_Toc159563143)

[11.4. monitoring at local site/ SPONSOR MONITORING 54](#_Toc159563144)

[12. regulatory issues 54](#_Toc159563145)

[12.1. Ethics approval 54](#_Toc159563146)

[12.2. Assent and CONSENT 55](#_Toc159563147)

[12.3. Confidentiality 57](#_Toc159563148)

[12.4. Indemnity 57](#_Toc159563149)

[12.5. Sponsor 57](#_Toc159563150)

[12.6. Funding 58](#_Toc159563151)

[12.7. Audits and Inspections 58](#_Toc159563152)

[12.8. PROTOCOL DEVELOPMENT 58](#_Toc159563153)

[13. Trial Management 58](#_Toc159563154)

[13.1. Trial Steering Committee (TSC) 58](#_Toc159563155)

[13.2. Independent Data Monitoring and Ethics Committee (IDMEC) 59](#_Toc159563156)

[13.3. Trial Management Group (TMG) 59](#_Toc159563157)

[14. Stakeholder engagement anD application of results 60](#_Toc159563158)

[15. Publication Policy 61](#_Toc159563159)

[16. References 62](#_Toc159563160)

[17. AnnexeS 66](#_Toc159563161)

**Glossary of Abbreviations**

| BASHH | British Association of Sexual Health and HIV |
| --- | --- |
| BWS | Best-worst scaling |
| CASI | Computer assisted self-interview |
| CHU9D | Child Health Utility 9-dimension questionnaire |
| CRF | Case report form |
| DCE | Discrete choice experiment |
| FDA | Food and rug Administration |
| FGD | Focus group discussion |
| ICF | Informed consent form |
| IDI | In-depth interview |
| IDMEC | Independent Data Monitoring and Ethics Committee |
| MH | Menstrual Health |
| MHH | Menstrual health and hygiene |
| MHM | Menstrual hygiene management |
| MOES | Ministry of Education and Sports |
| MOH | Ministry of Health |
| MRC | UK Medical Research Council |
| NGO | Non-governmental organisation |
| oUC | optimised Usual Care |
| PBM | Preference-based measures |
| PE | Process Evaluation |
| PIASCY | Presidential Initiative on AIDS strategy for Communication to the Youth |
| PPP | Public-Private Partnership |
| QALYs | Quality adjusted life years |
| QoL | Quality of life |
| RTI | Reproductive Tract Infection |
| SG | Standard gamble |
| SRH | Sexual and reproductive health |
| SSA | Sub Saharan Africa |
| TMG | Trial Management Group |
| TSC | Trial Steering Committee |
| TSS | Toxic Shock Syndrome |
| TTO | Time trade-off |
| UNBS | Uganda National Bureau of Standards |
| UNCST | Uganda National Council for Science and Technology |
| UNEB | Uganda National Examination Board |
| USE | Universal Secondary Education |
| UVRI | Uganda Virus Research Institute |
| WASH | Water, sanitation and hygiene |
| WHO | World Health Organization |

**Keywords**

Menstrual health, school-based intervention, adolescent health, education, global health, Uganda

**Study Summary**

| **TITLE** | Menstrual health interventions, schooling and mental health symptoms among Ugandan students (MENISCUS): a school-based cluster-randomised trial |
| --- | --- |
| **DESIGN** | An open-cohort cluster randomised trial (CRT) with 60 schools (clusters) randomised 1:1 to 2 arms, with a mixed-methods process evaluation, and economic and policy analyses. Within each school, we will collect baseline data during Q4 2021 from the class of students due to start Secondary 2 in August 2021 (~3600 females and a sample of ~900 males). In Q1 2023 (approximately), we will collect endline data from the same class cohort, expected to be in Secondary 3 at this time. Selected secondary outcomes will be assessed in a sample of approximately ~1500 girls at endline. The exact timing of the baseline and endline surveys may change due to school closures and the resulting shifts to the academic calendar. |
| **AIMS** | 1. To evaluate whether the MENISCUS intervention improves educational attainment and reduces mental health symptoms (primary outcomes) among Secondary girls in Uganda. 2. To evaluate whether the MENISCUS intervention improves: 3. knowledge of puberty and menstruation; attitudes towards menstruation (girls and boys); 4. menstrual practices at last menstrual period (LMP); 5. pain management during LMP; 6. self-efficacy of MH; 7. quality of life measurement and valuation and happiness; 8. prevalence of urinary tract infections (among post-menarchal girls) 9. school and class absence during menses (subsample); 10. school and class absence overall (subsample); 11. self-confidence in maths and (separately) science abilities 12. To evaluate the costs of setting up and running the intervention package, the unit cost per female student reached, and the incremental cost-effectiveness of the intervention per unit increase in selected policy-relevant outcomes, relative to optimised usual care 13. To assess whether the intervention was implemented with fidelity, and to understand the contextual factors affecting implementation, the acceptability to participants, and the intervention mechanisms. We will achieve this through a process evaluation including quantitative indicators and qualitative data collected from in-depth interviews and focus group discussions. 14. To assess the policy environment around menstrual health in Uganda, focusing on how implementing the intervention contributes to, and aligns with, the attainment of the Government policy objectives on menstruation management in schools. We will assess the policy/regulatory frameworks to which the outcomes of the intervention contribute, identify the supportive and constraining factors to the implementation of the policy guidelines and how the findings of the intervention inform refinement of current policy. |
| **POPULATION** | Students in the class cohort due to start Secondary 2 (S2) in 2021 (median age ~15 years) who attend 60 eligible schools in Wakiso and Kalungu Districts, Uganda. |
| **ELIGIBILITY** | Baseline survey: All female students and a random sample of male students in the class cohort starting S2 in 2021 present during the survey administration period (Q4 2021); Endline survey: All female students and a random sample of male students in the same class cohort (expected to be in S3 in Q1 2023) present during the survey administration period in Q1 2023, who were present for at least part of the intervention period. |
| **OUTCOME MEASURES**  **INTERVENTION** | **Primary outcomes:**  1. Educational attainment in girls  2. Mental health symptoms in girls  **Secondary outcomes:**  1. Knowledge of puberty and menstruation; attitudes towards menstruation in girls and boys  2. Menstrual practices at last menstrual period (LMP)  3. pain management at LMP  4. Self-efficacy of menstrual health  5. Quality of life measurement and valuation and happiness  6. Prevalence of urinary tract infections in girls (among post-menarchal girls)  7. School and class absence during menses (assessed in sample of girls)  8. School and class absence overall in girls (assessed in sample of girls)  9. Self-confidence in maths and (separately) science abilities  **Exploratory outcomes:**  School dropout in girls; educational attainment in each subject separately (biology, maths, and English) in girls as assessed by UNEB  School-based intervention addressing menstrual health (MH) knowledge, social (attitudes, stigma) and physical barriers (menstrual products, improved water, sanitation and hygiene (WASH) facilities) to MHH, and pain management.  **Intervention:**   1. **Puberty Education Workshop:** Train teachers of the participating students on how to deliver the puberty session to girls and boys. Teachers to develop an action plan, delivery of which will be overseen by a MH Leadership Group and the District Inspector of School. 2. **Drama skit:** MH drama skit developed by female and male students and performed at parents’ day or other suitable occasion. 3. **Provision of an MH kit:** Distribution of a MH kit (reusable sanitary pads provided in a bag with underwear, a water bottle, soap and a towel) with the option to receive a medical-grade silicone, re-usable menstrual cup and a container for disinfection and storage. Teachers and prefects to train students to use the products. Training includes an education session on menstruation for girls and boys, and a gender-specific participatory session. 4. **Pain relief:** Information on effective period pain management, addressing common myths included in MH Training. Vouchers for analgesics will be included in the MH kit, each to be redeemed for 6 paracetamol or ibuprofen tablets per month from the school nurse or specified teacher. 5. **Improving school WASH facilities:** Improvements to school WASH facilities (fixing doors, providing locks, sanitary bins, toilet paper cages, water carriers, liquid soap (5L), and water (20L) containers) over seen by The MH Leadership Group, monitored by the District Inspector of Schools during routine termly school inspection visits.   **Control arm**: Optimised usual care (distribution of government guidelines for MHM in schools). |
| **duration** | 29 months |

**Reference diagram**

Date

**Figure 1: MENISCUS trial flowchart**

Number of secondary schools in Wakiso and Kalungu district assessed for eligibility (n=X1)

Q2 2021

Did not meet eligibility criteria (n=)

Eligible schools for random allocation (Wakiso n=X2; Kalungu n=X2)

Selected 60 schools for random allocation to trial arm;

Open cohort: All students starting S2 on class registers

Q2 2021

Q2 & Q3 2021

Community sensitization and parental/caregiver consent

Parental consent refused Open cohort (n=X3)

**Open cohort baseline survey**

All students with parental consent

Q4 2021

Student assent refused

Open cohort (n=X4)

**MENISCUS arm**

30 schools

**Randomisation of schools**

**Optimised usual care arm**

30 Schools

Q4 2021

**Received intervention**

X5 schools

Average open cohort size girls (n=X6)

Average open cohort size boys (n=X7)

**Received optimised usual care**

X8 schools

Average open cohort size girls (n=X9)

Average open cohort size boys (n=X10)

Q1-Q4 2022

**Endpoint Survey**

X14 schools

Open cohort assessment completed girls (n=X15)

Open cohort assessment completed boys (n=X16)

**Endpoint Survey**

X11 schools

Open cohort assessment completed girls (n=X12)

Open cohort assessment completed boys (n=X13)

Q1 2023

**Analysis**

X17 schools

Open cohort – girls (n=X18)

Open cohort – boys (n=X19)

Subsample - diary (n=X20)

**Analysis**

X20 schools

Open cohort – girls (n=X22)

Open cohort – boys (n=X23)

Subsample - diary (n=X24)

Q2 2023

# INTRODUCTION

## BACKGROUND

Menstrual health (MH) is a neglected public health issue. Poor management of menstruation affects many girls in low- and middle-income countries (LMIC) (1,2). Challenges associated with effective menstrual hygiene management (MHM) include lack of access to hygienic absorbents, inadequate facilities to change, clean and dispose of these, lack of access to soap and water, and lack of privacy. In addition, inadequate social support and presence of taboos can lead to psychosocial consequences of menstruation including shame, fear, anxiety and distraction(2–6). These can potentially affect girls’ ability to succeed and thrive within the school environment (7).

Effective MH interventions may lead to sustained, long-term benefits to education (8), health (mental, reproductive, sexual) (9), productivity (1) and the environment (10). There is increasing awareness of MH globally including in Uganda (e.g. “menstrual activism) (11,12) but a lack of robust evidence on the effectiveness of interventions and hence lack of evidence-based guidance on addressing MH (4,13,14) for policy-makers to use.

Schools provide an important setting for addressing both the challenges of stigma around MH, lack of menstrual literacy, and practical challenges in managing menstruation. The first priority of UNICEF/ Columbia University’s “MHM in Ten” initiative to advance the MH agenda in schools by 2024 is to generate robust evidence on whether improving MH improves girls' attendance and attainment at school (13–15). Such evidence is critical for achieving scale-up with the education sector. Improving secondary education for girls, is a global priority and a key determinant of health and development through multiple pathways (e.g. earnings and standards of living; child marriage and early childbearing; fertility and population growth; health, nutrition, and well-being; agency and decision-making; and social capital and institutions)(16). For many indicators, the gains associated with educational attainment tend to be substantial only with a secondary education (16).

In Uganda, the Government has proven political will to improve MH (17), for example by forming a National MHM Steering Committee, holding the first international MHM conference in 2014, launching of the Menstrual Hygiene Charter in 2015 (18) and celebrating International MHM Day. In 2015, The Ministry of Education and Sports (MoES) issued a circular to all primary and secondary schools with instructions for improving MH (circular No. 1/2015). A recent review found that although most schools received this circular, few had acted upon it. Reasons for this included unconducive social and physical school environments (e.g. boys teasing girls, unavailability of MHM facilities and materials), limited budget for MH, unclear roles and responsibilities for MH within schools, and MHM issues being left entirely to the senior teachers (19).

- - 1. **Current knowledge and research to be addressed**

MH research has predominantly been qualitative and descriptive(4). A recent systematic review of menstrual experiences in LMICs concluded that rigorous trials are needed to evaluate interventions which (like MENISCUS) address common antecedents of menstrual experience, including knowledge, social support, restrictive behavioural expectations, and the physical environment(20). A 2016 systematic review (8) found promising evidence of the effectiveness of MH interventions on educational and psychosocial outcomes, but insufficient evidence of effect due to a small number of trials (N=8), high risk of bias, and substantial heterogeneity. The effect of MH interventions on UTIs remains unclear (9). A 2013 systematic review (9) of the health and social effects of poor MH found that of 11 studies (including only one RCT) investigating the association between MH and urogenital infections, 7 found an increased risk associated with “worse” MH (defined differently for each study but generally meaning not using disposable sanitary pads).

Our formative research has shown that poor MH is a key factor associated with girls missing secondary school in Wakiso District, Uganda, and affects their wellbeing (“MENISCUS-1”) (21). The study confirmed an unmet need for effective interventions to enable girls to better manage both the psychosocial (anxiety, stigma and distress) and physical aspects (pain management, use of appropriate materials to eliminate leakage of menstrual blood) of menstruation (21). MENISCUS-1 highlighted the importance of including boys and teachers in a school-based MH intervention. Poor MH may be associated with UTI.

Based on these findings, we co-developed a school-based MH intervention (MENISCUS) with stakeholders. The intervention is grounded in social cognitive theory (SCT) (Figure 2) and was piloted in two schools in Entebbe during the MENISCUS-2 study (22).


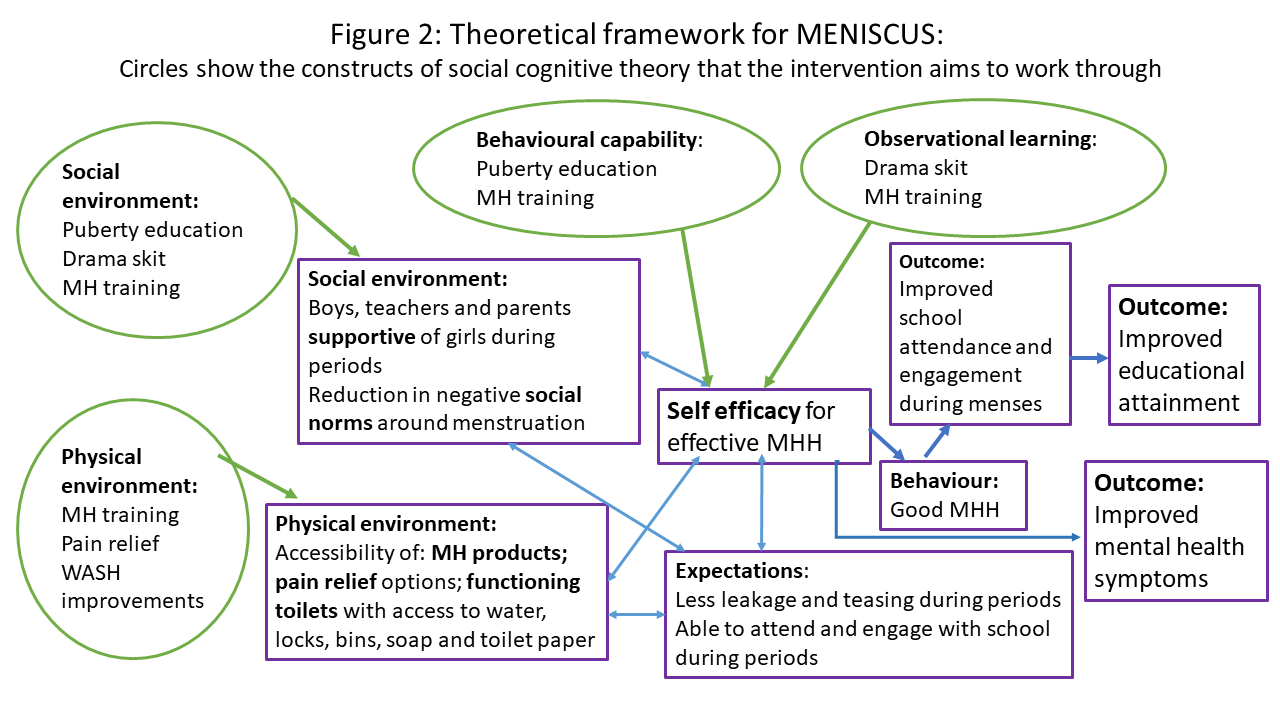
Figure 2: Theoretical Framework for MENISCUS

The pilot study confirmed that an intervention trial to evaluate the impact of the MENISCUS intervention on education, health and wellbeing outcomes can feasibly be conducted in secondary schools in Wakiso District, with high levels of parental consent and student assent (89%). Over the 9 months of intervention delivery, the mean Strengths and Difficulties Questionnaire (SDQ-25) score decreased from 10.3 to 9.2 in girls (standardised mean difference=0.20; p=0.006 adjusted for clustering), indicating improved mental wellbeing. No decrease in SDQ-25 was seen among boys (baseline score=9.88, endline score=9.91; p-value for difference=0.98), as hypothesized. Qualitative findings attributed improved school attendance in girls due to the intervention. Data from daily diaries supported this, with the odds ratio (OR) associated with missing school on period days compared to non-period days decreasing from OR=2.17 (95%CI:1.61-2.92) at baseline to OR=1.29 (95%CI:1.02-1.63) at endline (p=0.01 for interaction)(22). There were improvements in all mediating factors on our theoretical framework (Figure 2). The intervention was highly acceptable to teachers, parents, girls, boys and District and Ministry officials. The cost of delivering the intervention was £6614 per school, similar to other Ugandan school interventions.

| **Outcome among girls** | **Baseline %**  **(N=232)** | **Endline %**  **(N=188)** | **Adj. prevalence ratio**^[[1]](#footnote-2)^ **(95%CI)** |
| --- | --- | --- | --- |
| Answered all 9 knowledge questions correctly | 12% | 24% | 3.07 (1.49-6.32) |
| Used only manufactured methods^[[2]](#footnote-3)^ at last menstrual period (LMP) | 73% | 89% | 1.23 (1.12-1.35) |
| Used effective pain relief method if had pain at LMP | 76% | 92% | 1.19 (1.08-1.32) |
| Reported that boys tease girls about periods | 14% | 9% | 0.57 (0.34-0.97) |
| Reported leaking blood during LMP | 47% | 35% | 0.76 (0.57-1.01) |
| Reported feeling anxious about next period | 59% | 34% | 0.57 (0.46-0.69) |

Table 1:Key results from the MENISCUS-2 pilot study

In summary, these findings provide compelling evidence that the MENISCUS intervention may improve girls’ education and health**.**

- - 1. **Innovation**

The intervention has been designed to be culturally appropriate, aligned with Government guidelines, cost-effective, environmentally-friendly and practically sustainable within the schools. We will assess these elements through a process evaluation, health economics component and policy analysis.

The proposed intervention is novel in several ways. It will be the first to i) be truly multi-component (i.e. not focusing primarily on either education, provision of pads, or improvement of toilet facilities), ii) address pain management (a major reason for school absence in girls), iii) focus on boys as well as girls (enabling us to address stigma and improve the school environment), and iv) include secondary schools in rural and peri-urban areas (most previous studies have been in primary schools in rural areas).

# RATIONALE FOR CURRENT STUDY

### Hypothesis

Our hypothesis is that the MENISCUS intervention will lead to improved educational performance, mental health outcomes and related health- and well-being outcomes among Ugandan secondary school girls. We hypothesize that this will be achieved by improving self-efficacy for effective MH in schools, through improving the MH social and physical environment, behavioural capability and observational learning.

### Principal research questions

1. Does the MENISCUS menstrual health intervention improve educational attainment and mental health symptoms (primary outcomes) among secondary school girls in Uganda?
2. Does the intervention improve (i) menstrual practices, self-efficacy in MH, school attendance, pain management and prevalence of urinary tract infections in girls, and (ii) puberty and menstrual knowledge and attitudes in boys and girls (secondary outcomes)?
3. How much will it cost to scale-up the intervention, and how can the results best inform national and regional MH policies?

### How this trial complements other trials

The proposed trial will complement other trials in the following ways:

**Multi-component intervention**: In line with the SCT underpinning our research, the multi-component intervention addresses individual, social, behavioural and environmental barriers to good MH, mental health symptoms and educational engagement. There is consensus that MH interventions need to address menstrual stigma and literacy as well as the provision of products or improving WASH facilities (13). The recent systematic review of menstrual experiences confirmed that multiple aspects of menstrual experience contribute to adverse educational engagement and psychosocial health (20). Further, our pilot work showed synergies between the intervention elements e.g. the drama skit reinforces puberty knowledge, reduces stigma and engages parents and boys. We will assess each intervention element through the PE that will include quantitative indicators of each element, and qualitative data on their feasibility, dose and reach.

**Pain during menses** is a major contributor towards absenteeism and poor attention at school, including in this setting(21) and to our knowledge, our proposed trial is the first large-scale trial to directly address this issue. Results from the pilot study show substantial improvements in pain management.

**Inclusion of boys**: Most MH trials focus exclusively on girls, but sustainable changes in MH depend on addressing stigma around menstruation, and inclusion of boys is central to changing the MH environment. Our focus on including boys aligns with SCT (i.e. the intervention provides positive reinforcement for behavioural change by improving the school environment), and was highlighted by our qualitative findings, and discussions with stakeholders. The focus on boys as well as girls is also aligned with the WHO framework for Health Promoting Schools(23) and a report commissioned by the Gates Foundation on Menstrual Health and Gender Equity(24).

**Secondary schools in a peri-urban area:** Many trials to date have been in primary schools, and in rural areas. We have shown that MH is a critical issue in a peri-urban area in Entebbe, where most girls can buy disposable pads for some of the time. A trial in secondary schools is important given the recognised importance of girls’ secondary education to future development(16), and the increased maturity of girls of this age lending itself to self-efficacy.

### Generalisability of results

We designed the intervention to be sustainable and cost-effective, and developed it during workshops with the Ministry of Education and Sports (MoES) and Ministry of Health (MoH) to facilitate national scale-up. We will address generalisability by i) continuing to work with stakeholders to identify the best methods to increase sustainable school ownership of interventions, including strategies for ongoing monitoring of elements of the intervention by Inspectors of Schools and Community Development Officers at District level; ii) conducting policy analyses to assess the policy environment around MH in Uganda, focusing on how best to improve MH in line with Government policy framework, including financial sustainability; iii) assessing how the MENISCUS findings can inform refinement of the Government MHM Guidelines and implementation.

Our design enables us to evaluate the impact of these current guidelines in the control arm (“optimised Usual Care” (oUC)) and in diverse settings. We will estimate the incremental cost and cost-effectiveness of delivering the MENISCUS package, and estimate scale-up scenarios following Government guidelines. The process evaluation will aim to determine which contextual factors (e.g. setting, school, staff, student) affect implementation. The qualitative data on mechanisms and context plus planned subgroup analyses will provide data on the most promising settings for scale-up. This will provide an informed view of the potential transportability of the intervention. The presence of a UNFPA representative on the TSC will facilitate translation to policy. We will also use the trial to strengthen MH research in the region, through workshops with regional MH researchers with whom we have established by our leadership of a GCRF Networking Grant for an East and Southern African MHM Research Network.

# STUDY OBJECTIVES

The aim of the trial is to assess whether the MENISCUS intervention improves educational attainment, mental health symptoms and related health- and well-being outcomes among girls in secondary school in Uganda.

## PRIMARY OBJECTIVES

To evaluate whether the MENISCUS intervention i) improves educational attainment and ii) reduces mental health symptoms (primary outcomes) among female secondary students in Uganda.

## SECONDARY OBJECTIVES

1. To evaluate whether the MENISCUS intervention improves:
2. knowledge of puberty and menstruation; attitudes towards menstruation (girls and boys);
3. menstrual practices at last menstrual period (LMP);
4. pain management during LMP;
5. self-efficacy of MH;
6. quality of life measurement, valuation and happiness;
7. prevalence of urinary tract infections (among post-menarchal girls)
8. school and class absence during menses;
9. school and class absence overall;
10. self-confidence in maths and (separately) science abilities

Objectives vii-viii will be assessed in a subsample only.

Exploratory outcomes: To evaluate the proportion of girls who drop out of school between baseline and endline and educational attainment in each subject separately (biology, maths, and English) in girls as assessed by UNEB

1. To evaluate the costs of setting up and running the intervention package, the unit cost per student reached, and the incremental cost-effectiveness of the intervention per unit increase in selected policy-relevant outcomes, relative to optimised usual care.
2. To assess whether the intervention was implemented with fidelity, and to understand the contextual factors affecting implementation, the acceptability to participants, and the intervention mechanisms. We will achieve this through a process evaluation including quantitative indicators and qualitative data collected from in-depth interviews and focus group discussions.
3. To assess the policy environment around menstrual health in Uganda, focusing on how implementing the intervention contributes to, and aligns with, the attainment of the Government policy objectives on menstruation management in schools. We will conduct an assessment of the policy/regulatory frameworks to which the outcomes of the intervention contribute, identify the supportive and constraining factors to the implementation of the policy guidelines and how the findings of the intervention inform refinement of current policy.

# STUDY DESIGN

This study will be an open-cohort cluster randomised control trial with 60 schools^[[3]](#footnote-4)^ (clusters) randomised 1:1 to 2 arms, with a mixed-methods process evaluation, and economic and policy analyses to evaluate the effectiveness and cost-effectiveness of a school-based MH intervention to improve education, health and wellbeing outcomes. Use of an open-cohort design allows students entering schools after baseline to participate in the endline survey.

The trial timeline is shown in Figure 3. The total duration of the study is 36 months. At baseline, approximately 4,500 participants starting S2 in 2021 will be recruited (~3600 females and ~900 males) through a cross-sectional survey. The expected median age of participants is ~15 years.

The intervention will be implemented over the duration of one year in all intervention schools (N=30).

At endline (2023), outcomes in all students in the class cohort expected to be in S3, who were present at baseline or joined during the intervention year, will be assessed in a repeat of the cross-sectional survey.

Selected secondary outcomes will be assessed in a randomly-selected sample of approximately 1500 girls identified ahead of the endline survey.

Control schools (N=30) will be offered the intervention after the endline evaluation is completed.

Figure 3: Trial timeline


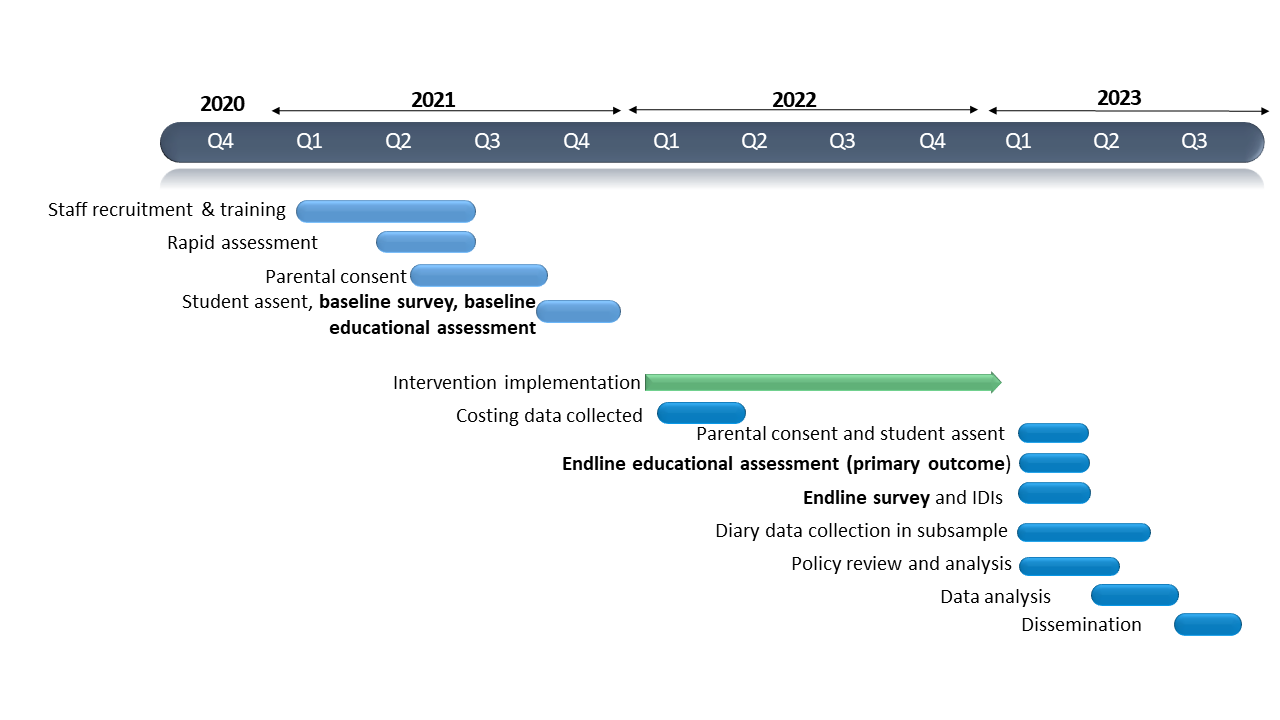


**STUDY SETTING**

The trial will be conducted by scientists at the London School of Hygiene and Tropical Medicine (LSHTM) in London, and the MRC/Uganda Virus Research Institute (UVRI) and LSHTM Uganda Research Unit in Entebbe. The implementing partner is WoMena Uganda in Kampala.


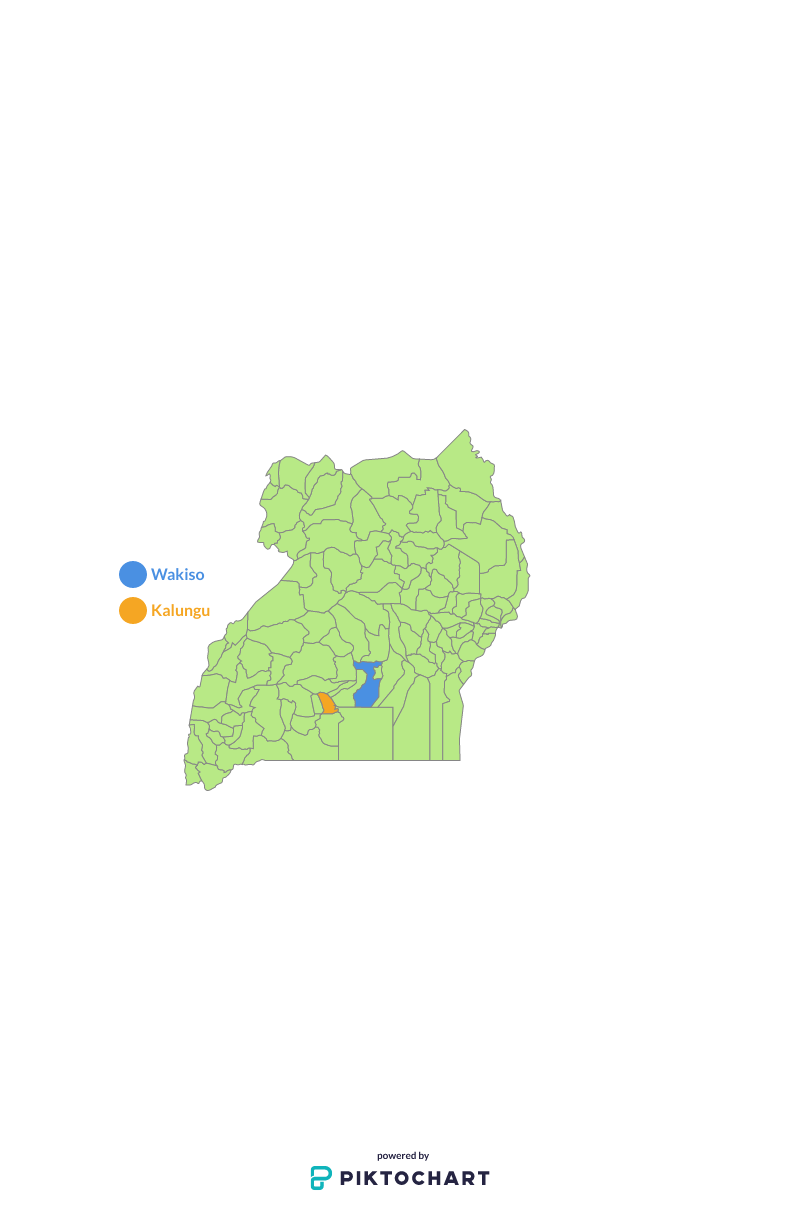


Figure 4: Map of Uganda - Kalungu and Wakiso districts

The study will be delivered to secondary schools in Wakiso and Kalungu Districts in the Central Region of Uganda. The MRC/UVRI and LSHTM Uganda Research Unit in Entebbe will be the primary research base for the MENISCUS trial. The Unit has a field station in Kalungu District, established in 1989 (around 120 km south west of Entebbe) with administrative offices, laboratories and a study clinic. The field station(25) will be the base for the MENISCUS field team in Kalungu District.

In 2007, Uganda became the first country in sub-Saharan Africa to introduce universal secondary education (USE), following success in improving access to primary education(26). However, secondary school attendance remains low (estimated at 24% in 2016). Most secondary schools in Uganda are private schools, and the USE policy included the introduction of a Public-Private Partnership (PPP) under which the Government transfers a subsidy to private USE schools. A review published in 2018 estimated that about 1/3 of all students enrolled in a secondary school in Uganda are part of the PPP scheme (26).

Wakiso District has a total area of 2,704 square kilometres and partly encircles Kampala (Uganda’s capital city), and borders Lake Victoria. The population in 2014 was estimated as 2.00 million, more than double the estimated population size of 0.9 million in 2002. Of these, an estimated 446,195 (22.3%) were aged 10-19 years, and 48.9% of 13-18 year olds attended secondary school (twice the national average). There are an estimated 500 secondary schools in Wakiso District(27). Our formative work was conducted in Wakiso District, and the menstrual health intervention was received enthusiastically by the District Education Officer and the schools we collaborated with.

Kalungu District is largely rural with an estimated population of 353,825 in 2014, of whom an estimated 93,000 (26.2%) were aged 10-19 years, and 36% of 13–18-year-olds attending secondary school. There are an estimated 46 secondary schools in the district (28). Our preliminary discussions with the District Inspector of Schools indicated that there were no MH programs in the district but that the study would be welcomed. This was supported by views from the heads of two schools we visited in 2019.

## STUDY OUTCOME MEASURES

### Primary outcomes measures

The primary outcomes are educational attainment and mental health symptoms among all girls included in the endline survey (Table 2). These are the primary hypothesized, and complementary, outcomes in our theoretical framework (Fig 2). Educational attainment and mental health symptoms will be assessed in girls only because we do not hypothesize an intervention effect in boys.

**Educational attainment** of girls will be independently and objectively evaluated by assessing the maths, English and biology curricula taught during the intervention implementation year. Two examinations will be independently developed, pre-tested, administered and marked by the Uganda National Examination Board (UNEB). One will test material taught in 2020 (administered at baseline), and the other will test material taught in 2022 (administered at endline). UNEB assessors will be masked to trial arm. The outcome will be the composite score of these subjects at endline, adjusted for baseline score using mixed-model repeated measures to increase precision and allow for missing data. The rationale for the subject choice is that the biology curriculum includes puberty knowledge and is directly related to our intervention, and English and maths are the subjects recognised by policymakers as influential for scale-up.

**Mental health** **symptoms** will be assessed in all girls at baseline and endline using the Total Difficulties Scale of the Strengths and Difficulties Questionnaire (SDQ-25). This includes emotional symptoms, attention, and peer-relationship problems which the intervention aims to improve. The SDQ-25 has been widely validated among adolescents in sub-Saharan Africa(29) and was used in our pilot (Section 2.4) in which there was a reduction in SDQ-25 score in girls following the intervention (standardised mean difference=0.20; p=0.006)(22).

### Secondary outcomes

The secondary outcomes (Table 2) are:

1. knowledge of puberty and menstruation; attitudes towards menstruation (among all girls and a random sample of boys); and (among all girls):
2. menstrual practices at last menstrual period (LMP);
3. practice of pain management during LMP;
4. self-efficacy in addressing menstrual needs experiences at LMP;
5. quality of life measurement, valuation and happiness;
6. prevalence of  urinary tract infections (UTIs) among post-menarchal girls;
7. proportion of school days and classes missed during period-days after one year of the intervention (among sample of ~1500 girls);
8. proportion of all school days with school and/or class absence (regardless of menstruation) (among sample of ~1500 girls).
9. Self-confidence in maths and (separately) science abilities

### Exploratory outcome

i) Proportion of girls who drop out of school between baseline and endline.

ii-iv) Educational attainment in each subject separately (Biology, Maths, and English) in girls as assessed by UNEB

Table 2: Impact outcome measures

| **Primary Outcome** | **Measure** | **Variable type** | **Data source** | **Analysis population** |
| --- | --- | --- | --- | --- |
| 1) Educational attainment | Performance on English, Mathematics, and Biology (weighted equally) taught during the intervention year, using item response theory. | Continuous | UNEB examination | All female participants |
| 2) Mental health problems | Mean Total Difficulties Score from the Strengths and Difficulties questionnaire (SDQ-25) (30) | Continuous | Endline survey | All female participants |
| i) Knowledge of puberty and menstruation; attitudes towards menstruation | - Number of knowledge items answered correctly (out of 9). - Number of attitudes items with positive responses (out of 3) | Count | Endline survey | All female and male participants (stratified by gender) |
| ii) Menstrual practices at last menstrual period (LMP) | - Proportion using adequate menstrual materials that are appropriately cleaned or disposed of at LMP^1^ - Mean score on Menstrual Practice Needs Scale (MPNS)(31) | Binary  Continuous | Endline survey | Post-menarchal female participants |
| iii) Pain management at LMP | - Proportion who used an effective pain management method (at least 1 of 6 effective method and none of the 4 ineffective methods listed^2^) | Binary | Endline survey | Post-menarchal female participants who report pain at LMP |
| iv) Self-efficacy of MH | - Mean score on Self-efficacy in Addressing Menstrual Needs Scale (SAMNS)(32) | Continuous | Endline survey | Post-menarchal female participants |
| v) Quality of life | - CHU9D score, calculated as a weighted sum using adolescent utility tariffs (33) | Continuous | Endline survey | All female participants |
| vi) Prevalence of urinary tract infection (UTI) | - Proportion with a UTI, defined as at least one genital symptom plus leucocyte esterase and/or nitrates with a urine dipstick test | Binary | Urine Multistix 8 dipstick | Post-menarchal female participants |
| vii) School and class absence during menses | - Proportion of full school days missed by girls during their period^3^ (adjusted for absence on non-period days) - Proportion of school days with lessons missed by girls during their period (adjusted for absence on non-period days) | Binary  Binary | Daily diary | Random subsample of ~1500 post-menarchal female  participants |
| viii) School and class attendance overall | - Proportion of full school days missed - Proportion of school days with lessons missed | Binary  Binary | Daily diary | Random subsample of ~1500 post-menarchal female  participants |
| ix) Self-confidence in Mathematics and science abilities | - Mean score on the Students Confidence in Mathematics scale (34) - Mean score on the Students Confidence in Science scale (34) | Continuous  Continuous | Endline survey | All female participants |

# Selection and withdrawal of PARTICIPANTs

##

## Pre-randomisation or pre-registration evaluations

In year 1, we will conduct awareness-raising of the trial in secondary schools in the two Districts and assess schools for eligibility. A rapid assessment (previously submitted for ethical approval) will be conducted over a 2-week period per school in Q2, 2021, to obtain data that will inform differences about the school environment in relation to policies and programmes that can influence MH, and factors to be randomised on including potential confounding factors i.e. school examination results, school type (government/private), current WASH facilities, implementation of Government MHM guidelines and area (urban/rural).

For intervention schools, the results will be used to assist schools in identifying additional community gatekeepers and key influencers in the school community to form the MH Leadership Group responsible for maintaining the intervention.

## Inclusion Criteria

**Inclusion criteria for schools:**

- Mixed-sex secondary schools with S1-S3 classes
- Day, or mixed day/boarding schools
- At least minimal WASH facilities, including an improved water source and sex-specific sanitation facilities that are functional, usable, and accessible to female students at the time of the survey.^[[4]](#footnote-5)^
- Estimated enrolment of ~50-125 female students in S1 as of January 2020 in Wakiso, and ~40-125 female students in S1 in Kalungu (to be refined following the rapid assessment and estimated school enrolment following COVID-19 school closures).

**Inclusion criteria for trial participants**:

- All female students starting S2 during 2021^[[5]](#footnote-6)^ who are present and whose parents have given consent will be eligible for the baseline survey and to receive the intervention (the baseline survey will be conducted pre-randomisation in each stratum of schools, over a 6-8 week period).
- A simple random sample of ~15 male students (starting S2 during 2021) per school (900 total) who are present during Q4 2021 will be selected to participate in the baseline survey and asked to provide assent for data collection. The sampling will be done by the trial statistician using the random-number generator in Stata.
- The endline survey and assessment will be in Q1-2 2023, and all female students present and who have given consent/assent for the research will be eligible, as will the male students with baseline data.

**Inclusion criteria for the diary sub-study**

- A simple random sample of 1500 female students (~25 per school) in both arms will be randomly selected (by the trial statistician using a random-number generator) ahead of the endline survey to participate in the diary sub-study.

## EXCLUSION CRITERIA

**Exclusion criteria for schools:**

- Schools that are currently participating in a menstrual health related programme.
- Exclusively boarding schools
- Boys- or girls-only schools
- Schools exclusively for students with disabilities.
- Schools where more than approximately ~10% of students or parents of students do not understand Luganda or English.

#

# RANDOMISATION, masking AND ENROLMENT PROCEDUREs

## ENROllment / Registration PRACTICALITIES

### Recruitment of schools

We will recruit schools in each District following a Rapid Assessment (UVRI REC GC/127/20/01/811) which will involve a desk review of secondary schools in each district, a stakeholder meeting with the District Education Officer, all Head Teachers and local MoES officials to explain the trial, and visits to potentially eligible schools (Q2 2021) to assess eligibility and seek approval of from head teacher to participate.

### Recruitment of trial participants

During the rapid assessment we will obtain a listing of students due to start S2 in 2021 for the 60 eligible schools. Parent/caretaker consent for female students’ participation in individual-level intervention components and data collection will be at a series of information meetings with parents/caretaker and students in the study schools, and will include follow-up contact or home visits if parents/caretaker do not attend (September-December 2021). As recruitment can be negatively affected by school holidays and other events, potentially including COVID-related closures, the timing of the baseline activities and trial start will be closely coordinated with the school academic timetable and head teacher liaison to minimise unexpected delays.

## Randomisation

The 60 selected eligible schools will be randomised 1:1 using restricted randomisation within strata. The allocations will be restricted to minimise imbalance by potential confounding factors assessed during a rapid assessment prior to the start of the study and the baseline survey (i.e. school examination results, school type, mean SDQ score, current WASH facilities, and geographic area). These factors will be verified during a rapid qualitative assessment in Q2 2021 using participatory methods to gather information on the setting. Eligible allocations will be sent to the data manager by the trial statistician, and one allocation will be selected at semi-public randomisation ceremonies held for each stratum of schools following completion of the baseline survey, in the presence of representatives from schools, the District Education Offices, the MoES and MoH.

## MASKING

We will ensure allocation concealment and prevent biased recruitment by conducting the randomisation immediately after the baseline survey in each stratum (Q4 2021)(36). Masking of participants, school staff and implementers is not possible as it will be clear whether the intervention is being implemented. The primary outcome of educational assessment will be masked as it will be independently administered by UNEB with no knowledge of intervention arm. We will minimise bias in other outcome assessments by separating the implementation (by WoMena Uganda) from the outcome assessment (MRC/UVRI). Students will know from the information and consent forms that the study aim is to improve menstrual management but information on the specific education and health symptom outcomes will not be given, to minimise biased reporting.

Systems for reporting suspected unexpected serious adverse reactions (SUSAR) and serious adverse reactions (SAR) will, as far as possible, maintain masking of the clinical officer and trial manager. Further details of the unmasking process for SUSAR and SAR are given in Section 8.

The independent statistician will produce reports for the Independent Data Monitoring and Ethics Committee (IDMEC) to preserve masking of the trial statistician. All data will be anonymised, analysed and interpreted masked to allocation information. We will minimise bias due to attrition for the outcomes using mixed-models for repeated measures (MMRM)(37) and will compare reasons for students leaving school, and baseline characteristics associated with dropout, between arms.

## 5.4 CONTAMINATION

It is possible that some contamination between the control and intervention arm schools may arise if, for example, students change schools or head teachers share information. However, the intervention includes physical components based at the school, such as basic improvements to toilet and sanitation facilities and provision of painkillers. It is also unlikely that other components, such as the drama skit, would be delivered in control schools, even in the event of some students changing schools. We therefore expect contamination to be minimal, and will monitor and evaluate it through the planned process evaluation (described in section 9.4 below).

# Intervention

## Optimised usual care

Control arm schools will receive only the MoES Guidelines on Menstrual Hygiene Management (MHM) in Schools (circular No. 1/2015) and the 2018 National Sexuality Education Framework (NSEF)(38). Students will receive the Government Menstruation Management Reader. The Guidelines recommend provision of clean, private, toilet facilities, regular supply of water and soap, emergency supplies of pads and painkillers, training of teachers, health assistants and inspectors, involvement of parents in supporting and providing MH information and materials. The Guidelines should be circulated to all schools but are often not implemented^10^. This provision is thus optimised Usual Care (oUC). The MENISCUS intervention will be offered to control arm schools after the endline survey (Q2-3 2023).

## Intervention components

Intervention arm schools will receive the oUC plus the MENISCUS intervention (Figure 5). The intervention will be overseen and delivered by the implementation partner, WoMena Uganda. Each intervention school will be asked to sign a Memorandum of Understanding covering all 5 intervention elements, and to develop an **MH Action Group** (of ~6-10 teachers, parents, prefects and students) responsible for ensuring that each element of the intervention is delivered, maintained and sustained. The intervention will be delivered over one year, starting in 2022, to the class of students who were due to start S2 in 2021.

Figure 5: MENISCUS intervention package


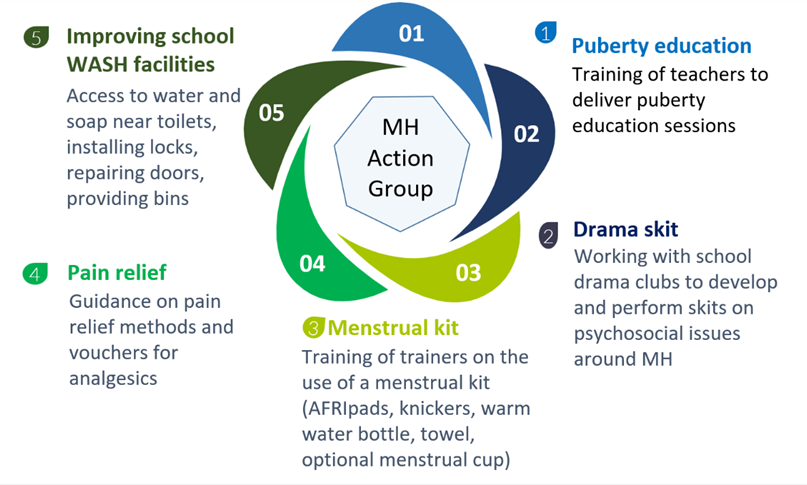


1. **Puberty Education Workshop:**

At the start of intervention delivery, WoMena Uganda will hold participatory workshops to train male and female teachers of Secondary students who deliver Government puberty education (including MH) bringing together teachers from the 30 intervention schools. The first session (2 days) will include an introduction to the intervention, discussion of current materials, and advice on delivering the Government MHM Guidelines. At session 2 (1 day), the teachers are taught how to deliver the session. They will practice with WoMena Uganda, who will provide feedback and recommendations. Teachers will be asked to develop an action plan, delivery of which will be overseen by the MH Leadership Group and the District Inspector of School.

1. **Drama skit**

MENISCUS-1 and 2 demonstrated the key role that development and performance of a drama skit has on providing ownership of good MH messages by male and female students and teachers. This component was popular and informative for parents and the school community. Facilitation sessions led by WoMena Uganda and a drama consultant will introduce the content (strategies for pain management, preventing and managing teasing, using diaries to monitor the menstrual cycle, and using a menstrual materials). Students will be asked to develop a title, storyline and dialogue, and to rehearse the skit. WoMena Uganda will attend a rehearsal of the adapted skit and provide feedback. This will be followed by subsequent rehearsals with further feedback. The students will perform the skit at the annual parents’ day or another suitable event to which parents and the school community are invited.

1. **Provision of an MH kit**

WoMena Uganda will train trainers to use a kit containing re-usable Ugandan-manufactured sanitary pads, provided in a bag including underwear, a water bottle (for pain relief), soap and a towel. Trainers will be selected from the MH Action Group and other motivated teachers and prefects, and will be responsible for training students to use the products, under WoMena Uganda supervision. Trainers and MH Action Group members will receive MH kits themselves. Training includes an education session on menstruation for girls and boys, and a gender-specific participatory session. The girls’ session includes how to use the MH kit components. The training will also include recognising signs of RTIs and other infections and safe use of painkillers. The boys’ session will include male puberty, genital hygiene, circumcision, and attitudes towards menstruating girls. Intervention schools will be encouraged to arrange additional education sessions for those students who are absent on the initial training days. Up to 3 follow-up sessions will be provided by WoMena Uganda as needed to provide additional guidance to trainers.

- 1. **Reusable sanitary pads:** We will provide all female participants with 5 to 6 reusable pads (AFRIpads), which are quality certified by the Uganda National Bureau of Standards (UNBS) with Standard US 1782: 2017. AFRIpads are designed to last for over 12 months. Girls will be trained in safe use and care of reusable pads. See Annex 5a for the product brochure and Annex 6a for safe use and care information.
  2. **Optional Menstrual Cup:** Girls will have the option to receive a medical-grade silicone, re-usable menstrual cup (Ruby Cup) with a container for disinfection and storage. A separate information and consent/assent form (Annexes 35-36) will be provided for the menstrual cup, so it is possible for parents/students to agree to all parts of the intervention without agreeing to receive the cup. The cup is available in two sizes, girls will receive guidance on selecting the correct size for them. Girls will take part in an additional training session on how to use the Ruby Cup, including guidance on proper techniques for insertion and removal as well as how to follow the hygiene procedures for safe use. Girls will also receive further information on recognising any potential adverse reactions to the menstrual cup and will receive contact details for WoMena staff and for the Marie Stopes toll-free Hotline number for advice and support. Eight mothers or female caregivers per intervention school will also receive a MH Kit including the menstrual cup to try and training in its safe use and care. WoMena will attend follow-up visits (up to 3 visits as needed) to provide additional guidance to the trainers. Trainers will be encouraged to arrange support meetings or follow up with menstrual cup recipients to ensure they have access to support throughout the intervention. Girls who have lost their cup due to misplacing it, theft or damage will receive a replacement cup. By supplying girls with both pads and a cup, they will be able to use the re-usable pads if they are not comfortable using the cup. See Annex 5b for the product brochure and Annex 6b for safe use and care information.

1. **Pain relief:** Our pilot work showed that 73% of girls reported pain at LMP (22). This was a major contributor to absenteeism and attention in class. WoMena Uganda will educate girls on effective period pain management (warm water bottles, stretching), and addressing common myths (e.g. that painkillers cause infertility). Vouchers for analgesics will be included in the MH kit, each to be redeemed for 6 paracetamol or ibuprofen tablets per month from the school nurse or specified teacher. See Annex 6c for safe use of analgesics information. School nurses will receive training on the administration of painkillers in exchange for vouchers and on identifying and referring any adverse reactions to the study clinician.
2. **Improving School WASH facilities:** Functional single-sex toilets are necessary for adequate MH(35). At baseline, the MENISCUS intervention includes sustainable measures to improve school WASH facilities (fixing doors, providing locks, sanitary bins, toilet paper cages, water carriers, liquid soap (5L), and water (20L) containers). The MH Leadership Group will be responsible for identifying the optimal method to keep the toilet and pit latrine areas clean, and for maintaining WASH supplies throughout the intervention period, monitored by the District Inspector of Schools during their routine termly school inspection visits.

## PRETEST OF DELIVERY APPROACH

To refine the training of trainers (ToT) delivery model for the MH kit and role of the MH Action Groups, we will conduct participatory workshops with relevant school staff in two pre-test schools not selected for the main trial. This work will take place ahead of intervention rollout in the trial schools and will help ensure quality delivery.

**Schools selected for pre-test**: A purposive sample of one school in each District will be selected for this pilot study. The schools will be selected from those included in the Rapid Assessment but excluded from the trial due to existing participation in a MHM project. These schools will have participated in the stakeholder workshops described in section 12.2.2 and the lessons learnt from previous MHM implementation in their schools will assist refinement of our delivery model.

**Procedures**: At each of the two selected schools, up to 10 school staff will be invited to participate in a workshop. The participants will be selected by the schools with guidance from the WoMena team and will be school staff with relevant experience i.e. teachers who teach puberty education, teachers involved in MHM interventions in the school, senior teachers, school nurses.

WoMena Uganda staff will facilitate one workshop per school with participants using a discussion guide (Annex 47). These will be 3-hour workshops to discuss strategies to strengthen implementation of the ToT model and Action Groups and to gain feedback on training materials. For example, participants will be asked to give insights into the needs of teachers for implementing the proposed intervention and how to effectively train teachers to deliver training to students. Participants will be given samples of the resource materials and asked for feedback on the challenges and opportunities for implementation and any potential areas for improvements.

WoMena facilitators will include the WoMena Project Manager, Project Officers and MHM trainers, all of whom have training and experience in qualitative research and/or facilitating workshops. Written informed consent will be sought from all participants (school staff) prior to their taking part in the workshop, following the procedures in section 12.2. Notes will be taken in paper format and will be collected and stored in accordance with the Data Management Plan (Annex 2).

## Stakeholder’s engagement

A Stakeholders’ workshop will be held in 2023 to disseminate findings and discuss policy implications with representatives from the MOES and MOH. See section 14 for further details on stakeholder engagement throughout and after the study. We will also seek to engage the existing MRC/UVRI and LSHTM Community Advisory Boards in Wakiso and Kalungu for feedback on trial plans and results interpretation.

## Adherence to study intervention protocol and assessment of compliance

Based on our pilot, we anticipate high levels of uptake of the individual and behavioural components (puberty education, drama skit, MH kit, and pain management), and potentially lower compliance with the WASH component. To further improve school-level compliance of the WASH component, the MH Leadership Groups will be tasked with compliance for this component, with monitoring from the District Inspector of Schools. We will evaluate school-level compliance as part of the process evaluation (section 9.4) and individual-level exposure in the process evaluation and in the endline survey.

## Name and description of each non-investigation medicinal product (NIMP)

The NIMPs in this study are paracetamol (500mg per tablet) or ibuprofen (200mg per tablet) per month which girls can obtain in exchange for vouchers from the school nurse or local pharmacy. The chosen doses are lower than the maximum recommended dose (4000mg/day for paracetamol and 3200mg/day for ibuprofen). Adverse effects of Paracetamol are rare but hypersensitivity including skin rash may occur. Commonly reported side effects of ibuprofen include: haemorrhage, vomiting, anaemia, decreased haemoglobin, eosinophilia, and hypertension.  Girls will be taught about appropriate analgesic use during the training sessions and will be advised to see the school nurse or contact a member of the study or implementation team, if they notice adverse events. Students will receive training in recognising adverse reactions related to painkillers.

# RIsks and BENEFITS

The trial will adhere to the principles of Good Clinical Practice (GCP) and the MRC guidelines for management of global health trials. The rights and safety of trial participants are our primary concern. In this section we summarise anticipated risks and benefits to trial participants. Further details are provided in Annex 3.

## Risks

### Participation in the research data collection

**Risks to students for specific research components:** There is a risk of embarrassment in answering questions about periods and teasing of female students taking part in the daily menstrual diaries. To mitigate this, teachers will be sensitized to reduce possible embarrassment of girls completing research activities.

**Urine sample:** At endline, all participants will be asked about urinary tract symptoms.. Girls with at least one symptom of a urinary tract infection (UTI) will be asked to provide a urine sample for testing and the test results will be included on the referral form to the local clinic. There is a risk that girls may feel embarrassed answering questions about genital symptoms. Girls may also feel embarrassed going to the clinic for urinary tract symptoms, especially if the clinics are not adolescent-friendly. To mitigate the risk of embarrassment, a self-completed form on a tablet will be used, and we will provide the girls with clear instructions using illustrations on how to effectively take a urine sample. Operational procedures for referral to local clinics will be established by the full-time MENISCUS clinical officer, and agreed with district medical officers. Local clinics will receive training for syndromic management and on working with adolescents in an appropriate manner if necessary, and stock outs of essential medicines will be monitored.

### Participation in the intervention

The head teacher provides agreement for the school-level activities for girls and boys (puberty education, drama skit and WASH components) and we don’t anticipate any risks associated with these. The provision of the MH kit and of analgesics to girls have potential risks and require individual-level parental consent and student assent.

**Provision of menstrual products (Reusable pads and optional menstrual cup):** Reusable pads are widely used and there is no evidence that they are associated with reproductive tract infections (RTIs) or other urogenital infections, although few studies examined this robustly (9,39) and it is plausible that there are risks if the pads are not washed and dried correctly. The menstrual cup is made from flexible medical-grade silicone that collects menstrual blood. A recent systematic review concluded that menstrual cups are an effective and safe alternative to other menstrual products (40). However, initially, participants may feel discomfort when inserting and removing the cup, and wrong placement may cause soreness until the participant becomes proficient in use. Based on previous studies, we expect this to improve over several months, as girls become more accustomed to using the cup (41–43). To mitigate the risk of pain, discomfort, leakages and incorrect use, training from WoMena Uganda will include information on how to use the reusable pads and the cup, including guidance on the hygiene procedures for safe use and for menstrual cups, proper techniques for insertion and removal. Schools will be encouraged to ensure support on product use is accessible to students throughout the intervention.

**Risk of infection:** Menstrual toxic shock syndrome (mTSS) is a rare condition with a risk of 1-16 cases per 100,000 women years (44), and has been associated with the use of high-absorbency tampons and continual usage. It is caused from vaginal colonisation with a toxin-producing strain of *Staphylococcus aureus*, called TSST-1. We anticipate low levels of tampon use in the trial, based on our formative data. In a systematic review of 43 studies, use of the menstrual cup showed no adverse effects on the vaginal flora and the authors concluded that data showed no reason for concern about TSST-1 among cup users(45). A recent study among school girls in Kenya found no cases of TSS-1 toxin associated with menstrual cup use (43). Although the risk is negligible, participants will be informed of the risk of TSS-1 and we will monitor for adverse reactions and events with advice on rapid access to tertiary care in case of this rare event (Section 8).

**Socio-cultural risks**: In prior research, we invited 40 parents (33 females, 7 males) from 9 schools in Wakiso District to participate in group discussions to assess their perceptions on the acceptability of the menstrual cup among their daughters. Only 2 parents had heard of the menstrual cup before our team introduced it. The key concerns were its size, possibility of infections and concern that the cup would affect girls’ virginity. Most parents (25/40) said they would allow their daughters to use the cup if girls were first provided with the necessary information, and if the mothers also had the opportunity to try the cup. Based on this, we will offer the cup with an additional opt-in informed consent process, so that if the parent and girl do not wish to receive a cup, they can still receive the other intervention components and participate in the study. We will also explicitly address common concerns about use of the cup in our education sessions. A small group of mothers or female caregivers per intervention school will also receive a menstrual cup as part of the MH Kit for personal use with training on its safe use and care and other mothers and female caregivers will be provided with information on where to buy a cup for themselves to use.

### COVID-19 related risks

All research activities will be carried out in accordance to COVID-19 safety guidelines issue by the MRC/UVRI and LSHTM Unit and UNCST(46). Staff will be trained on how to conduct research activities to limit COVID-19 related risks in accordance with the COVID-19 Risk Management Plan (Annex 4).

Due to COVID-19 restrictions, schools in Uganda have been largely closed since March 2020. As a result of COVID-19 disruptions, some schools have permanently closed, some may reopen with a smaller student population and students may face increased challenges attending school and paying school fees. Students have also been absent from school for a substantial time, which could impact students’ performance and progression between classes. As part of a Rapid Assessment (previously submitted for ethical approval) to prepare for the trial, we will assess the impact of COVID-19 on eligible schools. Our primary outcome, educational attainment, is assessed through bespoke examinations set by the Uganda National Examinations Board (UNEB) and will examine the specific material taught during the year prior to the baseline and endline surveys, respectively, even if this differs from the material originally planned to be taught. If schools do remain closed for longer periods, we will consider delaying the start of the baseline survey and intervention delivery.

## Benefits

All schools will be provided with the current Government circular with instructions for improving MH (circular No. 1/2015). Girls and boys in the intervention arm schools will be exposed to increased education about puberty and menstruation, and improved WASH facilities. Girls will have training and access to menstrual products and pain relief strategies. Based on our pilot work, we anticipate that these exposures will improve self-efficacy to manage menstruation, and lead to an improved social and physical environment around puberty, menstrual health, sanitation and illness management. Teachers and parents will benefit from increased information about MH and changes in the social environment. Pupils and teachers in schools in the control arm will be offered the intervention after the endline survey has been completed. Participants will also be taught how to use a tablet computer to answer survey questions. Despite this, some girls and boys may experience no individual benefits for participating in this study.

# safety reporting for non-drug trials

##

## Definitions

*Table 3: Definition of adverse events and reactions*

| **Term** | **Definition** |
| --- | --- |
| Adverse Event (AE) | Any untoward medical occurrence in a patient or study participant. The medical occurrence does not necessarily have a causal relationship with this treatment. |
| Serious Adverse Event (SAE) | A serious event is any untoward medical occurrence that:  Results in death  Is life-threatening  Requires inpatient hospitalisation or prolongation of existing hospitalisation  Results in persistent or significant disability/incapacity  Consists of a congenital anomaly or birth defect  All incidences of Toxic Shock Syndrome  Other ‘important medical events’ may also be considered serious if they jeopardise the participant or require an intervention to prevent one of the above consequences. |
| Serious Adverse Reaction (SAR) | An adverse event that is both serious and, in the opinion of the reporting investigator, believed with reasonable probability to be due to one of the trial treatments, based on the information provided. |
| Suspected Unexpected Serious Adverse Reaction (SUSAR) | A serious adverse reaction, the nature and severity of which is not consistent with the information about the medicinal product in question set out:  In the case of a product with a marketing authorisation, in the summary of product characteristics (SmPC) for that product |

## Intensity

The intensity of each AE recorded in the case report form should be assigned to a grade (1-5), which will be determined following the definitions set forth in the Common Terminology Criteria for Adverse Events v5.0 (CTCAE) (47) Use of these standardized guidelines will allow for uniform reporting. The grades are defined as follows:

- Grade 1: Mild AE
- Grade 2: Moderate AE
- Grade 3: Severe AE
- Grade 4: Life-threatening or disabling AE
- Grade 5: Death related to AE

## Reporting Procedures

The principles of ICH GCP require that both investigators and sponsors follow specific procedures when notifying and reporting adverse events (AEs) or reactions in clinical trials. AEs and serious adverse events (SAEs) will be monitored, managed and recorded during the study (Annex 26 and 27).

### Non-serious AEs

In the intervention arm schools, AEs identified by the study clinician, school nurse, or a member of the study or intervention team at follow-up visits and from the endline survey will be assessed by the clinical officer. All suspected AEs will be noted in the Adverse Events Log (Annex 26) after assessment. The clinical officer will review these events to determine a possible relationship with the interventions and the occurrence of each AE. The study and intervention team and school nurses will receive training in referring adverse events to the clinical officer. Mild adverse events will be noted in the participant’s case report form. AEs will be reported annually in an aggregated report. AEs that will not be reported include common illnesses that do not result in hospitalisation, including but not limited to respiratory, gastrointestinal, and skin diseases, unless they are considered at least possibly related to the intervention.

### Serious AEs

In both arms, in the case of any severe adverse event (such as TSS, or severe violence), participants will be rapidly referred to the hospital for management. Transportation to the hospital will be provided. All hospitalised participants will undergo record review to identify potential adverse consequences of study participation.

The MENISCUS clinical officer (Ssenyondwa) will be responsible for initially assigning causality. All SAEs will be reported to the study co-ordinator (Kansiime), in-country PI (Kyegombe ) and trial PI (Weiss) within 24 hours of the staff becoming aware of it, using an SAE form (Annex 27). SAEs that are unexpected and are at least ‘possibly related’ to the study interventions (analgesic use or the menstrual cup) will be reported to the IDMEC within 24 hours of the PI becoming aware of it. An SAE form will be completed and submitted to the study coordination centre with as much detail of the event that is available at that time. If awaiting further details, a follow-up SAE report will be submitted promptly upon receipt of any outstanding information. Any events relating to a pre-existing condition or any planned hospitalisations for elective treatment of a pre-existing condition do not need reporting as SAEs.

At endline, all girls in the trial will be advised about symptoms of urinary tract infections. All girls who self-report UTI symptoms at endline, and have a positive Multistix 10 test result for leucocytes or nitrites will be referred to a local clinic for syndromic management as per Ugandan Clinical Guidelines. In addition, the full-time MENISCUS clinical officer will provide training to the school nurses on symptom identification and referral.

Although extremely rare, surveillance of TSS among girls who are provided cups in the trial is warranted. TSS will be monitored at school level by trained nurses and/or staff for early detection of signs and symptoms, and a management pathway will be defined for each school by the Clinical Officer (Figure 6). To facilitate this, participants, families and the MH Leadership Group will receive information leaflets describing signs and symptoms of TSS (Annex 8a), where to seek emergency care, and contact information. School nurses and local clinical staff will be provided with information about TSS by the MENISCUS clinical officer (Annex 8b). Any verified case of TSS will be reported to the IDMEC and any that are among participants provided with menstrual cups will also be reported to the cup manufacturer. Any SAEs in participants in any group will be reported to the IDMEC.

Figure 6: Management of suspected TSS
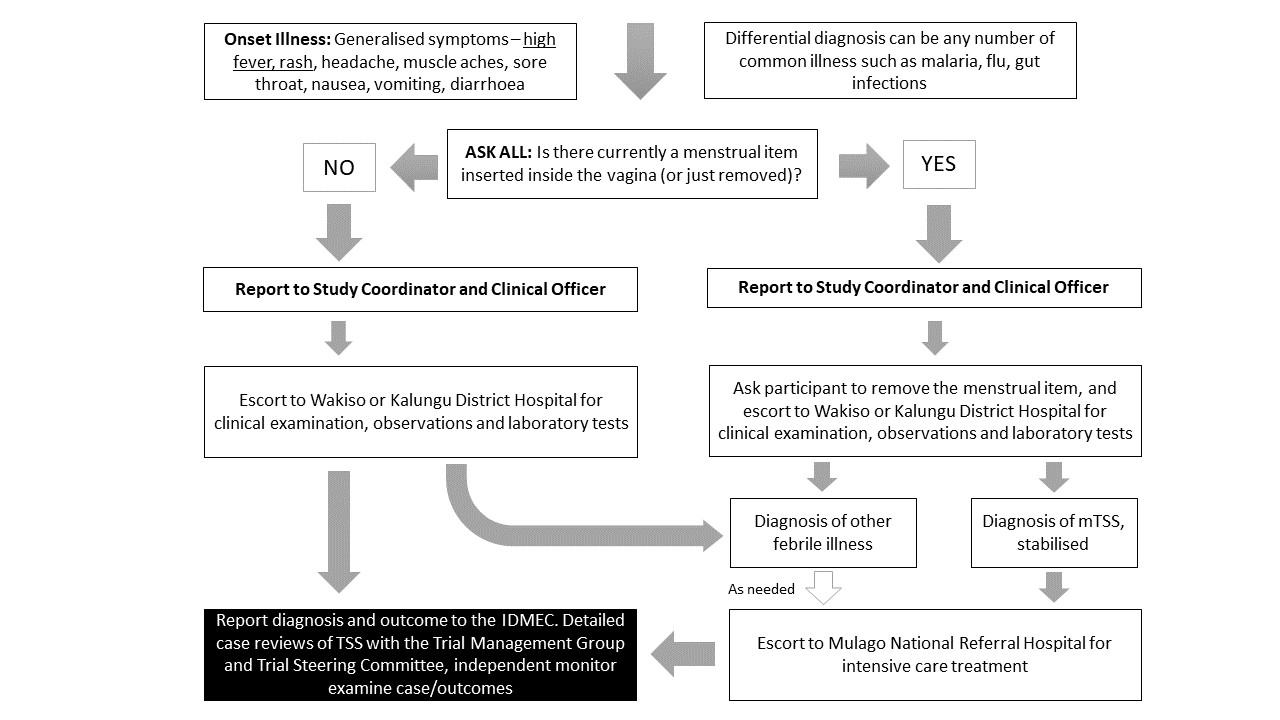


As stated above, at the beginning of the study, senior study staff will meet with the district medical officers to confirm operational procedures for referral to local clinics. Local clinics will be visited by the clinical officer who will provide any training needed and her contact information. Girls will also be provided with the toll-free number for the Marie Stopes Uganda contact centre (phone: 0800 220 333; whatsapp: 754001 503), which operates 7 days per week and is managed by a team of 12 qualified counsellors and 2 nurses to provide confidential advice on sexual and reproductive health and referral to services in Luganda and English. Over 10,000 calls are received each month with 60% of those from young people (15-24 years). WoMena Uganda have previously trained contact centre staff on menstrual health, use of menstrual cups, and TSS.

# ASSESSMENT AND FOLLOW-UP

## INTERVENTION IMPACT ASSESSMENT

### Baseline assessment of self-reported outcomes

All participants with parental consent and student assent will be provided with a Trial ID card and asked to complete the baseline survey in Q3 2021. We estimate there will be an average (harmonic mean) of 60 girls per school and we will include all girls in due to start S2 in 2021 (menstruating and non-menstruating girls) to avoid stigmatising non-menstruators. Based on our pilot, >95% of the girls will have started menstruating at baseline and this is allowed for in sample size calculations where relevant. We will randomly select 15 boys per school to complete a brief assessment on menstrual knowledge and attitudes.

### Baseline assessment of educational performance

The Uganda National Examination Board (UNEB) will conduct an independent examination in Q3 2021 to assess the common core curricula taught in S1 in 2020-2021, among all girls. The examinations will be independently developed, pre-tested, administered and marked by UNEB.

### Endline assessment of self-reported outcomes

All baseline participants will be asked to complete the endline survey in Q1-2, 2023. For boys this only includes the outcomes of knowledge and attitudes towards menstruation. We will aim to follow up boys who have left the school they were at for baseline via phone.

### Endline assessment of educational performance

The UNEB will conduct an independent objective endline assessment in Q1-2 2023 to assess the biology, maths and English curricula taught during the intervention year among all female participants. The examinations will be independently developed, pre-tested, administered and marked by UNEB, who will be masked to study arm. We will collect reasons for students leaving school (especially regarding dropout, which may be associated with poor MHH, versus moving to a different school) and compare this between arms as differential dropout by arm which may bias the intervention effect on educational performance.

## EDUCATIONAL OUTCOMES SUB-STUDY

**Rationale:** There is an evidence gap on the validity of methods to assess school attendance and performance, as well as on the impact of school-based health interventions on educational outcomes.

**Objectives:**

i) To evaluate whether the MENISCUS intervention affects a) school and class absence during menses; b) school and class absence overall and c) school dropout

ii) To validate self-completed daily diaries against other measures of assessing school absence

iii) To collect data on reasons for school dropout and baseline factors associated with school dropout

iv) To assess associations of menstruation and menstrual pain with school and class absence, confidence in maths and science abilities, educational performance, and school dropout respectively.

**Study procedures:** A random sample of ~1500 female participants with parental consent and student assent will be assessed for daily school and class attendance through self-completion of a daily diary (Annex 24) to collect data on school attendance, menstruation and pain. This will be administered for a minimum of 12 weeks from April-June 2023. MENISCUS research assistants will make unannounced visits to the schools approximately every month to check the daily diaries (more frequently for the first 2 months as girls learn how to use them) and will collect the booklet 2 weeks before the end of each term (to avoid loss or damage). Data from the paper daily diaries will be entered electronically at the end of each term when diaries are collected. Semi-structured interviews on perceptions of use of the diaries will be conducted after diary collection with ~24 diary participants (Annex 26), purposively sampled to ensure overall variation across age, urban/rural area and socio-economic status.

The daily diary was used successfully in MENISCUS-1 and MENISCUS-2, and found to be complete (data were entered for >99.5% of days in both studies), and highly correlated with unannounced observation checks (in MENISCUS-2, girls were seen on 328/330 (99.4%) of the girl-days when the diary stated they were present and were not seen on 100% of the 37 girl-days when the diary stated they were absent). Analyses of menstrual patterns indicate that menses were appropriately recorded in the diaries. Further, the qualitative data indicated that the girls’ primary focus in completing the diaries was to track their periods, not the association of menstruation and school attendance suggesting minimal bias with this method. However, successful use of the diaries is dependent on the confidence that girls have in completing them, and we will conduct additional validation studies as part of the main trial (e.g. comparing with observational checks, looking at exercise books to check dates when students were in class, and asking teachers to complete a register for this specific purpose).

## Urinary Tract Infection SUB-STUDY

**Rationale:** A 2013 systematic review found that of 11 studies (only one RCT) investigating the association between MH and urogenital infections, 7 found an increased risk associated with “worse” MH (defined differently for each study but generally meaning not using disposable sanitary pads), one found the reverse to be true (an increased risk from using disposable sanitary pads), and three found no association(9). The menstrual cup may be particularly beneficial as it does not disrupt the vaginal flora (48), but there is relatively little evidence on this to date. (48)(49)

In this study, all female participants with parental consent and student assent will be assessed for a UTI (among those with symptoms of a UTI) at endline.

**Objectives:**

i) To evaluate whether the MENISCUS intervention affects UTIs

ii) To assess associations of menstrual health practices with urinary tract infections

**Study procedures:** Female participants will be asked a set of additional questions at the end of the endline questionnaire to obtain information on current urinary tract symptoms (Annex 12). Participants with at least one symptom of an UTI (e.g. urinary burning and frequency) will be asked to provide a urine sample to be tested by Multistix 10 dipstick for nitrite and leucocyte esterase. Those with positive test results will be referred to the local clinic for management.

## 9.4 SOCIAL NORMS SUB-STUDY

**Rationale:** Social norms are key mechanisms through which long-lasting change in behaviour can occur. Hence, it is important to unpack whether the intervention has changed existing social norms on menstrual health and if so, how. Information on the network structure and network relationships are important to understand the role of central peers and their influence on social expectations of menstrual health behaviours.

**Objectives:**

i) To understand whether the intervention has changed existing social norms on menstrual health and how

ii) To unpack the role of central peers and their influence on social expectations of menstrual health behaviours

iii) To inform potential intervention adaptations in the control groups based on the results from the social norms and social network surveys

**Study procedures:**

1. Social norms questionnaire: We will add questions to the endline survey for all trial participants. The norms questions will be piloted in February-April 2022 with cognitive interviews. The questions cover beliefs and expectations about menstrual health behaviours.

2. Addition of the social network tool. We propose to add a tool to understand network relationships amongst trial participants in schools with fewer than 60 students. The network will enquire about social ties between students and with the MH Action Group. Students will have available in ODK a list of names of students in their year and will be able to select their friends and comment on how frequently they interact together. No identifiable information will be used for the purpose of the analysis.

## 9.5 Process Evaluation

**Rationale:** A detailed mixed-methods PE informed by the MRC framework (50) will have three key areas of investigation related to three core process evaluation functions: implementation, mechanisms of impact and context.

**Aim:** To examine the implementation and potential mechanisms of impact of the MENISCUS intervention, and how these might be affected by context.

**Objectives:**

1. To examine intervention fidelity, reach and acceptability.
2. To describe usual treatment in control schools.
3. To explore potential mechanisms of impact including potential unintended consequences and potential harms.
4. To describe how implementation and mechanisms of impact are affected by context.

**Study procedures:** The key areas of investigation, along with the related data collection methods and sources are outlined in detail in Table 3. Data for the process evaluation will be collected at multiple time points using a range of methods and sources. The process evaluation will remain flexible and dynamic to be responsive and adapt to emerging trial and process findings and so additional data collection may be conducted to investigate emerging findings. Data will be triangulated from multiple sources, and quantitative and qualitative analyses will build upon and reinforce each other Process evaluation findings will not be shared with the implementing partner nor with those conducting the outcome analyses until after the statistical analysis plan has been finalised. This is to maintain a separation between the evaluators and implementers, and to maintain the integrity of the blinded outcome analysis

The methods for each of the 4 objectives are as follows.

*i) Implementation: fidelity, reach, acceptability*

Fidelity of implementation of all intervention components by schools and by WoMena Uganda will be assessed quantitatively. To understand the fidelity and reach of the various intervention components, we will collect and analyse the following data in all intervention schools: log-books completed by WoMena Uganda staff delivering training to teachers and students, and providing MH Kits to students; teachers’ puberty lesson work-plans and records of delivery of puberty lessons; log-books completed by WoMena Uganda staff facilitating drama skit activities; log-books/meeting minutes completed by the MH Leadership Groups; structured researcher observations of drama skit performance content and attendance (Annex 23); structured researcher observations of WASH facilities (Annex 22); structured observations of menstrual health and puberty trainings (Annex 23a); and records of analgesic voucher redemption (Annex 24). We will collect further data on intervention reach and acceptability using student endline surveys (assessing use of re-usable pads, menstrual cups and analgesics, perceptions of WASH facilities) (Annexes 12-13).

*ii) Usual treatment in control schools*

We will examine activities relating to puberty lessons and MH activities, and WASH facilities, in all control schools. Data will be collected via: baseline/endline student surveys (Annexes 10-14); structured phone interview with one senior staff member per control school at baseline and endline (Annex 14); and structured observations of WASH facilities (Annex 22).

*iii) Potential mechanisms of impact*

Informed by May’s Theory of Implementation (51), we will explore implementation processes and mechanisms of impact, and how these vary across the schools, using qualitative data. Data will be collected from all intervention schools via: phone interviews with one senior staff member at baseline and endline (Annex 14); semi-structured interviews with one member of the school’s MH Leadership Group member (Annex 15), purposively sampled to ensure overall variation across schools in gender and type of member (e.g. staff, student, other stakeholder) at mid-line and endline.

In addition, we will conduct semi-structured interviews with the intervention providers (WoMena; n=8) just after initial implementation and then later on during follow-up (Annex 18). Further, we will identify four case-study schools. In each case-study school, we will conduct the following immediately after intervention delivery and at endline: focus group with 6-8 staff purposively sampled by gender; focus group with 6-8 female students; focus group with 6-8 male students; three in-depth interviews per school, spread between staff and female students, purposively sampled to ensure variation in degree of involvement with the intervention and (for female students) whether they opted in to receive a cup. We will also interview 2 female caregivers per case-study school.

Table 4: Overview of qualitative data collection activities

| **Tool** | **Participants** | **When** |
| --- | --- | --- |
| Semi-structured phone interviews with senior school staff (Annex 14) | 60 senior staff members (one per school, N=60) | Baseline and endline |
| Semi-structured interviews with MH Leadership group members (Annex 15) | 30 members of the school’s MHM leadership group member (one per intervention school N=30) (e.g. staff, student, other stakeholder) | After initial intervention delivery and endline |
| Semi-structured interviews with intervention providers (Annex 18) | 8 WoMena Uganda staff | After initial intervention delivery and endline |
| **In case-study schools (N=4)** | | |
| Focus groups (Annex 19-21) | 4 FGs with 6-8 female students (N = 16)  4 FGs with 6-8 school staff (N=16)  4 FGs with 6-8 male students (N=16) | After initial intervention delivery and endline |
| Semi-structured interviews with school staff and female students (Annex 16 and 17) | 24 staff members and female students (menstrual cup recipients and non-recipients) | After initial intervention delivery and endline |
| Semi-structured interviews with female caregivers (Annex 18a) | 8 female caregiver MH Kit recipients | Endline |

1. *Effect of context on implementation and mechanisms of impact*

Detailed data on context and its impact will come from the rapid assessment study and the qualitative research described above. Additionally, we will draw on observations and log-books to understand what social, structural, and logistical factors impede or facilitate how the intervention was implemented and sustained, and how staff and students were able to engage with it.

Table 5: Process Evaluation research questions and data collection methods and sources

| **Research Domain** | | **Research Questions / Key Areas of Investigation** | **Data Collection Methods**  **and Sources** |
| --- | --- | --- | --- |
| **Implementation**  *What is implemented and how?* | **Fidelity** | Were training, and school components implemented as planned?  What adaptations were made?  How did fidelity vary between schools?  What were the barriers and facilitators to implementation fidelity? | Log books  Structured observations  Endline surveys with male and female students  IDIs and / or FGDs with students, staff, MHM leadership group members, intervention deliverers  WASH checklist |
|  | **Reach & acceptability** | What proportion of students received and accessed various components of the intervention?  How acceptable was the intervention to students?  How did reach and acceptability vary by student characteristics?  What contextual factors affected reach and acceptability? |  |
| **Usual treatment** | **Comparator** | What is usual provision of puberty lessons and MHM activities in control schools?  What puberty and MHM activities are happening within schools and in the wider community?  What WASH provision exists in control schools? | Baseline/endline surveys with male and female students  Rapid assessment data  Structured interview with senior staff member at endline  WASH checklist |
| **Mechanism of Impact**  *How does intervention lead to change?* | **Interactions and Consequences** | How did intervention providers and school staff and students describe how they used the intervention resources to enact the intervention, including any adaptations?  What mechanisms did participants say were triggered by enactment of intervention activities and what impacts and other consequences did these have on students and the school? | IDIs and / or FGDs with students, staff, MHM leadership group members, intervention deliverers  Structured interview with senior staff member at endline |
| **Context**  *How context affects implementation and shapes outcomes?”* | **Proximal and Distal** | How did participants describe enactment and mechanisms as affected by school context?  What were contextual reasons for adaptations to the intervention and its delivery? | IDIs and / or FGDs with students, staff, MHM leadership group members, intervention deliverers  Rapid assessment data  Structured interview with senior staff member at endline  Log books |

## 9.6 Economic Evaluation

**Rationale:** The economic evaluation will evaluate the costs of setting up and running the intervention package, the unit cost per female student reached, and the incremental cost-effectiveness of the intervention per unit increase in selected policy-relevant outcomes, relative to optimised usual care. The cost-effectiveness measures will be compared to similar school-based interventions in the region to inform scalability and financial sustainability.

**Aim:** The aim of this economic evaluation is to determine the cost-effectiveness of the intervention compared with enhanced standard practice.

**Objectives:**  To conduct:

1. Cost–utility analysis comparing costs and utilities of the two groups with costs considered from a provider perspective and health outcomes measured by QALYs based on the Child Health Utility–9D (CHU9D).
2. Cost-effectiveness analyses of the intervention effect on SDQ score
3. Cost-wellbeing analysis, with costs considered from a provider perspective and outcomes measured by improvement in life satisfaction.

**Study procedures:** Resource use will be identified through early discussions with the trial team, the implementing partner, and the schools. Resource use will be measured over the duration of the trial and will involve the following data collection: (1) resource use due to the setting up and delivery of the intervention including staff time, training, materials, drugs and supplies; (2) school resource use including staff time and materials. Outcome data will be collected at baseline and endline as part of the quantitative surveys.

## 9.7. QUALITY OF LIFE MEASUREMENT AND VALUATION SUB-STUDY

**Rationale:** The CHU9D tool can be used to measure adolescents’ quality of life for economic evaluation. However, there is currently, no published evidence of its validity and use in Uganda and in the context of menstrual health. There are also no tariffs for any of the countries in Africa, so studies using the CHU9D in Africa, such as MENISCUS, rely on tariffs from other regions (United Kingdom, China, Netherlands, Australia) to generate health utilities for economic evaluation. This could potentially bias the quality adjusted life years (QALY) estimate used in economic evaluation. We therefore need to estimate country/population-specific tariffs for the tool for use in Africa. Existing conventional methods for generating tariffs for tools like the CHU9D are the standard gamble (SG) and time trade-off (TTO). These pose a challenge for children and adolescents because they involve consideration of death or trading time and risks, concepts young people may not fully comprehend. Other methods such as the best-worst scaling (BWS) method and discrete choice experiment (DCE) have been proposed for use among young people.

**Aim:** To validate and generate tariffs for the child health utility 9-dimension tool among adolescents in secondary schools in Uganda and also assess the impact of the MENISCUS interventions on quality of life.

**Objectives:**

1. Assess the feasibility, reliability, and validity of the CHU9D tool in the Ugandan context.
2. Estimate tariffs for the CHU9D tool for Uganda and explore the factors associated with the tariffs.
3. Assess the impact of the MENISCUS intervention on adolescent girls’ QoL using the CHU9D and how this impact is distributed across SES and location.

**Study procedures:** This study will use selected baseline and endline data from the MENISCUS trial and interviews conducted after the endline of the MENISCUS trial (Table 6). The CHU9D tariffs will be generated with MENISCUS trial participants between the ages of 11-17 years from 12 schools in the MENISCUS trial. In each of the 12 schools, all boys and a random sample of 25 girls between the ages of 11-17 years will be selected to participate in the valuation exercise. Prior to the valuation exercise, the BWS method and DCE will be pre-tested to select the preference elicitation methods will be piloted to select the most suitable method for adolescents in Uganda. The CHU9D tool will also be validated for cultural and linguistic appropriateness for Uganda. Because the BWS and DCE do not generate values on the 1-0 full health–dead QALY scale and require re-anchoring with adult values generated through SG or TTO method, ~120 parents (males and females) of trial participants to be part of the valuation exercise will be randomly selected to generate values for the CHU9D using the TTO method for the purpose of re-anchoring.

**Study participants**

Pre-test: Two schools (one per district) will be selected to participate in the pretesting of the CHU9D valuation as well as the linguistic and cultural validation exercise. In each of these schools, 10 adolescent boys (n=5) and girls (n=5) will be randomly selected to participate in the pre-test of the adolescent CHU9D valuation and linguistic and cultural validation exercises. Parents (males and females) of 10 of the adolescents (boys and girls) will participate in the pre-test of the adult CHU9D valuation exercise using TTO method. The 2 schools which will participate in the pre-test will be excluded from the CHU9D valuation exercise below.

CHU9D valuation exercises: 12 MENISCUS schools – 4 in Kalungu and 8 in Wakiso – will be conveniently selected to participate in the CHU9D valuation exercises. The breakdown of the 12 schools will be as follows:

Kalungu= 2 control schools (1 private and 1 government owned) and 2 intervention schools (1 private and 1 government owned); Wakiso=4 control schools (2 private and 2 government owned) and 4 intervention schools (2 private and 2 government owned). In each of the 12 schools, all male participants (n=15 per school), and a random sample of 25 females between the ages of 11 to 17 years in the MENISCUS trial will be selected to participate in the adolescents’ CHU9D valuation exercise. We expect a sample size of about 450 participants. This is because some schools are likely to have fewer than 15 boys within the 11-17 years age bracket. A review of studies which used the BWS method for instance has reported that sample size used in BWS studies range between 16 and 5026 with a mean of 442 respondents (50).

The parents of ~120 trial participants involved in the CHU9D valuation exercise will be purposively selected and invited to participate in the adult valuation exercise using the TTO method. These parents will be selected using a simple random sampling method.

Quality of life assessment: data for this analysis will be for all adolescent girls in the MENISCUS trial seen at endline.

The breakdown of the sample for the different activities of the study is presented in table 6.

Table 6: Study activities, sample, data source and period of data collection

| **Study components** | **Activities** | **Sample** | **Data source** | **Data time point** |
| --- | --- | --- | --- | --- |
| CHU9D Pre-test and cultural and linguistic validation | CHU9D valuation pre-test and cognitive debriefing with adolescents | Total of 20 adolescent boys and girls in 2 schools – 1 in Kalungu and 1 in Wakiso | Field interviews | Before CHU9D valuation exercise and after MENISCUS endline data collection) – July and August 2023l |
|  | Adults’ TTO valuation exercise | 10 parents of adolescents engaged in the in the pre-test (same schools as above) |  |  |
| Objective 1 | CHU9D internal consistency | 3844 adolescent girls | MENISCUS baseline CHU9D data | MENISCUS baseline (June/July 2022) |
|  | CHU9D construct validity (groups of known differences) | 3844 adolescent girls | MENISCUS baseline data:SDQ-25, life satisfaction and happiness scores, Socio-economic status, CHU9D utility scores |  |
|  |  | ~450 adolescent boys and girls in MENISCUS | Field interviews: self-reported general health status and quality of life rating | CHU9D valuation exercise (after MENISCUS endline data collection) – August 2023 (Kalungu) and October 2023 (Wakiso) |
|  | Feasibility | ~450 adolescent boys and girls | Field interviews |  |
| Objective 2 | Adolescents CHU9D valuation exercise | ~450 adolescent boys and girls |  |  |
|  | Adults CHU9D valuation exercise | Parents (males and females) of ~120 adolescent boys and girls |  |  |
| Objective 3 | Quality of life assessment | 3844 adolescent girls | MENISCUS baseline and endline CHU9D data | MENISCUS baseline and endline |

**Study activities**

1. Pre-test:

Prior to the CHU9D valuation exercise, the BWS method and DCE will be pre-tested among 20 adolescent boys and girls selected from 2 MENISCUS schools – 1 in Kalungu and 1 in Wakiso – to select the preference elicitation method most suitable for adolescents in Uganda. With the BWS method, the adolescents will be required to consider one health state at a time and select their best and worst attribute as shown in figure 7 below. In the DCE method, participants are required to compare two health states at a time and select their best health state as shown in table 8 below.

The time-trade-off method will also be tested among parents of 10 adolescents selected from the same schools.

Table 7: Sample BWS task


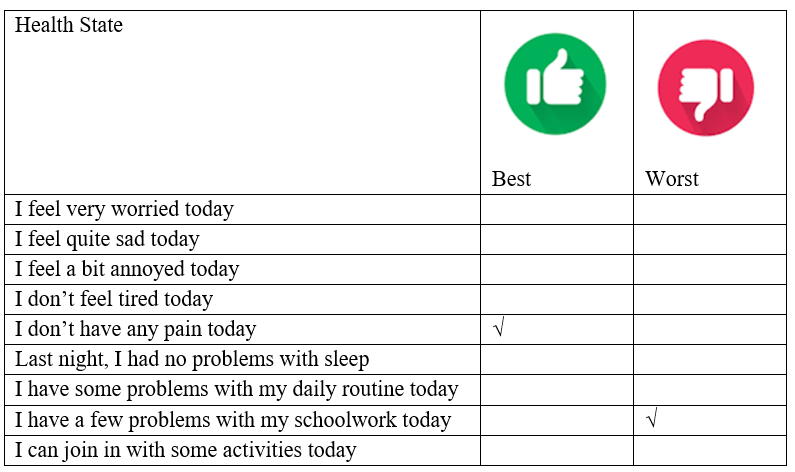


Table 8: Sample DCE task

| Health State A | Health State B |
| --- | --- |
| You feel a little bit worried  You feel a bit sad  You have a bit of pain  You feel quite tired  You feel quite annoyed  You can’t do work/housework  You have a few problems sleeping  You can’t do your daily routine  You can join in with any activities | You feel a little bit worried  You feel very sad  You don’t have any pain  You feel quite tired  You don’t feel annoyed  You have many problems with your work/housework  You can’t sleep at all  You have a few problems with your daily routine  You can join in with any activities |
| Which do you prefer | |

2. Linguistic and cultural adaptation of the CHU9D tool for Uganda:

Since the CHU9D has not yet been validated in Uganda, validation process for cultural and linguistic suitability of the tool for use among adolescents in Uganda will be conducted. This will involve a cognitive debriefing with selected adolescents to assess their understanding of the tool. The 20 adolescent boys and girls who will be part of the pre-test explained above will be engaged in the cognitive debriefing exercise. They will be asked to complete the CHU9D tool whilst thinking aloud. This way they will voice out their understanding of the different dimensions and levels. An interviewer will observe, note down and audio-record respondents’ responses as they complete the form. Recordings will be transcribed and analyzed.

3. Objective 1:

Objective 1 will provide evidence of psychometric performance of the CHU9D. The following psychometric tests will be conducted: internal consistency reliability, construct validity and feasibility.

*Internal consistency* will assess the inter-item correlation between the different dimensions of the CHU9D. This test will be conducted using baseline data from MENISCUS.

*Construct validity* will assess the CHU9D’s ability to discriminate between groups of known differences. First, it will assess the extent to which adolescent girls’ CHU9D utility scores compare with their SDQ scores, life satisfaction and happiness scores. Second, it will assess the extent to which adolescent boys and girls’ CHU9D utility scores compare with their socio-demographic factors (age and SES), self-reported general health status and quality of life rating. Age will be grouped into two, younger adolescents (≤14 years) and older adolescents (15–19 years).

*Feasibility:* the time (start and end time) the adolescents use to complete the CHU9D before the valuation exercise will be recorded. The total number of unanswered questions will also be recorded. These two pieces of information will be used to assess feasibility.

4. Objective 2:

In this objective, tariffs for the CHU9D will be estimated. All 1,953,125 unique health states of the CHU9D cannot be included in a valuation exercise to generate the tariffs. A sample of health states will be selected for valuation. There will be two valuation exercises, one with adolescents and another with parents as explained below:

*Adolescents CHU9D valuation exercise*

About 450 adolescent boys and girls selected (from 12 MENISCUS schools) to participate in the adolescents’ CHU9D valuation exercise will be asked to complete the CHU9D before the valuation exercise. Before the valuation exercise, the concept of health states and the valuation exercise will be explained using a vignette developed in English and Luganda. An example of the task to be undertaken will also be explained in the vignette. The adolescents will then be presented with the pre-selected health states (maximum of 10) and asked to value them based on the elicitation method selected after the pre-testing (as explained above). The CHU9D valuation exercise will be self-completed on tablets by the adolescents with the assistance of a study data collection team member. During the valuation, the adolescent boys and girls will be told to assume themselves in the health states presented. At the end of the exercise, they will be asked questions about their general health status, whether they are living with a disability and or a long-term chronic disease. They will also be asked to rate their quality of life on a scale of 1-5 where 1= poor and 5=excellent; and what factors they consider as important as they rate their quality of life. This will help to know what quality of life means to adolescents in the study setting and whether these were captured by the dimensions of the CHU9D.

*Adults CHU9D valuation process*

Parents/guardians of ~120 adolescent boys and girls who will be part of the adolescents’ valuation exercise explained above will be randomly selected to participate in the adults’ valuation exercise. They will be asked to choose their preferred health state from pre-selected health states using the time-trade-off (TTO) method. The TTO method will require them to choose between living in a perfect health state for a period (example 8 years) and die or living in a sub-perfect health state for a longer period (10 years) and die. The number of years in the perfect health state will be varied until the respondent is indifferent between the two health states. The adult CHU9D valuation exercise will be administered by an interviewer. The valuation exercise will be conducted in Luganda for the adult CHU9D valuation exercise where the parent participant does not understand the English language. After the CHU9D valuation exercise, they will be asked open questions on their perception about the CHU9D tool and the difficulty/ ease of the valuation exercise. Their responses will help to explain any differences that will be identified between the adult and adolescents’ CHU9D values.

## 9.8 SLEEP sub-study

**Rationale:** Poor sleep is common during adolescence, and is associated with poor menstrual health, and poor mental health. Uganda is an ideal setting to develop a school-based sleep intervention, with an increasingly-recognised problem of poor sleep quality^7^ and a high proportion of students of all socio-economic backgrounds who board, which increases the potential feasibility and impact of a sleep intervention. There is a strong link between menstrual health and sleep health, as for example, symptoms that vary across the menstrual cycle (dysmenorrhoea, heavy bleeding, breast tenderness, temperature fluctuations, anxiety) are associated with poor sleep. In turn, poor sleep can cause menstrual irregularities and increased sensitivity to menstrual pain through disruption of circadian rhythms and/or effects on pain regulatory systems in the central nervous system. (Figure). We will conduct a sub-study on sleep health which builds on our experience in developing, delivering and evaluating school health interventions in Uganda, and our strong stakeholder relationships.

Most schools in Uganda have day and boarding students (44.6% were boarding students in MENISCUS), providing the opportunity to assess the impact of structural school-level changes on reduced noise and light in the evenings and morning light exposure. The study is low-risk due to our research experience, infrastructure and stakeholder engagement in school health in this setting. Discussions with 41 teachers in the MENISCUS trial, and the District Education Officer indicate that improving school structures around sleep time is feasible and valuable.


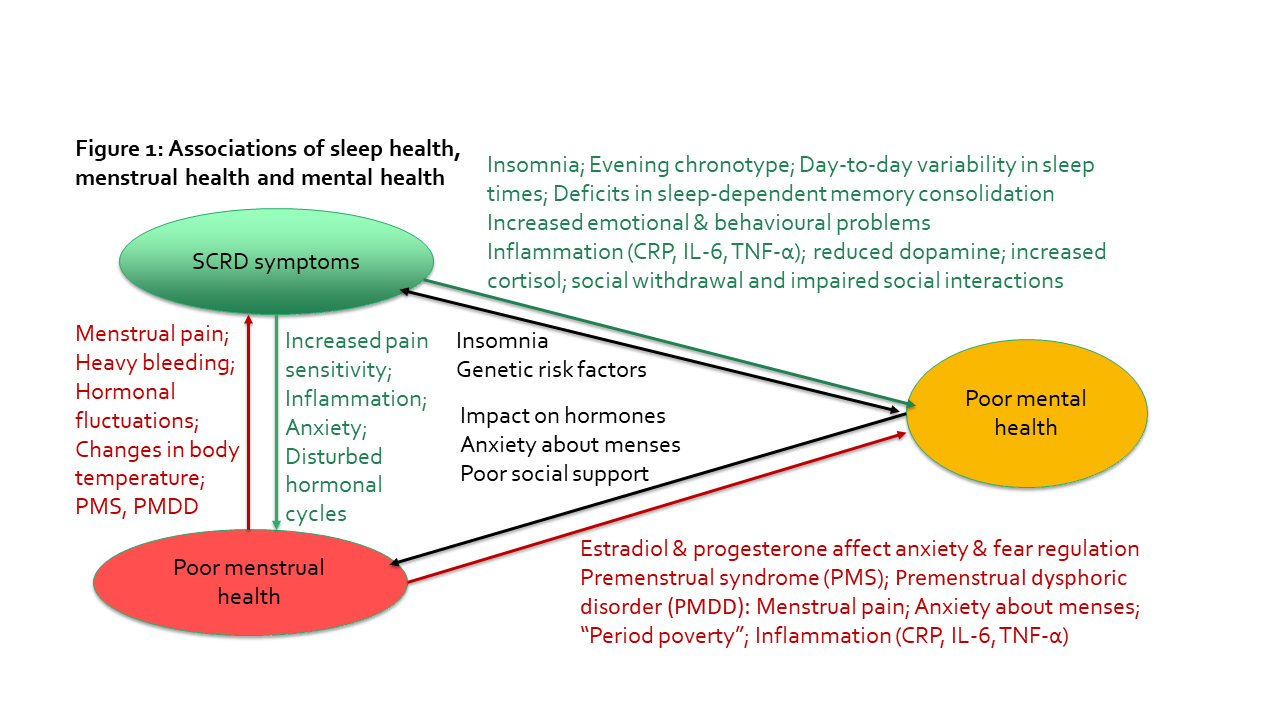


**Aims of the sub-study:**

Phase 1: Inform early-stage intervention development by understanding the social, cultural and physical contexts of sleep in Ugandan secondary schools, and the prevalence of different forms of sleep problems among students;

Phase 2: Co-develop a theory-based sleep health intervention to improve sleep health and subsequently mental health in schools;

Phase 3: Disseminate findings and prepare a detailed plan to evaluate the intervention in a future trial.

**Study procedures**: Fieldwork will take place in 2 mixed day/boarding schools in Wakiso District. These will be selected from MENISCUS trial schools with whom we have a good working relationship, and fulfil the following criteria:

School-level eligibility criteria are:

- Mixed sex schools with approximately 40 S3 female students in our endline survey
- Schools with both day and boarding students
- Willing to participate in an intervention to modify school structures to improve sleep health
- Within easy reach of Entebbe town

Individual-level eligibility criteria are:

Students in Secondary 2 or Secondary 3

**Phase 1: Informing intervention design through a contextual understanding of sleep health (June-August 2024)**

**i) Qualitative assessment of the sleep environment:**

At each school, approximately 17 participants will be involved in a rapid assessment of the school physical and social sleep environment (table 9). Participants will be day and boarding students and staff. The aim is to collect enough in-depth data from individuals or groups to be able to capture variations in informants’ perspectives and experiences related to the research questions. Generalisability is not the main goal of this qualitative research. Instead, our intention is to understand the *meaning* of what we hear or see regarding the research question in a broader *context* (which might be social, cultural, economic, environmental etc.). Prior to the quantitative survey, a random selection of participants in each school will be asked to participant in cognitive testing of the questions that will appear in the quantitative survey. The purpose of the cognitive testing is to understand whether students understand the question properly.

The data collection tools are provided in Annexes 57-66. The methods are directly drawn from those we have used in the main MENISCUS study^[[6]](#footnote-7)^. We will use semi-structured interviews, participatory group discussions and structured observations. Central to the methodological approach is observation; spending time in the school communities chatting to teachers, students and parents and learning about school environment. Whilst being open about the purpose of the fieldwork, researchers will aim not to be intrusive.

**ii) Quantitative assessment of sleep health:** To assess prevalence of the multiple dimensions of sleep health and behaviours and to evaluate the performance and feasibility of administering self-reported sleep health tools, we will conduct a quantitative survey in all male and female students in S2 or S3 (around 300 students in total). Mean age is 15.7 years (SD 1.02) in S2.

Following parental consent/student assent, we will select around 5 participants per school to participate in 1-1 cognitive interviews for the following Sleep tools which have not previously been used in Uganda:

- **Insomnia Severity Index**: Clinically-validated 7-item screening tool for insomnia^[[7]](#footnote-8)^.
- **Munich Chronotype Questionnaire**: 8-item tool for adolescents assessing circadian rhythm disturbances (sleep timing and duration, sleep onset latency for school and non-school days^[[8]](#footnote-9)^)
- **Dysfunctional Beliefs and Attitudes about Sleep (Child) Scale**: 10-item tool to assesses sleep-related cognitions including dysfunctional expectations ^[[9]](#footnote-10)^

**Cognitive interviews:** Participants will be purposefully selected for different levels of fluency in English. The cognitive interviews follow a semi-structured format discussing each item’s face and construct validity, assessing local understanding and appropriateness of items, explore how participants interpret items/questions, why they provide a specific response, and how items of interest fit in their life^[[10]](#footnote-11)^. This process will be conducted using techniques such as “thinking aloud” and verbal probing to assess suitability and interpretation of the response options and understand the process whereby the response was chosen. To reduce participant burden, participants will be asked to focus on items they find difficult to understand or feel could be rephrased to improve clarity. Potential changes will be discussed during debriefing by the team prior to any changes being made.

**Quantitative survey**

The survey will include the following sections:

- **Socio-demographic questions** (age, gender, SES, boarding/day status, maternal/paternal education)
- **Pubertal status** (growth, skin changes, hair changes, voice changes, breast development, menarche)
- **Experience at last menstrual period** (duration, flow, pain, pain management, impact on school and social participation)
- **Patient Health Questionnaire-9** for depressive symptoms
- **Generalised Anxiety Disorder Assessment-7** for anxiety
- **Global psychopathology (SDQ**) for mental health symptoms

Access to a counsellor will be arranged if participants experience distress or need support during or after the interview. Participants reporting current suicidal ideation will be offered the opportunity to be referred to a local support services.

**Phase 2a: Stakeholder workshops to review theory and co-develop a programme theory and intervention (September 2024)**

The workshops will include discussions with i) students (led by RAHU peer educators); and ii) school staff, parents and policymakers in the Ministries of Education and Health. Building on Phase 1 and existing theory, participants will initiate group ideation around preferred intervention components. We will adapt cognitive behavioural therapy for insomnia (CBT-I) components that are deemed impractical in this setting, such as recommendations to only use the bedroom for activities related to sleep. Such advice is unlikely to be possible for students living in families with little living space or at boarding school^[[11]](#footnote-12)^. The outcome will be a programme theory and intervention with content selected and adapted from relevant protocols. The draft intervention components are shown in Table 10. The logic model (Figure 7) shows the likely Step 1 (whole-school) and Step 2 (additional targeted) components and mechanisms of action.

Findings will be used to complete the Template for Intervention Description and Replication (TIDieR) framework, and a draft intervention manual, materials and training package. Educational Consultant Alezuyo will work with the peer educators and psychologists to design the Step 1 school level education sessions, drawing on open-access online resources (e.g. [Sleep School Pack - Mental Health Foundation](https://www.mentalhealth.org.uk/our-work/programmes/families-children-and-young-people/resources/sleep-school-pack); [Sleep Resources - School Wellbeing](https://www.schoolwellbeing.co.uk/articles/new-rise-above-sleep-resources)). Psychologist Co-Leads Bannink-Mbazzi and Orchard will guide drafting of Step 2 for students with sleep problems.

**Phase 2b: Feasibility studies to refine the programme theory and intervention**

Step 1: In June-July 2024, Alezuyo and the peer educators will train teachers to deliver Step 1 sleep education material to S2 and S3 students (~300 students). In September-October 2024, RAHU will support schools in implementing structural changes to noise/light and school start times. Step 2 (for ~40 students identified with sleep problems on the Insomnia Severity Index; 2 groups per school) will be delivered by 2 counsellors with a psychology degree, trained and supervised by Bannink-Mbazzi, and assessed for core CBT-I competencies. We anticipate 5x1 hour weekly sessions including TranS-C/CBT-I components as described in Table 10 (e.g. i) introduction and goal setting; ii) understanding the importance of sleep regularity and schedules; using a sleep diary to regulate these; iii) cognitive restructuring to reduce worrying by challenging and replacing unhelpful beliefs with constructive thoughts, and developing skills to cope the day after a night of poor sleep; relaxation exercises; iv) behavioural elements (addressing unhelpful social and physical environments) and v) maintenance of behaviour change and relapse prevention). As part of the intervention, students will be asked to complete the Consensus Sleep Diary, a prospective 7-day diary to monitor sleep duration, timing and quality^[[12]](#footnote-13)^. The diaries will be collected for data entry.

**Assessment of feasibility and acceptability**: For Step 1, RAHU will complete a checklist on the number and content of delivered sleep education sessions and will assess structural changes through observation and 8 endline qualitative interviews with selected staff and students. For Step 2, Bannink-Mbazzi will complete a fidelity checklist (Annex 66), and participants will complete an endline self-report of enactment of the strategies (i.e. whether intervention participants use the intervention skills in practice. We will also conduct an endline quantitative survey repeating the Phase 1 survey in these 40 participants to assess improvements in sleep health and mental health (Annex 57). Together these will capture the extent to which schools are engaged with and delivering the intervention, and stakeholder viewpoints towards the intervention, how the intervention is disseminated within the school community, barriers to successful delivery, and acceptability of the data collection tools.

**Phase 3: Dissemination of findings (February-March 2025**) including i) feedback to local and national stakeholders; ii) papers on sleep health in this population, and the feasibility and acceptability of intervention delivery; iii) a revised programme theory and plan for the intervention to be evaluated in a future trial.

| **Table 9: Rapid qualitative assessment: Research activities, objectives questions, methods & participants** | | | | |
| --- | --- | --- | --- | --- |
| **Research activity** | **Objective** | **Key research questions** | **Methods** | **Participants** |
| **Interview with head teacher** | To explain the purpose of the exercise, and understand the scope for improving sleep health in the school | 1. What are the key factors contributing to poor sleep and tiredness among students? 2. Which aspects of the current structures around student sleep time can be adjusted or changed and which classes would it be possible with? 3. What would the challenges be in developing an intervention to improve sleep for day and/or boarding students? | Semi-structured interviews (Annex 1) | School head teacher (N=2) (or delegated senior teacher) at each school |
| **Interview with matron, students and parents** | To understand the physical and social sleep environment |  |  | 1) Matron (person in charge of dormitory) (N=2)  2) Female students (N=10)  3) Male students (N=10)  4) Parents (N=8) |
| **Interview with Wakiso District officials** | To understand the scope for implementing a sleep health intervention in District |  |  | Two district-level school officials |
| **Participatory group interviews** | To explore sleep patterns, knowledge and perceptions of links between sleep, menstrual health, academic performance and mental health | Description of the school environment (including WASH and illness management)  Perceptions of the interventions, support structures and facilities related to WASH and reproductive or menstrual health in the school  What COVID-related interventions are taking place in schools?  What activities and groups or committees do students take part in? | Adolescent-centred participatory methods (e.g. role- play and listing/ranking exercises). (Annex 4) | One group discussions (GD) in each school. 10 participants in each group |
| **Participatory transect walk** | To observe the social and physical environment of the school, relevant to sleep health | 1. What is the school setting? 2. What is the physical sleep environment for boarding students? 3. How is the managed and supervised? 4. What is the social environment for sleep? | Transect walk starting from central point, moving in concentric circles around the school and taking care to stop, listen, look and chat on the way.  A rough sketch map of the setting, showing particular features associated with the sleep environment (Annex 2)  Structured checklist to describe sleep environment (location of dormitories, management and supervision) (Annex 3) | A wide range of the school community will be engaged in informal conversations during the walk.  Teachers, and/or student representatives will act as guides. |

| **Table 10: Intervention components CBT-I and Trans-C *adapted from Egbegi et al 2021*** | |
| --- | --- |
| **Session 1:**  Introduction and setting rules  Importance of sleep | Facilitator and participants introduced each other  Group rules agreed including confidentiality  Overview of the 5 sessions  The importance of sleep for good physical health, mental health, and optimum daily  function including better concentration and attention and the potential benefits for a student. |
| **Session 2:**  Psychoeducation; Physiology of sleep and sleep schedules | Review Session 1  How normal sleep occurs including sleep drive (role of Adenosine), and Circadin  Rhythm (role of Melatonin)  Components of sleep hygiene and how they contribute to improving sleep  Importance of sleep regularity, schedules and quantity for adolescents; role of sleep diaries  Impact of caffeine on blocking Adenosine and sleep drive, and role of light in blocking  Melatonin |
| **Session 3:**  Cognitive restructuring, and managing bedtime worries; Relaxation techniques | Review previous sessions  Effect of stress on sleep and how to reduce stress  Managing bedtime rumination on worries—by recounting positive experiences in the day and focusing on these to distract from worries  Developing skills to cope the day after a night of poor sleep  Types of relaxation techniques—deep slow breathing, progressive muscle relaxation, and positive imagery How to make relaxation a habit by practising relaxation techniques throughout the |
| **Session 4:**  Behavioural changes and stimulus control | Review previous sessions  Key behavioural changes that can be introduced  Further discussion on roles of light, noise, caffeine and use of gadgets  Stimulus control as part of improving the sleep environment (acknowledging limitations from physical environment) |
| **Session 5:**  Maintenance of behaviour change & relapse prevention | Review of all key messages from sessions 1 to 4  Checking that participants are practising the techniques and troubleshooting any difficulties  Relapse prevention  Formal ending of the intervention |

***
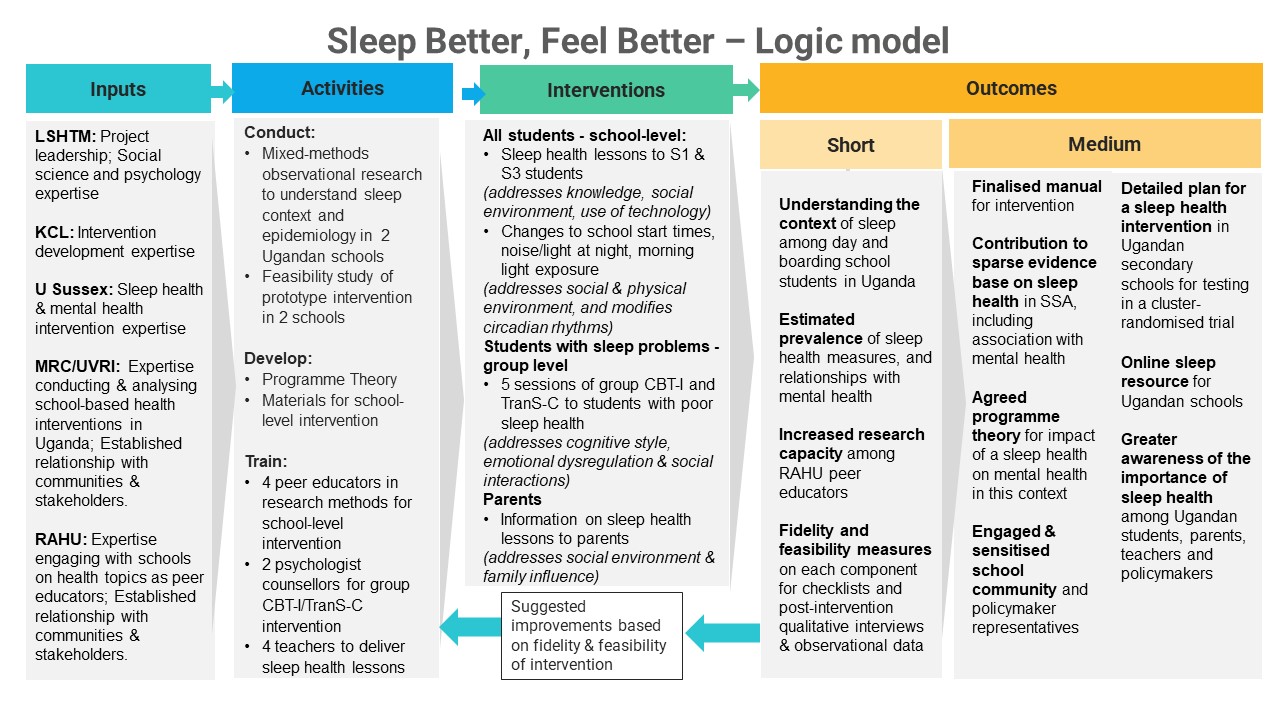
***

Figure 7 Logic Model

## 9.8 Policy Analysis

**Rationale:** To assess the policy environment around MH in Uganda, focusing on how implementing the intervention contributes to, and aligns with, the attainment of the Government policy objectives on menstruation management in schools.

**Aim:** To assess the policy environment and financial sustainability of our intervention in line with Government policies and frameworks for MH and sexual and reproductive health.

**Objectives:**

1. To identify the key objectives of relevant policies (including the Government MH policy, NSEF policy, Adolescent Reproductive Health Policy and the National Girls Education Strategy), and how the MENISCUS intervention contributes to, and aligns with, these objectives;
2. To assess the extent to which the trial findings inform how these policies can be optimally delivered nationally;
3. To assess the economic benefits of the intervention to inform scaling up and better informing the existing relevant policies.

**Study procedures:** As part of the process evaluation, we will assess the implementation of the MHM guidelines and NSEF in both trial arms and identify the supportive and constraining factors to the implementation of these guidelines and how the findings of the MENISCUS study inform their refinement and formulation of a national policy. We will adopt the basic approach to policy analysis of assessing the inclusiveness, magnitude of the problem; feasibility and clarity of objectives and implementation, social and economic environment; monitoring and evaluation frameworks and sustainability.

We will evaluate the impact of Government MH and other relevant policies delivered in the control arm, through baseline observation checks, PE data and endline data.

## 9.9 loss to follow-up

**Open cohort:** We have designed the trial as an open cohort cluster-randomised trial, to allow for a relatively high degree of turnover of individuals due to moving or school dropout. In the MENISCUS-2 study, 82% of participants present at baseline (S2) were present at the endline survey 9 months later (S3). In the current trial, the follow-up period spans ~15 months, so loss to follow-up may be greater than 20%. In addition, we would like to include students who joined during the intervention year and are present at endline. Our power is therefore based on the intervention vs control comparison at endline, adjusting for baseline measures of outcome variables. It is possible, however, that there will be differential loss-to-follow-up by arm, if the intervention reduces school dropout. We will collect data on school dropout in girls and adjust for this in endline analyses.

## 9.10 trial closure

Following primary data analyses and discussion of results by the team, the TSC and the DSMB, we will conduct a Stakeholders’ workshop in each District to disseminate findings and discuss policy implications. Schools in the intervention arm will retain all hardware received as part of the MENISCUS intervention. Schools in the control arm will be offered the opportunity to receive the MENISCUS intervention. Data storage and sharing is as described in the Data Management Plan (Annex 2).

## 9.11 CONSENTING FOR FURTHER SURVEYS

In Q1 and Q2 2024, we will re-contact parents on female trial participants to request consent to re-administer the endline survey to their daughters in the future, to assess longer-term outcomes of the intervention.

# data management and analysis

Data collection and management is described in detail in the Data Management Plan (Annex 2). Briefly, survey data will be self-collected electronically on tablets using ODK software, and uploaded to the LSHTM secure server, and paper-based data will be entered using ODK. IDIs and FGDs will be audio-recorded and transcribed verbatim, including relevant non-verbal communications and translated into English by the study interviewers. Cost data will be inputted into an Excel-based costing tool.

## Sample size justification

The sample size of 60 schools with a harmonic mean of 60 girls per school in the endline survey provides 84% power to detect an effect size (standardised mean difference) of 0.2 for educational attainment and for SDQ Total Difficulties Score (mental health symptoms), assuming an intra-cluster correlation (ρ) of 0.05 and α=0.05. The effect size of 0.2 is that observed for SDQ score for girls in MENISCUS-2 (baseline score=10.3, endline score=9.2, SD at baseline=5.56)(22). It is considered ‘moderate’ for educational outcomes(52) and hence of policy relevance. The analyses for each outcome will use MMRM to increase precision given missing data(37). We have not adjusted the Type 1 error because these two outcomes are independent, and an improvement in one without the other will be informative. For example, it is plausible that the intervention will improve SDQ score but not have an effect on educational attainment (Figure 2) and vice-versa.

Secondary outcomes (Table 2) will be assessed at endline, stratified by gender (for knowledge and attitudes) and adjusted for the relevant baseline measure. We are well-powered for these outcomes, e.g. 90% power to detect 12% vs 20% participants answering all 9 knowledge questions correctly in the control vs intervention arms for girls (assuming ρ=0.05), and 85% power to detect 4% vs 12% for boys (as in the pilot), assuming 10 boys per school seen at endline (22). From our pilot, we expect >95% of girls to be menstruating, and this is accounted for in the power calculation where relevant. Intervention effects will be estimated with logistic or linear regression for binary or continuous outcomes, using random-effects to adjust for clustering by school and fixed effects for restriction variables.

The post-menarche girls in the nested cohort (N=~1500); will provide diary data on school/class attendance and their menstrual cycle over 12 weeks (60 school days) at endline. This sample size provides >98% power to detect a 20% reduction in period-related absence at endline (based on pilot data (22)), and ~89% power to detect this difference by district or other equally-sized sub-groups.

The estimated harmonic mean of 60 girls per school present for the endline survey accounts for expected decreases in school size following COVID-19 school closures (in addition to non-consent and net attrition from baseline to endline). If school registers obtained prior to seeking parental consent indicate that schools are larger than estimated, we may reduce the number of schools included in the trial (to a minimum of 48 schools) in order to avoid a large increase in the number of individual participants.

## Statistical Analyses

We will follow CONSORT guidelines for analysis of CRTs (53). A Statistical Analysis Plan will be drafted prior to data analysis and finalised after approval by the TSC and DSMB.

1. **Main trial analyses:**

**Participant flow:**  A participant flow diagram will be completed, following CONSORT guidelines for CRTs (Figure 1).

**Descriptive statistics:** Descriptive statistics for school-level and individual-level baseline characteristics will be shown by arm and will include mean and SD (or median and range if non-Normal) (for continuous variables) and counts and proportions (for categorical variables). Factors showing substantial baseline imbalance by arm will be noted for inclusion in effectiveness analysis.

**Effectiveness analysis:** The primary and secondary outcome analyses (Table 2) will be carried out for all schools as randomised (intention-to-treat). Intervention effects will be estimated using individual-level mixed-model repeated measures (MMRM) regression analysis, adjusted for clustering by school (random effect) and baseline measures of outcome variables, restriction variables and factors imbalanced by arm at baseline. Continuous outcomes will be analysed using linear MMRM, and binary with logistic MMRM analyses. Results will be presented as standardised mean difference (continuous) or odds/prevalence ratio (binary – depending on prevalence of the outcome) with a 95% confidence interval. For the analyses of daily diary data, there are repeated measures per girl, and analyses will additionally adjust for within-girl clustering using random-effects.

We will conduct a-priori defined sub-group analyses such as by age (<16 vs >16 years), type of school, and by District.

1. **Process Evaluation analysis**:

Descriptive statistics will describe fidelity, reach and acceptability using Chi-square tests to examine differences between schools. These will examine whether fidelity differs by type of school and school resource, and whether student-level reach and acceptability differ by student-level factors including socio-economic status. Quantitative and qualitative data from control schools will be analysed to describe usual treatment in control schools. Qualitative data will be subject to thematic content analysis informed by May’s implementation theory to: examine implementation processes, potential intervention mechanisms and how these vary between schools and students.

Qualitative data will also be used to explore hypotheses about implementation processes/intervention mechanisms and how these might vary between schools or students. Transcribed data will be reviewed for accuracy. Data will be analysed using thematic content analysis first by in-vivo (verbatim) codes and then axial coding to identify relationships between the codes. Transcribed recorded data and field notes will be read and coded independently by 2 social scientists and thematic interpretations will be discussed further at team meetings led by the senior social scientist to discuss and compare the coded work to ensure inter-rater reliability. Thematic analytical memos will be developed and shared with the wider team for discussion as well as forming the basis for outputs.

1. **Economic Evaluation:**

All analyses will be performed on individual participant-level data, taking clustering into account, and collected from the trial. A costing analysis will provide the costs of setting up and running the intervention package, describe the distribution of costs across 5 intervention elements, the unit cost per student reached and the cost of delivering all activities in intervention schools. A combination of top-down and ingredients-based costing approaches will be used to generate cost estimates for the whole intervention and for each component from the provider’s perspective (i.e. the schools, and implementing partner, WoMena Uganda). Costs will be extracted from financial records, project accounts and staff time sheets. Economic costs will be estimated for those resources with no financial transactions. All costs will be annuitised over the expected life span of the intervention. Research costs will be excluded.

Health utilities will be measured using the CHU9D, a paediatric generic preference-based measure of health-related quality of life. This consists of a descriptive system and a set of preference weights, giving utility values for each health state described by the descriptive system, allowing the calculation of QALYs. In the absence of country or regional specific weights, the CHU9D will be scored using weights based on Australian adolescent population values. We will follow the OECD guidelines on measuring subjective wellbeing among students, using a single question on overall life satisfaction from 0 “not at all satisfied” to 10 “completely satisfied”).

Incremental cost-effectiveness ratios (ICERs) will be estimated by dividing the difference in mean costs between groups by the difference in mean effects between groups. ICER estimates will be compared with different thresholds. To allow for uncertainty, a series of sensitivity analyses will be performed.

1. **Quality of life measurement and valuation sub-study**

The 3 objectives will be analysed as follows:

**Objective 1:**

- - 1. Internal consistency reliability will be assessed by computing the Cronbach’s alpha (α). I hypothesize that CHU9D will show acceptable to excellent internal consistency at 0.7 ≤ α (51).

1. Construct validity will be determined by computing the Kruskal Wallis test and Mann Whitney U test. It is expected that higher CHU9D utility scores will be associated with low SDQ score; higher SES group; higher life satisfaction and happiness scores; higher general health status rating; and higher quality of life rating.
2. *Feasibility*: the average time participants used to complete the CHU9D and the number of questions without responses will be computed. These will be compared to those reported for the CHU9D and other child-adolescent tools in other settings as reported in published literature.

Quantitative data will be analyzed using STATA version 17. All recordings will be transcribed and analyzed with NVivo software for qualitative analysis.

**Objective 2:**

A multinomial logit model will be used to estimate choice probabilities. The best possible model will be guided by Bayesian information criterion (BIC). A lower BIC (<10) will be considered better than a higher BIC. The mean of the set of CHU9D values will be estimated. Regression analyses will be conducted to assess the factors which influence participants’ CHU9D values. These factors will include participants baseline characteristics (age, sex, SES, geographical location). For adolescent girls, their baseline SDQ, happiness and life-satisfaction scores, as estimated in the MENISCUS trial will also be considered in the regression analysis.

**Objective 3:**

A descriptive analysis of the adolescent girls’ baseline characteristics will be computed by study arm. Adolescent girls’ response to the CHU9D will be summarized in percentages for each of the levels of the 9 dimensions. This will be compared across the study arms. The CHU9D scores for each level of each dimension will be weighted with the Ugandan CHU9D tariffs. These will be summed for each adolescent to generate their overall CHU9D score (QoL score). The mean (and standard deviation) QoL score for each study arm at baseline and endline will be computed and compared across arms. Regression analysis will be conducted to assess the relationship between intervention arm, and QoL scores and how the impact of the intervention on QoL is distributed across SES and geographical location The analysis will be adjusted for clustering by school, restricting variables and factors imbalanced by arm at baseline.

Data will be analysed with STATA version 17. Data and all appropriate documentation will be stored for a minimum of 5 years after the completion of the study, including the follow-up period.

1. **Sleep sub-study**

Phase 1 of the study which aims to inform the intervention design by understanding the contextual factors affecting sleep health will be analysed both quantitatively and qualitatively.

The quantitative survey will be analysed using descriptive analyses and logistic regression to describe the sleep measures and associated factors. The feasibility of administering the tools will be assessed by data completeness, time taken and endline qualitative interviews. Confirmatory factor analysis will be used to check the scale structure, and construct validity will be explored through hypothesised relationships among the sleep measures, and internal consistency using ordinal alpha.

For the qualitative data, we will use thematic analysis, transcripts and field notes will be managed using NVIVO software to inductively identify emerging codes. Meaning units will be abstracted and coded, and the codes compared for similarities and differences and grouped into categories. Key themes will be generated by interpreting categories for their underlying meaning.

## Proposed frequency of analyses

No interim analyses will be performed as the outcomes will only be available at endline.

# monitoring

## Risk assessment

The trial will adhere to the principles of Good Clinical Practice (GCP) and the MRC guidelines for management of global health trials. The study is considered low risk. The anticipated risks and safety reporting procedures are summarised in Section 7 above, and in full in the Risk Assessment Annex (Annex 3). The COVID Risk Management Plan (Annex 4) gives specific details of mitigation of COVID-related risks to the research team and participants, which follow MRC/UVRI and UNCST policy, and will be updated as appropriate.

## Data Monitoring committee

An Independent Data Monitoring and Ethics Committee (IDMEC) has been established. The role of the IDMEC is to safeguard the interests of trial participants, monitor the main outcome measures, and monitor the overall conduct of the trial. This includes to:

- monitor evidence for treatment differences in the main efficacy outcome measures
- monitor evidence for treatment harm (e.g. toxicity, SAEs and SARs, deaths)
- assess data quality, including completeness (and by so doing encourage collection of high quality data)
- maintain confidentiality of all trial information that is not in the public domain
- monitor recruitment figures
- monitor planned sample size assumptions, preferably with regards to a priori assumptions about the control arm outcome

## MoNITORING AT STUDY COORDINATION CEntre/data management

Full details of data entry, management and storage are given in the Data Management Plan (Annex 2). The trial data manager (Ayoub Kakonde) will oversee data entry of quantitative data, quality assurance, metadata creation, and security, in consultation with the Trial Statistician. The social scientist will be responsible for overseeing management of qualitative data. Overall data management responsibility lies with the PI.

## monitoring at local site/ SPONSOR MONITORING

The study sponsor (LSHTM) will organise for monitors to review the source documents as needed, to determine whether the data reported are complete and accurate. The primary objectives of the monitor during the trial will be to educate, support and solve problems. The monitors will discuss the protocol in detail and identify and clarify any areas of weakness. The monitors will audit the overall quality and completeness of the data, examine source documents, interview investigators and coordinators, and confirm that the co-ordinating centre has complied with the requirements of the protocol. The monitors will verify that all adverse events were documented in the correct format, and are consistent with protocol definition.

If a problem is identified during the visit (i.e., inadequate or insufficient staff to conduct the study, missing study documents) the monitor will assist the team in resolving the issues.

# regulatory issues

## Ethics approval

We will seek approval for this protocol, the informed consent forms and draft CRFs from the Uganda Virus Research Institute Research & Ethics Committee (UVRI-REC) and the Uganda National Council of Science and Technology (UNCST) and the LSHTM Research Ethics Committee. The Investigator will make safety and progress reports to the Ethics Committees annually (or as required), and within three months of trial completion.

- - 1. Protocol amendments

Any modifications to the protocol which may impact on the conduct of the study, potential benefit of the patient or may affect patient safety, including changes of study objectives, study design, patient population, sample sizes, study procedures, or significant administrative aspects will require a formal amendment to the protocol. Such amendment will be agreed upon by TMG and TSC, and approved by the Ethics Committee prior to implementation. Administrative changes of the protocol are minor corrections and/or clarifications that have no effect on the way the study is to be conducted. These administrative changes will be agreed upon by the TMG and TSC, and will be documented in a memorandum.

## Assent and CONSENT

- - 1. Informed consent and assent

All team members who will be involved in conducting informed consent and assent will receive training from the study co-ordinator, and will take an online ethics module which includes principles of informed consent. Information sheets and materials and consent forms have been translated into Luganda and back-translated to English. Consent/ assent will be obtained prior to conducting any study procedures.

- - 1. School-level approval

School Head Teachers of potentially eligible schools will be invited to a stakeholder workshop in April-May 2021, where the trial will be explained. Potentially eligible schools will then be visited as part of the rapid assessment, during which head teachers will be asked for their approval to participate in the trial. This provides agreement that the school-level intervention elements will be operationalised (UNEB educational assessment, puberty education training, WASH improvement and the drama skit), as well as formation of the MH Leadership Group.

For the sleep study school-level approval will be sought from the School Head Teachers, who are currently part of the MENISCUS programme and with whom we have good relationships. Schools will be visited as part of the quantitative assessment, during which head teachers will be asked for their formal approval to participate in the study and to sign an MoU.

- - 1. Participant Information

A series of information meetings will be held at all schools after head teacher approval to ensure that students, parents/caregivers of girls, and teachers are fully informed about the study before parental consent and participant assent is sought. Students due to start S2 in 2021 and parents of girls will be invited to study meetings, communicated through the head teacher where the study will be described in detail, offering the chance to ask questions. Parents/caregivers will receive 10,000 Ugandan Shillings (per student) compensation for their time to attend the information meetings. School staff members, teachers, female caregivers and implementors will be asked to take part in interviews at baseline, after intervention delivery and at endline for the process evaluation. All adult participants will be asked for their consent prior to taking part in interviews.

- - 1. Parental consent

Parents of all female students starting S2 in 2021 will be approached at the end of an information meeting at school, at home follow-up visits, or – in the case of COVID-19 restrictions – by phone (see below), to request informed consent:

1. To participate in baseline and endline surveys
2. To participate in qualitative research, , and self-complete a daily diary for school attendance and menstrual cycles, if selected
3. To receive a menstrual health kit (containing reusable pads, underwear, a water bottle and a towel, in a backpack) and analgesic vouchers
4. Separate consent will be sought for girls to receive a menstrual cup (consent for the menstrual cup is optional so parents/caregivers can give consent for other aspects of the trial but not the cup) (Annex 36)

The female parents or caregivers selected to participate in the MH Leadership group will receive a menstrual kit as part of this to assist with advising girls. They will be asked for consent to participate in interviews regarding their participation in these activities (Annex 37).

The parents (males and females) of adolescents (boys and girls) selected to participate in the quality-of-life measurement and valuation sub-study will be asked to consent to participate in the adult CHU9D valuation exercise (explained on page 40).

The team will be trained to administer informed consent, check understanding, and answer any questions. Signing will be private with a trained member of the study team. Reasons for non-consent will be documented. Our pilot data shows high levels of parental consent and student assent (89%) but that collecting parental consent is time-consuming and we have planned to expand the field team temporarily to allow for this. We have allowed 3 months for this process to allow for home visits to parents (July-September 2021). Parental consent will also be sought for any female students present at endline survey in 2023. Additional parental consent (for girls) and student assent (for all students) will occur prior to endline survey for students who were not present at baseline.

We are requesting a waiver for parental consent for boys as the research elements for boys carries no more than minimal risk, that is it involves a brief baseline and endline survey (Annexes 11 and 13) which asks for their factual knowledge of puberty and menstruation. This would be similar to material tested in school biology examinations. We will ask parental consent for boys participating in qualitative interviews (Annex 38). We are also requesting a waiver for parental consent of the networking tool which will ask about social ties between the students and MH group (Annex 51). The data that is being collected is not sensitive information and provides minimal risk to the students. We are requesting for a waiver for parental consent for adolescents (boys and girls) who will participate in the quality-of-life measurement and valuation sub-study as the CHU9D tool to be used is the same tool which was administered at the baseline and endline surveys. The difference here is that the different dimensions and levels of the CHU9D are put together to form different health states and the adolescents are required to select which health state they prefer and/or which attributes of the health state is their best and worst attribute (as shown in tables 7 and 8 (pages 39-40) above). The questions on the CHU9D tool and the health and quality of life questionnaire (Annex 55) provide minimal risk to the students.

For the sleep sub study, we will seek parental consent for their child to participate in the quantitative survey, and possibly to be asked to participant in i) cognitive testing of the quantitative survey 2) qualitative individual or group interviews on the sleep environment 3) receiving CBT-I if they display symptoms of insomnia.

If necessary, for example in the case of continued COVID-19 restrictions that preclude holding parental information meetings or conducting home visits, we will seek informed parental consent by telephone. Contact information for parents of students will be obtained from school head teachers who may assist in making introductions and confirming identity as needed. Research team members will be trained in seeking consent by phone. They will describe the study and communicate the content of the information sheet, answer any questions, and provide a link where the participant can access an online copy of the study information materials. Consent will be documented in a hard-copy log including the date, the name of the parent and participant, and the name of the researcher seeking consent.

- - 1. Student assent

In Q4 2021, prior to the baseline survey, female students will be asked to review the participant information, shown in digital form on a tablet, and to provide secure individual electronic (e-) assent for trial procedures as listed for the parental consent above (Annex 29). Student e-assent will also be sought for any female students present at endline survey in 2023, and an optional opt-in assent for girls to receive the menstrual cup (Annex 35). The male students randomly selected to participate in the baseline and endline surveys or group discussions will also be asked for e-assent (Annex 31).

The participant information will cover the same points outlined in the relevant Annexes (29 and 31) and will be viewed by students on the tablets ahead of the survey or group discussion. Participants will then be asked to indicate their assent using an electronic signature on the tablet. The student will be given a paper sheet stating that they have seen the informational materials and agree to participate, which they will sign and keep. This paper sheet will have the study team’s contact details.

For the quality-of-life measurement and valuation sub-study, the adolescent boys and girls will be asked to provide assent (annexes 57 and 58) before participating the study activities – pre-test, cognitive debriefing, CHU9D valuation exercise and post-CHU9D valuation questionnaire. Information about the study and activities will be provided as outlined in the annexes 57 and 58.

For the sleep study, students will be asked to assent to cognitive testing. Separately student assent will be collected on the tablet for the quantitative survey and the possibility of being asked to participate in 1) qualitative group or individual interviews 2) CBT-I if they report symptoms of insomnia in the quantitative survey.

## Confidentiality

Any participants’ identifiable data collected by the Study Coordination Centre will be stored securely and their confidentiality protected in accordance with the Data Protection Act 1998.

- - 1. Privacy

Personal and medical information relating to research participants will be treated as confidential. The risk of disclosure will be minimized by secure storage of documents and use of linked data by replacing personal identifiers with a unique study code to conceal the identity of participants.

- - 1. Assignment of study IDs

Participants who provide informed assent and whose parents consented will be issued a participant trial identification (PTID) number. The PTID will be used to identify the subject on the CRFs. Each study school will be assigned a number (1-60) and PTID numbers will include the school ID. Numbers will be assigned to participants from student registers. Once a number has been assigned no attempt will be made to use that number again. Students will receive a study ID card to facilitate future identification for post-baseline trial procedures.

- - 1. Privacy of individual

Girls who are symptomatic for a UTI and receive a positive UTI multistix 10 test result will be referred to the local health facility with the test result, to provide point of care. This will include the participants’ PTID to confirm linkage of the test result with the correct individual, and the participant will be asked to show her Study ID card to facilitate this. All personal information will only be available to relevant trained study staff on a need-to-know basis and (only if appropriate) will be recorded in the participants’ medical record book in addition to study CRFs (Annex 26). Participants will be encouraged to bring their parents to the designated health centre when receiving their result.

- - 1. Confidentiality of data

All information regarding the participants will remain confidential to the extent allowed by law. Unique numerical ID codes will be used instead of names or other personal identifiers. All screening forms and case report forms will be kept in a secured location (locked cabinet if paper, password protected if electronic) with access limited to authorised study staff. Unique numerical identifiers will be used for the computer-based data entry and blood samples. Data management will be conducted by the research team members. Co-investigators and researchers will be involved in the analysis. Any shared data with these persons will not include personally identifying information. Reports and publications will not link individual names to data, and study results will be reported as aggregate data. No identifying information will be included. Consent and assent forms will be stored separately in a locked cupboard as will all other forms, logbooks and appointment books that link participant ID numbers to other identifying information.

## Indemnity

London School of Hygiene & Tropical Medicine holds Public Liability ("negligent harm") and Clinical Trial ("non-negligent harm") insurance policies which apply to this trial.

## Sponsor

London School of Hygiene & Tropical Medicine will act as the main sponsor for this study. Delegated responsibilities will be assigned locally.

## Funding

Funding has been provided by the Department of Health and Social Care (DHSC) through the National Institute for Health Research (NIHR), Foreign, Commonwealth and Development Office (FCDO), the Medical Research Council (MRC) and the Wellcome Trust through the Joint Global Health Trials scheme

The quality-of-life sub-study is funded by an unrestricted donation to LSHTM from Reckitt PLC.

The adult CHU9D valuation exercise is funded by the Hamish Ogston Foundation Platinum Jubilee Grants for Early Career Research Professionals.

## Audits and Inspections

The study may be subject to audit by the London School of Hygiene & Tropical Medicine under their remit as Sponsor to ensure adherence to GCP.

## PROTOCOL DEVELOPMENT

This protocol was developed by Ms Laura Hytti with the following investigators who are responsible for the development of, and agreeing to, the final protocol. Subsequent changes to the final protocol will require the agreement of the TSC.

1. Professor Helen Weiss, Chief Investigator, LSHTM, Keppel Street, London, WC1E 7HT, United Kingdom, Email: Helen.Weiss@lshtm.ac.uk, Phone: 0207 927 2087
2. Prof Janet Seeley, Professor of Anthropology and Health, LSHTM, UK and Head of Social Science Programme, MRC/UVRI and LSHTM Email: janet.seeley@lshtm.ac.uk
3. Dr Stella Neema, Senior Lecturer in Anthropology, Makerere University, Email: sheisim@yahoo.com
4. Prof Chris Bonell, Professor of Public Health Sociology, LSHTM, UK, Email: chris.bonell@lshtm.ac.uk
5. Dr Fred Matuvo, Senior Lecturer and Director, PADRI, Makerere University, Kampala Uganda Email: frmatov2000@yahoo.co.uk
6. Prof John Jerrim, Professor of Education and Social Statistics, UCL Institute of Education, University College London, UK, Email: j.jerrim@ucl.ac.uk
7. Dr Belen Torondel, Assistant Professor, Faculty of Infectious and Tropical Diseases, LSHTM, UK. Email: belen.torondel@lshtm.ac.uk
8. Dr Suzanna Francis, Associate Professor, LSHTM, UK, Tel. +44 (0)20 7927 2245, Email: Suzanna.francis@lshtm.ac.uk
9. Dr Clare Tanton, Assistant Professor in Epidemiology, LSHTM, UK, Tel. +44 20 7958 8393, Email: Clare.Tanton@lshtm.ac.uk
10. Dr Giulia Greco, Assistant Professor and MRC Fellow (Health Economics), LSHTM, UK, Email: Giulia.Greco@lshtm.ac.uk
11. Dr Catherine Kansiime, MRC/UVRI and LSHTM P.O.Box 49, Entebbe-Uganda. Plot 51-59 Nakiwogo Road, Email: Catherine.Kansiime@mrcuganda.org
12. Ms Kate Nelson, Research Fellow, LSHTM, UK: Email [kate.nelson@lshtm.ac.uk](mailto:kate.nelson@lshtm.ac.uk)
13. Mr Stephen Lagony, MRC/UVRI and LSHTM P.O.Box 49, Entebbe-Uganda. Plot 51-59 Nakiwogo Road, stephen.lagony@mrcuganda.org
14. Rebecca Prah, Ph.D. student, LSHTM, UK: Email: [rebecca.prah@lshtm.ac.uk](mailto:rebecca.prah@lshtm.ac.uk);

# Trial Management

## Trial Steering Committee (TSC)

A Trial Steering Committee had been set up to act as the oversight body for this trial on behalf of the Sponsor and Funder. The role of the TSC is to provide oversight for the trial. The TSC consists of 7 independent members, including a representative from the Ministry of Education, and 3 Trial Management Group (TMG) members. It will provide advice through its independent Chair (Dr Nelly Mugo) to the TMG and the funding representative (Ms Caroline Harris) who will participate as an observer on the TSC. The TSC will meet 1-2 times per year throughout the trial. The role and membership of the TSC is described in more detail in Annex 1.

## Independent Data Monitoring and Ethics Committee (IDMEC)

An independent Data Monitoring and Ethics Committee has been set up to safeguard the interests of trial participants, monitor the main outcome measures, and monitor the overall conduct of the trial. The IDMEC consist of 3 independent members who are knowledgeable in the subject area, safety monitoring, and clinical trials. They will meet 1-2 times per year during the trial to provide a review of blinded (and if requested unblinded) data to ensure the safety, rights and well-being of trial participants. The role and membership of the IDMEC is described in more detail in Annex 1.

## Trial Management Group (TMG)

A Trial Management Group (TMG) has been appointed and will be responsible for overseeing the progress of the trial. The day-to-day management of the trial will be co-ordinated through the London School of Hygiene & Tropical Medicine Study Coordination Centre. The role and membership of the TMG is described in more detail in Annex 1.

Figure 8: MENISCUS Trial Management structure
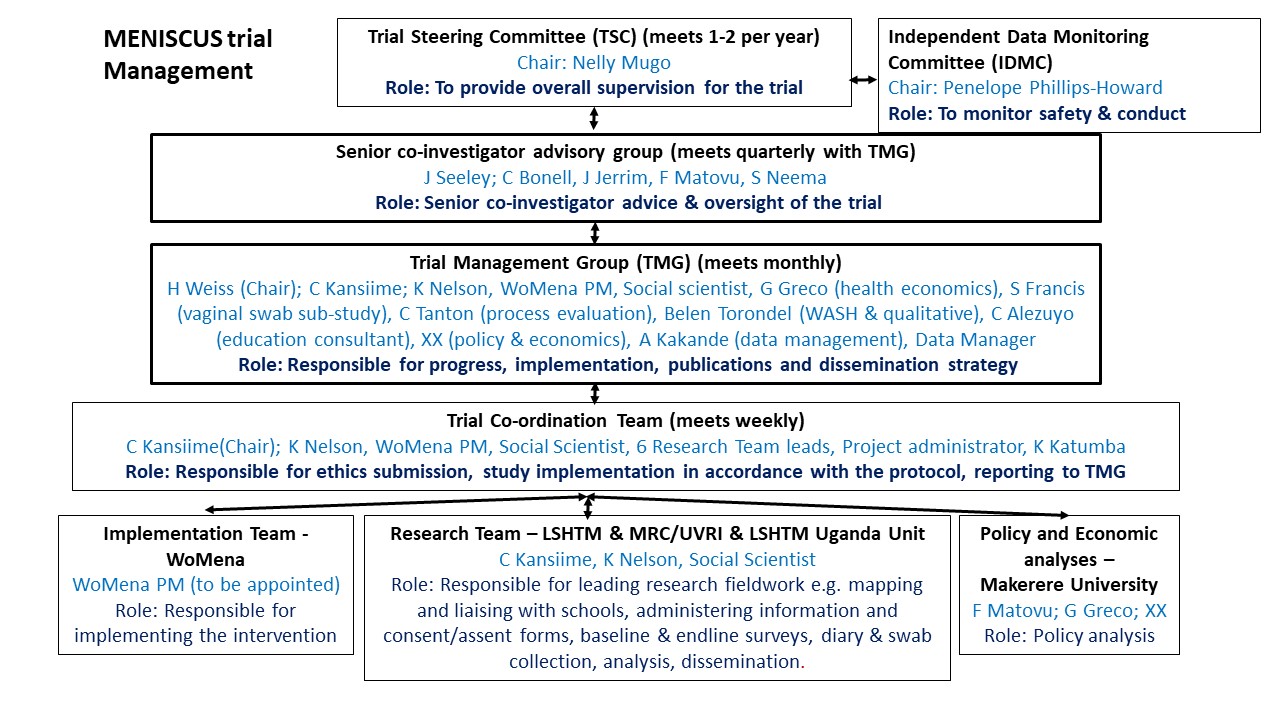


# Stakeholder engagement anD application of results

Our research will be communicated to the public and stakeholders in Uganda, the UK and globally through various means, with an ultimate aim of policy engagement and action to positively impact menstrual health globally.

At the end of the trial, we will visit each school to hold a facilitated discussion about the findings with the students, parents and teachers, and will offer the intervention to schools randomised to the control arm. We will conduct a stakeholder workshop in the final year, including local and national stakeholders (school representatives, parents, community leaders, policy makers from the Ministries of Education and Health, NGOs, and other academic researchers) to discuss policy implications of the findings.

At a national and district level in Uganda the Ministry of Education and Sports and District offices in will be key partners in the study. The intervention has been designed to directly improve teaching of the MHH and puberty education components of the 2018 National Sexuality Education framework in schools using a sustainable Training of Trainers model. We will align with the new Ugandan Life Education Learning Area Syllabus for schools which has sexuality, reproductive and menstrual health components. Other relevant Government policies for the trial include the National Adolescent Health Policy (due for revision, which our results can inform), and the Adolescent Health Policy Guidelines. Our policy analysis will focus on how our intervention can be best aligned with the implementation of these policies and framework and will assess implementation gaps. The policy analysis will also assess whether the schools have the requirements for effective implementation of these policies and suggest how they can be more effectively implemented.

We will engage with the wider public by i) presenting study and findings at the International MHM Day celebrations held every year in Uganda on May 28th; ii) creating a film of the MENISCUS intervention to communicate the message of good MH and the steps that can be taken to improve MH in similar settings; iii) co-creating a youth-friendly MH infographic with study participants which could be shared on social media, the Ugandan Ministry of Education and Sports, and the Ministry of Health, and with Ugandan secondary schools.

We will hold two regional meetings with MH researchers from Kenya, Tanzania and Zimbabwe to share our findings and experiences across MH research projects in each country. These groups will also facilitate links with others working on MH in the region, for example the Duke-UNICEF Innovation Accelerator, of which 5 (of 6) are in East Africa (https://tinyurl.com/rfc6pn2). Communication with the wider academic community will include publication of the trial protocol and results as well as the economic, qualitative, and process evaluations in peer-reviewed academic journals with open access, guided by a dissemination plan.

The main trial results will be launched with a press release from MRC/UVRI, LSHTM and Makerere University, with social media in Uganda, UK and globally. We will present the study results at international meetings including the International Association for Adolescent Health conference, to key audiences (WHO, UNICEF) and funders (including MRC, Wellcome Trust and DFID). We will also communicate our findings through the MRC/UVRI seminar series and at LSHTM through the MARCH (Maternal, Adolescent, Reproductive and Child Health Centre (Weiss is co-lead of the Adolescent Theme of MARCH and we have included 0.1FTE in Year 3 to contribute time for the MARCH Communications Officer to disseminate MENISCUS findings).

# Publication Policy

All publications and presentations relating to the study will be authorised by the TMG. The first publication of the trial results will be in the name of the TMG, if this does not conflict with the journal’s policy. If there are named authors, these will include at least the trial’s Chief Investigator, Statistician and Trial Coordinator. Members of the TSC and the IDMEC will be listed and contributors will be cited by name if published in a journal where this does not conflict with the journal’s policy. Authorship of parallel studies initiated outside of the TMG will be according to the individuals involved in the project but must acknowledge the contribution of the TMG.

# References

1. Sommer M, Caruso BA, Sahin M, Calderon T, Cavill S, Mahon T, et al. A Time for Global Action: Addressing Girls’ Menstrual Hygiene Management Needs in Schools. PLOS Med. 2016 Feb 23;13(2):e1001962.

2. Sommer M, Sahin M. Overcoming the Taboo: Advancing the Global Agenda for Menstrual Hygiene Management for Schoolgirls. Am J Public Health. 2013 Sep;103(9):1556–9.

3. Mason L, Nyothach E, Alexander K, Odhiambo FO, Eleveld A, Vulule J, et al. ‘We Keep It Secret So No One Should Know’ – A Qualitative Study to Explore Young Schoolgirls Attitudes and Experiences with Menstruation in Rural Western Kenya. Molyneux C ‘Sassy’, editor. PLoS ONE. 2013 Nov 14;8(11):e79132.

4. Phillips-Howard PA, Caruso B, Torondel B, Zulaika G, Sahin M, Sommer M. Menstrual hygiene management among adolescent schoolgirls in low- and middle-income countries: research priorities. Glob Health Action. 2016 Dec 1;9(1):33032.

5. McMahon SA, Winch PJ, Caruso BA, Obure AF, Ogutu EA, Ochari IA, et al. ‘The girl with her period is the one to hang her head’ Reflections on menstrual management among schoolgirls in rural Kenya. BMC Int Health Hum Rights. 2011;11:7.

6. Sommer M. Where the education system and women’s bodies collide: The social and health impact of girls’ experiences of menstruation and schooling in Tanzania. J Adolesc. 2010 Aug;33(4):521–9.

7. Sommer M, Hirsch JS, Nathanson C, Parker RG. Comfortably, Safely, and Without Shame: Defining Menstrual Hygiene Management as a Public Health Issue. Am J Public Health. 2015 May 14;105(7):1302–11.

8. Hennegan J, Montgomery P. Do Menstrual Hygiene Management Interventions Improve Education and Psychosocial Outcomes for Women and Girls in Low and Middle Income Countries? A Systematic Review. PLOS ONE. 2016 Feb 10;11(2):e0146985.

9. Sumpter C, Torondel B. A Systematic Review of the Health and Social Effects of Menstrual Hygiene Management. PLOS ONE. 2013 Apr 26;8(4):e62004.

10. Elledge MF, Muralidharan A, Parker A, Ravndal KT, Siddiqui M, Toolaram AP, et al. Menstrual Hygiene Management and Waste Disposal in Low and Middle Income Countries—A Review of the Literature. Int J Environ Res Public Health. 2018 Nov;15(11):2562.

11. AFP. New Vision. 2017 [cited 2020 Dec 28]. Blood And Fury: Menstrual Activism Sweeps The World. Available from: https://www.newvision.co.ug/news/1466683/blood-fury-menstrual-activism-sweeps-world

12. Radnor A. ‘We’re having a menstrual liberation’: how periods got woke. The Guardian [Internet]. 2017 Nov 11 [cited 2020 Dec 28]; Available from: http://www.theguardian.com/society/2017/nov/11/periods-menstruation-liberation-women-activists-abigail-radnor

13. Chris Bobel. The Managed Body- Developing Girls and Menstrual Health in the Global South [Internet]. Palgrave Macmillan; 2019 [cited 2019 Jan 21]. 351 p. Available from: //www.palgrave.com/br/book/9783319894133

14. Hennegan JM. Menstrual Hygiene Management and Human Rights: The Case for an Evidence-Based Approach. Womens Reprod Health. 2017;4(3):212–31.

15. Columbia University, UNICEF. ‘MHM in Ten’: Advancing the MHM Agenda in WASH in Schools. In 2014. Available from: https://www.unicef.org/wash/schools/files/MHM_in_Ten_2014_Meeting_Report.pdf

16. Wodon Q, Montenegro C, Nguyen H, Onagoruwa A. MISSED OPPORTUNITIES: THE HIGH COST OF NOT EDUCATING GIRLS. Washington C: World Bank; 2018. (The Cost of Not Educating Girls Notes Series).

17. MoEST. Menstruation: Breaking the silence, taking action. [Internet]. 2015. Available from: https://www.ircwash.org/sites/default/files/20151007_mhm_publication_final_for_web.pdf

18. Ministry of Education, Science, Technology and Sports (MoESTS). Menstrual Hygiene Management Charter - Uganda 2015 [Internet]. Uganda: MoESTS; 2015. Available from: https://www.ircwash.org/sites/default/files/menstrual_hygiene_management_charter_finalised_april_2015_1_.pdf

19. Ali C. Provision of menstrual hygiene management facilities for girls and female teachers in primary and secondary schools in Uganda : study on implementation of the Ministry of Education and Sports circular. Kampala, Uganda: IRC Uganda and the Ministry of Education and Sports; 2018. p. 67 p. : 4 fig., 9 tab.

20. Hennegan J, Shannon AK, Rubli J, Schwab KJ, Melendez-Torres GJ. Women’s and girls’ experiences of menstruation in low- and middle-income countries: A systematic review and qualitative metasynthesis. PLOS Med. 2019 May 16;16(5):e1002803.

21. Miiro G, Rutakumwa R, Nakiyingi-Miiro J, Nakuya K, Musoke S, Namakula J, et al. Menstrual health and school absenteeism among adolescent girls in Uganda (MENISCUS): a feasibility study. BMC Womens Health. 2018 Jan 3;18:4.

22. Kansiime C, Hytti L, Nalugya R, Nakuya K, Namirembe P, Nakalema S, et al. Menstrual health intervention and school attendance in Uganda (MENISCUS-2): a pilot intervention study. BMJ Open. 2020 Feb 1;10(2):e031182.

23. Langford R, Bonell C, Jones H, Pouliou T, Murphy S, Waters E, et al. The World Health Organization’s Health Promoting Schools framework: a Cochrane systematic review and meta-analysis. BMC Public Health. 2015 Feb 12;15(1):130.

24. Geertz A, Iyer L, Karen P, Mazzola F, Peterson K. An opportunity to address menstrual health and gender equity. FSG; 2016.

25. Asiki G, Murphy G, Nakiyingi-Miiro J, Seeley J, Nsubuga RN, Karabarinde A, et al. The general population cohort in rural south-western Uganda: a platform for communicable and non-communicable disease studies. Int J Epidemiol. 2013 Feb 1;42(1):129–41.

26. O’Donoghue J, Crawfurd L, Makaaru J, Otieno P, Perakis R. A review of Uganda’s Universal Secondary Education Public Private Partnership programme. :80.

27. Uganda Bureau of Statistics. The National Population and Housing Census 2014 – Area Specific Profile Series - Wakiso, Kampala, Uganda. Kampala, Uganda: Uganda Bureau of Statistics; 2017.

28. Uganda Bureau of Statistics. The National Population and Housing Census 2014 – Area Specific Profile Series - Kalungu. Kampala, Uganda.: Uganda Bureau of Statistics; 2017.

29. Hoosen N, Davids EL, de Vries PJ, Shung-King M. The Strengths and Difficulties Questionnaire (SDQ) in Africa: a scoping review of its application and validation. Child Adolesc Psychiatry Ment Health. 2018 Jan 11;12(1):6.

30. Goodman R. The Strengths and Difficulties Questionnaire: A Research Note. J Child Psychol Psychiatry. 1997 Jul;38(5):581–6.

31. Hennegan J, Nansubuga A, Smith C, Redshaw M, Akullo A, Schwab KJ. Measuring menstrual hygiene experience: development and validation of the Menstrual Practice Needs Scale (MPNS-36) in Soroti, Uganda. BMJ Open. 2020 Feb 1;10(2):e034461.

32. Hunter EC, Murray SM, Sultana F, Alam MU, Sarker S, Rahman M, et al. Development and validation of the Self-Efficacy in Addressing Menstrual Needs Scale (SAMNS-26) in Bangladeshi schools: A measure of girls’ menstrual care confidence. PLOS ONE. 2022 Oct 6;17(10):e0275736.

33. Ratcliffe J, Huynh E, Chen G, Stevens K, Swait J, Brazier J, et al. Valuing the Child Health Utility 9D: Using profile case best worst scaling methods to develop a new adolescent specific scoring algorithm. Soc Sci Med 1982. 2016 May;157:48–59.

34. International Association for the Evaluation of Educational Achievement (IEA). Trends in International Mathematics and Science Study - TIMSS 2011. Boston: TIMSS & PIRLS Internatinoal Study Center, Lynch School of Education; 2012.

35. UNICEF, WHO. Drinking water, sanitation and hygiene in schools: global baseline report 2018. New York; 2018.

36. Hahn S, Puffer S, Torgerson DJ, Watson J. Methodological bias in cluster randomised trials. BMC Med Res Methodol. 2005 Mar 2;5(1):10.

37. Ashbeck EL, Bell ML. Single time point comparisons in longitudinal randomized controlled trials: power and bias in the presence of missing data. BMC Med Res Methodol. 2016 Apr 12;16(1):43.

38. Ministry of Education and Sports. Uganda National Sexuality Education Framework. MoES Uganda; 2018.

39. Hennegan J, Dolan C, Steinfield L, Montgomery P. A qualitative understanding of the effects of reusable sanitary pads and puberty education: implications for future research and practice. Reprod Health. 2017 Jun 27;14(1):78.

40. van Eijk AM, Laserson KF, Nyothach E, Oruko K, Omoto J, Mason L, et al. Use of menstrual cups among school girls: longitudinal observations nested in a randomised controlled feasibility study in rural western Kenya. Reprod Health. 2018 Aug 17;15(1):139.

41. Gade A, Hytti L. Menstrual health in Rhino Camp refugee settlement, West Nile, Uganda. Pilot project intervention report. WoMena Uganda and ZOA,; 2017.

42. Hyttel M, Faldt Thomsen C, Luff B, Storrusten H, Nyakato VN, Tellier M. Drivers and Challenges to Use of Menstrual Cups Among Schoolgirls in Rural Uganda: A Qualitative Study. Waterlines Pract Action Publ. 2017;36(2):16.

43. Juma J, Nyothach E, Laserson KF, Oduor C, Arita L, Ouma C, et al. Examining the safety of menstrual cups among rural primary school girls in western Kenya: observational studies nested in a randomised controlled feasibility study. BMJ Open. 2017 May;7(4):e015429.

44. Todd J, Fishaut M, Kapral F, Welch T. TOXIC-SHOCK SYNDROME ASSOCIATED WITH PHAGE-GROUP-I STAPHYLOCOCCI. The Lancet. 1978 Nov 25;312(8100):1116–8.

45. Eijk AM van, Zulaika G, Lenchner M, Mason L, Sivakami M, Nyothach E, et al. Menstrual cup use, leakage, acceptability, safety, and availability: a systematic review and meta-analysis. Lancet Public Health [Internet]. 2019 Jul 16 [cited 2019 Jul 18];0(0). Available from: https://www.thelancet.com/journals/lanpub/article/PIIS2468-2667(19)30111-2/abstract

46. UNCST. National Guidelines for Conduct of Research during Coronavirus Disease 2019 (COVID-19) Pandemic. 2020.

47. Common Terminology Criteria for Adverse Events (CTCAE). 2017;155.

48. Phillips-Howard PA, Nyothach E, Ter Kuile FO, Omoto J, Wang D, Zeh C, et al. Menstrual cups and sanitary pads to reduce school attrition, and sexually transmitted and reproductive tract infections: a cluster randomised controlled feasibility study in rural Western Kenya. BMJ Open. 2016 Nov 6;6(11):e013229.

49. Francis SC, Miiro G, Nakuya K, Rutakumwa R, Nakiyingi-Miiro J, Nabaggala G, et al. Self-Collection of Vaginal Swabs Among Adolescent Girls in a School-Setting in East Africa. Sex Transm Dis. 2019 May;46(5):335–41.

50. Moore GF, Audrey S, Barker M, Bond L, Bonell C, Hardeman W, et al. Process evaluation of complex interventions: Medical Research Council guidance. The BMJ [Internet]. 2015 Mar 19 [cited 2021 Jan 5];350. Available from: https://www.ncbi.nlm.nih.gov/pmc/articles/PMC4366184/

51. May C. Towards a general theory of implementation. Implement Sci. 2013 Feb 13;8(1):18.

52. Education Endowment Foundation [Internet]. [cited 2021 Jan 5]. Impact. Available from: https://educationendowmentfoundation.org.uk/evidence-summaries/about-the-toolkits/attainment

53. Campbell MK, Piaggio G, Elbourne DR, Altman DG, CONSORT Group. Consort 2010 statement: extension to cluster randomised trials. BMJ. 2012 Sep 4;345:e5661.

# AnnexeS

| 1 | Composition of IDMEC/TSC/TMG |
| --- | --- |
| 2 | Data Management Plan |
|  |  |
| 3 | Risk Assessment |
| 4 | COVID Risk Management Plan |
| 5a | Product Brochure - AFRIpads |
| 5b | Product Brochure – Ruby Cups |
| 6a | Safe use and care guidelines for product use - AFRIpads |
| 6b | Safe use and care guidelines for product use - Ruby Cup |
| 6c | Safe use and care guidelines for product use - Pain Killers |
| 8a | Information leaflet on signs and symptoms of mTSS and seeking care |
| 8b | Check list for school nurses and local clinical staff on signs and symptoms of mTSS |
| 9 | Safeguarding Policy |
| Case Report Forms | |
| 10 | CRF 1 - Baseline Survey - Girls |
| 11 | CRF 2 - Baseline Survey - Boys |
| 12 | CRF 3 – Endline Survey - Girls |
| 13 | CRF 4 - Endline Survey - Boys |
| 14 | CRF 5 – Semi-structured Interview Guide - senior staff member |
| 15 | CRF 6 – Semi-structured Interview Guide - MH Leadership Groups |
| 16 | CRF 7 - Semi-structured Interview Guide – Female Students |
| 17 | CRF 8 - Semi-structure Interview Guide - Teachers |
| 18 | CRF 9 - Semi-structured Interview Guide - Implementor |
| 18a | CRF 9a - Semi-structured Interview Guide – Female Caregivers |
| 19 | CRF 10 – Focus Group Discussion Guide - Teachers |
| 20 | CRF 11– Focus Group Discussion Guide - Female Students |
| 21 | CRF 12 – Focus Group Discussion Guide - Male Students |
| 22 | CRF 13 - WASH Spot checks MHM Observation visit checklist |
| 23 | CRF 14 - Drama Skit Structured Observation Form |
| 23a | CRF 14a – Training Structured Observation Form |
| 24 | CRF 15 – Analgesics Voucher |
| 25 | CRF 16 - Daily Diary |
| 26 | CRF 17 – Semi-structured Interview Guide - Diary Participants |
| 27 | CRF 18 – Adverse Events Log Form |
| 28 | CRF 19 – Serious Adverse Event Reporting Form |
| 47 | CRF 20 – Pre-test Focus Group Discussion Guide - Teachers |
| Informed Consent Forms | |
| 29 | ICF 1 – Assent for girls |
| 30 | ICF 2 - Parental Consent for girls |
| 31 | ICF 3 – Assent for boys |
| 32 | ICF 4 – Teacher Consent |
| 35 | ICF 7 – Optional Menstrual Cup assent for girls |
| 36 | ICF 8 – Optional Menstrual Cup consent for parents of girls |
| 37 | ICF 9 - Female caregiver/Staff consent for menstrual cup |
| 38 | ICF 10 - Parental consent for boys for FGDs |
| 39 | ICF11 – Paper information sheet for students to keep following e-assent |
| 40 | ICF 12 – Caregiver/Staff consent for MH Kit |
| 41 | ICF 13 – Parental consent for MH Kit for prefects and AG members |
| 42 | ICF 14 – Student assent for MH Kit for prefects and AG members |
| 43 | ICF 15 – Parental consent for menstrual cup for prefects and AG members |
| 44 | ICF 16 – Student assent for menstrual cup for prefects and AG members |
| 45 | ICF 17 – Head teacher consent for school-level participation in the intervention |
| 46 | ICF 18 – Teacher consent for pre-test workshop |

1. Estimated using random effects logistic regression to compare endline vs baseline measures; adjusted for within-girl clustering; [↑](#footnote-ref-2)
2. Defined as re-usable or disposable pads, tampons or menstrual cups [↑](#footnote-ref-3)
3. The sample size of 60 schools is based on an estimated school size of ~60 female students per school (harmonic mean) seen at endline. This allows for expected decreases in school enrolment following COVID-19 school closures. If registers obtained before the baseline survey indicate that schools are larger than expected, the number of schools included in the trial may be revised down to maintain a similar number of individual participants. (Additional detail included in section 10.1, below). [↑](#footnote-ref-4)
4. Schools with basic access to water and sanitation facilities according JMP/WHO definition: 1) Access to drinking water: there is drinking water from an improved source* available at the school at the time of the survey. 2) Access to sanitation: there are improved sanitation^ facilities at the school that are single-sex and usable (available to female students, functional and private) at the time of the survey, or can be made private with minor door repairs. (*Improved water sources include: piped water, borehole or tubewells, protected dug wells, protected springs and packaged or delivered water; ^Improved sanitation facilities include: flush/pour flush toilets, ventilated improved pit latrines, composting toilets and pit latrines with a slab) (35) [↑](#footnote-ref-5)
5. Based on the revised school calendar set out in the MoES statement on 19^th^ May 2021 [↑](#footnote-ref-6)
6. Ssemata AS, Ndekezi D, Kansiime C, Bakanoma R, Tanton C, Nelson KA, et al. Understanding the social and physical menstrual health environment of secondary schools in Uganda: A qualitative methods study. PLOS Glob Public Health. 2023;3(11):e0002665. Epub 20231129. doi: 10.1371/journal.pgph.0002665. PubMed PMID: 38019777; PubMed Central PMCID: PMCPMC10686490. [↑](#footnote-ref-7)
7. Bastien CH, Vallieres A, Morin CM. Validation of the Insomnia Severity Index as an outcome measure for insomnia research. Sleep Med. 2001;2(4):297-307. Epub 2001/07/05. doi: 10.1016/s1389-9457(00)00065-4. PubMed PMID: 11438246. [↑](#footnote-ref-8)
8. Roenneberg T, Kuehnle T, Juda M, Kantermann T, Allebrandt K, Gordijn M, et al. Epidemiology of the human circadian clock. Sleep Med Rev. 2007;11(6):429-38. Epub 2007/10/16. doi: 10.1016/j.smrv.2007.07.005. PubMed PMID: 17936039. [↑](#footnote-ref-9)
9. Gregory AM, Cox J, Crawford MR, Holland J, Harvey AG, Steps T. Dysfunctional beliefs and attitudes about sleep in children. J Sleep Res. 2009;18(4):422-6. Epub 20090816. doi: 10.1111/j.1365-2869.2009.00747.x. PubMed PMID: 19686237. [↑](#footnote-ref-10)
10. [↑](#footnote-ref-11)
11. Egbegi DR, Bella-Awusah T, Omigbodun O, Ani C. A controlled trial of Cognitive Behavioural Therapy-based strategies for insomnia among in-school adolescents in southern Nigeria. Child Adolesc Psychiatry Ment Health. 2021;15(1):52. Epub 2021/09/27. doi: 10.1186/s13034-021-00406-1. PubMed PMID: 34563220; PubMed Central PMCID: PMCPMC8465787. [↑](#footnote-ref-12)
12. Carney CE, Buysse DJ, Ancoli-Israel S, Edinger JD, Krystal AD, Lichstein KL, et al. The consensus sleep diary: standardizing prospective sleep self-monitoring. Sleep. 2012;35(2):287-302. Epub 2012/02/02. doi: 10.5665/sleep.1642. PubMed PMID: 22294820; PubMed Central PMCID: PMCPMC3250369. [↑](#footnote-ref-13)
