# Supplementary material for: Associations of menstrual health with school absenteeism and examination performance among Ugandan secondary school students: A prospective study
Source: PLoS One. 2026 Jan 23;21(1):e0326549. doi: 10.1371/journal.pone.0326549 (PMC12829969; doi:10.1371/journal.pone.0326549)
Supplement: Attachment — Submitted filename: PONE-D-25-29440.EAO 14JUL2025docx.docx [file pone.0326549.s005.docx]

**PLOS ONE**

**Associations of menstrual health with school absenteeism and educational performance among Ugandan secondary school students: A longitudinal study**

--Manuscript Draft--

| **Manuscript Number:** | PONE-D-25-29440 |
| --- | --- |
| **Article Type:** | Clinical Trial |
| **Full Title:** | Associations of menstrual health with school absenteeism and educational performance among Ugandan secondary school students: A longitudinal study |
| **Short Title:** | Associations of menstrual health with school absenteeism and educational performance |
| **Corresponding Author:** | Christopher Baleke, MSc  MRC/UVRI Uganda Research Unit On AIDS: MRC/UVRI and LSHTM Uganda Research Unit  Kampala, UGANDA |
| **Keywords:** | Menstrual health, school absenteeism, academic performance, education |
| **Abstract:** | Background: Relatively few studies have quantified the amount of school missed due to poor menstrual health, or the impact of menstrual health on educational performance.  Methods: We analysed baseline and longitudinal data from participants in a cluster- randomised trial of a menstrual health intervention in 60 Ugandan secondary schools. We measured school absenteeism as the self-reported number of days absent due to menstruation per month and assessed educational performance using examination results, at baseline and endline. We estimated adjusted incidence rate ratios (aIRR) for associations with school absenteeism, using negative binomial regression adjusted for school-level clustering. We estimated adjusted standardsied mean differences (aSMD) in examination scores using mixed-effects linear regression.  Results: At baseline, 3312 participants reported menstruating in the past 6 months (mean age 15.6 years). Of these, 323 (9.8%) participants reported missing at least one day of school per month due to menstruation and 1286 (38.8%) missed at least one day per month for any reason. Of the 1192 participants in the control arm seen at endline, 135 (11.3%) missed at least one day due to menstruation (mean days missed=0.30 per month (95%CI 0.27-0.34)). Menstrual-related absenteeism and poorer examination performance at endline were both associated with multiple dimensions of menstrual health (use of inadequate menstrual materials, negative menstrual attitudes, unmet menstrual practice needs, and experience of menstrual teasing). In addition, absenteeism (due to menstruation and overall) was associated with menstrual pain.  Conclusion: Among Ugandan students, multiple dimensions of menstrual health are associated with school absenteeism and educational performance. |
| **Order of Authors:** | Christopher Baleke, MSc |
|  | Levicatus Mugenyi, MSc, PhD |
|  | Kate A Nelson, MSc |
|  | Katherine A Thomas, MSc |
|  | Denis Ndekezi, MSc |
|  | Jonathan Reuben Enomut, BA |
|  | Connie Alezuyo, MSc |
|  | John Jerrim, MSc, PhD |
|  | Helen A Weiss, MSc, PhD |
| **Additional Information:** |  |
| **Question** | **Response** |

| **Financial Disclosure**  Enter a financial disclosure statement that describes the sources of funding for the work included in this submission. Review the [submission guidelines](http://journals.plos.org/plosone/s/submission-guidelines#loc-financial-disclosure-statement) for detailed requirements. View published research articles from [*PLOS ONE*](https://journals.plos.org/plosone/) for specific examples.  This statement is required for submission and **will appear in the published article** if the submission is accepted. Please make sure it is accurate. | Yes |
| --- | --- |
| **Funded studies**  Enter a statement with the following details:   - Initials of the authors who received each award - Grant numbers awarded to each author - The full name of each funder - URL of each funder website - Did the sponsors or funders play any role in the study design, data collection and analysis, decision to publish, or preparation of the manuscript? |  |
| Did you receive funding for this work? |  |
| Please add funding details.  as follow-up to "**Financial Disclosure**  Enter a financial disclosure statement that describes the sources of funding for the work included in this submission. Review the [submission guidelines](http://journals.plos.org/plosone/s/submission-guidelines#loc-financial-disclosure-statement) for detailed requirements. View published research articles from [*PLOS ONE*](https://journals.plos.org/plosone/) for specific examples.  This statement is required for submission and **will appear in the published article** if the submission is accepted. Please make sure it is accurate. | This work was supported by the Joint Global Health Scheme with funding from the UK Foreign, Commonwealth and Development Office, the UK Medical Research Council, the UK Department of Health and Social Care through the National Institute of Health Research (NIHR), and Wellcome (Grant Ref. MR/ V005634/1). The funder had no role in the design of the study; in the collection, analyses, or interpretation of the data; in the writing of the article; or in the decision to publish the results. |

| **Funded studies**  Enter a statement with the following details:   - Initials of the authors who received each award - Grant numbers awarded to each author - The full name of each funder - URL of each funder website - Did the sponsors or funders play any role in the study design, data collection and analysis, decision to publish, or preparation of the manuscript? |  |
| --- | --- |
| Did you receive funding for this work?" |  |
| Please select the country of your main research funder (please select carefully as in some cases this is used in fee calculation).  as follow-up to "**Financial Disclosure**  Enter a financial disclosure statement that describes the sources of funding for the work included in this submission. Review the [submission guidelines](http://journals.plos.org/plosone/s/submission-guidelines#loc-financial-disclosure-statement) for detailed requirements. View published research articles from [*PLOS ONE*](https://journals.plos.org/plosone/) for specific examples.  This statement is required for submission and **will appear in the published article** if the submission is accepted. Please make sure it is accurate. | UNITED KINGDOM - GB |
| **Funded studies**  Enter a statement with the following details:   - Initials of the authors who received each award - Grant numbers awarded to each author - The full name of each funder - URL of each funder website - Did the sponsors or funders play any role in the study design, data collection and analysis, decision to publish, or preparation of the manuscript? |  |
| Did you receive funding for this work?" |  |
| **Competing Interests** | The authors declare that they have no competing interests. |

| Use the instructions below to enter a competing interest statement for this submission. On behalf of all authors, disclose any [competing interests](http://journals.plos.org/plosone/s/competing-interests) that could be perceived to bias this work—acknowledging all financial support and any other relevant financial or non- financial competing interests.  This statement is required for submission and **will appear in the published article** if the submission is accepted. Please make sure it is accurate and that any funding sources listed in your Funding Information later in the submission form are also declared in your Financial Disclosure statement.  View published research articles from  [*PLOS ONE*](http://journals.plos.org/plosone/) for specific examples. |  |
| --- | --- |
| **NO authors have competing interests**  Enter: *The authors have declared that no competing interests exist.*  **Authors with competing interests**  Enter competing interest details beginning with this statement:  *I have read the journal's policy and the authors of this manuscript have the following competing interests: [insert competing interests here]* |  |
| * typeset |  |
| **Ethics Statement**  Enter an ethics statement for this submission. This statement is required if the study involved:   - Human participants - Human specimens or tissue - Vertebrate animals or cephalopods - Vertebrate embryos or tissues | Ethical approval for MENISCUS protocol, the informed consent forms and draft CRFs were obtained from the Uganda Virus Research Institute Research & Ethics Committee (UVRI-REC) (reference GC/127/819) and the Uganda National Council of Science and Technology (UNCST) (reference HS1525ES) and the LSHTM Research Ethics Committee (reference 22952-2). |

| - Field research   Write "N/A" if the submission does not require an ethics statement.  General guidance is provided below. Consult the [submission guidelines](http://journals.plos.org/plosone/s/submission-guidelines#loc-guidelines-for-specific-study-types) for detailed instructions. **Make sure that all information entered here is included in the Methods section of the manuscript.** |  |
| --- | --- |

| **Format for specific study types**  **Human Subject Research (involving human participants and/or tissue)**   - Give the name of the institutional review board or ethics committee that approved the study - Include the approval number and/or a statement indicating approval of this research - Indicate the form of consent obtained (written/oral) or the reason that consent was not obtained (e.g. the data were analyzed anonymously)   **Animal Research (involving vertebrate animals, embryos or tissues)**   - Provide the name of the Institutional Animal Care and Use Committee (IACUC) or other relevant ethics board that reviewed the study protocol, and indicate whether they approved this research or granted a formal waiver of ethical approval - Include an approval number if one was obtained - If the study involved *non-human primates*, add *additional details* about animal welfare and steps taken to ameliorate suffering - If anesthesia, euthanasia, or any kind of animal sacrifice is part of the study, include briefly which substances and/or methods were applied   **Field Research**  Include the following details if this study involves the collection of plant, animal, or other materials from a natural setting:   - Field permit number - Name of the institution or relevant body that granted permission |  |
| --- | --- |
|  |  |
| **Data Availability**  Authors are required to make all data underlying the findings described fully available, without restriction, and from the time of publication. PLOS allows rare exceptions to address legal and ethical concerns. See the [PLOS Data Policy](http://journals.plos.org/plosone/s/data-availability) and [FAQ](http://journals.plos.org/plosone/s/data-availability#loc-faqs-for-data-policy) for detailed information. | Yes - all data are fully available without restriction |

| A Data Availability Statement describing where the data can be found is required at submission. Your answers to this question constitute the Data Availability Statement and **will be published in the article**, if accepted. |  |
| --- | --- |
| **Important:** Stating ‘data available on request from the author’ is not sufficient. If your data are only available upon request, select ‘No’ for the first question and explain your exceptional situation in the text box. |  |
| Do the authors confirm that all data underlying the findings described in their manuscript are fully available without restriction? |  |
| **Describe where the data may be found in full sentences. If you are copying our sample text, replace any instances of XXX with the appropriate details.**   - If the data are **held or will be held in a public repository**, include URLs, accession numbers or DOIs. If this information will only be available after acceptance, indicate this by ticking the box below. For example: *All XXX files are available from the XXX database (accession number(s) XXX, XXX.*). - If the data are all contained **within the manuscript and/or Supporting Information files**, enter the following: *All relevant data are within the manuscript and its Supporting Information files.* - If neither of these applies but you are able to provide **details of access elsewhere**, with or without limitations, please do so. For example:   *Data cannot be shared publicly because of [XXX]. Data are available from the XXX Institutional Data Access / Ethics Committee (contact via XXX) for researchers who meet the criteria for access to confidential data.*  *The data underlying the results presented in the study are available from (include the name of the third party* | The data are held in a public repository here: LSHTM Data Compass  Weiss, H, Nelson, K, Mugenyi, L, Batuusa, L, Thomas, K and Baleke, C (2024). MENISCUS Trial. [Project]. London School of Hygiene & Tropical Medicine, London, United Kingdom. https://doi.org/10.17037/DATA.00003822. |

| *and contact information or URL).*   - This text is appropriate if the data are owned by a third party and authors do not have permission to share the data.   * typeset |  |
| --- | --- |
| Additional data availability information: | Tick here if the URLs/accession numbers/DOIs will be available only after acceptance of the manuscript for publication so that we can ensure their inclusion before publication. |

Manuscript	[Click here to access/download;Manuscript;Manuscript.docx](https://www2.cloud.editorialmanager.com/pone/download.aspx?id=41010621&guid=e8ab620b-9912-483c-9754-20c180b7b7d1&scheme=1)[<https://www2.cloud.editorialmanager.com/pone/download.aspx?id=41010621&guid=e8ab620b-9912-483c-9754-20c180b7b7d1&scheme=1>](https://www2.cloud.editorialmanager.com/pone/download.aspx?id=41010621&guid=e8ab620b-9912-483c-9754-20c180b7b7d1&scheme=1)

1 **Title:** Associations of menstrual health with school absenteeism and educational performance
2 among Ugandan secondary school students: A longitudinal study

3

4 This study is conducted under a MENISCUS with **Trial Registration and Recruitment**

### 5 Details:

6 **Registry Name:** ISRCTN
7 **Registration Number:** ISRCTN45461276
8 **DOI:** <https://doi.org/10.1186/ISRCTN45461276>
9 **Recruitment Start Date:** 21/03/2022
10 **Recruitment End Date:** 31/03/2023
11 The trial paper has been published here:	doi: **10.1016**/**S2214**-**109X**(**25**)**00007-5** with its
12 CONSORT

13

14 **Authors:** Christopher Baleke^a^, Levicatus Mugenyi^a^, Kate A Nelson^b^, Katherine A Thomas^b^,
15 Denis Ndekezi^a^, Jonathan Reuben Enomut^a^, Connie Alezuyo^c^, John Jerrim^d^, Helen A Weiss^b^

16

### 17 Affiliations:

18 ^a^ MRC/UVRI and LSHTM Uganda Research Unit, PO Box 49, Entebbe, Plot 51-59
19 Nakiwogo Road, Uganda ([Christopher.baleke@lshtm.ac.uk](mailto:Christopher.baleke@lshtm.ac.uk);
20 [Levicatus.mugenyi@mrcuganda.org,](mailto:Levicatus.mugenyi@mrcuganda.org) [denis.ndekezi@mrcuganda.org,](mailto:denis.ndekezi@mrcuganda.org) [enomutj@gmail.com](mailto:enomutj@gmail.com))
21 ^b^ International Statistics and Epidemiology Group; Faculty of Epidemiology and Population
22 Health, London School of Hygiene & Tropical Medicine, Keppel Street, London, WC1E
23 7HT,	United	Kingdom	([kate.nelson@lshtm.ac.uk](mailto:kate.nelson@lshtm.ac.uk);	[katherine.thomas1@lshtm.ac.uk](mailto:katherine.thomas1@lshtm.ac.uk);
24 [helen.weiss@lshtm.ac.uk](mailto:helen.weiss@lshtm.ac.uk) )
25 ^c^ Ministry of Education and Sports, Uganda, P. O. BOX 7063 Kampala, Uganda King George
26 VI Way, Embassy House, Uganda ([calezuyo@gmail.com](mailto:calezuyo@gmail.com) )
27 ^d^ UCL Institute of Education, University College London, 20 Bedford Way, London WC1H
28 0AL, United Kingdom ([j.jerrim@ucl.ac.uk](mailto:j.jerrim@ucl.ac.uk) )

29

30 *Correspondence to be addressed to:
31 Christopher Baleke
32 Email: [Christopher.baleke@lshtm.ac.uk](mailto:Christopher.baleke@lshtm.ac.uk)

33

34 **Title:** Associations of menstrual health with school absenteeism and educational performance
35 among Ugandan secondary school students: A longitudinal study

### 36 ABSTRACT

37 **Background:** Relatively few studies have quantified the amount of school missed due to
38 poor menstrual health, or the impact of menstrual health on educational performance.
39 **Methods:** We analysed baseline and longitudinal data from participants in a cluster-
40 randomised trial of a menstrual health intervention in 60 Ugandan secondary schools. We
41 measured school absenteeism as the self-reported number of days absent due to menstruation
42 per month and assessed educational performance using examination results, at baseline and
43 endline. We estimated adjusted incidence rate ratios (aIRR) for associations with school
44 absenteeism, using negative binomial regression adjusted for school-level clustering. We
45 estimated adjusted standardised mean differences (aSMD) in examination scores using
46 mixed-effects linear regression.
47 **Results:** At baseline, 3312 participants reported menstruating in the past 6 months (mean age
48 15.6 years). Of these, 323 (9.8%) participants reported missing at least one day of school per
49 month due to menstruation and 1286 (38.8%) missed at least one day per month for any
50 reason. Of the 1192 participants in the control arm seen at endline, 135 (11.3%) missed at
51 least one day due to menstruation (mean days missed=0.30 per month (95%CI 0.27-0.34)).
52 Menstrual-related absenteeism and poorer examination performance at endline were both
53 associated with multiple dimensions of menstrual health (use of inadequate menstrual
54 materials, negative menstrual attitudes, unmet menstrual practice needs, and experience of
55 menstrual teasing). In addition, absenteeism (due to menstruation and overall) was associated
56 with menstrual pain.
57 **Conclusion:** Among Ugandan students, multiple dimensions of menstrual health are
58 associated with school absenteeism and educational performance.

### 59 Keywords

60 Menstrual health, school absenteeism, academic performance, education

61

62

63

### 64 1. Introduction

65 Menstrual health (MH) is defined as complete physical, mental, and social wellbeing in
66 relation to the menstrual cycle (1) . Poor menstrual health is prevalent in low- and middle-
67 income countries (LMICs), and a review of qualitative studies in LMICs found that multiple
68 aspects of menstrual experience contribute to school absenteeism, including inadequate
69 menstrual materials, poor menstrual knowledge and confidence, menstrual pain, and
70 inadequate infrastructure for managing menstruation in schools (2).
71 A widely-cited statistic that “one in 10 school-age girls in Africa misses school or drops out
72 for reasons related to her period” is not evidence-based (3, 4). A systematic review of 15
73 quantitative studies on school absenteeism during menstruation in sub-Saharan Africa found
74 that 31% (95%CI 24-39%) of female students reported school absenteeism during
75 menstruation either at last menstrual period (LMP) or over a longer duration, and that
76 menstruation also affected concentration, academic performance, and participation in sports
77 (5). However, the number of school days missed were not quantified and there was a high
78 degree of unexplained heterogeneity between studies (I^2^=97.8%) with prevalence of
79 absenteeism due to menstrual pain ranging from 6% in Ghana to 65% in Ethiopia. The
80 reference period for measurement of school absenteeism varied, with some using LMP and
81 others using longer durations - this may partly be responsible to variations in prevalence. A
82 recent multi-country study of data from 673,380 women and girls aged 15-49 in 47 countries
83 found that the proportion reporting menstrual-related school absenteeism during LMP was
84 15.0% (95%CI 12.7-17.3%), with the highest absenteeism among 15-19 year olds (17.7%,
85 95%CI 15.1-20.3%) (6). There is mixed evidence of an impact of menstrual health
86 interventions on school absenteeism. A recent systematic review identified nine relevant
87 studies in LMICs, of which six found evidence of a positive effect of an intervention on
88 absenteeism, but the studies deemed to be of highest quality tended to find no effect (7).
89 Reasons for the lack of knowledge on the extent to which menstruation affects school
90 attendance include a lack of appropriate and cross-validated absenteeism measures (8), a lack
91 of detail on whether absence was due to menstruation or non-menstrual reasons (such as
92 general illness or lack of ability to pay school fees), and the time period over which absence
93 was assessed (9).
94 A systematic review and meta-analysis of the association of menstrual pain (dysmenorrhea)
95 with absenteeism among adolescents and young women identified 38 studies (including 23
96 from LMIC), and found that 20.1% of participants reported absence from school or university
97 due to dysmenorrhea (95% CI 14.9–26.7) (10). In another systematic review of menstrual experiences
98 among university students, 74 quantitative studies globally (26 in Africa)
99 reported consequences of menstruation on academic performance, mainly due to
100 menstrual pain or heavy menstrual bleeding (11). There is relatively little, and inconclusive
101 evidence on the impact of menstruation on broader educational issues such as self-esteem,
102 participation in school and educational performance (12, 13). In the review among university
103 students, few studies provided details of how the educational outcomes were measured, and
104 none used extant data (official examination results and attendance records) to validate self-
105 reported measures (11).
106 Formative research in Ugandan secondary school students has shown strong evidence that
107 students missed school more frequently during menstruation than on other days, based on
108 prospective daily diaries (28.4% of schooldays were missed during menstruation compared
109 with 6.5% when not menstruating; p<0.001) (14). Our recent cluster-randomised trial
110 evaluating the impact of a multi-component menstrual health intervention showed strong
111 evidence of an intervention effect on multiple dimensions of menstrual health over a one-year
112 period (15), but little evidence of an impact on school absence (measured using
113 prospectively-collected daily diaries) during menstruation (11.2% vs 13.5% days missed;
114 adjusted odds ratio (aOR)=0.81, 95%CI 0.62, 1.05). There was no evidence of an impact of
115 the intervention on examination performance (adjusted mean difference (aMD)=0.06, 95%CI
116 -0.12, 0.24) or on school absence overall (10.5% days missed in both arms).
117 The aim of this paper is to quantify factors associated with menstrual-related school
118 absenteeism among Ugandan secondary school students, using data from the MENISCUS
119 trial. These findings will add to the knowledge based on the impact of multiple dimensions of
120 menstruation on the number of school days missed due to menstruation, and on examination
121 performance.

### 122 2. Methods

123 **2.1 Study Setting**
124 The study was conducted in Kalungu and Wakiso districts of Uganda. Wakiso district is the
125 largest and largely urban district in central Uganda with an estimated population of 3.4
126 million people (16) . In Wakiso, 17% of residents were aged 10-17 years, of whom 49% were
127 attending secondary schools (17). Kalungu is a rural district with population size 183,232
128 (2014 census). In Kalungu, 23% of the enrolled students were aged 10-17 years, of whom 36% were enrolled in

secondary school (18).

130

### 131 2.2 Data source

132 This study used baseline and endline survey data from the MENISCUS trial (“Menstrual
133 health interventions, schooling, and mental health problems among Ugandan students”)
134 which aimed to evaluate whether a multi-component menstrual health intervention improved
135 education, health and wellbeing among female secondary school students in Central Uganda.
136 Details of the trial design and results have been published previously (15, 19). In brief, the
137 trial was a parallel-arm, cluster-randomised controlled trial in 60 schools with schools
138 randomised 1:1 to either immediate or delayed intervention delivery.

139

### 140 2.3 Sampling of schools

141 All government schools and a random selection of private schools were contacted through
142 phone calls and assessed for eligibility using the government’s 2019 master list of Education
143 Institutions. Schools were eligible if they were mixed-sex, secondary, day- or mixed
144 day/boarding, and had at least minimum water sanitation and hygiene (WASH) facilities (an
145 improved water source and functional sex-specific sanitation facilities accessible to female
146 students), and an estimated enrolment as of January 2020 of 50-125 or 40-125 female
147 students in Secondary 1 (first year of secondary school) in Wakiso and Kalungu district,
148 respectively. We selected a random sample of 60 schools confirmed eligible and willing to
149 participate, stratified by government versus private schools and district.

### 150 2.4 Participant recruitment and informed consent/assent

151 All female students enrolled in Secondary 2 at the start of the 2022 were eligible to
152 participate in the MENISCUS trial. We sought school-level written consent from
153 headteachers and written informed consent from parents/guardians of students aged <18
154 years, and from students aged ≥18 years. Students aged <18 years were asked to assent if we
155 had received parental consent.

156

### 157 2.5 Data collection

158 Baseline data were collected from 21 March to 5 July 2022 and endline data were collected
159 from 5 June to 22 August 2023. The surveys included items on sociodemographic
160 characteristics, mental health and multiple dimensions of menstrual health including school
161 absenteeism due to menstruation (“menstrual-related absenteeism”) or due to other reasons,
162 and were self-completed using Open Data Kit software on tablets. The examinations were
163 independently administered and marked by the Uganda National Examination Board
164 (UNEB), with examinations administered on 16-17 March 2022 (baseline) and 24-26 July
165 2023 (endline). Baseline examinations comprised materials taught in Secondary 1 and
166 included four Mathematics and six Biology questions, of which one question was on puberty
167 and reproduction. Endline examinations additionally included four English Language
168 questions, and comprised materials taught in Secondary 1 and Secondary 2.

169

### 170 2.6 Study outcomes

171 Baseline menstrual-related absenteeism was assessed as self-reported number of school days
172 missed due to menstruation, excluding days where the main reason for missing school was
173 unrelated to menstruation (“Since schools reopened in January this year, how many days of
174 class did you miss during your period?” “What is the main reason for missing school during
175 your period?”). The days of school missed for any reason was obtained from the question
176 (“Since schools reopened in January this year, how many days of class did you miss for any
177 reason?”). We calculated the number of days in the denominator from the official date
178 schools re-opened post COVID-19 (10 January 2022) to the date of interview for each
179 participant, excluding school holidays. Endline menstrual-related absenteeism was assessed
180 as self-reported number of school days missed during menstruation in Terms 1 and 2 of 2023
181 until the interview date. Term 1 was from 6 February to 5 May 2023, and Term 2 started on
182 29 May 2023. Baseline and endline examination scores were calculated as the mean of
183 subject-specific z-scores at each time point.

184

### 185 2.7 Statistical analyses

186 Data were exported to Stata version 18 for final cleaning and analysis. We used a hierarchical
187 conceptual framework based on the integrated model of menstrual experience (Hennegan et
188 al 2019) to hypothesize relationships of baseline menstrual-related factors with baseline and
189 endline school absenteeism and educational performance and guide adjustment for
190 confounders. ( Figure 1: A conceptual framework show relationship of multiple dimensions
191 of menstrual health with school absenteeism and education performance). In line with the
192 conceptual framework, we defined variables as being in Level 1 (most distal - socio-
193 demographic variables), Level 2 (social support during menstruation), Level 3 (menstrual
194 knowledge, attitudes and adequate menstrual product use), Level 4 (menstrual pain
195 management, menstrual practice needs, experience of menstrual-related teasing), Level 5
196 (Self-efficacy in addressing menstrual needs scale [SAMNS](20) -menstrual care confidence
197 with menstrual preparedness and pain management sub-scales) and Level 6 (most proximal -
198 trouble concentrating in class). Menstrual-related factors are defined in Table 1.

199

200

### Table 1: Definitions of menstrual-related exposure factors used in the conceptual framework

| **Level Survey measure** | | **Brief description** | **Calculation** | **Range & interpretation** |
| --- | --- | --- | --- | --- |
| 2 | Social support for menstruation | An individual has someone who they feel okay asking for support for your period if needed (for advice, resources, emotional support) | Binary | Binary (yes/no) |
| 3 | Adequate product use | Used at least one adequate menstrual material and no inadequate materials at LMP | Binary | Binary (yes/no) |
| 3 | Knowledge of puberty & menstruation | Factual knowledge about puberty, menstruation, and the menstrual cycle | Number of 9 knowledge questions answered correctly | 0-9  (number of questions answered correctly) |
| 3 | Attitudes towards menstruation | Attitudes about what it is okay for people to do while menstruating and myths about painkillers | Number of 3 attitude questions answered positively | 0-3  (number of questions answered positively) |
| 4 | Pain at LMP | Reported having any pain at LMP. This includes reporting any pain, or a symptom of headache, backache, stomach ache or cramp at LMP. | Binary | Binary (yes/no) |
| 4 | Effective pain management strategy | Effective pain strategies defined as stretching, painkillers, eating foods with lots of water, exercising, drinking lots of clean water, holding a warm water bottle on the stomach*.* Ineffective methods defined as: doing nothing, taking antibiotics, eating spicy foods, drinking soda | Using at least one effective method and no ineffective methods, among girls reporting pain at LMP. | Binary (yes/no) |
| 4 | Menstrual Practice Needs Scale (MPNS- 36)(21) | The extent to which individuals’ menstrual management practices and environments were perceived to meet their needs during their LMP. | Weighted average of i) core items and school- specific items (75% weight) and ii) relevant material-specific items | Range 0-3. A higher score indicates fewer unmet menstrual needs (i.e. better than a lower score). |

| (25% weight). | | | | |
| --- | --- | --- | --- | --- |
| 4 | Pain relief | The amount of pain reduced | Binary | All/most or some/none |
| 4 | Experience of teasing | Whether boys or girls tease the participant about their period | Binary | Binary (yes/no) |
| 5 | Self-efficacy in addressing menstrual needs scale (SAMNS- 26) (20) | Individuals’ confidence in their capabilities to address their menstrual needs. This SAMNS scale has 3 sub-scales 1) The 17-item Menstrual Hygiene Preparation and Maintenance (MHPM) sub-scale includes items regarding self-efficacy in preparing for menstruation (e.g., tracking one’s cycle and anticipating days of bleeding), accomplishing various tasks related to maintaining menstrual hygiene (e.g., obtaining, using, cleaning, and disposing, menstrual materials in a variety of contexts), and seeking assistance when needed 2) the 4-item Executing Stigmatized Tasks (EST) sub-scale, which also included items related to obtaining and disposing menstrual materials and seeking assistance—but in situations where accomplishing the tasks involves greater risk of revealing one’s menstrual status to a male person and 3) the 5-item Menstrual Pain Management (MPM), measured confidence in mitigating menstrual pain | Mean score | 0-100, representing the mean percentage confidence in addressing needs. Higher % is greater confidence |
| 6 | Trouble participating in class | Whether the participant had trouble participating in class due to period, during their most recent LMP at school | Binary | Binary (yes/no) |

201

202 For baseline analysis, we first estimated the mean number of days missed overall per month
203 and the mean number of days reported to be missed due to menstruation. We then used
204 negative binomial regression models to estimate adjusted incidence rate ratios (aIRR) and
205 95% confidence intervals (CI) for the association of exposures with school absenteeism due
206 to menstruation, allowing for over-dispersion. We accounted for school-level clustering by
207 using random-effects models. Similarly, we used linear regression models to estimate
208 adjusted standardized mean differences (aSMD) and 95%CIs for the association of exposures
209 with examination score, using random-effects to account for school-level clustering. Initially,
210 minimally-adjusted models were fitted for each outcome, adjusting for school-level
211 clustering. We then fitted multivariable models adjusting each variable for other variables at
212 the same or at more distal levels (Figure 1), with the exception of the SAMNS sub-scales,
213 which were not adjusted for each other. We used Wald tests to assess the strength of evidence
214 and refer to a p-value<0.001 as indicating strong evidence of association (22). We interpreted
215 the results in conjunction with effect sizes and confidence intervals to provide comprehensive
216 assessment of results.

217

218 To assess temporal associations of exposures with the outcomes, we also analysed
219 associations of baseline exposures with endline outcomes, using analogous methods. We
220 restricted these analyses to participants in the control arm to avoid possible moderating
221 effects of the intervention.

### 222 3. RESULTS

223 Of the 4281 eligible participants, 3878 (90.5%) consented/assented and completed the
224 baseline survey. Of these, 3312 (79.1%) participants reported menstruating in the past 6
225 months and were included in the baseline analyses on school absenteeism. A further 367
226 (11.1%) participants were not present for the baseline UNEB exam and were excluded from
227 baseline analyses of educational performance. Students who were missing baseline education
228 performance data were more likely to have greater unmet menstrual needs (MPNS score,
229 p=0.01) but there was no evidence of association with other dimensions of menstrual health.

230

### 231 3.1 Baseline characteristics of study population

232 The mean age of participants was 15.6 years (SD=0.9), with most (n=2161; 65.2%) attending
233 a privately-owned school. Around half (n=2070; 55.3%) were day students and had their
234 mother as the primary caregiver (n=1816; 54.8%). A total of 1227 (37.0%) participants lived
235 in households of ≥8 members and 571 (17.2%) reported having one or fewer meals the
236 previous day (Table 2). Overall, 2780 (83.9%) participants reported having social support
237 during their last menstrual period (LMP) and 3038 (91.7%) reported exclusive use of
238 adequate menstrual materials at LMP (Table 3). A total of 1696 (51.2%) participants
239 answered at least 2 of the 3 attitude questions positively, and 538 (16.2%) answered at least 7
240 of the 9 menstrual knowledge questions correctly. Most participants (2446; 73.9%) reported
241 experiencing menstrual pain at last menstrual period, of whom 1529 (62.5%) reported using
242 an effective pain management strategy. The mean MPNS score was 2.11 (SD=0.52), and
243 participants have a moderate level of menstrual care confidence regarding ability to manage
244 menstrual needs (mean=60.8, SD=19.0). Overall, 251 (7.6%) and 284 (8.6%) participants
245 reported being teased by boys and girls about menstrual periods respectively, and almost half
246 of participants reported trouble participating in class during periods (n=1414, 42.7%) (Table
247 3).

248

249 Participants reported missing a mean of 1.25 school days per month overall (95%CI 1.17,
250 1.33), and 0.30 days due to menstruation (95%CI 0.27, 0.34). A total of 991 (30%)
251 participants reporting missing school due to menstruation since schools reopened, with 323
252 (9.8%) reporting missing at least one day per month due to menstruation.

253

254

### 255 Table 2. Socio-demographic characteristics associated with school absenteeism due to menstruation and examination performance at

256 **baseline**

257

| **Variable** | **Number of**  **participants** | **Days of school missed due**  **menstruation per month (n= 3312)** | | **to** | **Examination performance (n=2945)** | |
| --- | --- | --- | --- | --- | --- | --- |
|  | **Frequency**  **(%)** | **Mean day**  **(SE)** | **aIRR**  **(95%CI)^1^** | **Standardized**  **mean exam score (SE)** | | **aSMD (95%CI)^1^** |
| **Structural-level characteristics** | | | | | | |
| **District** | | | P=0.38 |  | | P=0.23 |
| Wakiso | 2600 (78.5) | 0.29 (0.02) | 1 | 0.03 (0.05) | | 0 |
| Kalungu | 712 (21.5) | 0.37 (0.03) | 1.13 (0.86, 1.50) | -0.12 (0.10) | | -0.09 (-0.23, 0.05) |
| **School-level characteristics** | | | | | | |
| **School ownership** | | | P=0.80 |  | | P=0.99 |
| Private | 2161 (65.2) | 0.27 (0.02) | 1 | 0.07 (0.06) | | 0 |
| Government | 1151 (34.8) | 0.38 (0.03) | 0.97 (0.74, 1.27) | -0.16 (0.05) | | 0.00 (-0.14, 0.14) |
| **School-mean combined UNEB Math + Biology score** | | | P=0.05 |  | | P<0.001 |
| High UNEB | 1983 (53.0) | 0.25 (0.02) | 1 | 0.24 (0.05) | | 0 |
| Low UNEB | 1758 (47.0) | 0.37 (0.02) | 1.26 (1.00, 1.60) | -0.24 (0.03) | | -0.44 (-0.57, -0.32) |
| **Proportion of students boarding at school** | | | P=0.95 |  | | P=0.84 |
| < 50% | 1820 (55.0) | 0.35 (0.02) | 1 | -0.10 (0.06) | | 0 |
| >=50% | 1492 (45.0) | 0.26 (0.02) | 0.99 (0.77, 1.28) | 0.12 (0.05) | | 0.01 (-0.12, 0.15) |
| **School size (female baseline participants per school)** | | | P=0.57 |  | | P=0.24 |
| Greater or equal to 59 | 2258 (68.2) | 0.30 (0.02) | 1 | 0.03 (0.06) | | -0.07 (-0.20, 0.05) |
| Fewer than 59 | 1054 (31.8) | 0.34 (0.02) | 0.93 (0.74, 1.18) | -0.04 (0.06) | | 0 |
| **Student type** | | | P<0.001 |  | | P=0.001 |
| Boarding | 1496 (45.2) | 0.24 (0.02) | 1 | 0.11 (0.03) | | 0 |
| Day | 1816 (54.8) | 0.37 (0.02) | 1.45 (1.20, 1.77) | -0.07 (0.05) | | -0.12 (-0.18, -0.05) |

| **Individual-level socio-demographic & economic factors** | | | | | |
| --- | --- | --- | --- | --- | --- |
| **Age group** |  |  | P-trend=0.004 |  | P-trend=0.003 |
| <15 | 283 (8.5) | 0.22 (0.03) | 1 | 0.19 (0.07) | 0.00 |
| 15 | 1353 (40.9) | 0.25 (0.02) | 1.15 (0.83, 1.60) | 0.11 (0.05) | -0.02 (-0.13, 0.08) |
| 16 | 1234 (37.3) | 0.36 (0.02) | 1.54 (1.10, 2.15) | -0.08 (0.04) | -0.17 (-0.28, -0.06) |
| 17 | 341 (10.3) | 0.38 (0.05) | 1.66 (1.11, 2.49) | -0.14 (0.06) | -0.23 (-0.36, -0.09) |
| 18+ | 101 (3.0) | 0.48 (0.12) | 1.71 (0.97, 3.03) | -0.35 (0.10) | -0.34 (-0.53, -0.15) |
| Trend |  |  | 1.20 (1.09, 1.33) |  | -0.10 (-0.13, -0.06) |
| **Age at first menstruation** |  |  | P=0.02 |  | P-trend=0.003 |
| <13 years | 758 (22.9) | 0.34 (0.03) | 1 | 0.09 (0.06) | 0.00 |
| 13 years | 1230 (37.1) | 0.24 (0.02) | 0.74 (0.59, 0.92) | 0.05 (0.05) | -0.01 (-0.08, 0.06) |
| 14 years | 1027 (31.0) | 0.34 (0.03) | 0.92 (0.73, 1.16) | -0.07 (0.05) | -0.08 (-0.16, -0.00) |
| >14 years | 297 (9.0) | 0.40 (0.05) | 1.02 (0.73, 1.42) | -0.20 (0.06) | -0.14 (-0.25, -0.03) |
| Trend |  |  | n/a |  | -0.05 (-0.08, -0.02) |
| **Religion** |  |  | P=0.002 |  | P=0.19 |
| Catholic | 1052 (31.8) | 0.28 (0.02) | 1 | -0.03 (0.05) | 0 |
| Protestant/SDA | 1285 (38.8) | 0.29 (0.02) | 1.09 (0.88, 1.35) | 0.03 (0.05) | 0.06 (-0.01, 0.13) |
| Muslim | 959 (29.0) | 0.36 (0.03) | 1.45 (1.14, 1.84) | -0.05 (0.05) | -0.01 (-0.09, 0.06) |
| None/Other | 16 (0.5) | 0.87 (0.44) | 3.78 (1.22, 11.74) | 0.03 (0.27) | 0.07 (-0.38, 0.53) |
| **Ethnicity** |  |  | P=0.54 |  | P=0.83 |
| Muganda | 2280 (68.8) | 0.32 (0.02) | 1 | 0.00 (0.05) | 0 |
| Non Muganda | 1032 (31.2) | 0.30 (0.02) | 0.94 (0.79, 1.14) | 0.02 (0.04) | 0.01 (-0.05, 0.07) |
| **Primary caregiver** |  |  | P=0.17 |  | P=0.22 |
| Mother | 1938 (58.5) | 0.33 (0.02) | 1 | 0.00 (0.05) | 0 |
| Father | 805 (24.3) | 0.27 (0.02) | 0.85 (0.69, 1.05) | 0.06 (0.05) | 0.04 (-0.02, 0.11) |
| Self and others | 569 (17.2) | 0.29 (0.03) | 0.84 (0.67, 1.06) | -0.10 (0.05) | -0.03 (-0.11, 0.05) |
| **Education of primary caregiver** |  |  | P=0.15 |  | P=0.001 |

| Secondary or more | 2215 (59.2) | 0.29 (0.02) | 1 | -0.03 (0.05) | 0 |
| --- | --- | --- | --- | --- | --- |
| Primary or less | 881 (23.6) | 0.38 (0.03) | 1.09 (0.88, 1.34) | 0.04 (0.06) | 0.12 (0.05, 0.19) |
| Don't know | 645 (17.2) | 0.28 (0.03) | 0.83 (0.66, 1.05) | 0.05 (0.05) | 0.09 (0.02, 0.17) |
| **Household size** | |  | P=0.19 |  | P=0.89 |
| 0-5 | 1001 (30.2) | 0.28 (0.02) | 1 | -0.007 (0.05) | -0.01 (-0.08, 0.05) |
| 6-7 | 1084 (32.7) | 0.31 (0.03) | 1.20 (0.97, 1.49) | -0.001 (0.05) | -0.01 (-0.08, 0.05) |
| >=8 | 1227 (37.0) | 0.34 (0.03) | 1.18 (0.95, 1.45) | 0.00 (0.04) | 0 |
| **Number of meals eaten on the previous day** | |  | P-trend=0.008 |  | P-trend=0.005 |
| Three or more | 1058 (31.9) | 0.24 (0.02) | 1 | 0.05 (0.05) | 0 |
| Two | 1683 (50.8) | 0.31 (0.02) | 1.20 (0.99, 1.46) | 0.001 (0.05) | -0.03 (-0.09, 0.04) |
| One or fewer | 571 (17.2) | 0.41 (0.04) | 1.53 (1.19, 1.97) | -0.08 (0.05) | -0.13 (-0.21, -0.04) |
| Trend | |  | 1.24 (1.09, 1.40) |  | -0.06 (-0.10, -0.02) |
| **Socioeconomic status quintile** | |  | P=0.02 |  | P=0.04 |
| Highest | 656 (19.8) | 0.24 (0.03) | 1 | 0.005 (0.06) | 0 |
| Medium-high | 684 (20.7) | 0.25 (0.03) | 0.91 (0.69, 1.19) | 0.14 (0.04) | 0.12 (0.04, 0.21) |
| Medium | 655 (19.8) | 0.29 (0.03) | 0.99 (0.75, 1.30) | 0.009 (0.06) | 0.04 (-0.05, 0.13) |
| Low-medium | 676 (20.4) | 0.30 (0.03) | 0.92 (0.69, 1.22) | -0.02 (0.05) | 0.03 (-0.06, 0.13) |
| Low | 641 (19.4) | 0.47 (0.04) | 1.35 (1.01, 1.81) | -0.06 (0.05) | 0.00 (-0.09, 0.10) |
| ^1^ Adjusted for all socio-demographic factors and school level clustering | | | | | |

258

259

260

261

### Table 3. Menstrual characteristics associated with school absenteeism due to menstruation and examination performance at baseline

| **Variable** | **Number**  **participants** | **of** | **Days of school missed due to**  **menstruation per month (n= 3312)** | | **Examination performance (n=2945)** | |
| --- | --- | --- | --- | --- | --- | --- |
|  | **Frequency (%)** | **Mean day (SE)** | | **aIRR (95%CI)^1^** | **Standardized**  **mean exam score (SE)** | **aSMD (95%CI)^1^** |
| **Level 2 – Menstrual support** | | | | | | |
| **Social support for menstruation** |  |  | | P=0.77 |  | P=0.28 |
| Yes | 2780 (83.9) | 0.30 (0.01) | | 1 | -0.007 (0.04) | 0 |
| No | 532 (16.1) | 0.35 (0.04) | | 1.03 (0.82, 1.31) | 0.03 (0.06) | 0.04 (-0.03, 0.12) |
| **Level 3 – Menstrual knowledge, attitudes and adequate product use** | | | | | | |
| **Adequate product use** |  |  | | P<0.001 |  | P=0.008 |
| Yes | 3038 (91.7) | 0.29 (0.02) | | 1 | 0.009 (0.04) | 0 |
| No | 274 (8.3) | 0.54 (0.07) | | 1.82 (1.37, 2.42) | -0.14 (0.06) | -0.13 (-0.23, -0.03) |
| **Attitudes towards menstruation** |  |  | | P<0.001 |  | P<0.001 |
| Positive (2-3) | 1696 (51.2) | 0.25 (0.02) | | 1 | 0.10 (0.05) | 0 |
| Negative (0-1) | 1616 (48.8) | 0.37 (0.02) | | 1.42 (1.21, 1.68) | -0.10 (0.05) | -0.15 (-0.21, -0.10) |
| **Knowledge of puberty and menstruation** |  |  | | P-trend=0.58 |  | P-trend<0.001 |
| High (7-9) | 538 (16.2) | 0.27 (0.04) | | 1 | 0.34 (0.07) | 0 |
| Medium (4-6) | 2378 (71.8) | 0.31 (0.02) | | 1.05 (0.83, 1.33) | -0.03 (0.04) | -0.32 (-0.40, -0.25) |
| Low (0-3) | 396 (12.0) | 0.39 (0.05) | | 1.09 (0.79, 1.51) | -0.30 (0.05) | -0.55 (-0.66, -0.45) |
| Trend |  |  | | 1.06 (0.86, 1.30) |  | -0.28 (-0.33, -0.23) |
| **Level 4 – Menstrual pain, pain management and experience of teasing** | | | | | | |
| **Menstrual pain at LMP** |  |  | | p<0.001 |  | P=0.04 |
| No | 866 (26.1) | 0.15 (0.02) | | 1 | 0.02 (0.05) | 0 |
| Yes | 2446 (73.9) | 0.37 (0.02) | | 2.12 (1.73-2.59) | 0.001 (0.05) | 0.06 (0.00, 0.13) |

| **Effective pain management strategy at LMP** | |  | P<0.001 |  | P=0.09 |
| --- | --- | --- | --- | --- | --- |
| No pain | 866 (26.1) | 0.15 (0.02) | 1 | 0.02 (0.05) | 0 |
| Pain and used at least one effective  management strategy | 1529 (46.2) | 0.35 (0.02) | 2.13 (1.72, 2.63) | -0.02 (0.04) | 0.08 (0.01, 0.14) |
| Pain and did not use at least one effective  strategy | 917 (27.7) | 0.39 (0.03) | 2.10 (1.66, 2.66) | -0.06 (0.04) | 0.05 (-0.03, 0.12) |
| **Menstrual pain relief** | |  | P<0.001 |  | P=0.13 |
| No pain | 866 (26.1) | 0.15 (0.02) | 1 | 0.02 (0.05) | 0 |
| All/most pain relieved | 983 (29.7) | 0.34 (0.03) | 2.00 (1.59, 2.51) | 0.01 (0.05) | 0.06 (-0.01, 0.13) |
| Some/none of pain relieved | 1463 (44.2) | 0.38 (0.02) | 2.20 (1.78, 2.73) | -0.005 (0.04) | 0.07 (-0.00, 0.14) |
| **Menstrual Practice Needs Score (MPNS score)** | |  | P-trend<0.001 |  | P-trend<0.001 |
| High - few unmet needs (2.38-3.00] | 1116 (34.0) | 0.18 (0.02) | 1 | 0.12 (0.05) | 0 |
| Medium (1.88-2.38] | 1091 (33.2) | 0.27 (0.02) | 1.41 (1.15, 1.73) | -0.03 (0.04) | -0.10 (-0.17, -0.04) |
| Low - many unmet needs (0.00-1.88] | 1076 (32.8) | 0.48 (0.03) | 2.02 (1.63, 2.50) | -0.09 (0.05) | -0.14 (-0.21, -0.07) |
| Trend | |  | 1.44 (1.29-1.57) |  | -0.07 (-0.11, -0.03) |
| **Experience of teasing by boys** | |  | P=0.04 |  | P=0.18 |
| No | 3061 (92.4) | 0.29 (0.01) | 1 | 0.01 (0.04) | 0 |
| Yes | 251 (7.6) | 0.60 (0.08) | 1.38 (1.02, 1.88) | -0.20 (0.05) | -0.07 (-0.18, 0.03) |
| **Experience of teasing by girls** | |  | P=0.07 |  | p<0.001 |
| No | 3028 (91.4) | 0.29 (0.01) | 1 | 0.02 (0.04) | 0 |
| Yes | 284 (8.6) | 0.53 (0.07) | 1.31 (0.98,1.75) | -0.30 (0.06) | -0.17 (-0.27, -0.07) |
| **Level 5 – Menstrual care confidence** | | | | | |
| **Menstrual care confidence (SAMNS score)** | |  | P=0.01 |  | P=0.73 |
| High self-efficacy (69.62-100] | 1129 (34.1) | 0.25 (0.02) | 1 | 0.05 (0.05) | 0 |
| Medium (52.31-69.62] | 1102 (33.3) | 0.25 (0.02) | 0.88 (0.72, 1.08) | -0.02 (0.05) | -0.01 (-0.07, 0.06) |
| Low self-efficacy (0-52.31] | 1081 (32.6) | 0.43 (0.03) | 1.20 (0.98, 1.49) | -0.03 (0.05) | 0.02 (-0.05, 0.09) |
| **Menstrual preparedness sub-scale** | |  | P-trend<0.001 |  | P=0.86 |

| High (76.47-100] | 1058 (31.9) | 0.21 (0.02) | 1 | 0.07 (0.05) | 0 |
| --- | --- | --- | --- | --- | --- |
| Medium (58.25-76.47]- | 1127 (34.0) | 0.26 (0.02) | 1.15 (0.93, 1.41) | 0.002 (0.05) | 0.01(-0.05, 0.08) |
| Low (0-58.25] | 1127 (34.0) | 0.45 (0.03) | 1.54 (1.24, 1.91) | -0.06 (0.05) | 0.00 (-0.08, 0.07) |
| Trend |  |  | 1.25 (1.12, 1.39) |  | n/a |
| **Pain management sub-scale** |  |  | P=0.49 |  | P=0.71 |
| High (82-100] | 923 (27.9) | 0.28 (0.03) | 1 | -0.001 (0.05) | 0 |
| Medium (50-82] | 1241 (37.5) | 0.28 (0.02) | 0.98 (0.80, 1.21) | 0.02 (0.05) | 0.02 (-0.04, 0.09) |
| Low (0-50] | 1148 (34.7) | 0.37 (0.03) | 1.10 (0.89, 1.36) | -0.02 (0.05) | 0.03 (-0.04, 0.10) |
| **Level 6 – Class participation and attendance at LMP** | | | | | |
| **Trouble participating in class during LMP** |  |  | P<0.001 |  | P=0.91 |
| No | 1898 (57.3%) | 0.20 (0.01) | 1 | 0.007 (0.04) | 0 |
| Yes | 1414 (42.7%) | 0.46 (0.03) | 1.80 (1.51, 2.14) | -0.01 (0.05) | 0.00 (-0.06, 0.06) |
| **School absenteeism due to menstruation** |  |  |  |  | P=0.01 |
| Missed less than one day per month | n/a | n/a | n/a | 0.14 (0.04) | 0 |
| Missed at least one day per month | n/a | n/a | n/a | -0.20 (0.05) | -0.12 (-0.22, -0.03) |
| ^1^ Adjusted for school level clustering, level 1 (socio-demographic) factors and variables at the same or more distal levels. SAMNS scores and  sub-scales are not adjusted for each other. Trouble participating, and school absenteeism are not adjusted for each other. | | | | | |

262

263

264

### 265 3.2 Socio-demographic factors associated with menstrual-related absenteeism and

266 **examination performance at baseline**
267 Menstrual-related absenteeism and poorer examination performance were both associated
268 with being in a school with a poorer examination performance (aIRR=1.26, 95%CI 1.00,
269 1.60; aMD=-0.44, 95%CI -0.57, -0.32), being a day student (aIRR=1.45, 95%CI 1.20, 1.77;
270 aMD=-0.12, 95%CI -0.18, -0.05), older age (aIRR-trend=1.20, 95%CI 1.09, 1.33; aMD-
271 trend=-0.10, 95%CI -0.13, -0.06) and having fewer meals the previous day (aIRR-trend=1.24,
272 95%CI 1.09, 1.40; aMD-trend=-0.06, 95%CI -0.10, -0.02) (Table 2). In addition, poorer
273 examination performance was associated with older age at first menses (aMD-trend=-0.05,
274 95%CI -0.08, -0.02) and higher education of the primary caregiver (aMD=-0.12, -0.19, -0.05)
275 (Table 2).

276

### 277 3.3 Menstrual factors associated with school absenteeism due to menstruation and

278 **poorer examination performance at baseline**
279 Among the 991 participants reporting missing school due to menstruation, 1023 days were
280 missed per month (mean per participant =1.03 per month (SE=0.04) (Table 4). The most
281 commonly reported main reason was illness (back/stomach pain or cramps: n=588; 57.2% of
282 days missed; feeling generally unwell: n=170; 17.2% of days missed). Relatively few
283 participants reported other reasons (stigma-related, the physical environment or socio-
284 economic) as the main reason for missing school during menstruation (Table 4).

285

286

287

### Table 4: Main reason for missing school due to menstruation, reported at baseline

| **Main reason for missing school due to menstruation** | **Number of participants n (%)** | **Mean (SD) days per participant missed due to menstruation per month among those reporting**  **this reason** | **Total days missed due to menstruation per month with this as the main reason** | **Proportion of days missed due to menstruation per month with this as**  **the mains reason** |
| --- | --- | --- | --- | --- |
| **Total** | **991** | **1.03 (1.16)** | **1023** | **100%** |
| **Illness** | | | | |
| Back/stomach pain or cramps | 588 (59.3) | 1.00 (1.06) | 585 | 57.2% |
| Feeling generally unwell | 170 (17.2) | 0.98 (1.20) | 165 | 16.2% |
| **Stigma** | | | | |
| Scared of leaking blood on clothes | 59 (6.0) | 0.92 (0.95) | 54 | 5.3% |
| Not wanting to change pad or absorbents at school | 2 (0.2) | 2.49 (3.01) | 5 | 0.5% |
| Household members telling me not to go to school  during period | 12 (1.2) | 1.86 (2.38) | 22 | 2.2% |
| Worried that others may know I have my periods | 18 (1.8) | 0.79 (0.53) | 14 | 1.4% |
| Scared of having to ask for help because of  periods | 6 (0.6) | 0.94 (0.72) | 6 | 0.5% |
| **Physical environment** | | | | |
| School toilets or latrines not clean | 34 (3.4) | 1.20 (1.28) | 41 | 4.0% |
| Lack of access to toilet paper at school | 4 (0.4) | 0.56 (0.24) | 2 | 0.2% |
| Lack of access to water and/or soap at school | 17 (1.7) | 1.34 (2.27) | 23 | 2.2% |
| Not enough privacy in school toilet/latrine | 34 (1.4) | 1.20 (1.28) | 41 | 4.0% |
| **Socio-economic** | | | | |

| Unable to afford pads or other absorbents | 35 | 1.50 (1.45) | 52 | 5.1% |
| --- | --- | --- | --- | --- |
| Lack of knickers | 31 | 1.26 (1.35) | 39 | 4.8% |

288

289

290

291

292 After adjusting for factors at the same or more distal levels, there was strong evidence that
293 menstrual-related absenteeism and poorer examination performance were both associated
294 with not exclusively using adequate menstrual materials (aIRR=1.82, 95%CI 1.37, 2.42;
295 aMD=-0.13, 95%CI -0.23, -0.03), negative menstrual attitudes (aIRR=1.42, 95%CI 1.21,
296 1.68, aMD=-0.15, 95%CI -0.21, -0.10), menstrual pain at LMP (aIRR=2.12, 95%CI 1.73,
297 2.59; aMD=0.06, 95%CI 0.00, 0.13), more unmet menstrual practice needs (p-trend<0.001)
298 and reported experience of menstrual-related teasing at LMP by girls (aIRR=1.31, 95%CI
299 0.98, 1.75; aMD=-0.17, 95%CI -0.27, -0.07) (Table 3). In absolute terms, the difference was
300 relatively small, for example, the mean days missed per month among participants with no
301 menstrual pain at LMP was 0.15 (SE 0.02), compared with 0.37 (SE 0.02) among participants
302 with pain at LMP.

303

304 After adjustment, menstrual-related absenteeism was associated with lack of perceived pain
305 relief at LMP (aIRR=2.20, 95%CI 1.78, 2.73 for minimal/no pain relieved vs no pain),
306 menstrual teasing by boys (aIRR=1.38, 95%CI 1.02, 1.88), poor menstrual care confidence
307 (aIRR=1.20, 95%CI 0.98, 1.49 for lowest vs highest quartile, p-trend=0.01), especially due to
308 lack of menstrual preparedness (aIRR=1.54, 95%CI 1.24, 1.91, p-trend<0.001) and with
309 trouble participating in class during LMP (aIRR=1.80, 95%CI 1.51, 2.14). Similar factors
310 were associated with missing school overall (i.e. for any reason) (Supplementary Table 1).

311

### 312 3.4 Menstrual factors at baseline associated with menstrual-related absenteeism and

313 **poorer examination performance at endline.**
314 Among the 1652 participants in control arm schools at baseline, 1253 (75.8%) were in control
315 schools at endline and completed the endline survey. Of these, 1192 (95.1%) reported having
316 menstruated in the past 6 months and were included in endline analysis, and 1099 (92.2%)
317 had examination data performance at endline.

318

319 Among	these	participants,	menstrual-related	absenteeism	and	poorer	examination
320 performance at endline were both associated with using inadequate menstrual products at
321 baseline (aIRR=1.76, 95%CI 0.95, 3.24; aMD=-0.19, 95%CI -0.34, -0.03), negative
322 menstrual attitudes (aIRR=1.76, 95%CI 1.26, 2.46; aMD=-0.16, 95%CI -0.24, -0.08), more
323 unmet menstrual practice needs (aIRR=1.75, 95%CI 1.12-2.71; aSMD= -0.10, 95%CI -0.21
324 to 0.00) and experience of teasing about menstruation by girls (aIRR=1.89, 95%CI 1.06,
325 2.73; aMD=-0.33, 95%CI -0.48, -0.18) (Table 5).

326

327 In addition, menstrual-related absenteeism was associated with baseline menstrual pain
328 (aIRR=3.08, 95%CI 2.04, 4.65), including if there was pain management but no pain relief
329 (aIRR=3.42, 95%CI 2.18, 5.36 vs no pain), and trouble participating in class during LMP
330 (aIRR=1.69, 95%CI 1.20, 2.38). Endline examination performance was additionally
331 associated with poorer baseline menstrual knowledge (aMD=-0.44, 95%CI -0.60, -0.28)
332 (Table 5).

333

### 334 Table 5. Baseline menstrual characteristics associated with endline school absenteeism due to menstruation and examination

335 **performance at baseline**

336

**Variable	Number	of**

**Days	of	school	missed	due	to**

**Examination performance at endline**

| **participants menstruation at endline (n= 1192) (n=1099)** | | | | | |
| --- | --- | --- | --- | --- | --- |
|  | **Frequency (%)** | **Mean day (SE)** | **aIRR (95%CI)^1^** | **Standardized mean exam score**  **(SE)** | **aSMD (95%CI)^1^** |
| **Level 2 – Menstrual support** |  |  |  |  |  |
| **Social support for menstruation** |  |  | P=0.18 |  | P=0.15 |
| Yes | 1010 (84.7) | 0.32 (0.03) | 1 | -0.05 (0.08) | 0 |
| No | 182 (15.3) | 0.25 (0.04) | 0.73 (0.45, 1.16) | -0.06 (0.09) | -0.09 (-0.03, 0.20) |
| **Level 3 – Menstrual knowledge, attitudes and adequate product use** | | | | | |
| **Adequate product use** |  |  | P=0.07 |  | P=0.01 |
| Yes | 1106 (92.8) | 0.30 (0.02) | 1 | -0.08 (0.08) | 0 |
| No | 86 (7.2) | 0.48 (0.12) | 1.76 (0.95, 3.24) | -0.23 (0.11) | -0.19 (-0.34, -0.03) |
| **Attitudes towards menstruation** |  |  | P<0.001 |  | P<0.001 |
| Positive (2-3) | 614 (51.5) | 0.23 (0.03) | 1 | 0.01 (0.08) | 0 |
| Negative (0-1) | 578 (48.5) | 0.40 (0.04) | 1.76 (1.26, 2.46) | -0.18 (0.08) | -0.16 (-0.24, -0.08) |
| **Knowledge of puberty and menstruation** P=0.72 P-trend<0.001 | | | | | |
| High (7-9) | 198 (16.6) | 0.26 (0.05) | 1 | 0.26 (0.09) | 0 |
| Medium (4-6) | 839 (70.4) | 0.32 (0.03) | 1.13 (0.72, 1.79) | -0.06 (0.07) | -0.22 (-0.33, -0.10) |
| Low (0-3) | 155 (13.0) | 0.36 (0.08) | 0.96 (0.50, 1.83) | -0.29 (0.09) | -0.44 (-0.60, -0.28) |
| Trend |  |  |  |  | -0.22 (-0.30, -0.14) |
| **Level 4 – menstrual pain, pain management and experience of teasing** | | | | | |

**Menstrual pain at LMP**	P<0.001	P=0.68

| No pain | 305 (25.6) | 0.13 (0.04) | 1 | 0.03 (0.09) | 0 |
| --- | --- | --- | --- | --- | --- |
| Any pain | 887 (74.4) | 0.37 (0.03) | 3.08 (2.04, 4.65) | -0.07 (00.22, 0.09) | -0.01 (-0.11, 0.08) |
| **Effective pain management strategy** |  |  | P<0.001 |  | P=0.93 |
| No pain | 305 (25.6) | 0.13 (0.04) | 1 | 0.03 (0.09) | 0 |
| Pain and used at least one effective  management strategy | 538 (45.1) | 0.39 (0.04) | 3.07 (1.98, 4.76) | -0.06 (0.09) | -0.01 (-0.11, 0.10) |
| Pain and did not use at least one  effective strategy | 349 (29.3) | 0.36 (0.05) | 3.09 (0.95, 2.15) | -0.07 (0.08) | -0.02 (-0.13, 0.09) |
| **Menstrual pain relief** |  |  | P<0.001 |  | P=0.64 |
| No pain | 305 (25.6) | 0.13 (0.04) | 1 | 0.03 (0.09) | 0 |
| All/most pain relieved | 371 (31.1) | 0.35 (0.05) | 2.72 (1.72, 4.29) | -0.11 (0.10) | -0.04 (-0.15, 0.07) |
| Some/none of pain relieved | 516 (43.3) | 0.39 (0.03) | 3.42 (2.18, 5.36) | -0.04 (0.08) | 0.01 (-0.10, 0.11) |
| **Menstrual Practice Needs Score (MPNS score)** | |  | P=0.005 |  | P=0.06 |
| High - few unmet needs (2.38-3.00] 445 (37.6) | | 0.20 (0.03) | 1 | 0.05 (0.08) | 0 |
| Medium (1.88-2.38] 372 (31.4) | | 0.35 (0.05) | 1.43 (0.95, 2.15) | -0.07 (0.09) | -0.07 (-0.17, 0.04) |
| Low - many unmet needs (0.00-1.88] 366 (30.9) | | 0.42 (0.04) | 1.75 (1.12, 2.71) | -0.13 (0.08) | -0.10 (-0.21, 0.00) |
| **Experience of teasing by boys** | |  | P=0.18 |  | P=0.25 |
| No 1098 (92.1) | | 0.29 (0.02) | 1 | -0.04 (0.08) | 0 |
| Yes 94 (7.9) | | 0.59 (0.12) | 1.50 (0.83, 2.73) | -0.16 (0.13) | 0.07 (-0.09, 0.22) |
| **Experience of teasing by girls** | |  | P=0.03 |  | P<0.001 |
| No 1087 (91.2) | | 0.29 (0.02) | 1 | -0.02 (0.08) | 0 |
| Yes 105 (8.8) | | 0.56 (0.09) | 1.89 (1.06, 3.37) | -0.35 (0.12) | -0.33 (-0.48, -0.18) |
| **Level 5 – Menstrual care confidence** | |  |  |  |  |
| **Menstrual care confidence (SAMNS score)** | |  | p-trend=0.40 |  | P=0.55 |
| High self-efficacy (69.62-100) 420 (35.2) | | 0.28 (0.04) | 1 | 0.02 (0.09) | 0 |
| Medium (52.31-69.62) 397 (33.3) | | 0.30 (0.04) | 1.08 (0.71, 1.62) | -0.02 (0.09) | 0.02 (-0.08, 0.12) |
| Low (0-52.31) 375 (31.5) | | 0.36 (0.04) | 1.20 (0.78, 1.83) | -0.13 (0.08) | -0.04 (-0.15, 0.07) |

| Trend | | | | | 0.91 (0.74, 1.13) | |  | |
| --- | --- | --- | --- | --- | --- | --- | --- | --- |
| **Level 6 – Class participation and attendance at LMP** | | | | |  | |  | |
| **Trouble participating in class during LMP** | | | | | P<0.001 | | P=0.52 | |
| No |  |  |  | 678 (56.9) | 0.20 (0.02) | 1 | -0.05 (0.08) | 0 |
| Yes |  |  |  | 514 (43.1) | 0.47 (0.04) | 1.69 (1.20-2.38) | -0.06 (0.08) | 0.03 (-0.06, 0.12) |
| **School** | **absenteeism** | **due** | **to** |  |  |  |  |  |
| **menstruation** | | | n/a | | n/a | n/a |  | P=0.43 |
| Missed less than one day per month | | | n/a | | n/a | n/a | -0.04 (0.08) | 0 |
| Missed at least one day per month | | | n/a | | n/a | n/a | -0.20 (0.10) | -0.06 (-0.22, 0.09) |

^1^ Adjusted for school level clustering and baseline variables at Level 1 (socio-demographic) and at the same or more distal levels.

337

338

339

340

341

342

343 Similar factors were associated with school absenteeism for any reason (including
344 menstruation) (Supplementary Table 1). For example, the mean number of days missed due
345 to menstrual pain was 1.36 days per month, vs 0.88 among those without pain at LMP
346 (aIRR=1.41, 95%CI 1.26-1.57).

347

348 **4. DISCUSSION**
349 **4.1 Multiple dimensions of menstrual health are associated with school absenteeism and**
350 **poor examination performance**
351 In this setting, there is strong evidence that multiple dimensions of menstrual health were
352 associated with both increased menstrual-related absenteeism and poorer examination
353 performance. These included use of inadequate products, negative attitudes towards
354 menstruation, having many unmet menstrual needs, menstrual pain and experience of teasing.
355 These add to the limited evidence quantifying both the amount of menstrual-related
356 absenteeism, and the specific menstrual-related factors associated with absenteeism and
357 examination performance.

358

### 359 4.2 The impact of menstrual health on number of days of school missed due to

360 **menstruation**
361 Our findings that 10% of participants reported missing at least one school day per month due
362 to menstruation supports the widespread claim that one in 10 girls miss school due to
363 menstruation (3). However, on average, participants reported missing less than half a day of
364 school per month due to menstruation (0.30 days). The prevalence of menstrual-related
365 absenteeism is at the lower end of the range seen in the systematic review of studies in Sub-
366 Saharan Africa (6-65% missing school during menstruation) (5), and slightly lower than the
367 recent multi-country study (17.7%, 95%CI 15.1-20.3%) among 15-19 year olds (6). Reported
368 school absence may be lower in our study because i) students had missed almost two years of
369 school due to COVID-19 closures and may have felt pressure from teachers and parents to
370 make up the time; ii) reported days of school missed due to menstruation may be under-
371 reported by boarding students who interpreted being on school premises as attending school,
372 and iii) the relatively long period of reporting in our study may have led to underestimation
373 due to recall bias. The variation in estimates between studies is also likely due to challenges
374 measuring school attendance accurately (8). Measuring school attendance using self-report
375 often underestimates absenteeism compared to using school registers (23), which may be due
376 to social desirability bias (8, 24), stigma and taboos around menstruation, and recall bias.
377 This is supported by qualitative findings from a Kenyan study, in which girls rarely reported
378 school absenteeism themselves, but reported that ‘other’ girls did (25). However in some
379 settings, including Uganda, school registers are incomplete and/or inaccurate, due to teachers
380 not completing the registers, and social desirability bias if school funding is linked with
381 student numbers (23). Future studies should consider a combination of these methods, daily
382 diaries and spot checks to triangulate data on school attendance (23).

383

384 In our study, students reporting menstrual pain had more than double the rate of menstrual-
385 related absenteeism than those with no pain, and school absenteeism was highest among
386 those with inadequate pain relief. Further, we found that menstrual pain or feeling generally
387 unwell were responsible for about three-quarters of days of school absenteeism due to
388 menstruation. To our knowledge, this is the first study to quantify days of school absenteeism
389 attributable to specific menstrual-related factors.
390 At baseline, there was evidence that participants with low menstrual self-efficacy were more
391 likely to miss school due menstruation compared to those with high menstrual self-efficacy.
392 The association was strongest with the sub-scale of lack of menstrual preparedness (e.g.,
393 tracking one’s cycle, obtaining, using, cleaning, and disposing of menstrual products, and
394 seeking assistance when needed). This fits with findings from qualitative studies that students
395 with low menstrual confidence might feel uncomfortable to be around students or teachers
396 during menstruation and hence choose to miss school during menstruation (9, 26). This
397 association may also be due to girls fearing leakage of blood whilst in class and hence
398 missing school (2).

### 399 4.3 The impact of menstrual health on educational performance

400 Qualitative studies have described how participants perceive that menstruation affects their
401 academic performance (2) but there are few rigorous quantitative studies on this. Our findings
402 support the hypothesis that menstruation affects academic performance through multiple
403 pathways, through menstrual pain or use of inadequate materials, but also due to social
404 stigma and lack of menstrual knowledge. Using a standardised assessment set by the UNEB,
405 we found that poorer examination performance was associated with multiple dimensions of
406 menstrual health. There was strong evidence of associations between poorer examination
407 scores and use of inadequate materials, poorer menstrual attitudes, poorer menstrual
408 knowledge, having more unmet menstrual practice needs and experience of being teased in
409 relation to periods.

410

411 Our results support qualitative findings from the systematic review (2) and a qualitative study
412 of 120 girls aged 14-16 years in in Kenya where girls reported that their attention in class was
413 disturbed by worries of leakage of menstrual blood and staining of clothing (27). A further
414 study found that students reported failing to stand up and answer questions, write on the
415 board and miss exams during menstruation (12). Missing at least one day of school due to
416 menstruation at LMP at baseline was associated with poorer baseline examination scores, but
417 not with endline examination scores. This difference may be due to chance or due to the
418 longer temporal gap between missing school at baseline and examination performance at
419 endline. Persistent school absenteeism increases the risk of school dropout, lowers girls' self-
420 esteem, and compromises future productivity (28). A strength of our study was the use of an
421 independent examination set by UNEB. We identified one previous study in Uganda which
422 evaluated academic performance similarly, using assessments in English language,
423 Mathematics, Integrated Science, and Social Studies set by UNEB (29) . In this small
424 randomised controlled trial of 60 participants in two primary schools in Uganda, a menstrual
425 health intervention (which included telling menstrual stories and playing games related to
426 menstrual management) was associated with improved academic scores at 6 weeks after the
427 intervention (29)

428

### 429 4.4 Strengths and limitations of the study

430 Strengths of our study include the measurement of multiple dimensions of menstrual health;
431 use of both cross-sectional and longitudinal analyses (to minimise reverse causality);
432 measurement of reported number of schooldays missed due to menstruation over a defined
433 period; use of a self-completed questionnaire (to minimise social desirability bias); use of
434 validated tools for menstrual care confidence and unmet practice needs; and assessment of
435 educational performance using an examination set independently by UNEB, tailored to
436 material that had been taught across all 60 schools.

437

438 Limitations include possible recall bias from retrospective, self-reported outcome of school
439 attendance, residual confounding, and challenges related to the long duration (one year)
440 between baseline menstrual experiences and endline outcomes for the longitudinal analyses.
441 The educational performance outcome is objective but limited, especially at baseline, by the
442 relatively sparse amount of material that had been taught between schools re-opening after
443 COVID-9 in January 2022, and the assessment in March 2022. This resulted in a lack of
444 discrimination between potentially low- and high-achieving students.

445

### 446 5.5 Conclusion and Recommendation

447 Multiple dimensions of menstrual health are strongly associated with school absenteeism and
448 subsequent poor education performance among female students in Ugandan secondary
449 schools, but the absolute effect size is small as menstrual-related absenteeism is infrequent.
450 Our previous finding that there was no evidence of an effect of a multi-component menstrual
451 health intervention on school absenteeism and educational performance in this population,
452 despite modest improvements in multiple dimensions of menstrual health (15), suggests that
453 larger improvements in menstrual health are needed to impact on educational outcomes.
454 However, improving menstrual health remains a critical priority globally. Addressing this
455 issue is essential not only for safeguarding physical and psychological well-being but also
456 advancing gender equality and human rights issue.

457

### 458 Acknowledgements

459	We would like to thank the staff, students, and communities of participating schools for their

460	engagement with the study; the stakeholders from the Uganda Ministry of Education and

461	Sports; and District Education Officers in Wakiso and Kalungu districts.

462

463

464	**Abbreviations**

465

466 MH: Menstrual health; LMP: Last menstrual period; CI: Confidence interval; LRT:
467 Likelihood ratio test; SMD: Standardized mean difference; IRR: Incidence rate; MENISCUS:
468 Menstrual health interventions, schooling, and mental health problems among Ugandan
469 students; MHM: Menstrual hygiene management; SES: Socio-economic status; WASH:
470 Water, sanitation and hygiene; SAMNS: Self-efficacy in Addressing Menstrual Needs Scale;
471 MHPM: Menstrual Hygiene Preparation and Maintenance sub-scale; MPM: Menstrual Pain
472 Management sub-scale; EST: Executing Stigmatized Tasks sub-scale

473

### 474 Funding

475 This work was supported by the Joint Global Health Scheme with funding from the UK
476 Foreign, Commonwealth and Development Office, the UK Medical Research Council, the
477 UK Department of Health and Social Care through the National Institute of Health Research
478 (NIHR), and Wellcome (Grant Ref. MR/ V005634/1). The funder had no role in the design of
479 the study; in the collection, analyses, or interpretation of the data; in the writing of the article;
480 or in the decision to publish the results.

481

### 482 Declarations

483 **Ethical approval**
484 Ethical approval for MENISCUS protocol, the informed consent forms and draft CRFs were
485 obtained from the Uganda Virus Research Institute Research & Ethics Committee (UVRI-
486 REC) (reference GC/127/819) and the Uganda National Council of Science and Technology
487 (UNCST) (reference HS1525ES) and the LSHTM Research Ethics Committee (reference
488 22952-2).

### 489 Consent for publication

490 Participants consented to the research findings being published in international science
491 journals and electronic websites, on condition that that findings cannot be traced to
492 individuals.

### 493 Availability of data and materials

494 The datasets used and/or analysed during the current study are available from the
495 corresponding author on reasonable request.

### 496 Competing interests

497 The authors declare that they have no competing interests.

### 498 References

499 1.	Hennegan J, Winkler IT, Bobel C, Keiser D, Hampton J, Larsson G, et al. Menstrual
500 health: a definition for policy, practice, and research. Sex Reprod Health Matters.
501 2021a;29(1):1911618.
502 2.	Hennegan J, Shannon AK, Rubli J, Schwab KJ, Melendez-Torres GJ. Women’s and
503 girls’ experiences of menstruation in low- and middle-income countries: A systematic review
504 and qualitative metasynthesis. PLOS Medicine. 2019;16(5):e1002803.
505 3.	Wilkinson K. No data shows 1 in 10 African girls miss school because of
506 menstruation South Africa: Africa Check; 2016 [Available from: [https://africacheck.org/fact-](https://africacheck.org/fact-checks/reports/no-data-shows-1-10-african-girls-miss-school-because-menstruation)
507 [checks/reports/no-data-shows-1-10-african-girls-miss-school-because-menstruation](https://africacheck.org/fact-checks/reports/no-data-shows-1-10-african-girls-miss-school-because-menstruation).
508 4.	Bobel C. The Managed Body: Developing Girls and Menstrual Health in the Global
509 South. Cham, Switzerland.: Palgrave Macmillan / Springer Nature Switzerland AG; 2018.
510 5.	Tadesse AG. Prevalence of school absenteeism during menstruation and associated
511 factors in sub-Saharan Africa: A systematic review and meta-analysis. Research Square.
512 2023.
513 6.	Starr M, Harding R, Ataíde R, Von Dinklage N, Sinharoy SS, Jayasinghe Y, et al.
514 Epidemiology of menstrual-related absenteeism in 44 low-income and middle-income
515 countries: a cross-sectional analysis of Multiple Indicator Cluster Surveys. The Lancet Global
516 Health. 2025;13(2):e285-e97.
517 7.	Betsu BD, Medhanyie AA, Gebrehiwet TG, Wall LL. Menstrual hygiene management
518 interventions and their effects on schoolgirls' menstrual hygiene experiences in low and
519 middle countries: A systematic review. PLoS One. 2024;19(8):e0302523.
520 8.	Benshaul-Tolonen A, Zulaika G, Sommer M, Phillips-Howard PA. Measuring
521 Menstruation-Related Absenteeism Among Adolescents in Low-Income Countries. In: Bobel
522 C, Winkler IT, Fahs B, Hasson KA, Kissling EA, Roberts T-A, editors. The Palgrave
523 Handbook of Critical Menstruation Studies. Singapore: Springer Singapore; 2020. p. 705-23.
524 9.	Hennegan J, OlaOlorun FM, Oumarou S, Alzouma S, Guiella G, Omoluabi E, et al.
525 School and work absenteeism due to menstruation in three West African countries: findings
526 from PMA2020 surveys. Sex Reprod Health Matters. 2021b;29(1):1915940.
527 10.	Armour M, Parry K, Manohar N, Holmes K, Ferfolja T, Curry C, et al. The
528 Prevalence and Academic Impact of Dysmenorrhea in 21,573 Young Women: A Systematic
529 Review and Meta-Analysis. Journal of Women's Health. 2019;28(8):1161-71.
530 11.	Munro AK, Hunter EC, Hossain SZ, Keep M. A systematic review of the menstrual
531 experiences of university students and the impacts on their education: A global perspective.
532 PLoS One. 2021;16(9):e0257333.
533 12.	Tegegne TK, Sisay MM. Menstrual hygiene management and school absenteeism
534 among	female	adolescent	students	in	Northeast	Ethiopia.	BMC	Public	Health.
535 2014;14(1):1118.
536 13.	Keppens G. School absenteeism and academic achievement: Does the timing of the
537 absence matter? Learning and Instruction. 2023;86:101769.
538 14.	Miiro G, Rutakumwa R, Nakiyingi-Miiro J, Nakuya K, Musoke S, Namakula J, et al.
539 Menstrual health and school absenteeism among adolescent girls in Uganda (MENISCUS): a
540 feasibility study. BMC Women's Health. 2018;18(1):4.
541 15.	Nelson AKM, Stephen Lagony M, Catherine Kansiime P, Belen Torondel P, Clare
542 Tanton P, Denis Ndekezi M, et al. Effects and costs of a multi-component menstrualhealth
543 intervention (MENISCUS) on
544 mental health problems, educational performance, and menstrual health in Ugandan
545 secondary schools: an open-label, school-based, cluster-randomised controlled trial -in press.
546 The Lancet Global Health (in press). 2025.
547 16.	Statistics UBo. The National Population and Housing Census 2024 – Final Report -
548 Volume 1 (Main). Kampala, Uganda: Uganda Bureau of Statistics; 2024 DECEMBER 2024.
549 17.	UBOS. The national population and housing census 2014 – area specifc profle series -
550 Wakiso. Kampala: UBOS; 2017.
551 18.	UBOS. The National Population and Housing Census 2014 – Area
552 Specific Profile Series-Kalungu district. Kampala: UBOS; 2017.
553 19.	Kansiime C, Hytti L, Nelson KA, Torondel B, Francis SC, Tanton C, et al. Menstrual
554 health interventions, schooling, and mental health problems among Ugandan students
555 (MENISCUS):	study	protocol	for	a	school-based	cluster-randomised	trial.	Trials.
556 2022;23(1):759.
557 20.	Hunter EC, Murray SM, Sultana F, Alam MU, Sarker S, Rahman M, et al.
558 Development and validation of the Self-Efficacy in Addressing Menstrual Needs Scale
559 (SAMNS-26) in Bangladeshi schools: A measure of girls' menstrual care confidence. PLoS
560 One. 2022;17(10):e0275736.
561 21.	Hennegan J, Nansubuga A, Smith C, Redshaw M, Akullo A, Schwab KJ. Measuring
562 menstrual hygiene experience: development and validation of the Menstrual Practice Needs
563 Scale (MPNS-36) in Soroti, Uganda. BMJ Open. 2020;10(2):e034461.
564 22.	Sterne JA, Davey Smith G. Sifting the evidence-what's wrong with significance tests?
565 Bmj. 2001;322(7280):226-31.
566 23.	Evans DK, Mendez Acosta A. How to measure student absenteeism in low- and
567 middle-income countries. Economics of Education Review. 2023;96:102454.
568 24.	Keppens G, Spruyt B, Dockx J. Measuring School Absenteeism: Administrative
569 Attendance Data Collected by Schools Differ From Self-Reports in Systematic Ways. Front
570 Psychol. 2019;10:2623.
571 25.	Mason L, Nyothach E, Alexander K, Odhiambo FO, Eleveld A, Vulule J, et al. 'We
572 keep it secret so no one should know'--a qualitative study to explore young schoolgirls
573 attitudes and experiences with menstruation in rural western Kenya. PLoS One.
574 2013;8(11):e79132.
575 26.	El-Gilany AH, Badawi K, El-Fedawy S. Menstrual hygiene among adolescent
576 schoolgirls in Mansoura, Egypt. Reprod Health Matters. 2005;13(26):147-52.
577 27.	Mason L, Nyothach E, Alexander K, Odhiambo FO, Eleveld A, Vulule J, et al. ‘We
578 Keep It Secret So No One Should Know’ – A Qualitative Study to Explore Young
579 Schoolgirls Attitudes and Experiences with Menstruation in Rural Western Kenya. PLOS
580 ONE. 2013;8(11):e79132.
581 28.	Korpershoek H, T. CE, M. F-B, and de Boer H. The relationships between school
582 belonging and students’ motivational, social-emotional, behavioural, and academic outcomes
583 in	secondary	education:	a	meta-analytic	review.	Research	Papers	in	Education.
584 2020;35(6):641-80.
585 29.	Topistar N, Dennis Zami Atibuni P, David Kani Olema P, Ujeyo MSS. Demystifying
586 Menstrual Health Management to Enhance Academic Performance among Adolescent
587 Primary School Girls in Rural Uganda. East African Journal of Education Studies.
588 2022;5(2):298-309.

589

590

Figure	[Click here to access/download;Figure;Figure 1.jpg](https://www2.cloud.editorialmanager.com/pone/download.aspx?id=40626751&guid=c8d9bd13-07ba-4e95-9419-7c9293b896d0&scheme=1)[<https://www2.cloud.editorialmanager.com/pone/download.aspx?id=40626751&guid=c8d9bd13-07ba-4e95-9419-7c9293b896d0&scheme=1>](https://www2.cloud.editorialmanager.com/pone/download.aspx?id=40626751&guid=c8d9bd13-07ba-4e95-9419-7c9293b896d0&scheme=1)

Supporting Information

[Click here to access/download](https://www2.cloud.editorialmanager.com/pone/download.aspx?id=40626756&guid=29affd30-8682-4bc4-a78e-628b8016556c&scheme=1)

[**Supporting Information** Supplementary Table 1.docx](https://www2.cloud.editorialmanager.com/pone/download.aspx?id=40626756&guid=29affd30-8682-4bc4-a78e-628b8016556c&scheme=1)

Supporting Information

[Click here to access/download](https://www2.cloud.editorialmanager.com/pone/download.aspx?id=40999652&guid=0472ca9e-df89-4a0c-a6f2-68b79c4b3655&scheme=1)

[**Supporting Information** Supporting letter.docx](https://www2.cloud.editorialmanager.com/pone/download.aspx?id=40999652&guid=0472ca9e-df89-4a0c-a6f2-68b79c4b3655&scheme=1)

Amendment Approval Letter

[Click here to access/download](https://www2.cloud.editorialmanager.com/pone/download.aspx?id=40671564&guid=8a5334ea-1663-474a-a57b-fa02af6184b9&scheme=1)

# [Other](https://www2.cloud.editorialmanager.com/pone/download.aspx?id=40671564&guid=8a5334ea-1663-474a-a57b-fa02af6184b9&scheme=1)

## [Amendment Approval Letter.pdf](https://www2.cloud.editorialmanager.com/pone/download.aspx?id=40671564&guid=8a5334ea-1663-474a-a57b-fa02af6184b9&scheme=1)

Letter of Approval from LSHTM

[Click here to access/download](https://www2.cloud.editorialmanager.com/pone/download.aspx?id=40671566&guid=5f365c93-1312-4e81-925d-379df4ce429b&scheme=1)

# [Other](https://www2.cloud.editorialmanager.com/pone/download.aspx?id=40671566&guid=5f365c93-1312-4e81-925d-379df4ce429b&scheme=1)

## [Letter of Approval from LSHTM_March2023.pdf](https://www2.cloud.editorialmanager.com/pone/download.aspx?id=40671566&guid=5f365c93-1312-4e81-925d-379df4ce429b&scheme=1)

LSHTM LEO Main trial amendment approval

[Click here to access/download](https://www2.cloud.editorialmanager.com/pone/download.aspx?id=40671567&guid=44ac1677-213c-4afd-ac9d-262f20970c36&scheme=1)

# [Other](https://www2.cloud.editorialmanager.com/pone/download.aspx?id=40671567&guid=44ac1677-213c-4afd-ac9d-262f20970c36&scheme=1)

## [LSHTM LEO Main trial amendment approval 200921.pdf](https://www2.cloud.editorialmanager.com/pone/download.aspx?id=40671567&guid=44ac1677-213c-4afd-ac9d-262f20970c36&scheme=1)

LSHTM LEO Main trial approval

[Click here to access/download](https://www2.cloud.editorialmanager.com/pone/download.aspx?id=40671568&guid=3a5f8c0b-8b52-4248-8a2c-3a2a7b0a6c3d&scheme=1)

# [Other](https://www2.cloud.editorialmanager.com/pone/download.aspx?id=40671568&guid=3a5f8c0b-8b52-4248-8a2c-3a2a7b0a6c3d&scheme=1)

## [LSHTM LEO Main trial approval 030821.pdf](https://www2.cloud.editorialmanager.com/pone/download.aspx?id=40671568&guid=3a5f8c0b-8b52-4248-8a2c-3a2a7b0a6c3d&scheme=1)

LSHTM_Amendment_Approval

[Click here to access/download](https://www2.cloud.editorialmanager.com/pone/download.aspx?id=40671570&guid=c7459d64-f28e-42a9-8641-d0e3c0f0df5f&scheme=1)

## [**Other** LSHTM_Amendment_Approval_Sept23.pdf](https://www2.cloud.editorialmanager.com/pone/download.aspx?id=40671570&guid=c7459d64-f28e-42a9-8641-d0e3c0f0df5f&scheme=1)

LSHTM_Amendment_Approval

[Click here to access/download](https://www2.cloud.editorialmanager.com/pone/download.aspx?id=40671571&guid=7bd6ab37-a158-4fe0-aaa0-b6dfa52347cf&scheme=1)

## [**Other** LSHTM_Amendment_Approval_v3.0_01Mar2022.pdf](https://www2.cloud.editorialmanager.com/pone/download.aspx?id=40671571&guid=7bd6ab37-a158-4fe0-aaa0-b6dfa52347cf&scheme=1)

MENISCUS Protocol Amendment Approval

[Click here to access/download](https://www2.cloud.editorialmanager.com/pone/download.aspx?id=40671572&guid=3b1dac5f-7b22-412d-8ba9-15799afe366f&scheme=1)

# [Other](https://www2.cloud.editorialmanager.com/pone/download.aspx?id=40671572&guid=3b1dac5f-7b22-412d-8ba9-15799afe366f&scheme=1)

## [MENISCUS Protocol Amendment Approval_15Feb2022.pdf](https://www2.cloud.editorialmanager.com/pone/download.aspx?id=40671572&guid=3b1dac5f-7b22-412d-8ba9-15799afe366f&scheme=1)

MENISCUS REC Amendment approval

[Click here to access/download](https://www2.cloud.editorialmanager.com/pone/download.aspx?id=40671597&guid=3beedaf3-912a-45fc-b3a5-e2ff24a87201&scheme=1)

# [Other](https://www2.cloud.editorialmanager.com/pone/download.aspx?id=40671597&guid=3beedaf3-912a-45fc-b3a5-e2ff24a87201&scheme=1)

## [MENISCUS REC Amendment approval.pdf](https://www2.cloud.editorialmanager.com/pone/download.aspx?id=40671597&guid=3beedaf3-912a-45fc-b3a5-e2ff24a87201&scheme=1)

MENISCUS trial Amendment approval

[Click here to access/download](https://www2.cloud.editorialmanager.com/pone/download.aspx?id=40671619&guid=1d904f98-2cec-4c90-8036-5c4fad0ce271&scheme=1)

# [Other](https://www2.cloud.editorialmanager.com/pone/download.aspx?id=40671619&guid=1d904f98-2cec-4c90-8036-5c4fad0ce271&scheme=1)

## [MENISCUS trial Amendment approval 18thMay2022.pdf](https://www2.cloud.editorialmanager.com/pone/download.aspx?id=40671619&guid=1d904f98-2cec-4c90-8036-5c4fad0ce271&scheme=1)

MENISCUS TRIAL AMENDMENT APPROVAL

[Click here to access/download](https://www2.cloud.editorialmanager.com/pone/download.aspx?id=40671622&guid=b822a166-ebfc-4805-b7a9-c7a080993564&scheme=1)

# [Other](https://www2.cloud.editorialmanager.com/pone/download.aspx?id=40671622&guid=b822a166-ebfc-4805-b7a9-c7a080993564&scheme=1)

## [MENISCUS TRIAL AMENDMENT](https://www2.cloud.editorialmanager.com/pone/download.aspx?id=40671622&guid=b822a166-ebfc-4805-b7a9-c7a080993564&scheme=1)

[APPROVAL_31August2021.pdf](https://www2.cloud.editorialmanager.com/pone/download.aspx?id=40671622&guid=b822a166-ebfc-4805-b7a9-c7a080993564&scheme=1)

MENISCUS trial amendment revised approval- Change of PI

[Click here to access/download](https://www2.cloud.editorialmanager.com/pone/download.aspx?id=40671623&guid=43fc8b32-a321-4f4d-b470-e5ad34cf492d&scheme=1)

# [Other](https://www2.cloud.editorialmanager.com/pone/download.aspx?id=40671623&guid=43fc8b32-a321-4f4d-b470-e5ad34cf492d&scheme=1)

## [MENISCUS trial amendment revised approval- Change of PI.pdf](https://www2.cloud.editorialmanager.com/pone/download.aspx?id=40671623&guid=43fc8b32-a321-4f4d-b470-e5ad34cf492d&scheme=1)

MENISCUS TRIAL REC Approval

[Click here to access/download](https://www2.cloud.editorialmanager.com/pone/download.aspx?id=40671633&guid=8ca1bb54-3e38-4b4b-93f5-92616d44e52a&scheme=1)

# [Other](https://www2.cloud.editorialmanager.com/pone/download.aspx?id=40671633&guid=8ca1bb54-3e38-4b4b-93f5-92616d44e52a&scheme=1)

## [MENISCUS TRIAL REC Approval 26May2021.pdf](https://www2.cloud.editorialmanager.com/pone/download.aspx?id=40671633&guid=8ca1bb54-3e38-4b4b-93f5-92616d44e52a&scheme=1)

UNCST MENISCUS Main trial approval

[Click here to access/download](https://www2.cloud.editorialmanager.com/pone/download.aspx?id=40671637&guid=12481480-13c6-42f4-a0dd-d1562836e4fc&scheme=1)

# [Other](https://www2.cloud.editorialmanager.com/pone/download.aspx?id=40671637&guid=12481480-13c6-42f4-a0dd-d1562836e4fc&scheme=1)

## [UNCST MENISCUS Main trial approval 14July2021.pdf](https://www2.cloud.editorialmanager.com/pone/download.aspx?id=40671637&guid=12481480-13c6-42f4-a0dd-d1562836e4fc&scheme=1)

UVRI Amendment Approval

[Click here to access/download](https://www2.cloud.editorialmanager.com/pone/download.aspx?id=40671642&guid=17ab12f6-b449-436d-ad3d-a12ec43155ce&scheme=1)

# [Other](https://www2.cloud.editorialmanager.com/pone/download.aspx?id=40671642&guid=17ab12f6-b449-436d-ad3d-a12ec43155ce&scheme=1)

## [UVRI Amendment Approval_Dec2022.pdf](https://www2.cloud.editorialmanager.com/pone/download.aspx?id=40671642&guid=17ab12f6-b449-436d-ad3d-a12ec43155ce&scheme=1)

UVRI Approval June

[Click here to access/download](https://www2.cloud.editorialmanager.com/pone/download.aspx?id=40671652&guid=b905c603-eec6-4d59-9788-d20ef54823fe&scheme=1)

# [Other](https://www2.cloud.editorialmanager.com/pone/download.aspx?id=40671652&guid=b905c603-eec6-4d59-9788-d20ef54823fe&scheme=1)

## [UVRI Approval June 23.pdf](https://www2.cloud.editorialmanager.com/pone/download.aspx?id=40671652&guid=b905c603-eec6-4d59-9788-d20ef54823fe&scheme=1)

UVRI_MENISCUS TRIAL AMENDMENT APPROVAL

[Click here to access/download](https://www2.cloud.editorialmanager.com/pone/download.aspx?id=40671654&guid=93407f47-7f5a-4a8d-a031-12731ed0644a&scheme=1)

## [**Other** UVRI_MENISCUS TRIAL AMENDMENT](https://www2.cloud.editorialmanager.com/pone/download.aspx?id=40671654&guid=93407f47-7f5a-4a8d-a031-12731ed0644a&scheme=1)

[APPROVAL_31August2021.pdf](https://www2.cloud.editorialmanager.com/pone/download.aspx?id=40671654&guid=93407f47-7f5a-4a8d-a031-12731ed0644a&scheme=1)

CONSORT FOR THE TRIAL_PUBLISHED EARLIER

[Click here to access/download](https://www2.cloud.editorialmanager.com/pone/download.aspx?id=40999718&guid=7a9d0cab-79ee-41a4-b3d5-1c8628f095fb&scheme=1)

# [Other](https://www2.cloud.editorialmanager.com/pone/download.aspx?id=40999718&guid=7a9d0cab-79ee-41a4-b3d5-1c8628f095fb&scheme=1)

## [CONSORT FOR TRIAL RESULTS PAPER.pptx](https://www2.cloud.editorialmanager.com/pone/download.aspx?id=40999718&guid=7a9d0cab-79ee-41a4-b3d5-1c8628f095fb&scheme=1)

TRIAL PROTOCOL PAPER

[Click here to access/download](https://www2.cloud.editorialmanager.com/pone/download.aspx?id=40999728&guid=4daabcdc-a0cb-418b-a813-43510209acfe&scheme=1)

# [Other](https://www2.cloud.editorialmanager.com/pone/download.aspx?id=40999728&guid=4daabcdc-a0cb-418b-a813-43510209acfe&scheme=1)

[MENISCUS Protocol Version v7.0 February 2024.pdf](https://www2.cloud.editorialmanager.com/pone/download.aspx?id=40999728&guid=4daabcdc-a0cb-418b-a813-43510209acfe&scheme=1)
