# Supplementary material for: Associations of menstrual health with school absenteeism and examination performance among Ugandan secondary school students: A prospective study
Source: PLoS One. 2026 Jan 23;21(1):e0326549. doi: 10.1371/journal.pone.0326549 (PMC12829969; doi:10.1371/journal.pone.0326549)
Supplement: Attachment — Submitted filename: Response to Reviewers.docx [file pone.0326549.s007.docx]

Dear Editor

Thank you for reviewing the manuscript. We have revised our manuscript to address the Editor and reviewer comments. Our detailed responses are below.

1.Please ensure that your manuscript meets PLOS ONE's style requirements, including those for file naming. The PLOS ONE style templates can be found at

**Response: We have followed these style requirements**

2. Please note that funding information should not appear in any section or other areas of your manuscript. We will only publish funding information present in the Funding Statement section of the online submission form. Please remove any funding-related text from the manuscript.

**Response: The funding statement has been removed from manuscript and is only in the Funding Statement.**

3. When completing the data availability statement of the submission form, you indicated that you will make your data available on acceptance. We strongly recommend all authors decide on a data sharing plan before acceptance, as the process can be lengthy and hold up publication timelines. Please note that, though access restrictions are acceptable now, your entire data will need to be made freely accessible if your manuscript is accepted for publication. This policy applies to all data except where public deposition would breach compliance with the protocol approved by your research ethics board. If you are unable to adhere to our open data policy, please kindly revise your statement to explain your reasoning and we will seek the editor's input on an exemption. Please be assured that, once you have provided your new statement, the assessment of your exemption will not hold up the peer review process.

**Response: We confirm that the data are freely accessible upon request at the LSHTM Data Compass repository (**[MENISCUS Trial quantitative survey data](https://datacompass.lshtm.ac.uk/id/eprint/3865/)**) line 518-522**

4. Your ethics statement should only appear in the Methods section of your manuscript. If your ethics statement is written in any section besides the Methods, please move it to the Methods section and delete it from any other section. Please ensure that your ethics statement is included in your manuscript, as the ethics statement entered into the online submission form will not be published alongside your manuscript.

**Response: Thank you for the guidance. The ethic statement has been moved to the Methods section (lines 238-242)**

5. Please include a separate caption for each figure in your manuscript.

**Response: We have two figures and provide captions in the manuscript (lines 233 and lines 258 respectively).**

6. Please include captions for your Supporting Information files at the end of your manuscript, and update any in-text citations to match accordingly. Please see our Supporting Information guidelines for more information:

**Response: We have added the caption for the Supporting Information file at the end of the manuscript (line 640) and its in-text citation has been updated on lines 333 and line 357**

7. Please upload a copy of your study protocol that was approved by your ethics committee/IRB as a Supporting Information file. By the study protocol, we mean the complete and detailed plan for the conduct and analysis of the trial approved by the ethics committee/IRB. Please send this in the original language. If this is in a language other than English, please also provide a translation.

**Response: The protocol has been uploaded as a supporting file**

8. If the reviewer comments include a recommendation to cite specific previously published works, please review and evaluate these publications to determine whether they are relevant and should be cited. There is no requirement to cite these works unless the editor has indicated otherwise. 

**Response: There were no such comments**

**Reviewer Comments to the Author**

**Reviewer #1:**

The paper lacks clarity from the statistical perspective. The statistical strategy is poorly presented in this study. Section 10 of the supplemental protocol is well written with sample size justification and various analysis options per endpoints discussed.

**Response: Thank you for this comment. We have substantially revised the section on statistical analysis (Section 2.7) to clarify how we followed a conceptual framework (Figure 1) to build the multivariable models. Specifically, this first describes the two types of models used (mixed-effect negative binomial for school absenteeism, and mixed-effects linear regression for examination score) and then describes the conceptual framework and how the multivariable models were built for each level.**

**As this study was nested within the MENISCUS cluster-randomised controlled trial, we did not conduct a sample size calculation for this nested analysis.**

When one starts reading the paper, the design and parallel arms not even introduced in the Abstract. One has to wait until section 2.2 and the investigators finally state that the trial was a parallel-arm, cluster-randomized controlled trial in 60 schools with schools randomized 1:1 to either immediate or delayed intervention delivery.

**Response: We apologise for the confusion with the study design. We have clarified that this manuscript reports results from a secondary observational analysis using data from the published MENISCUS trial. It has been clarified in the abstract on lines 29-30, in the introduction on lines 114-119, and the methods on lines 130-144.**

1. What is the control arm mentioned in the abstract?

**Response: This is the control arm from the trial within which the current analyses are nested. We have clarified this in the abstract as follows “We conducted secondary observational analyses using data from a cluster-randomised trial of a menstrual health intervention in 60 Ugandan secondary schools. We used baseline data from trial participants in both arms, and endline data from the control arm participants. “(lines 29-30). We also now include a study flowchart (Figure 2) to help clarify this.**

2. Also, the actual longitudinal input is not clear to the reader. Please relate immediate, delayed, and endline in a longitudinal context with respect to Figure 1.

**Response: We thank the reviewer for this comment, and have removed the word ‘longitudinal’ throughout the manuscript. Instead, we describe the study as a prospective study in the title, and refer to baseline and endline data analysis in the abstract and manuscript (e.g. lines 29-30, 43-45 in the abstract), including in Figures 1 and the new Figure 2 which is the study flowchart.**

3. In the Conclusion the authors note that among Ugandan students, multiple dimensions of menstrual health are associated with school absenteeism and educational performance. Where is the analysis of immediate or delayed intervention or is that not part of this report?

**Response: Thank you for the comment. We have clarified in the Introduction section of the manuscript that this manuscript reports secondary data analysis of the trial, and that the intervention effects have been published previously (lines 103-113). We have also clarified that the estimate of the magnitude of effects in this paper help explain the lack of intervention effect (lines 380-383) “Through this observational analysis, we have shown that dimensions of menstrual health are associated with educational outcomes, but the absolute effect was small, which may help explain why the intervention was not sufficient to improve these educational outcomes.”**

4 It looks like Tables 2 to 4 are baseline and Table 5 are endline results from the longitudinal approach? Is that correct?

**Response: Yes that is correct. We apologise for an error in title of Table 5 which suggested the outcomes were at baseline (line 361).**

5. Some sentences are vague. For example ,please explain line 292, ’ After adjusting for factors at the same or more distal levels, there was strong evidence that menstrual-related absenteeism and poorer examination performance were both associated…etc’. What does 'distal level' mean? Please be exact when you mention adjustment throughout the presentation.

**Response: Thank you for this comment. We have revised the manuscript throughout to improve clarity. This includes rewriting Section 2.7 on Statistical Analyses to clarify the model building strategy . This includes definition of ‘distal level’ (to say “We use the term “distal” to refer to variables hypothesized to causally precede and influence variables at more proximal levels” (lines 209-220).**

6. The entire presentation has to be edited for organization and clarity.

**Response: We thank the reviewer for this comment and have edited the manuscript throughout to improve clarify.**

**Reviewer #2:**

1. Abstract
• Clearly explain how factors such as inadequate menstrual materials, negative attitudes toward menstruation, unmet menstrual practice needs, and experiences of menstrual teasing influence educational performance, not just absenteeism.

**Response: We had included this in the discussion but have moved to the introduction (lines 55-59).**“**Poor menstrual health is prevalent in low- and middle-income countries (LMICs), and may affect educational outcomes such as school absenteeism and examination performance through multiple pathways, including through shame, anxiety, lack of confidence, behavioural expectations, poor physical environments, and inadequate menstrual products”**

Also clarify what is meant by “multiple dimensions of menstrual health” and how these are associated with both absenteeism and educational performance (may be in the body).

**Response: We have defined the dimensions of menstrual health in the introduction (lines 70-73) “These dimensions include physical, behavioural and social components such as inadequate menstrual materials, menstrual pain, poor menstrual knowledge and confidence, and inadequate infrastructure for managing menstruation in schools”**

• Article type: Why is this described as a clinical trial? Since the study used secondary data and so sign of any medical test. Please justify the article type or indicate this clearly in the methods section.

**Response: Thank you for the comment. We have clarified in the manuscript that the paper is a secondary analysis using data from a cluster-randomised controlled trial (lines 123-124) and corrected this in the submission form.**

2. Introduction
• Citations: Use square brackets for in-text citations instead of parentheses, to maintain consistency with journal style.

**Response: Thank you. These have been changed to square brackets in line with journal style.**

• Objective: Ensure the objective gives equal emphasis to both class absenteeism and educational performance. This balance appears lacking in the last part of the introduction. **Response: We have revised the objective to include “examination performance” (line 114-117) and have revised the manuscript throughout to improve the balance.**

• MENISCUS: Define this acronym (e.g., “MENISCUS stands for...”) before using it, to help readers unfamiliar with the term.

**Response: We have defined “MENISCUS” as “Menstrual health interventions, schooling, and mental health problems among Ugandan students (MENISCUS)” at first use on lines 112-113.**

3. Methods
• Government vs private schools: What was the purpose of sampling both government and private schools if the outcomes are not contrasted by school type? Please clarify.

**Response: We have explained reasons for sampling both government and private schools on lines 156-158: “We over-sampled government schools as these are of interest to Ministry of Education stakeholders, and to increase generalisability of findings to these schools”.**

• Interventions: Specify the interventions received by the treatment schools versus what the control schools received.

**Response: We have added brief details of the intervention components for completeness on lines 137-140. We have also clarified that control schools were provided with a copy of the government guidelines on menstrual hygiene management and sexuality education and menstrual management reader.**

One of the selection criteria mentioned was the presence of WASH services, suggesting there might not have been an intervention. Please clarify this point. If there were interventions, how they were managed and how frequent they were?

**Response: We have clarified that we only use baseline trial data from the intervention arm in this paper, plus endline data from the control arm (lines 131-135) so there are no post-intervention data from the intervention arm. The WASH services mentioned were already present at the schools at baseline, and having “a minimum WASH facility” was one of the trial eligibility criteria. We have clarified this on lines 148-155.**

• Quality control: What methods were employed to minimize recall bias in reporting class absenteeism?

**Response: We have revised the methods to clarify “To minimize recall bias, we used cognitive testing before the survey to ensure clearly worded questions anchored to specific time frames, and provided reminders of school terms and holidays during survey completion. During self-completion of the questionnaires, research assistants were present to assist participants with clarifications about the recall period and absence reasons.” Lines 192-196**

• If the main results of MENISCUS have already been published, what new insights does this data analysis provide? Clearly, highlight the novel contributions/how different is this finding?

**Response: We have revised the discussion to highlight the new insights provided by this analysis (lines 376-382). “Through this observational analysis, we have shown that multiple dimensions of menstrual health are associated with educational outcomes, but the absolute effect was small, which may explain why the intervention was not sufficient to improve these educational outcomes”.**

• Why did you use both cross-sectional and longitudinal designs? Are there sufficient indicators that support the longitudinal approach? It seems data were collected only at baseline, end line, and through student self-reports, with no evidence of continuous data collection.

**Response: We chose to analyse prospective data in the control arm to address the possibility of reverse causality which could be in the baseline cross-sectional data. For example, it is possible that students with more absenteeism missed learning about how to manage their menstruation, and had poorer menstrual experiences. We chose to restrict this analysis to the control arm to assess this relationship in the absence of the MENISCUS intervention and hence make the results more generalisable. We have clarified this in the methods section (lines 227-231).**

**“To mitigate against the possibility of reverse causality, and to assess temporal associations of exposures with the outcomes, we also analysed associations of baseline exposures with endline outcomes in the control arm, using analogous methods. We restricted these analyses to participants in the control arm as the MENISCUS intervention may have modified the association.”**

4.

Results
• Findings on the effects of menstrual health on educational performance are limited and not strongly demonstrated. Consider discussing this limitation.

**Response: Thank you for your comment. We have improved the discussion for example line 434-436 “However, overall, associations of dimensions of menstrual health with examination scores were weaker than with school absenteeism. This is as expected given that absenteeism is more proximal to poor menstrual health than educational performance”**

**We have revised the limitations section to note that these findings are modest and should be interpreted cautiously due to potential limitations such as residual confounding and limited sensitivity of educational measures (Lines 458-462). Also future research with longer follow-up, larger sample sizes, more sensitive and diverse academic outcome measures, and designs that better isolate menstrual health effects from other educational influences is needed to fully understand the impact of menstrual health on academic achievement (lines 479-482)**

General comments
• Clearly distinguish between this study and the original MENISCUS study to avoid confusion.

**Response: Thank you for the comment. We have revised the paper throughout to clarify that this paper uses baseline data from the trial, plus endline data in the control arm, and does not estimate intervention effects on the educational outcomes as these were presented in the trial paper. This is highlighted in the introduction (lines 114-117), methods (lines 131-135), and discussion (lines 379-382).**

• Explicitly state the specific menstrual-related factors analyzed, instead of repeatedly using the vague term “multiple dimensions.” This will make your findings clearer and more impactful.

**Response: Thank for the guidance. We have edited the paper to specify the menstrual-related factors throughout the manuscript.**

• Follow PLOS ONE reference format

**Response: We apologise for this oversight. The citations are now in the PLoS One format.**

**Reviewer #3:**

The authors have presented an intelligble fashion and the grammar is standard. The author should look at the punctuation and the tenses. The methodolgy is okay and the it is technically sound. The authour should avoid being too wordy. Some paragraphs are too long. He should make them brief and to the point.

**Response: We thank the reviewer for their comments, and have revised the manuscript throughout for clarity, including shortening sentences and paragraphs.**

**Reviewer #4:**

Abstract

Overall the abstract needs a bit of work, context and clarification. Please provide a very brief (understanding the tight word count) description of the intervention in the methods (or background) to help orient the reader.

**Response: We thank the reviewer for their comment and have edited the abstract, including clarification that this is secondary analysis of observation (baseline and endline) data from cluster randomized trial (line 29-30). We prefer not to include a description of the intervention in the abstract as we are not using any post-intervention data in this analysis.**

39: Do you mean “we analysed baseline and endline data”?

**Response: Thank you for this correction. We have edited the abstract as suggested (line 31-32).**

The results section needs tailoring.

47: At baseline 3312 participants reported menstruating in the last 6 months. 3312 of how many? Or are you trying to say that your sample was the 3312 participants who reported menstruating in the last 6 months. Please clarify or consider revising: “Of the 3312 participants who reported menstruating in the past 6-months at baseline, 323 (9.8%) reported missing at least one day of school per month due to menstruation.”

**Response: We have edited the sentence as suggested (line 39-41)**

49: 1286 (38.8%) missed at least one day a month for any reason. Is this relevant? Is this key to be reported in the abstract? Without context it lacks definition of why it is relevant.

**Response: We have removed this sentence from the abstract.**

50: Of the 1192 participants in the control arm seen at endline, 135 (11.3%) missed at 51 least one day due to menstruation. Why is the control endline reported, and not the intervention arm endline? I don’t understand the relevance of reporting the control endline school absenteeism without the intervention data. What are you trying to say here? Can you add some clarifying text or otherwise provide some other data to clarify why this has been reported.

**Response: We clarified that we are reporting results for a secondary data of observation analysis (line 29-30) and clarified our rationale for not including intervention arm participants in the endline data analysis in the methods section, also this is not an evaluation analysis (lines 227-231) “To mitigate against the possibility of reverse causality, and to assess temporal associations of exposures with the outcomes, we also analysed associations of baseline exposures with endline outcomes in the control arm, using analogous methods. We restricted these analyses to participants in the control arm in the MENISCUS intervention which may have moderated the association”**

53: associated with multiple dimensions of menstrual health. Multiple dimensions of poor menstrual health. The bracketed examples are the inverse of the dimensions on the conceptual model (described later). Potentially refer to the model here, saying “as per a conceptual model described in the paper” - or something to orient a reader who is not familiar with the model. Another way might be to use the phrasing as per the introduction “aspects of menstrual experience”. Perhaps instead of brackets, change to such as…

**Response: We thank the reviewer for this suggestion. We have removed the wording about ‘dimensions’ of menstrual health in the results section in favour of specific menstrual practices, although we retain it in the conclusion to summarise the findings (lines 43-46). “There was evidence that menstrual-related absenteeism and poorer examination performance at endline were both associated with use of inadequate menstrual materials, negative menstrual attitudes, unmet menstrual practice needs, and experience of menstrual-related teasing”.**

55: Menstrual teasing. Please use term menstrual-related teasing. “Menstrual teasing” is not correct (and not used consistently throughout paper). Note: Line 306: “menstrual teasing” and Line 324 should be edited for consistency.

**Response: We have corrected this throughout the manuscript.**

Introduction

71: A widely-cited statistic that “one in 10 school-age girls in Africa misses school or drops out for reasons related to her period” is not evidence-based (3, 4). I get what you are trying to say here, but it reads strangely to me. Consider rephrasing. “One in ten school-age girls in Africa missed school or drops out for reasons related to her period” is a widely –cited statistic that lacks scientific backing.

**Response: Thank you for the suggestion. We have modified the sentence to “The statement "one in 10 school-age girls in Africa misses school or drops out for reasons related to her period" is a widely-used statistic, but it is not supported by scientific research [3, 4]. “ (lines 65-66)**

74: Consider: drop female – redundant. An average of 31%?

**Response: We have edited as suggested (line 68).**

75: Edit: Far too long sentence. Use full-stop after longer duration, then begin sentence with Additionally, the review found menstruation also affected...

**Response : Thank you, the sentence has been revised and edited (lines 70-71).**

89: Consider changing knowledge to evidence or data.

**Response: We have revised as suggested (line 80).**

96: LMICs
**Response: We have revised as suggested (line 81).**

99: Change to: ...with academic performance a reported consequence of menstruation, mainly due to menstrual pain or heavy menstrual bleeding.

**Response: We have revised this as “**A commonly-reported reason for menstrual-related absenteeism is menstrual pain, which can also contribute to reduced participation, concentration, and academic performance” **(line 89-90).**

102: Broader educational issues are written here as self-esteem, school participation, and educational performance. How is educational performance difference from academic performance? Above has been discussing academic performance, please clarify how or if educational performance is different and thus belongs to “broader educational issues.”

**Response: We have clarified the paper throughout to refer to educational outcomes as both school absenteeism and examination performance, and have replaced ‘academic performance’ and ‘educational performance’ throughout with either ‘educational outcomes’ or ‘examination performance’ as appropriate.**

102/103: Should this sentence: “In the review among university students...”come above the sentence “There is relatively little and inconclusive evidence..” Im confused as to why this sentence separates the following comment on the review. Please edit or justify.

**Response: We thank the reviewer for the comment, and have edited as suggested.**

109: You introduce us to: “Our recent cluster-randomised trial” is this the same cluster-randomised trail described in the methods? Aka the MENISCUS? If so, introduce it here not below. Please clarify or change the use of “our.” This is confusing.

**Response: Yes it’s the same, we have clarified this as follows: “Our recent cluster-randomised trial, the MENISCUS study (“Menstrual health interventions, schooling, and mental health problems among Ugandan students”) showed strong evidence that a multi-component menstrual health intervention improved multiple dimensions of menstruation, including knowledge about puberty and menstruation, use of effective pain management, menstrual self-efficacy, and reduced unmet menstrual needs, over a one-year period [15]. However, Lines 103-104.**

111: Add: showed strong evidence of an intervention effect on multiple dimensions of menstrual health, such as....

**Response: Revised as above. Line 105-108**

Please detail what dimensions were effected, considering below you say little on school absence and nothing on examination performance, or school absence overall.

**Response: Revised, as above Line 108-113 “there was little evidence of an intervention effect on school absence (measured using prospectively-collected daily diaries) during menstruation (11.2% vs 13.5% days missed; adjusted odds ratio (aOR)=0.81, 95%CI 0.62, 1.05). There was also no evidence of an intervention effect on examination performance (adjusted mean difference (aMD)=0.06, 95%CI -0.12, 0.24) or on school absence overall (10.5% days missed in both arms).”**

Additionally, is examination performace to be understood as different to academic performance, or educational performance. Please standardize or define.

**Response: As mentioned above, we have clarified the wording to be consistent in using examination performance as the UNEB examination, and ‘educational outcomes’ to mean both school absenteeism and examination performance. We have removed reference to academic performance and educational performance.**

117- 121: I suggest moving this last introductory paragraph to the top of the introduction to orient the reader and introduce MENISCUS.
After the first sentence introducing the MENISCUS trial, please provide a brief line to describe what the trial is.

**Response: We would prefer to first introduce the reader to the literature on menstrual health and educational outcomes before focusing on the specific data source for this study, the MENISCUS trial, but will follow the Editor’s guidance on this.**

The last sentence in this paragraph has a typo. Do you mean “add to the knowledge-base" or “add to the existing evidence on the impact of..” I would also advise revising the sentence to read: “based on the impact of multiple dimensions of menstrual health..” or “based on the impact of menstruation..”

**Response: We have revised this sentence as follows : “In this paper, we provide new insights by quantifying the associations of menstrual health experiences (including pain management, social support, menstrual practices, and attitudes) with number of days of school absenteeism and examination performance“(lines 117-120).**

Methods

124: Add “The MENSISCUS trial/study”

**Response: Revised (line 123)**

132: Be consistent with abstract ie: baseline and endline.

**Response: We have clarified this throughout to be consistent.**

132: This should not be the first time you are spelling out the acronym for MENISCUS. Please introduce in the introduction as suggested and spell out on first use.

**Response: The acronym has been included in the introduction (line 103-104).**

142-143: Decapitalise Education Institutions.

**Response: This has been corrected line 148**

143-148: This sentence is too long. Full-stop at 146 and then start sentence with “School enrolment was to be estimated as....”

**Response: This has been corrected.**

148: When you say “we” here, I am confused if you are talking about MENISCUS or your analysis of the trial? There needs to be clearer description of how what you are doing is related to MENISCUS and if you referring to it as “yours” etc.

**Response: We apologise for the confusion. The sampling was done once, for the trial, by the same team that conducted the analysis for the current manuscript. We have removed the word ‘we’ here as follows:** A random sample of 60 schools were selected after being confirmed as eligible and willing to participate**. Line 155-156**

151: Provide explanation of Secondary 2 in parenthesis as above for School 1.

**Response: We have clarified this as “Secondary 2 (second year of secondary school)” (line 160)**

166-167: Decapitalise subject names (Mathematics, Biology, English Language) (English remains capitalised- but not language)

**Response: We have corrected this (line 177-178)**

187: Different reference style

**Response: This has been corrected (line 209).**

188: Here you use the term “Menstrual-related factors”. I am wondering if you mean the same when you talk about “dimensions of menstrual health or “aspects of menstrual experience.”
If so, could you please choose one, define it and standardize throughout the paper.

**Response: We have edited this (line 169-172) and elsewhere to be ‘dimensions of menstrual health’ which we have defined earlier (lines 61-64) as “multiple dimensions of menstrual health contribute to school absenteeism and reduced engagement [2]. These dimensions include physical, behavioural and social components such as inadequate menstrual materials, menstrual pain, poor menstrual knowledge and confidence, and inadequate infrastructure for managing menstruation in schools”**

190: Delete full-stop after confounders.”

**Response: We have corrected this (line 210).**

198: See 188

**Response: Corrected**

242: Describe/contextualise MPSN score. Is 2.11positive/negative?

**Response: We have clarified as follows (lines 270-272). “The mean MPNS score of 2.11 (SD=0.52) corresponds to an average response of “sometimes” having unmet menstrual practice needs. The possible range is 0 to 3, with 3 corresponding to no unmet needs.”**

Discussion:

355: “These results add..”

**Response: We have corrected this (line 373)**

365: Support – remove s

**Response: We have revised the sentence to “Our finding that 10% of participants reported missing at least one school day per month due to menstruation supports the widespread claim that one in 10 girls miss school due to menstruation” (line 386-388)**

378: Sentence need clarification - “but reported that other girls were absent from school due to menstruation” or “but reported that other girls reported absenteeism”?

**Response: We have clarified this: “but reported that other girls were absent from school due to menstruation” (line 403)**

381-382: Perhaps belongs in strengths and limitations

**Response: We prefer to retain this here as it is a recommendation, rather than a strength or limitation of our study**

386: Suggest without adequate (not with inadequate).
**Response: We have made this change (line 412)**

392: Menstrual preparedness. Where is this definition of menstrual preparedness from? I don't see obtaining, using, cleaning and disposing of menstrual products and seeking assistance when needed as menstrual preparedness. Menstrual preparedness is yes about tracking cycle and body literacy. Or pre-menarche about prior knowledge, education and support

**Response: We have clarified this by defining the menstrual preparation and Maintenance sub-scale as ‘menstrual preparedness” in Table 1, and referring to this Table in the sentence referred to by the reviewer (line 417-418).**

**SCREEN-IT review**

Flow charts and attrition: We strongly recommend using flow charts because they provide an

overview of the study design and more information about attrition.

**Response: We have added the flowchart as Figure 2.**

Blinding: Please specify whether blinding was used at various phases of the experiment (e.g., blinding of patients, caregivers, outcome assessments, data analysis).

**Response: As this paper is not about intervention trial effects, we did not include this detail, but cite the main trial paper which includes details on blinding.**

Please state how you determined the sample size for the study. If you performed a power or sample size calculation, please provide details

**Response: As this study was nested within the MENISCUS cluster-randomised controlled trial, we did not conduct a sample size calculation for this nested analysis.**
