# Supplementary material for: Associations of menstrual health with school absenteeism and examination performance among Ugandan secondary school students: A prospective study
Source: PLoS One. 2026 Jan 23;21(1):e0326549. doi: 10.1371/journal.pone.0326549 (PMC12829969; doi:10.1371/journal.pone.0326549)
Supplement: Attachment — Submitted filename: PONE-D-25-29440_R1.docx [file pone.0326549.s008.docx]

PLOS ONE

**Associations of menstrual health with school absenteeism and examination performance among Ugandan secondary school students: A prospective study**

--Manuscript Draft--

| **Manuscript Number:** | PONE-D-25-29440R1 |
| --- | --- |
| **Article Type:** | Research Article |
| **Full Title:** | Associations of menstrual health with school absenteeism and examination performance among Ugandan secondary school students: A prospective study |
| **Short Title:** | Menstrual health and educational outcomes among secondary school students in Uganda |
| **Corresponding Author:** | Christopher Baleke, MSc  MRC/UVRI Uganda Research Unit On AIDS: MRC/UVRI and LSHTM Uganda Research Unit  Kampala, UGANDA |
| **Keywords:** | Menstrual health, school absenteeism, education outcomes |
| **Abstract:** | Background: Relatively few studies have quantified the amount of school missed due to poor menstrual health, or the impact of poor menstrual health on examination performance.  Methods: We conducted secondary observational analyses from data nested within a cluster-randomised trial of a menstrual health intervention in 60 Ugandan secondary schools (The trial is registered as ISRCTN45461276). We used baseline data from trial participants in both arms, and endline data from the control arm participants. School absenteeism was estimated as the self-reported number of days absent due to menstruation per month and examination performance was assessed by an independently set assessment by the Uganda National Examination Board. We estimated adjusted incidence rate ratios (aIRR) for associations with school absenteeism, using negative binomial regression adjusted for school-level clustering. We estimated adjusted standardised mean differences (aSMD) in examination scores using mixed-effects linear regression.  Results: Of the 3312 participants who reported menstruating in the past 6 months at baseline, 323 (9.8%) reported missing at least one day of school per month due to menstruation (mean days missed=0.30 per month, 95%CI 0.27-0.34). Similarly, of the 1192 participants in the trial control arm seen at endline, 135 (11.3%) reported missing at least one day due to menstruation (mean days missed=0.31 per month (95%CI 0.27-0.37)). There was evidence that menstrual-related absenteeism and poorer examination performance at endline were both associated with baseline use of inadequate menstrual materials, negative menstrual attitudes, unmet menstrual practice needs, and experience of menstrual-related teasing. In addition, absenteeism due to menstruation was associated with menstrual pain, and poorer examination performance was associated with poorer baseline menstrual knowledge.  Conclusion: Among Ugandan students, multiple dimensions of menstrual health are associated with school absenteeism and examination performance. |
| **Order of Authors:** | Christopher Baleke, MSc |
|  | Levicatus Mugenyi, MSc, PhD |
|  | Kate A Nelson, MSc |
|  | Katherine A Thomas, MSc |
|  | Denis Ndekezi, MSc |
|  | Jonathan Reuben Enomut, BA |
|  | Connie Alezuyo, MSc |
|  | John Jerrim, MSc, PhD |
|  | Helen A Weiss, MSc, PhD |

| **Response to Reviewers:** | Dear Editor  Thank you for reviewing the manuscript. We have revised our manuscript to address the Editor and reviewer comments. Our detailed responses are below.   1. Please ensure that your manuscript meets PLOS ONE's style requirements, including those for file naming. The PLOS ONE style templates can be found at Response: We have followed these style requirements 2. Please note that funding information should not appear in any section or other areas of your manuscript. We will only publish funding information present in the Funding Statement section of the online submission form. Please remove any funding-related text from the manuscript.   Response: The funding statement has been removed from manuscript and is only in the Funding Statement.   3. When completing the data availability statement of the submission form, you indicated that you will make your data available on acceptance. We strongly recommend all authors decide on a data sharing plan before acceptance, as the process can be lengthy and hold up publication timelines. Please note that, though access restrictions are acceptable now, your entire data will need to be made freely accessible if your manuscript is accepted for publication. This policy applies to all data except where public deposition would breach compliance with the protocol approved by your research ethics board. If you are unable to adhere to our open data policy, please kindly revise your statement to explain your reasoning and we will seek the editor's input on an exemption. Please be assured that, once you have provided your new statement, the assessment of your exemption will not hold up the peer review process.   Response: We confirm that the data are freely accessible upon request at the LSHTM Data Compass repository (MENISCUS Trial quantitative survey data) line 518-522   4. Your ethics statement should only appear in the Methods section of your manuscript. If your ethics statement is written in any section besides the Methods, please move it to the Methods section and delete it from any other section. Please ensure that your ethics statement is included in your manuscript, as the ethics statement entered into the online submission form will not be published alongside your manuscript.   Response: Thank you for the guidance. The ethic statement has been moved to the Methods section (lines 238-242)   5. Please include a separate caption for each figure in your manuscript.   Response: We have two figures and provide captions in the manuscript (lines 233 and lines 258 respectively).   6. Please include captions for your Supporting Information files at the end of your manuscript, and update any in-text citations to match accordingly. Please see our Supporting Information guidelines for more information:   Response: We have added the caption for the Supporting Information file at the end of the manuscript (line 640) and its in-text citation has been updated on lines 333 and line 357   7. Please upload a copy of your study protocol that was approved by your ethics committee/IRB as a Supporting Information file. By the study protocol, we mean the complete and detailed plan for the conduct and analysis of the trial approved by the ethics committee/IRB. Please send this in the original language. If this is in a language other than English, please also provide a translation.   Response: The protocol has been uploaded as a supporting file   8. If the reviewer comments include a recommendation to cite specific previously published works, please review and evaluate these publications to determine whether they are relevant and should be cited. There is no requirement to cite these works unless the editor has indicated otherwise.   Response: There were no such comments Reviewer Comments to the Author  Reviewer #1:  The paper lacks clarity from the statistical perspective. The statistical strategy is poorly presented in this study. Section 10 of the supplemental protocol is well written with sample size justification and various analysis options per endpoints discussed.  Response: Thank you for this comment. We have substantially revised the section on statistical analysis (Section 2.7) to clarify how we followed a conceptual framework (Figure 1) to build the multivariable models. Specifically, this first describes the two |
| --- | --- |

|  | types of models used (mixed-effect negative binomial for school absenteeism, and mixed-effects linear regression for examination score) and then describes the conceptual framework and how the multivariable models were built for each level.  As this study was nested within the MENISCUS cluster-randomised controlled trial, we did not conduct a sample size calculation for this nested analysis.  When one starts reading the paper, the design and parallel arms not even introduced in the Abstract. One has to wait until section 2.2 and the investigators finally state that the trial was a parallel-arm, cluster-randomized controlled trial in 60 schools with schools randomized 1:1 to either immediate or delayed intervention delivery.  Response: We apologise for the confusion with the study design. We have clarified that this manuscript reports results from a secondary observational analysis using data from the published MENISCUS trial. It has been clarified in the abstract on lines 29-30, in the introduction on lines 114-119, and the methods on lines 130-144.   1. What is the control arm mentioned in the abstract?   Response: This is the control arm from the trial within which the current analyses are nested. We have clarified this in the abstract as follows “We conducted secondary observational analyses using data from a cluster-randomised trial of a menstrual health intervention in 60 Ugandan secondary schools. We used baseline data from trial participants in both arms, and endline data from the control arm participants. “(lines 29- 30). We also now include a study flowchart (Figure 2) to help clarify this.   2. Also, the actual longitudinal input is not clear to the reader. Please relate immediate, delayed, and endline in a longitudinal context with respect to Figure 1.   Response: We thank the reviewer for this comment, and have removed the word ‘longitudinal’ throughout the manuscript. Instead, we describe the study as a prospective study in the title, and refer to baseline and endline data analysis in the abstract and manuscript (e.g. lines 29-30, 43-45 in the abstract), including in Figures 1 and the new Figure 2 which is the study flowchart.   3. In the Conclusion the authors note that among Ugandan students, multiple dimensions of menstrual health are associated with school absenteeism and educational performance. Where is the analysis of immediate or delayed intervention or is that not part of this report?   Response: Thank you for the comment. We have clarified in the Introduction section of the manuscript that this manuscript reports secondary data analysis of the trial, and that the intervention effects have been published previously (lines 103-113). We have also clarified that the estimate of the magnitude of effects in this paper help explain the lack of intervention effect (lines 380-383) “Through this observational analysis, we have shown that dimensions of menstrual health are associated with educational outcomes, but the absolute effect was small, which may help explain why the intervention was not sufficient to improve these educational outcomes.”  4 It looks like Tables 2 to 4 are baseline and Table 5 are endline results from the longitudinal approach? Is that correct?  Response: Yes that is correct. We apologise for an error in title of Table 5 which suggested the outcomes were at baseline (line 361).   5. Some sentences are vague. For example ,please explain line 292, ’ After adjusting for factors at the same or more distal levels, there was strong evidence that menstrual- related absenteeism and poorer examination performance were both associated…etc’. What does 'distal level' mean? Please be exact when you mention adjustment throughout the presentation.   Response: Thank you for this comment. We have revised the manuscript throughout to improve clarity. This includes rewriting Section 2.7 on Statistical Analyses to clarify the model building strategy . This includes definition of ‘distal level’ (to say “We use the term “distal” to refer to variables hypothesized to causally precede and influence variables at more proximal levels” (lines 209-220).   6. The entire presentation has to be edited for organization and clarity.   Response: We thank the reviewer for this comment and have edited the manuscript throughout to improve clarify.  Reviewer #2: |
| --- | --- |

|  | 1. Abstract    - Clearly explain how factors such as inadequate menstrual materials, negative attitudes toward menstruation, unmet menstrual practice needs, and experiences of menstrual teasing influence educational performance, not just absenteeism. Response: We had included this in the discussion but have moved to the introduction (lines 55-59).“Poor menstrual health is prevalent in low- and middle-income countries (LMICs), and may affect educational outcomes such as school absenteeism and examination performance through multiple pathways, including through shame, anxiety, lack of confidence, behavioural expectations, poor physical environments, and inadequate menstrual products”   Also clarify what is meant by “multiple dimensions of menstrual health” and how these are associated with both absenteeism and educational performance (may be in the body).  Response: We have defined the dimensions of menstrual health in the introduction (lines 70-73) “These dimensions include physical, behavioural and social components such as inadequate menstrual materials, menstrual pain, poor menstrual knowledge and confidence, and inadequate infrastructure for managing menstruation in schools”   - Article type: Why is this described as a clinical trial? Since the study used secondary data and so sign of any medical test. Please justify the article type or indicate this clearly in the methods section.   Response: Thank you for the comment. We have clarified in the manuscript that the paper is a secondary analysis using data from a cluster-randomised controlled trial (lines 123-124) and corrected this in the submission form.   2. Introduction    - Citations: Use square brackets for in-text citations instead of parentheses, to maintain consistency with journal style.   Response: Thank you. These have been changed to square brackets in line with journal style.   - Objective: Ensure the objective gives equal emphasis to both class absenteeism and educational performance. This balance appears lacking in the last part of the introduction. Response: We have revised the objective to include “examination performance” (line 114-117) and have revised the manuscript throughout to improve the balance. - MENISCUS: Define this acronym (e.g., “MENISCUS stands for...”) before using it, to help readers unfamiliar with the term.   Response: We have defined “MENISCUS” as “Menstrual health interventions, schooling, and mental health problems among Ugandan students (MENISCUS)” at first use on lines 112-113.   3. Methods    - Government vs private schools: What was the purpose of sampling both government and private schools if the outcomes are not contrasted by school type? Please clarify. Response: We have explained reasons for sampling both government and private schools on lines 156-158: “We over-sampled government schools as these are of interest to Ministry of Education stakeholders, and to increase generalisability of findings to these schools”.    - Interventions: Specify the interventions received by the treatment schools versus what the control schools received.   Response: We have added brief details of the intervention components for completeness on lines 137-140. We have also clarified that control schools were provided with a copy of the government guidelines on menstrual hygiene management and sexuality education and menstrual management reader.  One of the selection criteria mentioned was the presence of WASH services, suggesting there might not have been an intervention. Please clarify this point. If there were interventions, how they were managed and how frequent they were?  Response: We have clarified that we only use baseline trial data from the intervention arm in this paper, plus endline data from the control arm (lines 131-135) so there are no post-intervention data from the intervention arm. The WASH services mentioned were already present at the schools at baseline, and having “a minimum WASH facility” |
| --- | --- |

|  | was one of the trial eligibility criteria. We have clarified this on lines 148-155.   - Quality control: What methods were employed to minimize recall bias in reporting class absenteeism?   Response: We have revised the methods to clarify “To minimize recall bias, we used cognitive testing before the survey to ensure clearly worded questions anchored to specific time frames, and provided reminders of school terms and holidays during survey completion. During self-completion of the questionnaires, research assistants were present to assist participants with clarifications about the recall period and absence reasons.” Lines 192-196   - If the main results of MENISCUS have already been published, what new insights does this data analysis provide? Clearly, highlight the novel contributions/how different is this finding?   Response: We have revised the discussion to highlight the new insights provided by this analysis (lines 376-382). “Through this observational analysis, we have shown that multiple dimensions of menstrual health are associated with educational outcomes, but the absolute effect was small, which may explain why the intervention was not sufficient to improve these educational outcomes”.   - Why did you use both cross-sectional and longitudinal designs? Are there sufficient indicators that support the longitudinal approach? It seems data were collected only at baseline, end line, and through student self-reports, with no evidence of continuous data collection.   Response: We chose to analyse prospective data in the control arm to address the possibility of reverse causality which could be in the baseline cross-sectional data. For example, it is possible that students with more absenteeism missed learning about how to manage their menstruation, and had poorer menstrual experiences. We chose to restrict this analysis to the control arm to assess this relationship in the absence of the MENISCUS intervention and hence make the results more generalisable. We have clarified this in the methods section (lines 227-231).  “To mitigate against the possibility of reverse causality, and to assess temporal associations of exposures with the outcomes, we also analysed associations of baseline exposures with endline outcomes in the control arm, using analogous methods. We restricted these analyses to participants in the control arm as the MENISCUS intervention may have modified the association.”  4.  Results   - Findings on the effects of menstrual health on educational performance are limited and not strongly demonstrated. Consider discussing this limitation.   Response: Thank you for your comment. We have improved the discussion for example line 434-436 “However, overall, associations of dimensions of menstrual health with examination scores were weaker than with school absenteeism. This is as expected given that absenteeism is more proximal to poor menstrual health than educational performance”  We have revised the limitations section to note that these findings are modest and should be interpreted cautiously due to potential limitations such as residual confounding and limited sensitivity of educational measures (Lines 458-462). Also future research with longer follow-up, larger sample sizes, more sensitive and diverse academic outcome measures, and designs that better isolate menstrual health effects from other educational influences is needed to fully understand the impact of menstrual health on academic achievement (lines 479-482)  General comments   - Clearly distinguish between this study and the original MENISCUS study to avoid confusion.   Response: Thank you for the comment. We have revised the paper throughout to clarify that this paper uses baseline data from the trial, plus endline data in the control arm, and does not estimate intervention effects on the educational outcomes as these were presented in the trial paper. This is highlighted in the introduction (lines 114-117), methods (lines 131-135), and discussion (lines 379-382).   - Explicitly state the specific menstrual-related factors analyzed, instead of repeatedly |
| --- | --- |

|  | using the vague term “multiple dimensions.” This will make your findings clearer and more impactful.  Response: Thank for the guidance. We have edited the paper to specify the menstrual- related factors throughout the manuscript.   - Follow PLOS ONE reference format   Response: We apologise for this oversight. The citations are now in the PLoS One format.  Reviewer #3:  The authors have presented an intelligble fashion and the grammar is standard. The author should look at the punctuation and the tenses. The methodolgy is okay and the it is technically sound. The authour should avoid being too wordy. Some paragraphs are too long. He should make them brief and to the point.  Response: We thank the reviewer for their comments, and have revised the manuscript throughout for clarity, including shortening sentences and paragraphs.  Reviewer #4:  Abstract  Overall the abstract needs a bit of work, context and clarification. Please provide a very brief (understanding the tight word count) description of the intervention in the methods (or background) to help orient the reader.  Response: We thank the reviewer for their comment and have edited the abstract, including clarification that this is secondary analysis of observation (baseline and endline) data from cluster randomized trial (line 29-30). We prefer not to include a description of the intervention in the abstract as we are not using any post-intervention data in this analysis.  39: Do you mean “we analysed baseline and endline data”?  Response: Thank you for this correction. We have edited the abstract as suggested (line 31-32).  The results section needs tailoring.  47: At baseline 3312 participants reported menstruating in the last 6 months. 3312 of how many? Or are you trying to say that your sample was the 3312 participants who reported menstruating in the last 6 months. Please clarify or consider revising: “Of the 3312 participants who reported menstruating in the past 6-months at baseline, 323 (9.8%) reported missing at least one day of school per month due to menstruation.” Response: We have edited the sentence as suggested (line 39-41)  49: 1286 (38.8%) missed at least one day a month for any reason. Is this relevant? Is this key to be reported in the abstract? Without context it lacks definition of why it is relevant.  Response: We have removed this sentence from the abstract.  50: Of the 1192 participants in the control arm seen at endline, 135 (11.3%) missed at 51 least one day due to menstruation. Why is the control endline reported, and not the intervention arm endline? I don’t understand the relevance of reporting the control endline school absenteeism without the intervention data. What are you trying to say here? Can you add some clarifying text or otherwise provide some other data to clarify why this has been reported.  Response: We clarified that we are reporting results for a secondary data of observation analysis (line 29-30) and clarified our rationale for not including intervention arm participants in the endline data analysis in the methods section, also this is not an evaluation analysis (lines 227-231) “To mitigate against the possibility of reverse causality, and to assess temporal associations of exposures with the outcomes, we also analysed associations of baseline exposures with endline outcomes in the control arm, using analogous methods. We restricted these analyses to participants in the control arm in the MENISCUS intervention which may have moderated the association”  53: associated with multiple dimensions of menstrual health. Multiple dimensions of |
| --- | --- |

|  | poor menstrual health. The bracketed examples are the inverse of the dimensions on the conceptual model (described later). Potentially refer to the model here, saying “as per a conceptual model described in the paper” - or something to orient a reader who is not familiar with the model. Another way might be to use the phrasing as per the introduction “aspects of menstrual experience”. Perhaps instead of brackets, change to such as…  Response: We thank the reviewer for this suggestion. We have removed the wording about ‘dimensions’ of menstrual health in the results section in favour of specific menstrual practices, although we retain it in the conclusion to summarise the findings (lines 43-46). “There was evidence that menstrual-related absenteeism and poorer examination performance at endline were both associated with use of inadequate menstrual materials, negative menstrual attitudes, unmet menstrual practice needs, and experience of menstrual-related teasing”.  55: Menstrual teasing. Please use term menstrual-related teasing. “Menstrual teasing” is not correct (and not used consistently throughout paper). Note: Line 306: “menstrual teasing” and Line 324 should be edited for consistency.  Response: We have corrected this throughout the manuscript. Introduction  71: A widely-cited statistic that “one in 10 school-age girls in Africa misses school or drops out for reasons related to her period” is not evidence-based (3, 4). I get what you are trying to say here, but it reads strangely to me. Consider rephrasing. “One in ten school-age girls in Africa missed school or drops out for reasons related to her period” is a widely –cited statistic that lacks scientific backing.  Response: Thank you for the suggestion. We have modified the sentence to “The statement "one in 10 school-age girls in Africa misses school or drops out for reasons related to her period" is a widely-used statistic, but it is not supported by scientific research [3, 4]. “ (lines 65-66)  74: Consider: drop female – redundant. An average of 31%? Response: We have edited as suggested (line 68).  75: Edit: Far too long sentence. Use full-stop after longer duration, then begin sentence with Additionally, the review found menstruation also affected...  Response : Thank you, the sentence has been revised and edited (lines 70-71). 89: Consider changing knowledge to evidence or data.  Response: We have revised as suggested (line 80).  96: LMICs  Response: We have revised as suggested (line 81).  99: Change to: ...with academic performance a reported consequence of menstruation, mainly due to menstrual pain or heavy menstrual bleeding.  Response: We have revised this as “A commonly-reported reason for menstrual- related absenteeism is menstrual pain, which can also contribute to reduced participation, concentration, and academic performance” (line 89-90).  102: Broader educational issues are written here as self-esteem, school participation, and educational performance. How is educational performance difference from academic performance? Above has been discussing academic performance, please clarify how or if educational performance is different and thus belongs to “broader educational issues.”  Response: We have clarified the paper throughout to refer to educational outcomes as both school absenteeism and examination performance, and have replaced ‘academic performance’ and ‘educational performance’ throughout with either ‘educational outcomes’ or ‘examination performance’ as appropriate.  102/103: Should this sentence: “In the review among university students...”come above the sentence “There is relatively little and inconclusive evidence..” Im confused as to why this sentence separates the following comment on the review. Please edit or justify.  Response: We thank the reviewer for the comment, and have edited as suggested. |
| --- | --- |

|  | 109: You introduce us to: “Our recent cluster-randomised trial” is this the same cluster- randomised trail described in the methods? Aka the MENISCUS? If so, introduce it here not below. Please clarify or change the use of “our.” This is confusing.  Response: Yes it’s the same, we have clarified this as follows: “Our recent cluster- randomised trial, the MENISCUS study (“Menstrual health interventions, schooling, and mental health problems among Ugandan students”) showed strong evidence that a multi-component menstrual health intervention improved multiple dimensions of menstruation, including knowledge about puberty and menstruation, use of effective pain management, menstrual self-efficacy, and reduced unmet menstrual needs, over a one-year period [15]. However, Lines 103-104.  111: Add: showed strong evidence of an intervention effect on multiple dimensions of menstrual health, such as....  Response: Revised as above. Line 105-108  Please detail what dimensions were effected, considering below you say little on school absence and nothing on examination performance, or school absence overall. Response: Revised, as above Line 108-113 “there was little evidence of an intervention effect on school absence (measured using prospectively-collected daily diaries) during menstruation (11.2% vs 13.5% days missed; adjusted odds ratio (aOR)=0.81, 95%CI 0.62, 1.05). There was also no evidence of an intervention effect on examination performance (adjusted mean difference (aMD)=0.06, 95%CI -0.12,  0.24) or on school absence overall (10.5% days missed in both arms).” Additionally, is examination performace to be understood as different to academic performance, or educational performance. Please standardize or define.  Response: As mentioned above, we have clarified the wording to be consistent in using examination performance as the UNEB examination, and ‘educational outcomes’ to mean both school absenteeism and examination performance. We have removed reference to academic performance and educational performance.  117- 121: I suggest moving this last introductory paragraph to the top of the introduction to orient the reader and introduce MENISCUS.  After the first sentence introducing the MENISCUS trial, please provide a brief line to describe what the trial is.  Response: We would prefer to first introduce the reader to the literature on menstrual health and educational outcomes before focusing on the specific data source for this study, the MENISCUS trial, but will follow the Editor’s guidance on this.  The last sentence in this paragraph has a typo. Do you mean “add to the knowledge- base" or “add to the existing evidence on the impact of..” I would also advise revising the sentence to read: “based on the impact of multiple dimensions of menstrual health..” or “based on the impact of menstruation..”  Response: We have revised this sentence as follows : “In this paper, we provide new insights by quantifying the associations of menstrual health experiences (including pain management, social support, menstrual practices, and attitudes) with number of days of school absenteeism and examination performance“(lines 117-120).  Methods  124: Add “The MENSISCUS trial/study” Response: Revised (line 123)  132: Be consistent with abstract ie: baseline and endline. Response: We have clarified this throughout to be consistent.  132: This should not be the first time you are spelling out the acronym for MENISCUS. Please introduce in the introduction as suggested and spell out on first use.  Response: The acronym has been included in the introduction (line 103-104).  142-143: Decapitalise Education Institutions. Response: This has been corrected line 148  143-148: This sentence is too long. Full-stop at 146 and then start sentence with “School enrolment was to be estimated as. ” |
| --- | --- |

|  | Response: This has been corrected.  148: When you say “we” here, I am confused if you are talking about MENISCUS or your analysis of the trial? There needs to be clearer description of how what you are doing is related to MENISCUS and if you referring to it as “yours” etc.  Response: We apologise for the confusion. The sampling was done once, for the trial, by the same team that conducted the analysis for the current manuscript. We have removed the word ‘we’ here as follows: A random sample of 60 schools were selected after being confirmed as eligible and willing to participate. Line 155-156  151: Provide explanation of Secondary 2 in parenthesis as above for School 1. Response: We have clarified this as “Secondary 2 (second year of secondary school)” (line 160)  166-167: Decapitalise subject names (Mathematics, Biology, English Language) (English remains capitalised- but not language)  Response: We have corrected this (line 177-178)  187: Different reference style  Response: This has been corrected (line 209).  188: Here you use the term “Menstrual-related factors”. I am wondering if you mean the same when you talk about “dimensions of menstrual health or “aspects of menstrual experience.”  If so, could you please choose one, define it and standardize throughout the paper. Response: We have edited this (line 169-172) and elsewhere to be ‘dimensions of menstrual health’ which we have defined earlier (lines 61-64) as “multiple dimensions of menstrual health contribute to school absenteeism and reduced engagement [2]. These dimensions include physical, behavioural and social components such as inadequate menstrual materials, menstrual pain, poor menstrual knowledge and confidence, and inadequate infrastructure for managing menstruation in schools”  190: Delete full-stop after confounders.” Response: We have corrected this (line 210).  198: See 188  Response: Corrected  242: Describe/contextualise MPSN score. Is 2.11positive/negative?  Response: We have clarified as follows (lines 270-272). “The mean MPNS score of  2.11 (SD=0.52) corresponds to an average response of “sometimes” having unmet menstrual practice needs. The possible range is 0 to 3, with 3 corresponding to no unmet needs.”  Discussion:  355: “These results add..”  Response: We have corrected this (line 373)  365: Support – remove s  Response: We have revised the sentence to “Our finding that 10% of participants reported missing at least one school day per month due to menstruation supports the widespread claim that one in 10 girls miss school due to menstruation” (line 386-388)  378: Sentence need clarification - “but reported that other girls were absent from school due to menstruation” or “but reported that other girls reported absenteeism”? Response: We have clarified this: “but reported that other girls were absent from school due to menstruation” (line 403)  381-382: Perhaps belongs in strengths and limitations  Response: We prefer to retain this here as it is a recommendation, rather than a strength or limitation of our study |
| --- | --- |

|  | 386: Suggest without adequate (not with inadequate). Response: We have made this change (line 412)  392: Menstrual preparedness. Where is this definition of menstrual preparedness from? I don't see obtaining, using, cleaning and disposing of menstrual products and seeking assistance when needed as menstrual preparedness. Menstrual preparedness is yes about tracking cycle and body literacy. Or pre-menarche about prior knowledge, education and support  Response: We have clarified this by defining the menstrual preparation and Maintenance sub-scale as ‘menstrual preparedness” in Table 1, and referring to this Table in the sentence referred to by the reviewer (line 417-418).  SCREEN-IT review  Flow charts and attrition: We strongly recommend using flow charts because they provide an  overview of the study design and more information about attrition. Response: We have added the flowchart as Figure 2.  Blinding: Please specify whether blinding was used at various phases of the experiment (e.g., blinding of patients, caregivers, outcome assessments, data analysis).  Response: As this paper is not about intervention trial effects, we did not include this detail, but cite the main trial paper which includes details on blinding.  Please state how you determined the sample size for the study. If you performed a power or sample size calculation, please provide details  Response: As this study was nested within the MENISCUS cluster-randomised controlled trial, we did not conduct a sample size calculation for this nested analysis. |
| --- | --- |
| **Additional Information:** |  |
| **Question** | **Response** |
| **Financial Disclosure**  Enter a financial disclosure statement that describes the sources of funding for the work included in this submission. Review the [submission guidelines](http://journals.plos.org/plosone/s/submission-guidelines#loc-financial-disclosure-statement) for detailed requirements. View published research articles from [*PLOS ONE*](https://journals.plos.org/plosone/) for specific examples.  This statement is required for submission and **will appear in the published article** if the submission is accepted. Please make sure it is accurate. | Yes |
| **Funded studies**  Enter a statement with the following details:   - Initials of the authors who received each award - Grant numbers awarded to each author - The full name of each funder - URL of each funder website - Did the sponsors or funders play any role in the study design, data collection and analysis, decision to publish, or preparation of the manuscript? |  |
|  |  |

| Did you receive funding for this work? |  |
| --- | --- |
| Please add funding details.  as follow-up to "**Financial Disclosure**  Enter a financial disclosure statement that describes the sources of funding for the work included in this submission. Review the [submission guidelines](http://journals.plos.org/plosone/s/submission-guidelines#loc-financial-disclosure-statement) for detailed requirements. View published research articles from [*PLOS ONE*](https://journals.plos.org/plosone/) for specific examples.  This statement is required for submission and **will appear in the published article** if the submission is accepted. Please make sure it is accurate. | This work was supported by the Joint Global Health Scheme with funding from the UK Foreign, Commonwealth and Development Office, the UK Medical Research Council, the UK Department of Health and Social Care through the National Institute of Health Research (NIHR), and Wellcome (Grant Ref. MR/ V005634/1). The funder had no role in the design of the study; in the collection, analyses, or interpretation of the data; in the writing of the article; or in the decision to publish the results. |
| **Funded studies**  Enter a statement with the following details:   - Initials of the authors who received each award - Grant numbers awarded to each author - The full name of each funder - URL of each funder website - Did the sponsors or funders play any role in the study design, data collection and analysis, decision to publish, or preparation of the manuscript? |  |
| Did you receive funding for this work?" |  |
| Please select the country of your main research funder (please select carefully as in some cases this is used in fee calculation).  as follow-up to "**Financial Disclosure**  Enter a financial disclosure statement that describes the sources of funding for the work included in this submission. Review the [submission guidelines](http://journals.plos.org/plosone/s/submission-guidelines#loc-financial-disclosure-statement) for detailed requirements. View published research articles from [*PLOS ONE*](https://journals.plos.org/plosone/) for specific examples.  This statement is required for submission | UNITED KINGDOM - GB |

| and **will appear in the published article** if the submission is accepted. Please make sure it is accurate. |  |
| --- | --- |
| **Funded studies**  Enter a statement with the following details:   - Initials of the authors who received each award - Grant numbers awarded to each author - The full name of each funder - URL of each funder website - Did the sponsors or funders play any role in the study design, data collection and analysis, decision to publish, or preparation of the manuscript? |  |
| Did you receive funding for this work?" |  |
| **Competing Interests**  Use the instructions below to enter a competing interest statement for this submission. On behalf of all authors, disclose any [competing interests](http://journals.plos.org/plosone/s/competing-interests) that could be perceived to bias this work—acknowledging all financial support and any other relevant financial or non- financial competing interests.  This statement is required for submission and **will appear in the published article** if the submission is accepted. Please make sure it is accurate and that any funding sources listed in your Funding Information later in the submission form are also declared in your Financial Disclosure statement.  View published research articles from  [*PLOS ONE*](http://journals.plos.org/plosone/) for specific examples. | The authors declare that they have no competing interests. |

| **NO authors have competing interests**  Enter: *The authors have declared that no competing interests exist.*  **Authors with competing interests**  Enter competing interest details beginning with this statement:  *I have read the journal's policy and the authors of this manuscript have the following competing interests: [insert competing interests here]* |  |
| --- | --- |
| * typeset |  |
| **Ethics Statement**  Enter an ethics statement for this submission. This statement is required if the study involved:   - Human participants - Human specimens or tissue - Vertebrate animals or cephalopods - Vertebrate embryos or tissues - Field research   Write "N/A" if the submission does not require an ethics statement.  General guidance is provided below. Consult the [submission guidelines](http://journals.plos.org/plosone/s/submission-guidelines#loc-guidelines-for-specific-study-types) for detailed instructions. **Make sure that all information entered here is included in the Methods section of the manuscript.** | Ethical approval for MENISCUS protocol, the informed consent forms and draft CRFs were obtained from the Uganda Virus Research Institute Research & Ethics Committee (UVRI-REC) (reference GC/127/819) and the Uganda National Council of Science and Technology (UNCST) (reference HS1525ES) and the LSHTM Research Ethics Committee (reference 22952-2). |

| **Format for specific study types**  **Human Subject Research (involving human participants and/or tissue)**   - Give the name of the institutional review board or ethics committee that approved the study - Include the approval number and/or a statement indicating approval of this research - Indicate the form of consent obtained (written/oral) or the reason that consent was not obtained (e.g. the data were analyzed anonymously)   **Animal Research (involving vertebrate animals, embryos or tissues)**   - Provide the name of the Institutional Animal Care and Use Committee (IACUC) or other relevant ethics board that reviewed the study protocol, and indicate whether they approved this research or granted a formal waiver of ethical approval - Include an approval number if one was obtained - If the study involved *non-human primates*, add *additional details* about animal welfare and steps taken to ameliorate suffering - If anesthesia, euthanasia, or any kind of animal sacrifice is part of the study, include briefly which substances and/or methods were applied   **Field Research**  Include the following details if this study involves the collection of plant, animal, or other materials from a natural setting:   - Field permit number - Name of the institution or relevant body that granted permission |  |
| --- | --- |
|  |  |
| **Data Availability**  Authors are required to make all data underlying the findings described fully available, without restriction, and from the time of publication. PLOS allows rare exceptions to address legal and ethical concerns. See the [PLOS Data Policy](http://journals.plos.org/plosone/s/data-availability) and [FAQ](http://journals.plos.org/plosone/s/data-availability#loc-faqs-for-data-policy) for detailed information. | Yes - all data are fully available without restriction |

| A Data Availability Statement describing where the data can be found is required at submission. Your answers to this question constitute the Data Availability Statement and **will be published in the article**, if accepted. |  |
| --- | --- |
| **Important:** Stating ‘data available on request from the author’ is not sufficient. If your data are only available upon request, select ‘No’ for the first question and explain your exceptional situation in the text box. |  |
| Do the authors confirm that all data underlying the findings described in their manuscript are fully available without restriction? |  |
| **Describe where the data may be found in full sentences. If you are copying our sample text, replace any instances of XXX with the appropriate details.**   - If the data are **held or will be held in a public repository**, include URLs, accession numbers or DOIs. If this information will only be available after acceptance, indicate this by ticking the box below. For example: *All XXX files are available from the XXX database (accession number(s) XXX, XXX.*). - If the data are all contained **within the manuscript and/or Supporting Information files**, enter the following: *All relevant data are within the manuscript and its Supporting Information files.* - If neither of these applies but you are able to provide **details of access elsewhere**, with or without limitations, please do so. For example:   *Data cannot be shared publicly because of [XXX]. Data are available from the XXX Institutional Data Access / Ethics Committee (contact via XXX) for researchers who meet the criteria for access to confidential data.*  *The data underlying the results presented in the study are available from (include the name of the third party* | The data are held in a public repository here: LSHTM Data Compass  Weiss, H, Nelson, K, Mugenyi, L, Batuusa, L, Thomas, K and Baleke, C (2024). MENISCUS Trial. [Project]. London School of Hygiene & Tropical Medicine, London, United Kingdom. https://doi.org/10.17037/DATA.00003822. |

| *and contact information or URL).*   - This text is appropriate if the data are owned by a third party and authors do not have permission to share the data.   * typeset |  |
| --- | --- |
| Additional data availability information: | Tick here if the URLs/accession numbers/DOIs will be available only after acceptance of the manuscript for publication so that we can ensure their inclusion before publication. |

Manuscript	[Click here to access/download;Manuscript;Manuscript_clean](https://www2.cloud.editorialmanager.com/pone/download.aspx?id=42133019&guid=5525b042-776a-45d3-acf2-1e2537855aba&scheme=1)[<https://www2.cloud.editorialmanager.com/pone/download.aspx?id=42133019&guid=5525b042-776a-45d3-acf2-1e2537855aba&scheme=1> copy.docx](https://www2.cloud.editorialmanager.com/pone/download.aspx?id=42133019&guid=5525b042-776a-45d3-acf2-1e2537855aba&scheme=1)

1 **Title:** Associations of menstrual health with school absenteeism and examination
2 performance among Ugandan secondary school students: A prospective study
3 **Short title:** Menstrual health and educational outcomes among secondary school students in
4 Uganda
5 **Authors:** Christopher Baleke^a^ ^*^, Levicatus Mugenyi^a^, Kate A Nelson^b^, Katherine A Thomas^b^,
6 Denis Ndekezi^a^, Jonathan Reuben Enomut^a^, Connie Alezuyo^c^, John Jerrim^d^, Helen A Weiss^b^

# 7 Affiliations:

8 ^a^ MRC/UVRI and LSHTM Uganda Research Unit, PO Box 49, Entebbe, Plot 51-59
9 Nakiwogo Road, Uganda ([Christopher.baleke@lshtm.ac.uk](mailto:Christopher.baleke@lshtm.ac.uk);
10 [Levicatus.mugenyi@mrcuganda.org,](mailto:Levicatus.mugenyi@mrcuganda.org) [denis.ndekezi@mrcuganda.org,](mailto:denis.ndekezi@mrcuganda.org) [enomutj@gmail.com](mailto:enomutj@gmail.com))
11 ^b^ International Statistics and Epidemiology Group; Faculty of Epidemiology and Population
12 Health, London School of Hygiene & Tropical Medicine, Keppel Street, London, WC1E
13 7HT,	United	Kingdom	([kate.nelson@lshtm.ac.uk](mailto:kate.nelson@lshtm.ac.uk);	[katherine.thomas1@lshtm.ac.uk](mailto:katherine.thomas1@lshtm.ac.uk);
14 [helen.weiss@lshtm.ac.uk](mailto:helen.weiss@lshtm.ac.uk) )
15 ^c^ Ministry of Education and Sports, Uganda, P. O. BOX 7063 Kampala, Uganda King George
16 VI Way, Embassy House, Uganda ([calezuyo@gmail.com](mailto:calezuyo@gmail.com) )
17 ^d^ UCL Institute of Education, University College London, 20 Bedford Way, London WC1H
18 0AL, United Kingdom ([j.jerrim@ucl.ac.uk](mailto:j.jerrim@ucl.ac.uk) )
19 *Corresponding author email: [Christopher.baleke@lshtm.ac.uk](mailto:Christopher.baleke@lshtm.ac.uk)
20 This study is conducted under a MENISCUS with **Trial Registration and Recruitment**
21 **Details:	Registry	Name:** ISRCTN**;	Registration	Number:** ISRCTN45461276**;**
22 **DOI:** <https://doi.org/10.1186/ISRCTN45461276>
23 The trial paper has been published (doi:	10.1016/S2214-109X(25)00007-5**)** with its
24 CONSORT checklist

25

# 26 ABSTRACT

27 **Background:** Relatively few studies have quantified the amount of school missed due to
28 poor menstrual health, or the impact of poor menstrual health on examination performance.
29 **Methods:** We conducted secondary observational analyses from data nested within a cluster-
30 randomised trial of a menstrual health intervention in 60 Ugandan secondary schools (The
31 trial is registered as ISRCTN45461276). We used baseline data from trial participants in both
32 arms, and endline data from the control arm participants. School absenteeism was estimated
33 as the self-reported number of days absent due to menstruation per month and examination
34 performance was assessed by an independently set assessment by the Uganda National
35 Examination Board. We estimated adjusted incidence rate ratios (aIRR) for associations with
36 school absenteeism, using negative binomial regression adjusted for school-level clustering.
37 We estimated adjusted standardised mean differences (aSMD) in examination scores using
38 mixed-effects linear regression.
39 **Results:** Of the 3312 participants who reported menstruating in the past 6 months at baseline,
40 323 (9.8%) reported missing at least one day of school per month due to menstruation (mean
41 days missed=0.30 per month, 95%CI 0.27-0.34). Similarly, of the 1192 participants in the
42 trial control arm seen at endline, 135 (11.3%) reported missing at least one day due to
43 menstruation (mean days missed=0.31 per month (95%CI 0.27-0.37)). There was evidence
44 that menstrual-related absenteeism and poorer examination performance at endline were both
45 associated with baseline use of inadequate menstrual materials, negative menstrual attitudes,
46 unmet menstrual practice needs, and experience of menstrual-related teasing. In addition,
47 absenteeism due to menstruation was associated with menstrual pain, and poorer examination
48 performance was associated with poorer baseline menstrual knowledge.
49 **Conclusion:** Among Ugandan students, multiple dimensions of menstrual health are
50 associated with school absenteeism and examination performance.

# 51 Keywords

52 Menstrual health, school absenteeism, education outcomes

# 53 1. Introduction

54 Menstrual health (MH) is defined as complete physical, mental, and social wellbeing in
55 relation to the menstrual cycle [1]. Poor menstrual health is prevalent in low- and middle-
56 income countries (LMICs), and may affect educational outcomes such as school absenteeism
57 and examination performance through multiple pathways, including shame, anxiety,
58 lack of confidence, behavioural expectations, poor physical environments, and inadequate
59 menstrual products [2]. A review of 76 qualitative studies, which included 45 from sub-Saharan
60 Africa and 29 from South and East Asia, found that multiple dimensions of menstrual health
61 contributed to school absenteeism and reduced engagement [2]. These dimensions include
62 physical, behavioural and social components such as inadequate menstrual materials,
63 menstrual pain, poor menstrual knowledge and confidence, and inadequate infrastructure for
64 managing menstruation in schools [2].
65 The statement "one in 10 school-age girls in Africa misses school or drops out for reasons
66 related to her period" is a widely-used statistic, but is not supported by scientific research [3,
67 4]. A systematic review of 15 quantitative studies on school absenteeism during menstruation
68 in Africa found that an average of 31% (95%CI 24-39%) of students reported school
69 absenteeism during menstruation either at last menstrual period (LMP) or over a longer
70 duration [5]. The review found that menstruation also affected concentration, academic
71 performance, and participation in sports [5]. However, the number of school days missed
72 were not quantified and there was a high degree of unexplained heterogeneity between
73 studies (I^2^=97.8%), with prevalence of absenteeism due to menstrual pain ranging from 6% in
74 Ghana to 65% in Ethiopia. The variation in prevalence may be partly due to variation in the
75 reference period for measurement of school absenteeism, with some using LMP and others
76 using longer durations. A recent multi-country study of data from 673,380 women and girls
77 aged 15-49 in 47 countries found that the proportion reporting menstrual-related school
78 absenteeism during LMP was 15.0% (95%CI 12.7-17.3%), with the highest absenteeism
79 among 15-19 year olds (17.7%, 95%CI 15.1-20.3%) [6].
80 There is mixed evidence of an impact of menstrual health interventions on school
81 absenteeism. A recent systematic review identified nine relevant studies in LMICs, of which
82 six found evidence of a positive effect of an intervention on absenteeism [7]. However, the
83 three cluster-randomized control trials included in the review found no effect on absenteeism. Reasons for
84 the lack of evidence on the extent to which menstruation affects school attendance include a
85 lack of standardized and cross-validated absenteeism measures [8]. Additionally, these studies
86 lack details on whether absence was due to menstruation or non-menstrual related factors like
87 general illness or financial inadequacy to education, and the time period over which absence
88 was assessed [9].
89 A commonly-reported reason for menstrual-related absenteeism is menstrual pain, which can
90 also contribute to reduced participation, concentration, and academic performance [10]. A
91 systematic review of 38 studies (including 23 from LMICs) reported that 20.1% (95% CI
92 14.9–26.7) of adolescents and young women reported school or university absence due to
93 menstrual pain and 40.9% reporting an adverse effect on classroom performance or
94 concentration [11]. In these reviews, few studies provided details of how the educational
95 outcomes were measured, and none used extant data (official examination results and
96 attendance records) to validate self-reported measures [10]. There is relatively little, and
97 inconclusive evidence on the impact of menstruation on broader educational issues such as
98 self-esteem, participation in school and examination performance [10, 11]. Formative
99 research in Ugandan secondary school students has shown strong evidence that participants
100 missed school more frequently during menstruation than on other days, based on prospective
101 daily diaries (28.4% of schooldays were missed during menstruation compared with 6.5%
102 when not menstruating; p<0.001) [12].
103 Our	recently-published	MENISCUS	cluster-randomised	trial	(“Menstrual	health
104 interventions, schooling, and mental health problems among Ugandan students”) showed
105 strong evidence that a multi-component menstrual health intervention improved multiple
106 dimensions of menstruation, including knowledge about puberty and menstruation, use of
107 effective pain management, menstrual self-efficacy, and unmet menstrual needs, over a one-
108 year period [13]. However, the trial showed little evidence of an intervention effect on
109 educational outcomes. The proportion of school days missed during menstruation (measured
110 using prospectively collected daily diaries) was similar by arm (11.2% vs 13.5% in the
111 intervention vs control arms; adjusted odds ratio (aOR)=0.81, 95%CI 0.62, 1.05). Similarly,
112 there was no evidence of an impact of the intervention on examination performance (adjusted
113 mean difference (aSMD)=0.06, 95%CI -0.12, 0.24).
114 The aim of this paper is to conduct a secondary observational analysis using data from
115 MENISCUS trial [13, 14], to estimate associations of menstrual-related experiences with
116 menstrual-related school absenteeism and examination performance, among Ugandan
117 secondary school students. In this paper, we provide new insights by quantifying the
118 associations of menstrual health (including pain management, social support, menstrual
119 practices, and attitudes) with number of days of school absenteeism and examination
120 performance, respectively.

# 121 2. Methods

122 **2.1 Study Setting**
123 The MENISCUS trial was conducted in Kalungu and Wakiso districts of Uganda. Wakiso
124 district is the largest and largely urban district in central Uganda with an estimated population
125 of 3.4 million people [15] . In Wakiso, 17% of residents were aged 10-17 years, of whom
126 49% were attending secondary schools [16]. Kalungu is a rural district with population size
127 183,232 (2014 census). In Kalungu, 23% were aged 10-17 years, of whom 36% were enrolled

128

129

in secondary school [17].

# 130 2.2 Data source

131 This study is a secondary data analysis of data from the MENISCUS trial The trial aimed to
132 evaluate whether a multi-component menstrual health intervention improves education,
133 health and wellbeing among female secondary school students in Central Uganda [13]. In this
134 study, we report findings from secondary observational analyses of the baseline (both arms)
135 and endline (control arm only) trial data. Details of the trial design and results have been
136 published previously [13, 14]. In brief, the trial was a parallel-arm, cluster-randomised
137 controlled trial in 60 schools with schools randomised 1:1 to either the intervention package
138 or the control arm. A package of intervention components included puberty education, drama
139 skits, provision of menstrual health kits, training on pain management, and improvements to
140 WASH facilities. The schools randomised to the control arm were provided with a copy of
141 the government guidelines on menstrual hygiene management and sexuality education to the
142 headteacher, and Senior 2 students were given a copy of the government menstrual

143

144

management reader [14].

# 145 2.3 Sampling of schools

146 In preparation for the MENISCUS trial, all government schools and a random selection of
147 private schools were contacted through phone calls and assessed for eligibility using the
148 government’s 2019 master list of education institutions. Schools were eligible for the trial if
149 they were mixed-sex, secondary, day- or mixed day/boarding, and had at least minimum
150 water sanitation and hygiene (WASH) facilities (an improved water source and at least one
151 functional sex-specific toilet accessible to female students). Schools also had to have an
152 estimated size of 50-125 or 40-125 female students in Secondary 1 (first year of secondary
153 school) in Wakiso and Kalungu district, respectively. Schools were not eligible for the trial if
154 they were participating in a menstrual health related programme, were exclusively boarding
155 schools, or were single-sex schools. A random sample of 60 schools were selected after being
156 confirmed as eligible and willing to participate. We over-sampled government schools as
157 these are of interest to Ministry of Education stakeholders, and to increase generalizability of
158 findings to these schools.

# 159 2.4 Participant recruitment and informed consent/assent

160 All female students enrolled in Secondary 2 (i.e. the second year of secondary school) at the
161 start of the 2022 were eligible to participate in the MENISCUS trial. We sought school-level
162 written consent from headteachers and written informed consent from parents/guardians of
163 students aged <18 years, and from students aged ≥18 years. Students aged <18 years were

164

165

asked to assent if we had received parental consent.

# 166 2.5 Data collection

167 Baseline data were collected from 21 March to 5 July 2022 and endline data were collected
168 from 5 June to 22 August 2023. The surveys included items on sociodemographic
169 characteristics, mental health and multiple dimensions of menstrual health including physical
170 symptoms (e.g., pain), menstrual practices and materials, social experiences (e.g., teasing),
171 and menstrual knowledge and attitudes which were assessed alongside school absenteeism
172 due to menstruation (“menstrual-related absenteeism”) or due to other reasons. All were self-
173 completed using Open Data Kit software on tablets. The examinations were independently
174 administered and marked by the Uganda National Examination Board (UNEB), with
175 examinations administered on 16-17 March 2022 (baseline) and 24-26 July 2023 (endline).
176 Baseline examinations comprised materials taught in Secondary 1 and included four
177 mathematics and six biology questions, of which one question was on puberty and
178 reproduction. Endline examinations additionally included four English language questions,
179 and comprised materials taught in Secondary 1 and Secondary 2.

# 180 2.6 Study outcomes

181 Baseline menstrual-related absenteeism was assessed as self-reported number of school days
182 missed due to menstruation, excluding days where the main reason for missing school was
183 unrelated to menstruation (“Since schools reopened in January this year, how many days of
184 class did you miss during your period?” “What is the main reason for missing school during
185 your period?”). The days of school missed for any reason was obtained from the question
186 (“Since schools reopened in January this year, how many days of class did you miss for any
187 reason?”). We calculated the number of days in the denominator from the official date
188 schools re-opened post COVID-19 (10 January 2022) to the date of interview for each
189 participant, excluding school holidays. Endline menstrual-related absenteeism was assessed
190 as self-reported number of school days missed during menstruation in Terms 1 and 2 of 2023
191 until the interview date. Term 1 was from 6 February to 5 May 2023, and Term 2 started on
192 29 May 2023. To minimize recall bias, we used cognitive testing before the survey to ensure
193 clearly worded questions anchored to specific time frames, and provided reminders of school
194 terms and holidays during survey completion. During self-completion of the questionnaires,
195 research assistants were present to assist participants with clarifications about the recall
196 period and absence reasons. Baseline and endline examination scores were calculated as the

197

198

199

mean of subject-specific z-scores at each time point.

# 200 2.7 Statistical analyses

201 Data were exported to Stata version 18 for final cleaning and analysis. We estimated the
202 mean number of days missed overall per month and the mean number of days reported to be
203 missed due to menstruation using mixed-effects negative binomial regression models to
204 estimate adjusted incidence rate ratios (aIRR) and 95% confidence intervals (CI), allowing
205 for school-level clustering and over-dispersion. Similarly, we used mixed-effects linear
206 regression models to estimate adjusted standardized mean differences (aSMD) and 95%CIs
207 for examination score.
208 We built separate models for each group of exposure variables using a hierarchical
209 conceptual framework based on the integrated model of menstrual experience [2] to guide
210 adjustment for potential confounders (Figure 1). The dimensions of menstrual health are
211 defined in Table 1. In line with this framework, we grouped variables into six hierarchical
212 levels reflecting their conceptual proximity to the outcomes. We use the term “distal” to refer
213 to variables hypothesized to causally precede and influence variables at more proximal levels:
214 Level 1 (most distal - socio-demographic variables), Level 2 (social support during
215 menstruation), Level 3 (menstrual knowledge, attitudes and adequate menstrual product use),
216 Level 4 (menstrual pain management, menstrual practice needs, experience of menstrual-
217 related teasing), Level 5 (Self-efficacy in addressing menstrual needs scale [SAMNS] [20] -
218 menstrual care confidence with menstrual preparedness and pain management sub-scales) and
219 Level 6 (most proximal - trouble concentrating in class) (Figure 1).
220 We fitted multivariable models in which each variable was adjusted for other variables at the
221 same hierarchical level and for variables in more distal (upstream) levels, as defined in the
222 conceptual framework. An exception to this was the SAMNS sub-scales, which were not
223 adjusted for each other. We used Wald tests to assess the strength of evidence and refer to a
224 p-value<0.001 as indicating strong evidence of association [21]. We interpreted the results in
225 conjunction with effect sizes and confidence intervals to provide comprehensive assessment
226 of results.
227 To mitigate against the possibility of reverse causality, and to assess temporal associations of
228 exposures with the outcomes, we analysed associations of baseline exposures with endline
229 outcomes in the control arm, using analogous methods. We restricted these analyses to
230 participants in the control arm as the MENISCUS intervention may have modified the

231

232

association.

# 233 Figure 1: Conceptual framework for statistical analysis

234

# 235 Table 1: Definitions of menstrual-related exposure factors used in the conceptual framework

| **Level Survey measure** | | **Brief description** | **Calculation** | **Range & interpretation** |
| --- | --- | --- | --- | --- |
| 2 | Social support for menstruation | An individual has someone who they feel okay asking for support for your period if needed (for advice, resources, emotional support) | Binary | Binary (yes/no) |
| 3 | Adequate product use | Used at least one adequate menstrual material and no inadequate materials at LMP | Binary | Binary (yes/no) |
| 3 | Knowledge of puberty & menstruation | Factual knowledge about puberty, menstruation, and the menstrual cycle | Number of 9 knowledge questions answered correctly | 0-9  (number of questions answered correctly) |
| 3 | Attitudes towards menstruation | Attitudes about what it is okay for people to do while menstruating and myths about painkillers | Number of 3 attitude questions answered positively | 0-3  (number of questions answered positively) |
| 4 | Pain at LMP | Reported having any pain at LMP. This includes | Binary | Binary (yes/no) |

| reporting any pain, or a symptom of headache, backache, stomach ache or cramp at LMP. | | | | |
| --- | --- | --- | --- | --- |
| 4 | Effective pain management strategy | Effective pain strategies defined as stretching, painkillers, eating foods with lots of water, exercising, drinking lots of clean water, holding a warm water bottle on the stomach*.* Ineffective methods defined as: doing nothing, taking antibiotics, eating spicy foods, drinking soda | Using at least one effective method and no ineffective methods, among girls reporting pain at LMP. | Binary (yes/no) |
| 4 |  | | Weighted average of i) |  |
|  |  | | core items and school- | Range 0-3. A higher score |
|  | Menstrual Practice | The extent to which individuals’ menstrual |  |  |
|  |  | | specific items (75% | indicates fewer unmet |
|  | Needs Scale (MPNS- | management practices and environments were |  |  |
|  |  | | weight) and ii) relevant | menstrual needs (i.e. better |
|  | 36)[22] | perceived to meet their needs during their LMP. |  |  |
|  |  | | material-specific items | than a lower score). |
|  |  | | (25% weight). |  |
| 4 | Pain relief | The amount of pain reduced | Binary | All/most or some/none |

| 4 | Experience of menstrual-related teasing | Whether boys or girls tease the participant about their period | Binary | Binary (yes/no) |
| --- | --- | --- | --- | --- |
| 5 | Self-efficacy in addressing menstrual needs scale (SAMNS- 26) [20] | Individuals’ confidence in their capabilities to address their menstrual needs. This SAMNS scale has 3 sub-scales 1) The 17-item Menstrual Hygiene Preparation and Maintenance (MHPM) (“menstrual preparedness”) sub-scale includes items regarding self-efficacy in preparing for menstruation (e.g., tracking one’s cycle and anticipating days of bleeding), accomplishing various tasks related to maintaining menstrual hygiene (e.g., obtaining, using, cleaning, and disposing, menstrual materials in a variety of contexts), and seeking assistance when needed 2) the 4-item Executing Stigmatized Tasks  (EST) sub-scale, which also included items related to | Mean score | 0-100, representing the mean percentage confidence in addressing needs. Higher % is greater confidence |

| obtaining and disposing menstrual materials and seeking assistance—but in situations where accomplishing the tasks involves greater risk of revealing one’s menstrual status to a male person and  3) the 5-item Menstrual Pain Management (MPM), measured confidence in mitigating menstrual pain | | | | |
| --- | --- | --- | --- | --- |
| 6 | Trouble participating in class | Whether the participant had trouble participating in class due to period, during their most recent LMP at school | Binary | Binary (yes/no) |

236 **Ethical approval**
237 Ethical approval for MENISCUS protocol, the informed consent forms and draft CRFs were
238 obtained from the Uganda Virus Research Institute Research & Ethics Committee (UVRI-
239 REC) (reference GC/127/819) and the Uganda National Council of Science and Technology
240 (UNCST) (reference HS1525ES) and the LSHTM Research Ethics Committee (reference

241

242

22952-2).

# 243 Consent for publication

244 Participants consented to the research findings being published in international science
245 journals and electronic websites, on condition that that findings cannot be traced to

246

247

individuals.

# 248 3. RESULTS

249 Of the 4281 eligible participants, 3878 (90.5%) consented/assented and completed the
250 baseline survey (Figure 2). Of these, 3312 (79.1%) participants reported menstruating in the
251 past 6 months and were included in the baseline analyses on school absenteeism. A further
252 367 (11.1%) participants were not present for the baseline UNEB exam and were excluded
253 from baseline analyses of examination performance (Figure 2). Students who were missing
254 baseline education performance data were more likely to have greater unmet menstrual needs
255 (MPNS score, p=0.01) but there was no evidence of association with other dimensions of
256 menstrual health.

# 257 Figure 2: Study flowchart

258 **3.1 Baseline characteristics of study population**
259 The mean age of participants was 15.6 years (SD=0.9), with most (n=2161; 65.2%) attending
260 a privately-owned school. Around half (n=2070; 55.3%) were day students and had their
261 mother as the primary caregiver (n=1816; 54.8%). A total of 1227 (37.0%) participants lived
262 in households of ≥8 members and 571 (17.2%) reported having one or fewer meals the
263 previous day (Table 2). Overall, 2780 (83.9%) participants reported having social support
264 during their last menstrual period (LMP) and 3038 (91.7%) reported exclusive use of
265 adequate menstrual products at LMP (Table 3). A total of 1696 (51.2%) participants
266 answered at least 2 of the 3 attitude questions positively, and 538 (16.2%) answered at least 7
267 of the 9 menstrual knowledge questions correctly. Most participants (2446; 73.9%) reported
268 experiencing menstrual pain at last menstrual period, of whom 1529 (62.5%) reported using
269 an effective pain management strategy. The mean MPNS score of 2.11 (SD=0.52)
270 corresponds to on average response of “sometimes” having unmet menstrual practice needs.
271 The possible range is 0 to 3, with 3 corresponding to no unmet needs.
272 Additionally, participants have a moderate level of menstrual care confidence regarding
273 ability to manage menstrual needs (mean=60.8, SD=19.0). Overall, 251 (7.6%) and 284
274 (8.6%) participants reported menstrual-related teasing by boys and girls respectively, and
275 almost half of participants reported trouble participating in class during periods (n=1414,
276 42.7%) (Table 3).
277 Participants reported missing a mean of 1.25 school days per month overall (95%CI 1.17,
278 1.33), and 0.30 days due to menstruation (95%CI 0.27, 0.34). A total of 991 (30%)
279 participants reporting missing school due to menstruation since schools reopened, with 323

280

281

(9.8%) reporting missing at least one day per month due to menstruation.

# 282 3.2 Socio-demographic factors associated with menstrual-related absenteeism and

283 **examination performance at baseline**
284 Menstrual-related absenteeism and poorer examination performance were both associated
285 with being in a school with a poorer examination performance (aIRR=1.26, 95%CI 1.00,
286 1.60; aSMD=-0.44, 95%CI -0.57, -0.32), being a day student (aIRR=1.45, 95%CI 1.20, 1.77;
287 aSMD=-0.12, 95%CI -0.18, -0.05), older age (aIRR-trend=1.20, 95%CI 1.09, 1.33; aSMD-
288 trend=-0.10, 95%CI -0.13, -0.06) and having fewer meals the previous day (aIRR-trend=1.24,
289 95%CI 1.09, 1.40; aSMD-trend=-0.06, 95%CI -0.10, -0.02) (Table 2). In addition, poorer
290 examination performance was associated with older age at first menstruation (aSMD-trend=-
291 0.05, 95%CI -0.08, -0.02) and higher education level of the primary caregiver (aSMD=-0.12,

292

293

-0.19, -0.05) (Table 2).

# 294 3.3 Menstrual factors associated with school absenteeism due to menstruation and

295 **poorer examination performance at baseline**
296 Among the 991 participants reporting missing school due to menstruation, 1023 days were
297 missed per month (mean 1.03 per month per participant (SE=0.04) (Table 4). The most
298 commonly-reported main reason was illness (back/stomach pain or cramps: n=588; 57.2% of
299 days missed; feeling generally unwell: n=170; 17.2% of days missed). Relatively few

# 300 Table 2. Socio-demographic characteristics associated with school absenteeism due to menstruation and examination performance at

301 **baseline**

302

| **Variable** | **Number of**  **participants** | **Days of school missed due**  **menstruation per month (n= 3312)** | | **to** | **Examination performance (n=2945)** | |
| --- | --- | --- | --- | --- | --- | --- |
|  | **Frequency**  **(%)** | **Mean day**  **(SE)** | **aIRR**  **(95%CI)^1^** | **Standardized**  **mean exam score (SE)** | | **aSMD (95%CI)^1^** |
| **Structural-level characteristics** | | | | | | |
| **District** |  |  | P=0.38 |  | | P=0.23 |
| Wakiso | 2600 (78.5) | 0.29 (0.02) | 1 | 0.03 (0.05) | | 0 |
| Kalungu | 712 (21.5) | 0.37 (0.03) | 1.13 (0.86, 1.50) | -0.12 (0.10) | | -0.09 (-0.23, 0.05) |
| **School-level characteristics** | | | | | | |
| **School ownership** |  |  | P=0.80 |  | | P=0.99 |
| Private | 2161 (65.2) | 0.27 (0.02) | 1 | 0.07 (0.06) | | 0 |
| Government | 1151 (34.8) | 0.38 (0.03) | 0.97 (0.74, 1.27) | -0.16 (0.05) | | 0.00 (-0.14, 0.14) |

| **School-mean combined UNEB Math + Biology score** | P=0.05 |  | P<0.001 |
| --- | --- | --- | --- |
| High UNEB 1983 (53.0) 0.25 (0.02) | 1 | 0.24 (0.05) | 0 |
| Low UNEB 1758 (47.0) 0.37 (0.02) | 1.26 (1.00, 1.60) | -0.24 (0.03) | -0.44 (-0.57, -0.32) |
| **Proportion of students boarding at school** | P=0.95 |  | P=0.84 |
| < 50% 1820 (55.0) 0.35 (0.02) | 1 | -0.10 (0.06) | 0 |
| >=50% 1492 (45.0) 0.26 (0.02) | 0.99 (0.77, 1.28) | 0.12 (0.05) | 0.01 (-0.12, 0.15) |
| **School size (female baseline participants per school)** | P=0.57 |  | P=0.24 |
| Greater or equal to 59 2258 (68.2) 0.30 (0.02) | 1 | 0.03 (0.06) | -0.07 (-0.20, 0.05) |
| Fewer than 59 1054 (31.8) 0.34 (0.02) | 0.93 (0.74, 1.18) | -0.04 (0.06) | 0 |
| **Student type** | P<0.001 |  | P=0.001 |
| Boarding 1496 (45.2) 0.24 (0.02) | 1 | 0.11 (0.03) | 0 |
| Day 1816 (54.8) 0.37 (0.02) | 1.45 (1.20, 1.77) | -0.07 (0.05) | -0.12 (-0.18, -0.05) |
| **Individual-level socio-demographic & economic factors** | | | |
| **Age group** | P-trend=0.004 |  | P-trend=0.003 |
| <15 283 (8.5) 0.22 (0.03) | 1 | 0.19 (0.07) | 0.00 |
| 15 1353 (40.9) 0.25 (0.02) | 1.15 (0.83, 1.60) | 0.11 (0.05) | -0.02 (-0.13, 0.08) |

| 16 | 1234 (37.3) | 0.36 (0.02) | 1.54 (1.10, 2.15) | -0.08 (0.04) | -0.17 (-0.28, -0.06) |
| --- | --- | --- | --- | --- | --- |
| 17 | 341 (10.3) | 0.38 (0.05) | 1.66 (1.11, 2.49) | -0.14 (0.06) | -0.23 (-0.36, -0.09) |
| 18+ | 101 (3.0) | 0.48 (0.12) | 1.71 (0.97, 3.03) | -0.35 (0.10) | -0.34 (-0.53, -0.15) |
| Trend |  |  | 1.20 (1.09, 1.33) |  | -0.10 (-0.13, -0.06) |
| **Age at first menstruation** |  |  | P=0.02 |  | P-trend=0.003 |
| <13 years | 758 (22.9) | 0.34 (0.03) | 1 | 0.09 (0.06) | 0.00 |
| 13 years | 1230 (37.1) | 0.24 (0.02) | 0.74 (0.59, 0.92) | 0.05 (0.05) | -0.01 (-0.08, 0.06) |
| 14 years | 1027 (31.0) | 0.34 (0.03) | 0.92 (0.73, 1.16) | -0.07 (0.05) | -0.08 (-0.16, -0.00) |
| >14 years | 297 (9.0) | 0.40 (0.05) | 1.02 (0.73, 1.42) | -0.20 (0.06) | -0.14 (-0.25, -0.03) |
| Trend |  |  | n/a |  | -0.05 (-0.08, -0.02) |
| **Religion** |  |  | P=0.002 |  | P=0.19 |
| Catholic | 1052 (31.8) | 0.28 (0.02) | 1 | -0.03 (0.05) | 0 |
| Protestant/SDA | 1285 (38.8) | 0.29 (0.02) | 1.09 (0.88, 1.35) | 0.03 (0.05) | 0.06 (-0.01, 0.13) |
| Muslim | 959 (29.0) | 0.36 (0.03) | 1.45 (1.14, 1.84) | -0.05 (0.05) | -0.01 (-0.09, 0.06) |
| None/Other | 16 (0.5) | 0.87 (0.44) | 3.78 (1.22, 11.74) | 0.03 (0.27) | 0.07 (-0.38, 0.53) |
| **Ethnicity** |  |  | P=0.54 |  | P=0.83 |

| Muganda | 2280 (68.8) | 0.32 (0.02) | 1 | 0.00 (0.05) | 0 |
| --- | --- | --- | --- | --- | --- |
| Non Muganda | 1032 (31.2) | 0.30 (0.02) | 0.94 (0.79, 1.14) | 0.02 (0.04) | 0.01 (-0.05, 0.07) |
| **Primary caregiver** | |  | P=0.17 |  | P=0.22 |
| Mother | 1938 (58.5) | 0.33 (0.02) | 1 | 0.00 (0.05) | 0 |
| Father | 805 (24.3) | 0.27 (0.02) | 0.85 (0.69, 1.05) | 0.06 (0.05) | 0.04 (-0.02, 0.11) |
| Self and others | 569 (17.2) | 0.29 (0.03) | 0.84 (0.67, 1.06) | -0.10 (0.05) | -0.03 (-0.11, 0.05) |
| **Education of primary caregiver** | |  | P=0.15 |  | P=0.001 |
| Secondary or more | 2215 (59.2) | 0.29 (0.02) | 1 | -0.03 (0.05) | 0 |
| Primary or less | 881 (23.6) | 0.38 (0.03) | 1.09 (0.88, 1.34) | 0.04 (0.06) | 0.12 (0.05, 0.19) |
| Don't know | 645 (17.2) | 0.28 (0.03) | 0.83 (0.66, 1.05) | 0.05 (0.05) | 0.09 (0.02, 0.17) |
| **Household size** | |  | P=0.19 |  | P=0.89 |
| 0-5 | 1001 (30.2) | 0.28 (0.02) | 1 | -0.007 (0.05) | -0.01 (-0.08, 0.05) |
| 6-7 | 1084 (32.7) | 0.31 (0.03) | 1.20 (0.97, 1.49) | -0.001 (0.05) | -0.01 (-0.08, 0.05) |
| >=8 | 1227 (37.0) | 0.34 (0.03) | 1.18 (0.95, 1.45) | 0.00 (0.04) | 0 |
| **Number of meals eaten on the previous day** | |  | P-trend=0.008 |  | P-trend=0.005 |
| Three or more | 1058 (31.9) | 0.24 (0.02) | 1 | 0.05 (0.05) | 0 |

| Two | 1683 (50.8) | 0.31 (0.02) | 1.20 (0.99, 1.46) | 0.001 (0.05) | -0.03 (-0.09, 0.04) |
| --- | --- | --- | --- | --- | --- |
| One or fewer | 571 (17.2) | 0.41 (0.04) | 1.53 (1.19, 1.97) | -0.08 (0.05) | -0.13 (-0.21, -0.04) |
| Trend |  |  | 1.24 (1.09, 1.40) |  | -0.06 (-0.10, -0.02) |
| **Socioeconomic status quintile** |  |  | P=0.02 |  | P=0.04 |
| Highest | 656 (19.8) | 0.24 (0.03) | 1 | 0.005 (0.06) | 0 |
| Medium-high | 684 (20.7) | 0.25 (0.03) | 0.91 (0.69, 1.19) | 0.14 (0.04) | 0.12 (0.04, 0.21) |
| Medium | 655 (19.8) | 0.29 (0.03) | 0.99 (0.75, 1.30) | 0.009 (0.06) | 0.04 (-0.05, 0.13) |
| Low-medium | 676 (20.4) | 0.30 (0.03) | 0.92 (0.69, 1.22) | -0.02 (0.05) | 0.03 (-0.06, 0.13) |
| Low | 641 (19.4) | 0.47 (0.04) | 1.35 (1.01, 1.81) | -0.06 (0.05) | 0.00 (-0.09, 0.10) |
| ^1^ Adjusted for all socio-demographic factors and school level clustering | | | | | |

303

304

305

306

# Table 3. Menstrual characteristics associated with school absenteeism due to menstruation and examination performance at baseline

| **Variable** | **Number**  **participants** | **of** | **Days of school missed due to**  **menstruation per month (n= 3312)** | | **Examination performance (n=2945)** | |
| --- | --- | --- | --- | --- | --- | --- |
|  | **Frequency (%)** | **Mean day (SE)** | | **aIRR (95%CI)^1^** | **Standardized mean exam**  **score (SE)** | **aSMD (95%CI)^1^** |
| **Level 2 – Menstrual support** | | | | | | |
| **Social support for menstruation** |  |  | | P=0.77 |  | P=0.28 |
| Yes | 2780 (83.9) | 0.30 (0.01) | | 1 | -0.007 (0.04) | 0 |
| No | 532 (16.1) | 0.35 (0.04) | | 1.03 (0.82, 1.31) | 0.03 (0.06) | 0.04 (-0.03, 0.12) |
| **Level 3 – Menstrual knowledge, attitudes and adequate product use** | | | | | | |
| **Adequate product use at LMP** |  |  | | P<0.001 |  | P=0.008 |
| Yes | 3038 (91.7) | 0.29 (0.02) | | 1 | 0.009 (0.04) | 0 |
| No | 274 (8.3) | 0.54 (0.07) | | 1.82 (1.37, 2.42) | -0.14 (0.06) | -0.13 (-0.23, -0.03) |
| **Attitudes towards menstruation** |  |  | | P<0.001 |  | P<0.001 |

| Positive (2-3) | 1696 (51.2) | 0.25 (0.02) | 1 | 0.10 (0.05) | 0 |
| --- | --- | --- | --- | --- | --- |
| Negative (0-1) | 1616 (48.8) | 0.37 (0.02) | 1.42 (1.21, 1.68) | -0.10 (0.05) | -0.15 (-0.21, -0.10) |
| **Knowledge of puberty and menstruation** | |  | P-trend=0.58 |  | P-trend<0.001 |
| High (7-9) | 538 (16.2) | 0.27 (0.04) | 1 | 0.34 (0.07) | 0 |
| Medium (4-6) | 2378 (71.8) | 0.31 (0.02) | 1.05 (0.83, 1.33) | -0.03 (0.04) | -0.32 (-0.40, -0.25) |
| Low (0-3) | 396 (12.0) | 0.39 (0.05) | 1.09 (0.79, 1.51) | -0.30 (0.05) | -0.55 (-0.66, -0.45) |
| Trend | |  | 1.06 (0.86, 1.30) |  | -0.28 (-0.33, -0.23) |
| **Level 4 – Menstrual pain, pain management and experience of menstrual-related teasing** | | | | | |
| **Menstrual pain at LMP** | |  | p<0.001 |  | P=0.04 |
| No | 866 (26.1) | 0.15 (0.02) | 1 | 0.02 (0.05) | 0 |
| Yes | 2446 (73.9) | 0.37 (0.02) | 2.12 (1.73-2.59) | 0.001 (0.05) | 0.06 (0.00, 0.13) |
| **Effective pain management strategy at LMP** | |  | P<0.001 |  | P=0.09 |
| No pain | 866 (26.1) | 0.15 (0.02) | 1 | 0.02 (0.05) | 0 |
| Pain and used at least one effective  management strategy | 1529 (46.2) | 0.35 (0.02) | 2.13 (1.72, 2.63) | -0.02 (0.04) | 0.08 (0.01, 0.14) |
| Pain and did not use at least one effective | 917 (27.7) | 0.39 (0.03) | 2.10 (1.66, 2.66) | -0.06 (0.04) | 0.05 (-0.03, 0.12) |

| strategy | | | | |
| --- | --- | --- | --- | --- |
| **Menstrual pain relief** |  | P<0.001 |  | P=0.13 |
| No pain 866 (26.1) | 0.15 (0.02) | 1 | 0.02 (0.05) | 0 |
| All/most pain relieved 983 (29.7) | 0.34 (0.03) | 2.00 (1.59, 2.51) | 0.01 (0.05) | 0.06 (-0.01, 0.13) |
| Some/none of pain relieved 1463 (44.2) | 0.38 (0.02) | 2.20 (1.78, 2.73) | -0.005 (0.04) | 0.07 (-0.00, 0.14) |
| **Menstrual Practice Needs Score (MPNS score)** |  | P-trend<0.001 |  | P-trend<0.001 |
| High - few unmet needs (2.38-3.00] 1116 (34.0) | 0.18 (0.02) | 1 | 0.12 (0.05) | 0 |
| Medium (1.88-2.38] 1091 (33.2) | 0.27 (0.02) | 1.41 (1.15, 1.73) | -0.03 (0.04) | -0.10 (-0.17, -0.04) |
| Low - many unmet needs (0.00-1.88] 1076 (32.8) | 0.48 (0.03) | 2.02 (1.63, 2.50) | -0.09 (0.05) | -0.14 (-0.21, -0.07) |
| Trend |  | 1.44 (1.29-1.57) |  | -0.07 (-0.11, -0.03) |
| **Experience of menstrual-related teasing by boys** |  | P=0.04 |  | P=0.18 |
| No 3061 (92.4) | 0.29 (0.01) | 1 | 0.01 (0.04) | 0 |
| Yes 251 (7.6) | 0.60 (0.08) | 1.38 (1.02, 1.88) | -0.20 (0.05) | -0.07 (-0.18, 0.03) |
| **Experience of menstrual-related teasing by girls** |  | P=0.07 |  | p<0.001 |
| No 3028 (91.4) | 0.29 (0.01) | 1 | 0.02 (0.04) | 0 |
| Yes 284 (8.6) | 0.53 (0.07) | 1.31 (0.98,1.75) | -0.30 (0.06) | -0.17 (-0.27, -0.07) |

| **Level 5 – Menstrual care confidence** | | | | | |
| --- | --- | --- | --- | --- | --- |
| **Menstrual care confidence (SAMNS score)** |  |  | P=0.01 |  | P=0.73 |
| High self-efficacy (69.62-100] | 1129 (34.1) | 0.25 (0.02) | 1 | 0.05 (0.05) | 0 |
| Medium (52.31-69.62] | 1102 (33.3) | 0.25 (0.02) | 0.88 (0.72, 1.08) | -0.02 (0.05) | -0.01 (-0.07, 0.06) |
| Low self-efficacy (0-52.31] | 1081 (32.6) | 0.43 (0.03) | 1.20 (0.98, 1.49) | -0.03 (0.05) | 0.02 (-0.05, 0.09) |
| **Menstrual preparedness sub-scale** |  |  | P-trend<0.001 |  | P=0.86 |
| High (76.47-100] | 1058 (31.9) | 0.21 (0.02) | 1 | 0.07 (0.05) | 0 |
| Medium (58.25-76.47]- | 1127 (34.0) | 0.26 (0.02) | 1.15 (0.93, 1.41) | 0.002 (0.05) | 0.01(-0.05, 0.08) |
| Low (0-58.25] | 1127 (34.0) | 0.45 (0.03) | 1.54 (1.24, 1.91) | -0.06 (0.05) | 0.00 (-0.08, 0.07) |
| Trend |  |  | 1.25 (1.12, 1.39) |  | n/a |
| **Pain management sub-scale** |  |  | P=0.49 |  | P=0.71 |
| High (82-100] | 923 (27.9) | 0.28 (0.03) | 1 | -0.001 (0.05) | 0 |
| Medium (50-82] | 1241 (37.5) | 0.28 (0.02) | 0.98 (0.80, 1.21) | 0.02 (0.05) | 0.02 (-0.04, 0.09) |
| Low (0-50] | 1148 (34.7) | 0.37 (0.03) | 1.10 (0.89, 1.36) | -0.02 (0.05) | 0.03 (-0.04, 0.10) |
| **Level 6 – Class participation and attendance at LMP** | | | | | |
| **Trouble participating in class during LMP** |  |  | P<0.001 |  | P=0.91 |

| No | 1898 (57.3%) | 0.20 (0.01) | 1 | 0.007 (0.04) | 0 |
| --- | --- | --- | --- | --- | --- |
| Yes | 1414 (42.7%) | 0.46 (0.03) | 1.80 (1.51, 2.14) | -0.01 (0.05) | 0.00 (-0.06, 0.06) |
| **School absenteeism due to menstruation** |  |  |  |  | P=0.01 |
| Missed less than one day per month | n/a | n/a | n/a | 0.14 (0.04) | 0 |
| Missed at least one day per month | n/a | n/a | n/a | -0.20 (0.05) | -0.12 (-0.22, -0.03) |
| ^1^ Adjusted for school level clustering, level 1 (socio-demographic) factors and variables at the same or more distal levels. SAMNS scores and  sub-scales are not adjusted for each other. Trouble participating, and school absenteeism are not adjusted for each other. | | | | | |

307

308

309

310

311

# Table 4: Main reason for missing school due to menstruation, reported at baseline

| **Main reason for missing school due to menstruation** | **Number of participants n (%)** | **Mean (SD) days per participant missed due to menstruation per month among those reporting**  **this reason** | **Total days missed due to menstruation per month with this as the main reason** | **Proportion of days missed due to menstruation per month with this as**  **the mains reason** |
| --- | --- | --- | --- | --- |
| **Total** | **991** | **1.03 (1.16)** | **1023** | **100%** |
| **Illness** | | | | |
| Back/stomach pain or cramps | 588 (59.3) | 1.00 (1.06) | 585 | 57.2% |
| Feeling generally unwell | 170 (17.2) | 0.98 (1.20) | 165 | 16.2% |
| **Stigma** | | | | |
| Scared of leaking blood on clothes | 59 (6.0) | 0.92 (0.95) | 54 | 5.3% |
| Not wanting to change pad or absorbents at school | 2 (0.2) | 2.49 (3.01) | 5 | 0.5% |
| Household members telling me not to go to school  during period | 12 (1.2) | 1.86 (2.38) | 22 | 2.2% |

| Worried that others may know I have my periods | 18 (1.8) | 0.79 (0.53) | 14 | 1.4% |
| --- | --- | --- | --- | --- |
| Scared of having to ask for help because of  periods | 6 (0.6) | 0.94 (0.72) | 6 | 0.5% |
| **Physical environment** | | | | |
| School toilets or latrines not clean | 34 (3.4) | 1.20 (1.28) | 41 | 4.0% |
| Lack of access to toilet paper at school | 4 (0.4) | 0.56 (0.24) | 2 | 0.2% |
| Lack of access to water and/or soap at school | 17 (1.7) | 1.34 (2.27) | 23 | 2.2% |
| Not enough privacy in school toilet/latrine | 34 (1.4) | 1.20 (1.28) | 41 | 4.0% |
| **Socio-economic** | | | | |
| Unable to afford pads or other absorbents | 35 | 1.50 (1.45) | 52 | 5.1% |
| Lack of knickers | 31 | 1.26 (1.35) | 39 | 4.8% |

312 participants reported other reasons (stigma-related, the physical environment or socio-
313 economic) as the main reason for missing school during menstruation (Table 4).
314 After adjusting for variables at the same hierarchical levels of the hierarchical framework,
315 there was strong evidence that menstrual-related absenteeism and poorer examination
316 performance were both associated with not exclusively using adequate menstrual products
317 (aIRR=1.82, 95%CI 1.37, 2.42; aSMD=-0.13, 95%CI -0.23, -0.03), negative menstrual
318 attitudes (aIRR=1.42, 95%CI 1.21, 1.68, aSMD=-0.15, 95%CI -0.21, -0.10), menstrual pain
319 at LMP (aIRR=2.12, 95%CI 1.73, 2.59; aSMD=0.06, 95%CI 0.00, 0.13), more unmet
320 menstrual practice needs (p-trend<0.001) and reported experience of menstrual-related
321 teasing at LMP by girls (aIRR=1.31, 95%CI 0.98, 1.75; aSMD=-0.17, 95%CI -0.27, -0.07)
322 (Table 3). In addition, menstrual-related absenteeism was associated with lack of perceived
323 pain relief at LMP (aIRR=2.20, 95%CI 1.78, 2.73 for minimal/no pain relieved vs no pain),
324 menstrual-related teasing by boys (aIRR=1.38, 95%CI 1.02, 1.88), poor menstrual care
325 confidence (aIRR=1.20, 95%CI 0.98, 1.49 for lowest vs highest quartile, p-trend=0.01),
326 especially due to lack of menstrual preparedness (aIRR=1.54, 95%CI 1.24, 1.91, p-
327 trend<0.001) and with trouble participating in class during LMP (aIRR=1.80, 95%CI 1.51,
328 2.14). In absolute terms, the differences were relatively small, for example, the mean days
329 missed per month among participants with no menstrual pain at LMP was 0.15 (SE 0.02),
330 compared with 0.37 (SE 0.02) among participants with pain at LMP. Similar factors were
331 associated with missing school overall (i.e. for any reason) (Table S1).
332 Poorer examination performance was also associated with school absenteeism (aSMD=-0.12,

333

334

95%CI -0.22, -0.03 for ≥1 vs <1 day missed per month due to menstruation).

# 335 3.4 Menstrual factors at baseline associated with menstrual-related absenteeism and

336 **poorer examination performance at endline.**
337 Among the 1652 participants in control arm schools at baseline, 1253 (75.8%) were in control
338 schools at endline and completed the endline survey (Figure 2). Of these, 1192 (95.1%)
339 reported having menstruated in the past 6 months and were included in endline analysis, and
340 1099 (92.2%) had examination data performance at endline (Figure 2).
341 Among these participants, 135 (11.3%) reported missing at least one day of school due to
342 menstruation, and the mean days missed was 0.31 per month (95%CI 0.27-0.37). Menstrual-
343 related absenteeism and poorer examination performance at endline were both associated
344 with using inadequate menstrual products at baseline (aIRR=1.76, 95%CI 0.95, 3.24;
345 aSMD=-0.19, 95%CI -0.34, -0.03), negative menstrual attitudes (aIRR=1.76, 95%CI 1.26,
346 2.46; aSMD=-0.16, 95%CI -0.24, -0.08), more unmet menstrual practice needs (aIRR=1.75,
347 95%CI 1.12-2.71; aSMD= -0.10, 95%CI -0.21 to 0.00) and experience of menstrual-related
348 teasing by girls (aIRR=1.89, 95%CI 1.06, 2.73; aSMD=-0.33, 95%CI -0.48, -0.18) (Table 5).
349 In addition, menstrual-related absenteeism was associated with baseline menstrual pain
350 (aIRR=3.08, 95%CI 2.04, 4.65), including if there was pain management but no pain relief
351 (aIRR=3.42, 95%CI 2.18, 5.36 vs no pain), and trouble participating in class during LMP
352 (aIRR=1.69, 95%CI 1.20, 2.38). Endline examination performance was additionally
353 associated with poorer baseline menstrual knowledge (aSMD=-0.44, 95%CI -0.60, -0.28)
354 (Table 5). Similar factors were associated with school absenteeism for any reason (including
355 menstruation) (Table S1). For example, the mean number of days missed due to menstrual
356 pain was 1.36 days per month, vs 0.88 among those without pain at LMP (aIRR=1.41, 95%CI
357 1.26-1.57).

# 358 Table 5. Baseline menstrual characteristics associated with endline school absenteeism due to menstruation and examination

359 **performance**

360

**Variable	Number	of**

**participants**

**Days	of	school	missed	due	to menstruation at endline (n= 1192)**

**Examination performance at endline (n=1099)**

|  | **Frequency (%)** | **Mean day (SE)** | **aIRR (95%CI)^1^** | **Standardized** | **aSMD (95%CI)^1^** |
| --- | --- | --- | --- | --- | --- |
|  |  |  |  | **mean exam score** |  |
|  |  |  |  | **(SE)** |  |
| **Level 2 – Menstrual support** |  |  |  |  |  |
| **Social support for menstruation** |  |  | P=0.18 |  | P=0.15 |
| Yes | 1010 (84.7) | 0.32 (0.03) | 1 | -0.05 (0.08) | 0 |
| No | 182 (15.3) | 0.25 (0.04) | 0.73 (0.45, 1.16) | -0.06 (0.09) | -0.09 (-0.03, 0.20) |
| **Level 3 – Menstrual knowledge, attitudes and adequate product use** | | | | | |
| **Adequate product use** |  |  | P=0.07 |  | P=0.01 |
| Yes | 1106 (92.8) | 0.30 (0.02) | 1 | -0.08 (0.08) | 0 |

| No | 86 (7.2) | 0.48 (0.12) | 1.76 (0.95, 3.24) | -0.23 (0.11) | -0.19 (-0.34, -0.03) |
| --- | --- | --- | --- | --- | --- |
| **Attitudes towards menstruation** |  |  | P<0.001 |  | P<0.001 |
| Positive (2-3) | 614 (51.5) | 0.23 (0.03) | 1 | 0.01 (0.08) | 0 |
| Negative (0-1) | 578 (48.5) | 0.40 (0.04) | 1.76 (1.26, 2.46) | -0.18 (0.08) | -0.16 (-0.24, -0.08) |
| **Knowledge of puberty and menstruation** P=0.72 P-trend<0.001 | | | | | |
| High (7-9) | 198 (16.6) | 0.26 (0.05) | 1 | 0.26 (0.09) | 0 |
| Medium (4-6) | 839 (70.4) | 0.32 (0.03) | 1.13 (0.72, 1.79) | -0.06 (0.07) | -0.22 (-0.33, -0.10) |
| Low (0-3) | 155 (13.0) | 0.36 (0.08) | 0.96 (0.50, 1.83) | -0.29 (0.09) | -0.44 (-0.60, -0.28) |
| Trend |  |  |  |  | -0.22 (-0.30, -0.14) |
| **Level 4 – Menstrual pain, pain management and experience of teasing** | | | | | |
| **Menstrual pain at LMP** |  |  | P<0.001 |  | P=0.68 |
| No pain | 305 (25.6) | 0.13 (0.04) | 1 | 0.03 (0.09) | 0 |
| Any pain | 887 (74.4) | 0.37 (0.03) | 3.08 (2.04, 4.65) | -0.07 (00.22, 0.09) | -0.01 (-0.11, 0.08) |
| **Effective pain management strategy** |  |  | P<0.001 |  | P=0.93 |
| No pain | 305 (25.6) | 0.13 (0.04) | 1 | 0.03 (0.09) | 0 |
| Pain and used at least one effective | 538 (45.1) | 0.39 (0.04) | 3.07 (1.98, 4.76) | -0.06 (0.09) | -0.01 (-0.11, 0.10) |

| management strategy |  | | | | | | | |
| --- | --- | --- | --- | --- | --- | --- | --- | --- |
| Pain and did not use  effective strategy | at | least | one | 349 (29.3) | 0.36 (0.05) | 3.09 (0.95, 2.15) | -0.07 (0.08) | -0.02 (-0.13, 0.09) |
| **Menstrual pain relief** |  |  |  |  |  | P<0.001 |  | P=0.64 |
| No pain |  |  |  | 305 (25.6) | 0.13 (0.04) | 1 | 0.03 (0.09) | 0 |
| All/most pain relieved |  |  |  | 371 (31.1) | 0.35 (0.05) | 2.72 (1.72, 4.29) | -0.11 (0.10) | -0.04 (-0.15, 0.07) |
| Some/none of pain relieved | | 516 (43.3) | | | 0.39 (0.03) | 3.42 (2.18, 5.36) | -0.04 (0.08) | 0.01 (-0.10, 0.11) |
| **Menstrual Practice Needs Score (MPNS score)** | | | | |  | P=0.005 |  | P=0.06 |
| High - few unmet needs (2.38-3.00] 445 (37.6) | | | | | 0.20 (0.03) | 1 | 0.05 (0.08) | 0 |
| Medium (1.88-2.38] 372 (31.4) | | | | | 0.35 (0.05) | 1.43 (0.95, 2.15) | -0.07 (0.09) | -0.07 (-0.17, 0.04) |
| Low - many unmet needs (0.00-1.88] 366 (30.9) | | | | | 0.42 (0.04) | 1.75 (1.12, 2.71) | -0.13 (0.08) | -0.10 (-0.21, 0.00) |
| **Experience of menstrual-related teasing by boys** | | | | |  | P=0.18 |  | P=0.25 |
| No 1098 (92.1) | | | | | 0.29 (0.02) | 1 | -0.04 (0.08) | 0 |
| Yes 94 (7.9) | | | | | 0.59 (0.12) | 1.50 (0.83, 2.73) | -0.16 (0.13) | 0.07 (-0.09, 0.22) |
| **Experience of menstrual-related teasing by girls** | | | | |  | P=0.03 |  | P<0.001 |
| No 1087 (91.2) | | | | | 0.29 (0.02) | 1 | -0.02 (0.08) | 0 |

Yes	105 (8.8)	0.56 (0.09)	1.89 (1.06, 3.37)	-0.35 (0.12)	-0.33 (-0.48, -0.18)

**Level 5 – Menstrual care confidence**

**Menstrual care confidence (SAMNS score)**	p-trend=0.40	P=0.55

| High self-efficacy (69.62-100) | 420 (35.2) | 0.28 (0.04) | 1 | 0.02 (0.09) | 0 |
| --- | --- | --- | --- | --- | --- |
| Medium (52.31-69.62) | 397 (33.3) | 0.30 (0.04) | 1.08 (0.71, 1.62) | -0.02 (0.09) | 0.02 (-0.08, 0.12) |
| Low (0-52.31) | 375 (31.5) | 0.36 (0.04) | 1.20 (0.78, 1.83) | -0.13 (0.08) | -0.04 (-0.15, 0.07) |
| Trend |  |  | 0.91 (0.74, 1.13) |  |  |

**Level 6 – Class participation and attendance at LMP**

**Trouble participating in class during LMP**	P<0.001	P=0.52

| No |  |  |  | 678 (56.9) | 0.20 (0.02) | 1 | -0.05 (0.08) | 0 |
| --- | --- | --- | --- | --- | --- | --- | --- | --- |
| Yes |  |  |  | 514 (43.1) | 0.47 (0.04) | 1.69 (1.20-2.38) | -0.06 (0.08) | 0.03 (-0.06, 0.12) |
| **School** | **absenteeism** | **due** | **to** |  |  |  |  |  |
| **menstruation** | | | n/a | | n/a | n/a |  | P=0.43 |
| Missed less than one day per month | | | n/a | | n/a | n/a | -0.04 (0.08) | 0 |
| Missed at least one day per month | | | n/a | | n/a | n/a | -0.20 (0.10) | -0.06 (-0.22, 0.09) |

^1^ Adjusted for school level clustering and baseline variables at Level 1 (socio-demographic) and at the same or more distal levels.

361

362

363

364

365 **4. DISCUSSION**
366 **4.1 Multiple dimensions of menstrual health are associated with school absenteeism and**
367 **poor examination performance**
368 In this setting, there is strong evidence that use of inadequate menstrual products, negative
369 attitudes towards menstruation, having many unmet menstrual needs, menstrual pain and
370 experience of menstrual-related teasing were associated with both increased menstrual-
371 related absenteeism and poorer examination performance. These results add to the limited
372 evidence quantifying both the amount of menstrual-related absenteeism, and the specific
373 dimensions of menstrual health associated with absenteeism and examination performance.
374 We have previously shown that a multi-component menstrual health intervention improved
375 dimensions of menstrual health including knowledge, attitudes, self-efficacy and pain
376 management, there was no evidence of an intervention effect on educational outcomes
377 (absenteeism and examination performance) [13]. Through this observational analysis, we
378 have shown that multiple dimensions of menstrual health are associated with educational
379 outcomes, but the absolute effect was small, which may help explain why the intervention

380

381

was not sufficient to improve these educational outcomes.

# 382 4.2 The impact of menstrual health on number of days of school missed due to

383 **menstruation**
384 Our finding that 10% of participants reported missing at least one school day per month due
385 to menstruation supports the widespread claim that one in 10 girls miss school due to
386 menstruation [3]. However, on average, participants reported missing less than half a day of
387 school per month due to menstruation (0.30 days). The prevalence of menstrual-related
388 absenteeism is at the lower end of the range seen in the systematic review of studies in Sub-
389 Saharan Africa (6-65% missing school during menstruation) [5], and slightly lower than the
390 recent multi-country study (17.7%, 95%CI 15.1-20.3%) among 15-19 year olds [6]. Reported
391 school absence may be lower in our study because i) students had missed almost two years of
392 school due to COVID-19 closures and may have felt pressure from teachers and parents to
393 make up the time; ii) reported days of school missed due to menstruation may be under-
394 reported by boarding students who interpreted being on school premises as attending school,
395 and iii) the relatively long period of reporting in our study may have led to underestimation
396 due to recall bias. The variation in estimates between studies is also likely due to challenges
397 measuring school attendance accurately [8]. Measuring school attendance using self-report
398 often underestimates absenteeism compared to using school registers [23], which may be due
399 to social desirability bias [8, 24], stigma and taboos around menstruation, and recall bias.
400 This is supported by qualitative findings from a Kenyan study, in which girls rarely reported
401 school absenteeism themselves, but reported that other girls were absent from school due to
402 menstruation [25]. However in some settings including Uganda, school registers are
403 incomplete and/or inaccurate, due to teachers not completing the registers, and social
404 desirability bias if school funding is linked with student numbers [23]. Future studies should
405 consider a combination of these methods, daily diaries and spot checks to triangulate data on
406 school attendance [23].
407 To our knowledge, this is the first study in sub-Saharan Africa to quantify days of school
408 absenteeism attributable to specific dimensions of menstrual health. We found that students
409 reporting menstrual pain had more than twice the rate of menstrual-related absenteeism than
410 those with no pain, and school absenteeism was highest among those without adequate pain
411 relief. Further, we found that menstrual pain or feeling generally unwell were responsible for
412 about three-quarters of days of school absenteeism due to menstruation. At baseline, there
413 was evidence that participants with low menstrual self-efficacy were more likely to miss
414 school due menstruation compared to those with high menstrual self-efficacy. The association
415 was strongest with the menstrual preparedness sub-scale on the menstrual self-efficacy scale
416 (Table 1). This fits with findings from qualitative studies that students with low menstrual
417 confidence might feel uncomfortable to be around students or teachers during menstruation
418 and hence choose to miss school during menstruation [9, 26]. This association may also be
419 due to girls fearing leakage of blood whilst in class and hence missing school [2].

# 420 4.3 The impact of menstrual health on examination performance

421 Qualitative studies have described how participants perceive that menstruation affects their
422 educational outcomes [2] but there are few rigorous quantitative studies on this. Our findings
423 broadly support the hypothesis that menstrual health affects educational outcomes through
424 multiple pathways, through menstrual pain or use of inadequate materials, but also due to
425 social stigma and lack of menstrual knowledge, and through school absenteeism. Using a
426 standardised assessment set by the UNEB, we found that poorer examination performance
427 was associated with use of inadequate products, negative attitudes towards menstruation,
428 having many unmet menstrual needs, menstrual pain and experience of menstrual-related
429 teasing. There was strong evidence of associations between poorer examination scores and
430 use of inadequate materials, poorer menstrual attitudes, poorer menstrual knowledge, having
431 more unmet menstrual practice needs and experience of being teased in relation to periods.
432 However, overall, associations of dimensions of menstrual health with examination scores
433 were weaker than with school absenteeism. This is as expected given that absenteeism is
434 more proximal to poor menstrual health than educational performance.
435 Our results support qualitative findings from the systematic review [2] and a qualitative study
436 of 120 girls aged 14-16 years in in Kenya where girls reported that their attention in class was
437 disturbed by worries of leakage of menstrual blood and staining of clothing [27]. A further
438 study found that students reported failing to stand up and answer questions, write on the
439 board and miss exams during menstruation [28]. Missing at least one day of school due to
440 menstruation at LMP at baseline was associated with poorer baseline examination scores, but
441 not with endline examination scores. This difference may be due to chance or due to the
442 longer temporal gap between missing school at baseline and examination performance at
443 endline. Persistent school absenteeism increases the risk of school dropout, lowers girls' self-
444 esteem, and compromises future productivity [29]. A strength of our study was the use of an
445 independent examination set by UNEB. We identified one previous study in Uganda which
446 evaluated academic performance similarly, using assessments in English language,
447 Mathematics, Integrated Science, and Social Studies set by UNEB [30] . In this small
448 randomised controlled trial of 60 participants in two primary schools in Uganda, a menstrual
449 health intervention (which included telling menstrual stories and playing games related to
450 menstrual management) was associated with improved academic scores at 6 weeks after the

451

452

intervention [30].

453 Overall, research on the effects of menstrual health on examination performance remains
454 limited hence scarce causal evidence [5]. But several other different forms of school
455 absenteeism due sickness, domestic violence have been are associated with academic
456 performance [31] but these maybe hard from separate from menstrual related absenteeism. In
457 our study, although we observed associations between menstrual health factors and
458 examination performance, these findings are modest and should be interpreted cautiously due
459 to potential limitations such as residual confounding, limited sensitivity of educational

460

461

measures.

# 462 4.4 Strengths and limitations of the study

463 Strengths of our study include the measurement of multiple dimensions of menstrual health;
464 use of both cross-sectional and temporal analyses (to minimise reverse causality);
465 measurement of reported number of schooldays missed due to menstruation over a defined
466 period; use of a self-completed questionnaire (to minimise social desirability bias); use of
467 validated tools for menstrual care confidence and unmet practice needs; and assessment of
468 examination performance using an examination set independently by UNEB, tailored to

469

470

material that had been taught across all 60 schools.

471 Limitations include possible recall bias from retrospective, self-reported outcome of school
472 attendance, residual confounding, and challenges related to the long duration (one year)
473 between baseline menstrual experiences and endline outcomes. The examination performance
474 outcome is objective but limited, especially at baseline, by the relatively sparse amount of
475 material that had been taught between schools re-opening after COVID-9 in January 2022,
476 and the assessment in March 2022. This resulted in a lack of discrimination between
477 potentially low- and high-achieving students. Future research with longer follow-up, larger
478 sample sizes, more sensitive and diverse educational outcome measures, and designs that
479 better isolate menstrual health effects from other educational influences is needed to fully

480

481

understand the impact of menstrual health on examination performance.

# 482 5.5 Conclusion and Recommendation

483 Multiple dimensions of menstrual health such as use of inadequate products, negative
484 attitudes towards menstruation, having many unmet menstrual needs, menstrual pain and
485 experience of menstrual-related teasing are strongly associated with school absenteeism and
486 subsequent poor education performance among female students in Ugandan secondary
487 schools, but the absolute effect size is small as menstrual-related absenteeism is infrequent.
488 This small effect size is a plausible explanation for why the MENISCUS intervention
489 improved menstrual health indicators, but had no impact on educational outcomes [13]. The
490 gap between improving menstrual health versus achieving tangible gains in educational
491 outcomes emphasizes the need for more tailored, multifaceted approaches that address not
492 only menstrual health but also broader contextual barriers to enhance educational outcomes.
493 Nevertheless, improving menstrual health remains a critical priority globally. Addressing this
494 issue is essential not only for safeguarding physical and psychological well-being but also
495 advancing gender equality and human rights issues.

496

# 497 Acknowledgements

498	We would like to thank the staff, students, and communities of participating schools for their

499	engagement with the study; the stakeholders from the Uganda Ministry of Education and

500

501

Sports; and District Education Officers in Wakiso and Kalungu districts.

502

503

# Abbreviations

504 MH: Menstrual health; LMP: Last menstrual period; CI: Confidence interval; LRT:
505 Likelihood ratio test; SMD: Standardized mean difference; IRR: Incidence rate; MENISCUS:
506 Menstrual health interventions, schooling, and mental health problems among Ugandan
507 students; MHM: Menstrual hygiene management; SES: Socio-economic status; WASH:
508 Water, sanitation and hygiene; SAMNS: Self-efficacy in Addressing Menstrual Needs Scale;
509 MHPM: Menstrual Hygiene Preparation and Maintenance sub-scale; MPM: Menstrual Pain
510 Management sub-scale; EST: Executing Stigmatized Tasks sub-scale

511

512

513

# 514 Declarations

515 **Availability of data and materials**
516 The datasets used and/or analysed during the current study are available at LSHTM Data
517 Compass	and can be freely accessed on reasonable request. Details of location are;
518 MENISCUS Trial quantitative survey data. [Dataset]. London School of Hygiene & Tropical
519 Medicine,	London,	United	Kingdom.	10.17037/DATA.00003865	([MENISCUS	Trial](https://datacompass.lshtm.ac.uk/id/eprint/3865/)
520 [quantitative survey data](https://datacompass.lshtm.ac.uk/id/eprint/3865/) ).

# 521 Competing interests

522

523

524

525

526

527

528

529

530

531

532

533

534

535

536

537

538

The authors declare that they have no competing interests.

# 539 References

540 1.	Hennegan J, Winkler IT, Bobel C, Keiser D, Hampton J, Larsson G, et al. Menstrual
541 health: a definition for policy, practice, and research. Sex Reprod Health Matters.
542 2021a;29(1):1911618.
543 2.	Hennegan J, Shannon AK, Rubli J, Schwab KJ, Melendez-Torres GJ. Women’s and
544 girls’ experiences of menstruation in low- and middle-income countries: A systematic review
545 and qualitative metasynthesis. PLOS Medicine. 2019;16(5):e1002803.
546 3.	Wilkinson K. No data shows 1 in 10 African girls miss school because of
547 menstruation South Africa: Africa Check; 2016 [Available from: [https://africacheck.org/fact-](https://africacheck.org/fact-checks/reports/no-data-shows-1-10-african-girls-miss-school-because-menstruation)
548 [checks/reports/no-data-shows-1-10-african-girls-miss-school-because-menstruation](https://africacheck.org/fact-checks/reports/no-data-shows-1-10-african-girls-miss-school-because-menstruation).
549 4.	Bobel C. The Managed Body: Developing Girls and Menstrual Health in the Global
550 South. Cham, Switzerland.: Palgrave Macmillan / Springer Nature Switzerland AG; 2018.
551 5.	Tadesse AG. Prevalence of school absenteeism during menstruation and associated
552 factors in sub-Saharan Africa: A systematic review and meta-analysis. Research Square.
553 2023.
554 6.	Starr M, Harding R, Ataíde R, Von Dinklage N, Sinharoy SS, Jayasinghe Y, et al.
555 Epidemiology of menstrual-related absenteeism in 44 low-income and middle-income
556 countries: a cross-sectional analysis of Multiple Indicator Cluster Surveys. The Lancet Global
557 Health. 2025;13(2):e285-e97.
558 7.	Betsu BD, Medhanyie AA, Gebrehiwet TG, Wall LL. Menstrual hygiene management
559 interventions and their effects on schoolgirls' menstrual hygiene experiences in low and
560 middle countries: A systematic review. PLoS One. 2024;19(8):e0302523.
561 8.	Benshaul-Tolonen A, Zulaika G, Sommer M, Phillips-Howard PA. Measuring
562 Menstruation-Related Absenteeism Among Adolescents in Low-Income Countries. In: Bobel
563 C, Winkler IT, Fahs B, Hasson KA, Kissling EA, Roberts T-A, editors. The Palgrave
564 Handbook of Critical Menstruation Studies. Singapore: Springer Singapore; 2020. p. 705-23.
565 9.	Hennegan J, OlaOlorun FM, Oumarou S, Alzouma S, Guiella G, Omoluabi E, et al.
566 School and work absenteeism due to menstruation in three West African countries: findings
567 from PMA2020 surveys. Sex Reprod Health Matters. 2021b;29(1):1915940.
568 10.	Munro AK, Hunter EC, Hossain SZ, Keep M. A systematic review of the menstrual
569 experiences of university students and the impacts on their education: A global perspective.
570 PLoS One. 2021;16(9):e0257333.
571 11.	Armour M, Parry K, Manohar N, Holmes K, Ferfolja T, Curry C, et al. The
572 Prevalence and Academic Impact of Dysmenorrhea in 21,573 Young Women: A Systematic
573 Review and Meta-Analysis. Journal of Women's Health. 2019;28(8):1161-71.
574 12.	Miiro G, Rutakumwa R, Nakiyingi-Miiro J, Nakuya K, Musoke S, Namakula J, et al.
575 Menstrual health and school absenteeism among adolescent girls in Uganda (MENISCUS): a
576 feasibility study. BMC Women's Health. 2018;18(1):4.
577 13.	Nelson KA, Lagony S, Kansiime C, Torondel B, Tanton C, Ndekezi D, et al. Effects
578 and costs of a multi-component menstrual health intervention (MENISCUS) on mental health
579 problems, educational performance, and menstrual health in Ugandan secondary schools: an
580 open-label, school-based, cluster-randomised controlled trial. Lancet Glob Health.
581 2025;13(5):e888-e99.
582 14.	Kansiime C, Hytti L, Nelson KA, Torondel B, Francis SC, Tanton C, et al. Menstrual
583 health interventions, schooling, and mental health problems among Ugandan students
584 (MENISCUS): study protocol for a school-based cluster-randomised trial. Trials.
585 2022;23(1):759.
586 15.	Statistics UBo. The National Population and Housing Census 2024 – Final Report -
587 Volume 1 (Main). Kampala, Uganda: Uganda Bureau of Statistics; 2024 DECEMBER 2024.
588 16.	UBOS. The national population and housing census 2014 – area specifc profle series -
589 Wakiso. Kampala: UBOS; 2017.
590 17.	UBOS. The National Population and Housing Census 2014 – Area
591 Specific Profile Series-Kalungu district. Kampala: UBOS; 2017.
592 18.	Ssemata AS, Ndekezi D, Kansiime C, Bakanoma R, Tanton C, Nelson KA, et al.
593 Understanding the social and physical menstrual health environment of secondary schools in
594 Uganda: A qualitative methods study. PLOS Global Public Health. 2023;3(11):e0002665.
595 19.	Nelson KA, Lagony S, Kansiime C, Torondel B, Tanton C, Ndekezi D, et al. Effects
596 and costs of a multi-component menstrual health intervention (MENISCUS) on mental health
597 problems, educational performance, and menstrual health in Ugandan secondary schools: an
598 open-label, school-based, cluster-randomised controlled trial. The Lancet Global Health.
599 2025;13(5):e888-e99.
600 20.	Hunter EC, Murray SM, Sultana F, Alam MU, Sarker S, Rahman M, et al.
601 Development and validation of the Self-Efficacy in Addressing Menstrual Needs Scale
602 (SAMNS-26) in Bangladeshi schools: A measure of girls' menstrual care confidence. PLoS
603 One. 2022;17(10):e0275736.
604 21.	Sterne JA, Davey Smith G. Sifting the evidence-what's wrong with significance tests?
605 Bmj. 2001;322(7280):226-31.
606 22.	Hennegan J, Nansubuga A, Smith C, Redshaw M, Akullo A, Schwab KJ. Measuring
607 menstrual hygiene experience: development and validation of the Menstrual Practice Needs
608 Scale (MPNS-36) in Soroti, Uganda. BMJ Open. 2020;10(2):e034461.
609 23.	Evans DK, Mendez Acosta A. How to measure student absenteeism in low- and
610 middle-income countries. Economics of Education Review. 2023;96:102454.
611 24.	Keppens G, Spruyt B, Dockx J. Measuring School Absenteeism: Administrative
612 Attendance Data Collected by Schools Differ From Self-Reports in Systematic Ways. Front
613 Psychol. 2019;10:2623.
614 25.	Mason L, Nyothach E, Alexander K, Odhiambo FO, Eleveld A, Vulule J, et al. 'We
615 keep it secret so no one should know'--a qualitative study to explore young schoolgirls
616 attitudes and experiences with menstruation in rural western Kenya. PLoS One.
617 2013;8(11):e79132.
618 26.	El-Gilany AH, Badawi K, El-Fedawy S. Menstrual hygiene among adolescent
619 schoolgirls in Mansoura, Egypt. Reprod Health Matters. 2005;13(26):147-52.
620 27.	Mason L, Nyothach E, Alexander K, Odhiambo FO, Eleveld A, Vulule J, et al. ‘We
621 Keep It Secret So No One Should Know’ – A Qualitative Study to Explore Young
622 Schoolgirls Attitudes and Experiences with Menstruation in Rural Western Kenya. PLOS
623 ONE. 2013;8(11):e79132.
624 28.	Tegegne TK, Sisay MM. Menstrual hygiene management and school absenteeism
625 among female adolescent students in Northeast Ethiopia. BMC Public Health.
626 2014;14(1):1118.
627 29.	Korpershoek H, T. CE, M. F-B, and de Boer H. The relationships between school
628 belonging and students’ motivational, social-emotional, behavioural, and academic outcomes
629 in secondary education: a meta-analytic review. Research Papers in Education.
630 2020;35(6):641-80.
631 30.	Topistar N, Dennis Zami Atibuni P, David Kani Olema P, Ujeyo MSS. Demystifying
632 Menstrual Health Management to Enhance Academic Performance among Adolescent
633 Primary School Girls in Rural Uganda. East African Journal of Education Studies.
634 2022;5(2):298-309.
635 31.	Keppens G. School absenteeism and academic achievement: Does the timing of the
636 absence matter? Learning and Instruction. 2023;86:101769.

637

638

639

640

# S1 Table. Baseline menstrual-related exposures by endline school absenteeism for any reason

Figure	[Click here to access/download;Figure;Figure 1.jpg](https://www2.cloud.editorialmanager.com/pone/download.aspx?id=42269182&guid=c8d9bd13-07ba-4e95-9419-7c9293b896d0&scheme=1)[<https://www2.cloud.editorialmanager.com/pone/download.aspx?id=42269182&guid=c8d9bd13-07ba-4e95-9419-7c9293b896d0&scheme=1>](https://www2.cloud.editorialmanager.com/pone/download.aspx?id=42269182&guid=c8d9bd13-07ba-4e95-9419-7c9293b896d0&scheme=1)

Figure	[Click here to access/download;Figure;Figure 2.jpg](https://www2.cloud.editorialmanager.com/pone/download.aspx?id=42133030&guid=ff7da2cc-0990-4641-82d3-101a48ce43b5&scheme=1)[<https://www2.cloud.editorialmanager.com/pone/download.aspx?id=42133030&guid=ff7da2cc-0990-4641-82d3-101a48ce43b5&scheme=1>](https://www2.cloud.editorialmanager.com/pone/download.aspx?id=42133030&guid=ff7da2cc-0990-4641-82d3-101a48ce43b5&scheme=1)

Supporting Information

[Click here to access/download](https://www2.cloud.editorialmanager.com/pone/download.aspx?id=42269183&guid=29affd30-8682-4bc4-a78e-628b8016556c&scheme=1)

[**Supporting Information** Supplementary Table 1.docx](https://www2.cloud.editorialmanager.com/pone/download.aspx?id=42269183&guid=29affd30-8682-4bc4-a78e-628b8016556c&scheme=1)

Supporting Information

[Click here to access/download](https://www2.cloud.editorialmanager.com/pone/download.aspx?id=42269184&guid=0472ca9e-df89-4a0c-a6f2-68b79c4b3655&scheme=1)

[**Supporting Information** Supporting letter.docx](https://www2.cloud.editorialmanager.com/pone/download.aspx?id=42269184&guid=0472ca9e-df89-4a0c-a6f2-68b79c4b3655&scheme=1)

Revised Manuscript with Track Changes

1 **Title:** Associations of menstrual health with school absenteeism and ~~educational~~ examination

**Formatted:** Line spacing: Double

2 performance among Ugandan secondary school students: A prospective~~longitudinal~~ study

3

4 **Short title:** Menstrual health and educational outcomes among secondary school students in

**Formatted:** Font: Bold

5 Uganda
6 This study is conducted under a MENISCUS with **Trial Registration and Recruitment**

## 7 Details:

8 **Registry Name:** ISRCTN
9 **Registration Number:** ISRCTN45461276
10 **DOI:** <https://doi.org/10.1186/ISRCTN45461276>

**Formatted:** Font: (Default) Times New Roman, 12 pt

11 **Recruitment Start Date:** 21/03/2022
12 **Recruitment End Date:** 31/03/2023
13 The trial paper has been published here: doi: **10.1016**/**S2214**-**109X**(**25**)**00007-5** with its
14 CONSORT

15

**Formatted:** Font: Bold, Font color: Auto

**Formatted:** Space After: 0 pt, Line spacing: Double

16 **Authors:** Christopher Balekea ^*^, Levicatus Mugenyia, Kate A Nelsonb, Katherine A Thomasb,

**Formatted:** Line spacing: Double

17 Denis Ndekezia, Jonathan Reuben Enomuta, Connie Alezuyoc, John Jerrimd, Helen A Weissb

18

## 19 Affiliations:

20 ^a^ MRC/UVRI and LSHTM Uganda Research Unit, PO Box 49, Entebbe, Plot 51-59
21 Nakiwogo Road, Uganda ([Christopher.baleke@lshtm.ac.uk;](mailto:Christopher.baleke@lshtm.ac.uk)

**Formatted:** Font: (Default) Times New Roman, 12 pt

22 [Levicatus.mugenyi@mrcuganda.org,](mailto:Levicatus.mugenyi@mrcuganda.org) [denis.ndekezi@mrcuganda.org,](mailto:denis.ndekezi@mrcuganda.org) [enomutj@gmail.com](mailto:enomutj@gmail.com))

**Formatted:** Font: (Default) Times New Roman, 12 pt

**Formatted:** Font: (Default) Times New Roman, 12 pt

**Formatted:** Font: (Default) Times New Roman, 12 pt

23 ^b^ International Statistics and Epidemiology Group; Faculty of Epidemiology and Population
24 Health, London School of Hygiene & Tropical Medicine, Keppel Street, London, WC1E
25 7HT,	United	Kingdom	([kate.nelson@lshtm.ac.uk](mailto:kate.nelson@lshtm.ac.uk);	[katherine.thomas1@lshtm.ac.uk;](mailto:katherine.thomas1@lshtm.ac.uk)

**Formatted:** Font: (Default) Times New Roman, 12 pt

26 [helen.weiss@lshtm.ac.uk](mailto:helen.weiss@lshtm.ac.uk) )

**Formatted:** Font: (Default) Times New Roman, 12 pt

**Formatted:** Font: (Default) Times New Roman, 12 pt

27 ^c^ Ministry of Education and Sports, Uganda, P. O. BOX 7063 Kampala, Uganda King George
28 VI Way, Embassy House, Uganda ([calezuyo@gmail.com](mailto:calezuyo@gmail.com) )

**Formatted:** Font: (Default) Times New Roman, 12 pt

29 ^d^ UCL Institute of Education, University College London, 20 Bedford Way, London WC1H
30 0AL, United Kingdom ([j.jerrim@ucl.ac.uk](mailto:j.jerrim@ucl.ac.uk) )

**Formatted:** Font: (Default) Times New Roman, 12 pt

31

32 *Corresponding author ~~ence to be addressed to:~~
33 ~~Christopher Baleke~~
34 e~~E~~mail: [Christopher.baleke@lshtm.ac.uk](mailto:Christopher.baleke@lshtm.ac.uk)

**Formatted:** Portuguese (Portugal)

**Formatted:** Font: (Default) Times New Roman, 12 pt

**Formatted:** Portuguese (Portugal)

**Formatted:** Justified, Space After: 0 pt, Line spacing: Double

**Formatted:** Line spacing: Double

**Formatted:** Font: Bold

35

36 This study is conducted under a MENISCUS with **Trial Registration and Recruitment**

## 37 Details:

**Formatted:** Indent: Left: 0", Space After: 12 pt, Line spacing: Double

38 **Registry Name:** ISRCTN**;**
39 **Registration Number:** ISRCTN45461276**;**
40 **DOI:** <https://doi.org/10.1186/ISRCTN45461276>

**Formatted:** Font: (Default) Times New Roman, 12 pt

41 **~~Recruitment Start Date:~~** ~~21/03/2022~~

**Formatted:** Space After: 0 pt, Line spacing: Double

42 **~~Recruitment End Date:~~** ~~31/03/2023~~
43 The trial paper has been published (~~here:~~	doi: 10.1016/S2214-109X(25)00007-
44 5**~~10.1016~~**~~/~~**~~S2214~~**~~-~~**~~109X~~**~~(~~**~~25~~**~~)~~**~~00007-5~~)** with its CONSORT checklist

45

46

**Formatted:** Line spacing: Double

47 **Title:** Associations of menstrual health with school absenteeism and educational performance

**Formatted:** Left, Space After: 8 pt, Line spacing: Double

48 ~~among Ugandan secondary school students: A longitudinal study~~

## 49 ABSTRACT

**Formatted:** Line spacing: Double

50 **Background:** Relatively few studies have quantified the amount of school missed due to
51 poor menstrual health, or the impact of poor menstrual health on examination
52 performance~~ducational performance~~.

53

54 **Methods:** We conducted secondary observational analyses from data nested within ~~analysed~~
55 ~~secondary observation baseline and longitudinal endline (control arm only) data from~~
56 ~~participants in~~ a cluster-randomised trial of a menstrual health intervention in 60 Ugandan
57 secondary schools (The trial is registered as ISRCTN45461276). We used baseline data from
58 trial participants in both arms, and endline data from the control arm participants. School ~~We~~
59 ~~measured school~~ absenteeism was estimated as the self-reported number of days absent due
60 to menstruation per month and examination performance was assessed by an independently
61 set assessment by the Uganda National Examination Board ~~and assessed educational~~
62 ~~performance using examination results, at baseline and endline~~. We estimated adjusted
63 incidence rate ratios (aIRR) for associations with school absenteeism, using negative
64 binomial regression adjusted for school-level clustering. We estimated adjusted standardised
65 mean differences (aSMD) in examination scores using mixed-effects linear regression. ~~The~~
66 ~~trial is registered as ISRCTN45461276.~~
67 **Results:** Of the 3312 participants who reported menstruating in the past 6 -months at

**Formatted:** Font: (Default) Times New Roman, 12 pt, Not Highlight

**Formatted:** Font: (Default) Times New Roman, 12 pt, Not Highlight

68 baseline, 323 (9.8%) reported missing at least one day of school per month due to
69 menstruation (mean days missed=0.30 per month, (95%CI 0.27-0.34)~~At baseline, 3312~~
70 ~~participants reported menstruating in the past 6 months (mean age 15.6 years). Of these, 323~~
71 ~~(9.8%) participants reported missing at least one day of school per month due to menstruation~~
72 ~~and 1286 (38.8%) missed at least one day per month for any reason~~. Similarly, o~~O~~f the 1192
73 participants in the trial control arm ~~in the control arm~~ seen at endline, 135 (11.3%) reported
74 missing~~missed~~ at least one day due to menstruation (mean days missed=0.31~~0~~ per month
75 (95%CI 0.27-0.37~~4~~)). There was evidence that m~~As per a conceptual model described in the~~

**Formatted:** Font: (Default) Times New Roman, 12 pt

76 ~~paper, Mm~~enstrual-related absenteeism and poorer examination performance at endline were
77 both associated with ~~multiple dimensions of menstrual health~~ baseline (use of inadequate
78 menstrual materials, negative menstrual attitudes, unmet menstrual practice needs, and
79 experience of menstrual-related teasing). In addition, absenteeism (due to menstruation ~~and~~

**Formatted:** Font: (Default) Times New Roman, 12 pt

80 ~~overall)~~ was associated with menstrual pain, and poorer examination performance was
81 associated with poorer baseline menstrual knowledge.
82 **Conclusion:** Among Ugandan students, multiple dimensions of menstrual health are
83 associated with ~~educational outcomes of~~ school absenteeism and examination~~educational~~
84 performance.

85

## 86 Keywords

87 Menstrual health, school absenteeism, ~~academic performance,~~ education outcomes

88

**Formatted:** Space After: 10 pt, Line spacing: Double

89

**Formatted:** Line spacing: Double

90

91

## 92 1. Introduction

93 Menstrual health (MH) is defined as complete physical, mental, and social wellbeing in
94 relation to the menstrual cycle [1] . Poor menstrual health is prevalent in low- and middle-
95 income countries (LMICs), and ~~., and~~ may affect educational outcomes such as school
96 absenteeism and examination performance through multiple pathways, including through
97 shame, anxiety, lack of confidence, behavioural expectations, poor physical environments,
98 and inadequate menstrual products [2]. Aa review of 76 qualitative studies, including ~~with~~ 45
99 from sub-Saharan Africa and 29 from South and East Asia, ~~in LMICs~~ found that multiple
100 ~~aspects of~~dimensions of menstrual health ~~experience~~ contribute~~ing~~e to school
101 ~~absenteeism~~absenteeism and reduced engagement [2]. These dimensions include physical,
102 behavioural and social components such as ~~includeding~~inadequate menstrual materials,
103 menstrual pain, poor menstrual knowledge and confidence, ~~menstrual pain,~~ and inadequate
104 infrastructure for managing menstruation in schools [2]. ~~These factors reflect multiple~~

**Formatted:** Font: Not Italic

105 ~~dimensions of menstrual health encompassing physical, behavioural, social, and attitudinal~~

**Formatted:** Font: Not Italic

**Formatted:** Font: Not Italic

106 ~~components.~~
107 ~~A widely-cited statistic that~~
108 The statement "one in 10 school-age girls in Africa misses school or drops out for reasons
109 related to her period" is a widely-used statistic, but ~~it~~ is not supported by scientific research
110 ~~“Oone in 10 school-age girls in Africa misses school or drops out for reasons related to her~~
111 ~~period” is a widely-cited statistic that not evidence-based lacks scientific backing~~ [3, 4]. A
112 systematic review of 15 quantitative studies on school absenteeism during menstruation in
113 ~~sub-Saharan~~ Africa found that an average of 31% (95%CI 24-39%) of ~~of female~~ students
114 reported school absenteeism during menstruation either at last menstrual period (LMP) or
115 over a longer duration [5]. T ~~Additionally t~~he review found that ~~that~~ menstruation also
116 affected concentration, academic performance, and participation in sports [5]. However, the
117 number of school days missed were not quantified and there was a high degree of
118 unexplained heterogeneity between studies (I^2^=97.8%), with prevalence of absenteeism due
119 to menstrual pain ranging from 6% in Ghana to 65% in Ethiopia. The variation in prevalence
120 may be partly due to variation in the reference period for measurement of school absenteeism
121 ~~varied~~, with some using LMP and others using longer durations ~~- this may is partly be~~
122 ~~responsible to variations in prevalence~~. A recent multi-country study of data from 673,380
123 women and girls aged 15-49 in 47 countries found that the proportion reporting menstrual-
124 related school absenteeism during LMP was 15.0% (95%CI 12.7-17.3%), with the highest
125 absenteeism among 15-19 year olds (17.7%, 95%CI 15.1-20.3%) [6].
126 There is mixed evidence of an impact of menstrual health interventions on school
127 absenteeism. A recent systematic review identified nine relevant studies in LMICs, of which
128 six found evidence of a positive effect of an intervention on absenteeism ~~., but the studies~~
129 ~~deemed to be of highest quality tended to find no effect~~ [7]. However, the three~~3 of these~~
130 ~~studies which are~~ cluster- randomized control triails included~~with larger sample size hence~~
131 ~~deemed to be of highest quality~~ found no effect on absenteeism. Reasons for the lack of
132 ~~knowledge~~ evidence on the extent to which menstruation affects school attendance include a
133 lack of ~~appropriate~~ standardized and cross-validated absenteeism measures [8]., Additionally
134 these studies ~~a~~ lack ~~of~~ detail on whether absence was due to menstruation or non-menstrual
135 related factors like~~reasons~~ ~~(such as~~ general illness or ~~lack of ability to pay school~~
136 ~~fees)~~financial inadequacy to education, and the time period over which absence was assessed
137 [9].
138 A commonly-reported reason for menstrual-related absenteeism is menstrual pain, which can
139 also contribute to reduced participation, concentration, and academic performance~~Menstrual~~
140 ~~pain is a common reason for absenteeism, impaired participation and concentration and~~
141 ~~declining academic performance among university students~~ [10]. A~~A~~ systematic review of
142 38 studies (~~and meta-analysis of the association of menstrual pain (dysmenorrhea) with~~
143 ~~absenteeism among adolescents and young women identified 38 studies (~~including 23 from
144 LMICs)~~),~~ ~~and~~ finding~~ound~~ that 20.1% (95% CI 14.9–26.7) of ~~participants~~ adolescents and
145 young women reported school or university absence ~~from school or university~~ due to
146 menstrual pain and 40.9% reporting an adverse effect on classroom performance or
147 concentration ~~(dysmenorrhea) (95% CI 14.9–26.7)~~ [11]. ~~AIn another systematic review of~~

148 ~~menstrual experiences among university students, identified 74 quantitative studies globally~~
149 ~~(26 in Africa), with education performance a reported consequences of menstruation~~
150 ~~including academic performance, mainly due to menstrual pain or heavy menstrual bleeding~~
151 [10]~~.~~ In these~~is~~e reviews ~~among university students~~, few studies provided details of how the
152 educational outcomes were measured, and none used extant data (official examination results
153 and attendance records) to validate self-reported measures [10]. There is relatively little, and
154 inconclusive evidence on the impact of menstruation on broader educational issues such as
155 self-esteem, participation in school and examination~~educational academic~~ performance [10,
156 11]. ~~Here, academic performance refers specifically to measurable outcomes like~~
157 ~~examination results, while broader educational issues encompass factors influencing students’~~
158 overall engagement and success in education. In the review among university students, few
159 studies provided details of how the educational outcomes were measured, and none used
160 extant data (official examination results and attendance records) to validate self-reported
161 measures [10].
162 Formative research in Ugandan secondary school students has shown strong evidence that
163 participants missed school more frequently during menstruation than on other days, based on
164 prospective daily diaries (28.4% of schooldays were missed during menstruation compared
165 with 6.5% when not menstruating; p<0.001) [12].
166 Our recently-published MENISCUS cluster-randomised trial~~, the MENISCUS study~~
167 (“Menstrual health interventions, schooling, and mental health problems among Ugandan
168 students”)~~, evaluating the impact of a multi-component menstrual health intervention~~ showed
169 strong evidence that a multi-component menstrual health~~of an~~ intervention improved multiple
170 dimensions of menstruation, including ~~effect on multiple dimensions of menstrual health such~~
171 knowledge about puberty and menstruation, ~~more positive, greater~~ use of effective pain
172 management, menstrual self-efficacy, and ~~reduced fewer~~ unmet menstrual needs, over a one-
173 year period [13] ~~but~~. However, the trial showed little evidence of an intervention effect on
174 educational outcomes. The proportion of school days missed during menstruation ~~showed~~
175 ~~little evidence of an impact on school absence~~ (measured using prospectively -collected daily
176 diaries) was similar by arm~~during menstruation~~ (11.2% vs 13.5% ~~days missed~~in the
177 intervention vs control arms; adjusted odds ratio (aOR)=0.81, 95%CI 0.62, 1.05). Similarly, t.
178 ~~T~~here was no evidence of an impact of the intervention on examination performance
179 (adjusted mean difference (~~aMD~~aSMD)=0.06, 95%CI -0.12, 0.24) ~~or on school absence~~
180 ~~overall (10.5% days missed in both arms)~~.

181

182 The aim of this paper is to conduct a secondary ~~data~~observational analysis~~, nested within the~~
183 using data from MENISCUS trial [13, 14], to estimate associations of menstrual-related
184 experiences ~~quantify factors associated~~ with ~~the educational outcomes of~~ menstrual-related
185 school absenteeism and examination~~ducation~~ performance, among Ugandan secondary
186 school students~~[15]~~. ~~, using secondary observation data from the MENISCUS trial. While~~

**Formatted:** Font: Not Bold

187 ~~previous MENISCUS trail publication focused on evaluating intervention effects~~In this~~, the~~

**Formatted:** Font: Not Bold

**Formatted:** Font: Not Bold

**Formatted:** Font: Not Bold

**Formatted:** Font: Not Bold

**Formatted:** Font: Not Bold

**Formatted:** Font: Not Bold

**Formatted:** Font: Not Bold

**Formatted:** Font: Not Bold

**Formatted:** Font: Not Bold

**Formatted:** Font: Not Bold

188 ~~present~~ paper, we~~analysis~~ provides new insights by quantifying the associations of
189 ~~systematically identifying which specific~~ menstrual health ~~experiences~~ (including~~such as~~
190 pain management, social support, menstrual practices, and attitudes) ~~are most strongly~~
191 ~~associated~~ with number of days of school absenteeism and examination performance,
192 respectively. ~~. By using longitudinal data and employing advanced statistical approaches to~~
193 ~~disentangle these relationships and assess their independent contributions, our findings reveal~~
194 ~~critical targets for future interventions and illustrate the complex mechanisms linking~~
195 ~~menstrual health to educational outcomes in this context.~~
196 ~~These findings will add to the knowledge based on the impact of multiple dimensions of~~
197 ~~menstruation on the number of school days missed due to menstruation, and on examination~~
198 ~~performance.~~

## 199 2. Methods

200 **2.1 Study Setting**
201 The MENISCUS trial~~study~~ was conducted in Kalungu and Wakiso districts of Uganda.

**Formatted:** Font: (Default) Times New Roman, 12 pt

202 Wakiso district is the largest and largely urban district in central Uganda with an estimated
203 population of 3.4 million people [15] . In Wakiso, 17% of residents were aged 10-17 years, of
204 whom 49% were attending secondary schools [16]. Kalungu is a rural district with population
205 size 183,232 (2014 census). In Kalungu, 23% were aged 10-17 years, of whom 36% were

206

207

enrolled in secondary school [17].

## 208 2.2 Data source

209 This study is a secondary data analysis ~~study usesds~~of ~~secondary observation~~ data from the
210 MENISCUS trial, The trial ~~which~~~~aimed~~aimed to evaluate whether a multi-component
211 menstrual health intervention improves education, health and wellbeing among female
212 secondary school students in Central Uganda [13]. In this ~~paper~~study, we report findings
213 from secondary observational analyses ~~using~~of the~~both~~ baseline (both arms) and endline
214 (control arm only) ~~endline survey data from the~~trial data ~~MENISCUS trial (“Menstrual health~~
215 ~~interventions, schooling, and mental health problems among Ugandan students”) which~~
216 ~~aimed to evaluate whether a multi-component menstrual health intervention improves~~
217 ~~education, health and wellbeing among female secondary school students in Central Uganda~~.
218 Details of the trial design and results have been published previously [13, 14]. In brief, the
219 trial was a parallel-arm, cluster-randomised controlled trial in 60 schools with schools
220 randomised 1:1 to either the intervention package or the control arm.~~either immediate or~~
221 ~~delayed intervention delivery.~~ A package of intervention components included puberty
222 education, drama skits, provision of menstrual health kits, training on pain management, and
223 improvements to WASH facilities. The schools randomised to the~~in~~control arm were
224 provided~~give~~ with~~n~~ a copy of the government guidelines on menstrual hygiene management
225 and sexuality education to the headteacher, and ~~all male and female~~ Senior 2
226 ~~participants~~students were given a copy of the government menstrual management reader

227

228

229

.[14].

**Formatted:** Line spacing: Double

## 230 2.3 Sampling of schools

231 In preparation for the MENISCUS trial, a~~A~~ll government schools and a random selection of
232 private schools were contacted through phone calls and assessed for eligibility using the
233 government’s 2019 master list of e~~E~~ducation iInstitutions. Schools were eligible for the trial
234 if they were mix~~4~~ed-sex, secondary, day- or mixed day/boarding, and had at least minimum
235 water sanitation and hygiene (WASH) facilities (an improved water source and at least one
236 functional sex-specific ~~sanitation facilities~~toilet accessible to female students). ~~Also~~
237 ~~schools~~Schools also had to ~~be and were of~~have an estimated size of 50-125 or 40-125 female
238 students in Secondary 1 (first year of secondary school) in Wakiso and Kalungu district,
239 respectively.~~.~~ ~~However,~~S ~~S~~chools were not eligible ~~excluded from~~ for the triail if they were

**Formatted:** Font color: Text 1

**Formatted:** Font color: Text 1

**Formatted:** Font color: Text 1

**Formatted:** Font color: Text 1

**Formatted:** Font color: Text 1

**Formatted:** Font color: Text 1

**Formatted:** Font color: Text 1

**Formatted:** Font color: Text 1

240 participating in a menstrual health related programme, were exclusively boarding schools, or
241 were single-sex~~boys- or girls-only~~ schools. ~~School enrolment was to be and an estimated~~
242 enrolment as of January 2020 of 50-125 or 40-125 female students in Secondary 1 (first year
243 ~~of secondary school) in Wakiso and Kalungu district, respectively. We selected a~~A random
244 sample of 60 schools were selected after being confirmed as eligible and willing to
245 participate ~~an , stratifiedthen stratified by government versus private schools and district~~. We

**Formatted:** Font: Not Bold

246 over-sampled government schools as these are of interest to Ministry of Education
247 stakeholders, and to increase generalisability of findings to these schools. ~~Including both~~
248 government and private schools which differ in WASH facilities functionality [18, 19],

**Formatted:** Font color: Text 1

**Formatted:** Font color: Text 1

249 ~~education performance, and class attendance rates, ensured our sample was representative of~~

250

251

252

the full range of school types in Wakiso and Kalungu. This approach enhances the ~~generalizability of our findings across diverse school contexts.~~

## 2.4 Participant recruitment and informed consent/assent

**Commented [KN1]:** See comment in response doc - this isn't quite accurate

**Formatted:** Font: 12 pt

253 All female students enrolled in Secondary 2 (i.e. the second year of secondary school) at the
254 start of the 2022 were eligible to participate in the MENISCUS trial. We sought school-level
255 written consent from headteachers and written informed consent from parents/guardians of
256 students aged <18 years, and from students aged ≥18 years. Students aged <18 years were
257 asked to assent if we had received parental consent.

258

## 259 2.5 Data collection

260 Baseline data were collected from 21 March to 5 July 2022 and endline data were collected
261 from 5 June to 22 August 2023. The surveys included items on sociodemographic
262 characteristics, mental health and multiple dimensions of menstrual health including physical

**Formatted:** Font: Not Italic

263 symptoms (e.g., pain), menstrual practices and materials, social experiences (e.g., teasing),
264 and menstrual knowledge and attitudes ~~including~~ which were assessed alongside school
265 absenteeism due to menstruation (“menstrual-related absenteeism”) or due to other reasons.,
266 ~~and~~ All were self-completed using Open Data Kit software on tablets. The examinations were
267 independently administered and marked by the Uganda National Examination Board
268 (UNEB), with examinations administered on 16-17 March 2022 (baseline) and 24-26 July
269 2023 (endline). Baseline examinations comprised materials taught in Secondary 1 and
270 included four m~~M~~athematics and six b~~B~~iology questions, of which one question was on
271 puberty and reproduction. Endline examinations additionally included four English
272 l~~L~~anguage questions, and comprised materials taught in Secondary 1 and Secondary 2.

273

**Formatted:** Space After: 12 pt, Line spacing: Double

## 274 2.6 Study outcomes

**Formatted:** Line spacing: Double

275 Baseline menstrual-related absenteeism was assessed as self-reported number of school days
276 missed due to menstruation, excluding days where the main reason for missing school was
277 unrelated to menstruation (“Since schools reopened in January this year, how many days of
278 class did you miss during your period?” “What is the main reason for missing school during
279 your period?”). The days of school missed for any reason was obtained from the question
280 (“Since schools reopened in January this year, how many days of class did you miss for any
281 reason?”). We calculated the number of days in the denominator from the official date
282 schools re-opened post COVID-19 (10 January 2022) to the date of interview for each
283 participant, excluding school holidays. Endline menstrual-related absenteeism was assessed
284 as self-reported number of school days missed during menstruation in Terms 1 and 2 of 2023
285 until the interview date. Term 1 was from 6 February to 5 May 2023, and Term 2 started on
286 29 May 2023. To minimize recall bias, we used cognitive testing before the survey to ensure
287 clearly worded questions anchored to specific time frames, and provided reminders of school
288 terms and holidays during survey completion. During self-completion of the questionnaires,
289 research assistants were present to assist~~, and interviewers assisted~~ participants with
290 clarifications about the recall period and absence reasons. Baseline and endline examination

291

292

293

294

scores were calculated as the mean of subject-specific z-scores at each time point.

**Formatted:** English (United Kingdom)

**Formatted:** Line spacing: Double

## 295 2.7 Statistical analyses

296 Data were exported to Stata version 18 for final cleaning and analysis. We estimated the
297 mean number of days missed overall per month and the mean number of days reported to be
298 missed due to menstruation using mixed-effects negative binomial regression models to
299 estimate adjusted incidence rate ratios (aIRR) and 95% confidence intervals (CI), allowing
300 for school-level clustering and over-dispersion. Similarly, we used mixed-effects linear
301 regression models to estimate adjusted standardized mean differences (aSMD) and 95%CIs
302 for examination score.
303 We built separate models for~~To assess associations~~ ~~with~~ each group of exposure variables ,
304 ~~wWe~~ using~~ed~~ a hierarchical conceptual framework based on the integrated model of
305 menstrual experience [2] ~~(Hennegan et al 2019)~~ to ~~hypothesize relationships of baseline~~
306 ~~dimensions of menstrual health menstrual-related factors with baseline and endline school~~

**Formatted:** Font: (Default) Times New Roman, 12 pt

307 ~~absenteeism and educational performance and~~ guide adjustment ~~of each exposure variable~~
308 for potential confounders. ( Figure 1). The dimensions of menstrual health are defined in
309 Table 1.
310 ~~: A conceptual framework show relationship of multiple dimensions of menstrual health with~~
311 ~~school absenteeism and education performance).~~ In line with this~~e conceptual~~ framework, we
312 ~~defined~~ grouped variables into six hierarchical levels reflecting their conceptual proximity to
313 the outcomes. We use the term “distal” to refer to variables hypothesized to causally precede
314 and influence variables at more proximal levels: ~~as being in~~ Level 1 (most distal - socio-
315 demographic variables), Level 2 (social support during menstruation), Level 3 (menstrual
316 knowledge, attitudes and adequate menstrual product use), Level 4 (menstrual pain
317 management, menstrual practice needs, experience of menstrual-related teasing), Level 5
318 (Self-efficacy in addressing menstrual needs scale [SAMNS] [20] -menstrual care confidence

**Formatted:** Font: 12 pt

319 with menstrual preparedness and pain management sub-scales) and Level 6 (most proximal -
320 trouble concentrating in class) (Figure 1). ~~Variables were grouped into six hierarchical levels,~~

**Formatted:** Font: Not Italic

321 ~~ranging from Level 1 (most distal: socio-demographic characteristics) to Level 6 (most~~
322 ~~proximal: classroom concentration), reflecting their conceptual proximity to the outcomes,~~
323 ~~and adjustment proceeded from distal to proximal in the models (see Figure 1 for full~~
324 ~~framework).~~
325 ~~Dimensions of Mmenstrual-related factors health are defined in Table 1.~~ We fitted
326 multivariable models in which each variable was adjusted for other variables at the same
327 hierarchical level and for variables in more distal (upstream) levels, as defined in the
328 conceptual framework. An exception to this was the SAMNS sub-scales, which were not
329 adjusted for each other. We used Wald tests to assess the strength of evidence and refer to a
330 p-value<0.001 as indicating strong evidence of association [21]. We interpreted the results in
331 conjunction with effect sizes and confidence intervals to provide comprehensive assessment

332

333

of results.

334 To mitigate against the possibility of reverse causality, and to assess temporal associations of
335 exposures with the outcomes, we analysed associations of baseline exposures with endline
336 outcomes in the control arm, using analogous methods. We restricted these analyses to
337 participants in the control arm to maximise generalisability by estimating associations in the

**Commented [KN2]:** see comments in response doc

**Formatted:** Font: 12 pt

338 ~~absence of~~as the MENISCUS intervention ~~, which~~ may have modified~~erated~~ the association.

339

340

## 341 Figure 1: Conceptual ~~F~~framework for statistical analysis

**Formatted:** Left, Line spacing: Double

**Formatted:** Line spacing: Double

**Formatted:** Line spacing: Double

**Formatted:** Line spacing: Double

**Formatted:** Line spacing: Double

**Formatted:** Line spacing: Double

**Formatted:** Line spacing: Double

| **Table 1: Definitions of menstrual-related exposure factors used in the conceptual framework** | | | | |
| --- | --- | --- | --- | --- |
| **Level Survey measure** | | **Brief description** | **Calculation** | **Range & interpretation** |
| 2 | Social support for menstruation | An individual has someone who they feel okay asking for support for your period if needed (for advice, resources, emotional support) | Binary | Binary (yes/no) |
| 3 | Adequate product use | Used at least one adequate menstrual material and no inadequate materials at LMP | Binary | Binary (yes/no) |
| 3 | Knowledge of puberty & menstruation | Factual knowledge about puberty, menstruation, and the menstrual cycle | Number of 9 knowledge questions answered correctly | 0-9  (number of questions answered correctly) |
| 3 | Attitudes towards menstruation | Attitudes about what it is okay for people to do while menstruating and myths about painkillers | Number of 3 attitude questions answered positively | 0-3  (number of questions answered positively) |
| 4 | Pain at LMP | Reported having any pain at LMP. This includes | Binary | Binary (yes/no) |
| 16 | | | | |

342

343

| reporting any pain, or a symptom of headache, backache, stomach ache or cramp at LMP. | | | | |
| --- | --- | --- | --- | --- |
| 4 |  | Effective pain strategies defined as stretching, |  |  |
|  |  |  | Using at least one |  |
|  |  | painkillers, eating foods with lots of water, |  |  |
|  |  |  | effective method and no |  |
|  | Effective pain | exercising, drinking lots of clean water, holding a |  |  |
|  |  |  | ineffective methods, | Binary (yes/no) |
|  | management strategy | warm water bottle on the stomach*.* Ineffective |  |  |
|  |  |  | among girls reporting |  |
|  |  | methods defined as: doing nothing, taking antibiotics, |  |  |
|  |  |  | pain at LMP. |  |
|  |  | eating spicy foods, drinking soda |  |  |
| 4 |  | | Weighted average of i) |  |
|  |  | | core items and school- | Range 0-3. A higher score |
|  | Menstrual Practice | The extent to which individuals’ menstrual |  |  |
|  |  | | specific items (75% | indicates fewer unmet |
|  | Needs Scale (MPNS- | management practices and environments were |  |  |
|  |  | | weight) and ii) relevant | menstrual needs (i.e. better |
|  | 36)[22] | perceived to meet their needs during their LMP. |  |  |
|  |  | | material-specific items | than a lower score). |
|  |  | | (25% weight). |  |
| 4 | Pain relief | The amount of pain reduced | Binary | All/most or some/none |
| 17 | | | | |

**Formatted:** Line spacing: Double

**Formatted:** Line spacing: Double

**Formatted:** Line spacing: Double

| 4 | Experience of menstrual-related teasing | Whether boys or girls tease the participant about their period | Binary | Binary (yes/no) |
| --- | --- | --- | --- | --- |
| 5 | Self-efficacy in addressing menstrual needs scale (SAMNS- 26) [20] | Individuals’ confidence in their capabilities to address their menstrual needs. This SAMNS scale has 3 sub-scales 1) The 17-item Menstrual Hygiene Preparation and Maintenance (MHPM) (“menstrual preparedness”) sub-scale includes items regarding self-efficacy in preparing for menstruation (e.g., tracking one’s cycle and anticipating days of bleeding), accomplishing various tasks related to maintaining menstrual hygiene (e.g., obtaining, using, cleaning, and disposing, menstrual materials in a variety of contexts), and seeking assistance when needed 2) the 4-item Executing Stigmatized Tasks  (EST) sub-scale, which also included items related to | Mean score | 0-100, representing the mean percentage confidence in addressing needs. Higher % is greater confidence |
| 18 | | | | |

**Formatted:** Line spacing: Double

**Formatted:** Line spacing: Double

**Formatted:** Line spacing: Double

**Formatted:** Line spacing: Double

| obtaining and disposing menstrual materials and seeking assistance—but in situations where accomplishing the tasks involves greater risk of revealing one’s menstrual status to a male person and  3) the 5-item Menstrual Pain Management (MPM), measured confidence in mitigating menstrual pain | | | | |
| --- | --- | --- | --- | --- |
| 6 | Trouble participating in class | Whether the participant had trouble participating in class due to period, during their most recent LMP at school | Binary | Binary (yes/no) |

344

19

345 ~~For baseline analysis, we first estimated the mean number of days missed overall per month~~
346 ~~and the mean number of days reported to be missed due to menstruation. We then used~~
347 ~~negative binomial regression models to estimate adjusted incidence rate ratios (aIRR) and~~
348 ~~95% confidence intervals (CI) for the association of exposures with school absenteeism due~~
349 ~~to menstruation, allowing for over-dispersion. We accounted for school-level clustering by~~
350 ~~using random-effects models. Similarly, we used linear regression models to estimate~~
351 ~~adjusted standardized mean differences (aSMD) and 95%CIs for the association of exposures~~
352 ~~with examination score, using random-effects to account for school-level clustering. Initially,~~
353 ~~minimally-adjusted models were fitted for each outcome, adjusting for school-level~~
354 ~~clustering. We then fitted multivariable models in which each variable was adjusted for other~~

**Formatted:** Font: Not Italic

355 ~~variables at the same hierarchical level and for variables in more distal (upstream) levels, as~~
356 ~~defined in the conceptual framework (Figure 1). Here, “distal” refers to variables thought to~~
357 ~~causally precede and influence variables at more proximal levels specifically, Levels 1 to 5~~

**Formatted:** Font: Not Italic

358 ~~represent progressively less direct determinants of the outcomes, from socio-demographic~~
359 ~~characteristics (most distal) through menstrual knowledge, support, and self-efficacy, up to~~
360 ~~immediate classroom effects (most proximal). Except for the SAMNS sub-scales, each~~
361 ~~variable was adjusted according to this hierarchy.We then fitted multivariable models~~
362 ~~adjusting each variable for other variables at the same or at more distal levels (Figure 1), with~~
363 ~~the exception of the SAMNS sub-scales, which were not adjusted for each other. We used~~
364 ~~Wald tests to assess the strength of evidence and refer to a p-value<0.001 as indicating strong~~
365 ~~evidence of association~~ [22]~~. We interpreted the results in conjunction with effect sizes and~~
366 ~~confidence intervals to provide comprehensive assessment of results.~~
367 ~~To assess temporal associations of exposures with the outcomes, we also analysed~~
368 ~~associations of baseline exposures with endline outcomes, using analogous methods. We~~
369 ~~restricted these analyses to participants in the control arm to avoid possible moderating~~
370 ~~effects of the intervention.~~

## 371 Ethical approval

372 Ethical approval for MENISCUS protocol, the informed consent forms and draft CRFs were
373 obtained from the Uganda Virus Research Institute Research & Ethics Committee (UVRI-
374 REC) (reference GC/127/819) and the Uganda National Council of Science and Technology
375 (UNCST) (reference HS1525ES) and the LSHTM Research Ethics Committee (reference

376

377

22952-2).

## 378 Consent for publication

379 Participants consented to the research findings being published in international science
380 journals and electronic websites, on condition that that findings cannot be traced to

381

382

individuals.

## 383 3. RESULTS

384 Of the 4281 eligible participants, 3878 (90.5%) consented/assented and completed the
385 baseline survey (Figure 2). Of these, 3312 (79.1%) participants reported menstruating in the
386 past 6 months and were included in the baseline analyses on school absenteeism. A further
387 367 (11.1%) participants were not present for the baseline UNEB exam and were excluded
388 from baseline analyses of examination~~ducational~~ performance (Figure 2). Students who were
389 missing baseline education performance data were more likely to have greater unmet
390 menstrual needs (MPNS score, p=0.01) but there was no evidence of association with other
391 dimensions of menstrual health.

## 392 Figure 2: Study flowchart

393

**Formatted:** Line spacing: Double

## 394 3.1 Baseline characteristics of study population

395 The mean age of participants was 15.6 years (SD=0.9), with most (n=2161; 65.2%) attending
396 a privately-owned school. Around half (n=2070; 55.3%) were day students and had their
397 mother as the primary caregiver (n=1816; 54.8%). A total of 1227 (37.0%) participants lived
398 in households of ≥8 members and 571 (17.2%) reported having one or fewer meals the
399 previous day (Table 2). Overall, 2780 (83.9%) participants reported having social support
400 during their last menstrual period (LMP) and 3038 (91.7%) reported exclusive use of
401 adequate menstrual ~~materials~~ products at LMP (Table 3). A total of 1696 (51.2%) participants
402 answered at least 2 of the 3 attitude questions positively, and 538 (16.2%) answered at least 7
403 of the 9 menstrual knowledge questions correctly. Most participants (2446; 73.9%) reported
404 experiencing menstrual pain at last menstrual period, of whom 1529 (62.5%) reported using
405 an effective pain management strategy. The mean MPNS score of~~was~~ 2.11 (SD=0.52)
406 ~~indicates~~corresponds ~~tha~~ to~~suggests~~ on average response of “sometimes”~~, that the~~
407 ~~individualsparticipants reported sometimes~~having ~~experience largely~~ unmet menstrual
408 practice needs. The possible range is 0 to 3, with 3 corresponding to no unmet needs.

**Formatted:** Font: (Default) Times New Roman, 12 pt, Not Bold

**Formatted:** Line spacing: Double

409 as each item is rated from always having an unmet neednever having an unmet
410 ~~need~~Additionally, participants have a moderate level of menstrual care confidence regarding
411 ability to manage menstrual needs (mean=60.8, SD=19.0). Overall, 251 (7.6%) and 284
412 (8.6%) participants reported menstrual-related~~being~~ teasing~~ed~~ by boys and girls ~~about~~
413 ~~menstrual periods~~ respectively, and almost half of participants reported trouble participating
414 in class during periods (n=1414, 42.7%) (Table 3).

**Formatted:** Font: Font color: Text 1, English (United States)

415

416	Participants reported missing a mean of 1.25 school days per month overall (95%CI 1.17,

417	1.33), and 0.30 days due to menstruation (95%CI 0.27, 0.34). A total of 991 (30%)

418	participants reporting missing school due to menstruation since schools reopened, with 323

419	(9.8%) reporting missing at least one day per month due to menstruation.

420

421

**Formatted:** Justified, Space After: 0 pt, Line spacing: Double

**Formatted:** Line spacing: Double

## 422 3.2 Socio-demographic factors associated with menstrual-related absenteeism and

423 **examination performance at baseline**
424 Menstrual-related absenteeism and poorer examination performance were both associated
425 with being in a school with a poorer examination performance (aIRR=1.26, 95%CI 1.00,
426 1.60; aSMD=-0.44, 95%CI -0.57, -0.32), being a day student (aIRR=1.45, 95%CI 1.20, 1.77;
427 aSMD=-0.12, 95%CI -0.18, -0.05), older age (aIRR-trend=1.20, 95%CI 1.09, 1.33; aSMD-
428 trend=-0.10, 95%CI -0.13, -0.06) and having fewer meals the previous day (aIRR-trend=1.24,
429 95%CI 1.09, 1.40; aSMD-trend=-0.06, 95%CI -0.10, -0.02) (Table 2). In addition, poorer
430 examination performance was associated with older age at first menstruation (aSMD-trend=-
431 0.05, 95%CI -0.08, -0.02) and higher education level of the primary caregiver (aSMD=-0.12,

432

433

-0.19, -0.05) (Table 2).

## 434 3.3 Menstrual factors associated with school absenteeism due to menstruation and

435 **poorer examination performance at baseline**
436 Among the 991 participants reporting missing school due to menstruation, 1023 days were

**Formatted:** Justified, Space After: 0 pt, Line spacing: Double

437 missed per month (mean 1.03 per month per participant (SE=0.04) (Table 4). The most
438 commonly-reported main reason was illness (back/stomach pain or cramps: n=588; 57.2% of
439 days missed; feeling generally unwell: n=170; 17.2% of days missed). Relatively few

**Formatted:** Line spacing: Double

**Formatted:** Line spacing: Double

**Formatted:** Line spacing: Double

**Formatted:** Line spacing: Double

**Formatted:** Line spacing: Double

**Formatted:** Line spacing: Double

**Formatted:** Line spacing: Double

**Formatted:** Line spacing: Double

**Formatted:** Line spacing: Double

440 **Table 2. Socio-demographic characteristics associated with school absenteeism due to menstruation and examination performance at**
441 **baseline**

| 442 | | | | | | |
| --- | --- | --- | --- | --- | --- | --- |
| **Variable** | **Number of**  **participants** | **Days of school missed due**  **menstruation per month (n= 3312)** | | **to** | **Examination performance (n=2945)** | |
|  | **Frequency**  **(%)** | **Mean day**  **(SE)** | **aIRR**  **(95%CI)^1^** | **Standardized**  **mean exam score (SE)** | | **aSMD (95%CI)^1^** |
| **Structural-level characteristics** | | | | | | |
| **District** |  |  | P=0.38 |  | | P=0.23 |
| Wakiso | 2600 (78.5) | 0.29 (0.02) | 1 | 0.03 (0.05) | | 0 |
| Kalungu | 712 (21.5) | 0.37 (0.03) | 1.13 (0.86, 1.50) | -0.12 (0.10) | | -0.09 (-0.23, 0.05) |
| **School-level characteristics** | | | | | | |
| **School ownership** |  |  | P=0.80 |  | | P=0.99 |
| Private | 2161 (65.2) | 0.27 (0.02) | 1 | 0.07 (0.06) | | 0 |
| Government | 1151 (34.8) | 0.38 (0.03) | 0.97 (0.74, 1.27) | -0.16 (0.05) | | 0.00 (-0.14, 0.14) |

**Formatted:** Line spacing: Double

**Formatted:** Line spacing: Double

**Formatted:** Line spacing: Double

**Formatted:** Line spacing: Double

**Formatted:** Line spacing: Double

**Formatted:** Line spacing: Double

**Formatted:** Line spacing: Double

**Formatted:** Line spacing: Double

**Formatted:** Line spacing: Double

**Formatted:** Line spacing: Double

**Formatted:** Line spacing: Double

**Formatted:** Line spacing: Double

**Formatted:** Line spacing: Double

**Formatted:** Line spacing: Double

**Formatted:** Line spacing: Double

**Formatted:** Line spacing: Double

| **School-mean combined UNEB Math + Biology score** | P=0.05 |  | P<0.001 |
| --- | --- | --- | --- |
| High UNEB 1983 (53.0) 0.25 (0.02) | 1 | 0.24 (0.05) | 0 |
| Low UNEB 1758 (47.0) 0.37 (0.02) | 1.26 (1.00, 1.60) | -0.24 (0.03) | -0.44 (-0.57, -0.32) |
| **Proportion of students boarding at school** | P=0.95 |  | P=0.84 |
| < 50% 1820 (55.0) 0.35 (0.02) | 1 | -0.10 (0.06) | 0 |
| >=50% 1492 (45.0) 0.26 (0.02) | 0.99 (0.77, 1.28) | 0.12 (0.05) | 0.01 (-0.12, 0.15) |
| **School size (female baseline participants per school)** | P=0.57 |  | P=0.24 |
| Greater or equal to 59 2258 (68.2) 0.30 (0.02) | 1 | 0.03 (0.06) | -0.07 (-0.20, 0.05) |
| Fewer than 59 1054 (31.8) 0.34 (0.02) | 0.93 (0.74, 1.18) | -0.04 (0.06) | 0 |
| **Student type** | P<0.001 |  | P=0.001 |
| Boarding 1496 (45.2) 0.24 (0.02) | 1 | 0.11 (0.03) | 0 |
| Day 1816 (54.8) 0.37 (0.02) | 1.45 (1.20, 1.77) | -0.07 (0.05) | -0.12 (-0.18, -0.05) |
| **Individual-level socio-demographic & economic factors** | | | |
| **Age group** | P-trend=0.004 |  | P-trend=0.003 |
| <15 283 (8.5) 0.22 (0.03) | 1 | 0.19 (0.07) | 0.00 |
| 15 1353 (40.9) 0.25 (0.02) | 1.15 (0.83, 1.60) | 0.11 (0.05) | -0.02 (-0.13, 0.08) |

**Formatted:** Line spacing: Double

**Formatted:** Line spacing: Double

**Formatted:** Line spacing: Double

**Formatted:** Line spacing: Double

**Formatted:** Line spacing: Double

**Formatted:** Line spacing: Double

**Formatted:** Line spacing: Double

**Formatted:** Line spacing: Double

**Formatted:** Line spacing: Double

**Formatted:** Line spacing: Double

**Formatted:** Line spacing: Double

**Formatted:** Line spacing: Double

**Formatted:** Line spacing: Double

**Formatted:** Line spacing: Double

**Formatted:** Line spacing: Double

**Formatted:** Line spacing: Double

| 16 | 1234 (37.3) | 0.36 (0.02) | 1.54 (1.10, 2.15) | -0.08 (0.04) | -0.17 (-0.28, -0.06) |
| --- | --- | --- | --- | --- | --- |
| 17 | 341 (10.3) | 0.38 (0.05) | 1.66 (1.11, 2.49) | -0.14 (0.06) | -0.23 (-0.36, -0.09) |
| 18+ | 101 (3.0) | 0.48 (0.12) | 1.71 (0.97, 3.03) | -0.35 (0.10) | -0.34 (-0.53, -0.15) |
| Trend |  |  | 1.20 (1.09, 1.33) |  | -0.10 (-0.13, -0.06) |
| **Age at first menstruation** |  |  | P=0.02 |  | P-trend=0.003 |
| <13 years | 758 (22.9) | 0.34 (0.03) | 1 | 0.09 (0.06) | 0.00 |
| 13 years | 1230 (37.1) | 0.24 (0.02) | 0.74 (0.59, 0.92) | 0.05 (0.05) | -0.01 (-0.08, 0.06) |
| 14 years | 1027 (31.0) | 0.34 (0.03) | 0.92 (0.73, 1.16) | -0.07 (0.05) | -0.08 (-0.16, -0.00) |
| >14 years | 297 (9.0) | 0.40 (0.05) | 1.02 (0.73, 1.42) | -0.20 (0.06) | -0.14 (-0.25, -0.03) |
| Trend |  |  | n/a |  | -0.05 (-0.08, -0.02) |
| **Religion** |  |  | P=0.002 |  | P=0.19 |
| Catholic | 1052 (31.8) | 0.28 (0.02) | 1 | -0.03 (0.05) | 0 |
| Protestant/SDA | 1285 (38.8) | 0.29 (0.02) | 1.09 (0.88, 1.35) | 0.03 (0.05) | 0.06 (-0.01, 0.13) |
| Muslim | 959 (29.0) | 0.36 (0.03) | 1.45 (1.14, 1.84) | -0.05 (0.05) | -0.01 (-0.09, 0.06) |
| None/Other | 16 (0.5) | 0.87 (0.44) | 3.78 (1.22, 11.74) | 0.03 (0.27) | 0.07 (-0.38, 0.53) |
| **Ethnicity** |  |  | P=0.54 |  | P=0.83 |

**Formatted:** Line spacing: Double

**Formatted:** Line spacing: Double

**Formatted:** Line spacing: Double

**Formatted:** Line spacing: Double

**Formatted:** Line spacing: Double

**Formatted:** Line spacing: Double

**Formatted:** Line spacing: Double

**Formatted:** Line spacing: Double

**Formatted:** Line spacing: Double

**Formatted:** Line spacing: Double

**Formatted:** Line spacing: Double

**Formatted:** Line spacing: Double

**Formatted:** Line spacing: Double

**Formatted:** Line spacing: Double

**Formatted:** Line spacing: Double

**Formatted:** Line spacing: Double

| Muganda | 2280 (68.8) | 0.32 (0.02) | 1 | 0.00 (0.05) | 0 |
| --- | --- | --- | --- | --- | --- |
| Non Muganda | 1032 (31.2) | 0.30 (0.02) | 0.94 (0.79, 1.14) | 0.02 (0.04) | 0.01 (-0.05, 0.07) |
| **Primary caregiver** | |  | P=0.17 |  | P=0.22 |
| Mother | 1938 (58.5) | 0.33 (0.02) | 1 | 0.00 (0.05) | 0 |
| Father | 805 (24.3) | 0.27 (0.02) | 0.85 (0.69, 1.05) | 0.06 (0.05) | 0.04 (-0.02, 0.11) |
| Self and others | 569 (17.2) | 0.29 (0.03) | 0.84 (0.67, 1.06) | -0.10 (0.05) | -0.03 (-0.11, 0.05) |
| **Education of primary caregiver** | |  | P=0.15 |  | P=0.001 |
| Secondary or more | 2215 (59.2) | 0.29 (0.02) | 1 | -0.03 (0.05) | 0 |
| Primary or less | 881 (23.6) | 0.38 (0.03) | 1.09 (0.88, 1.34) | 0.04 (0.06) | 0.12 (0.05, 0.19) |
| Don't know | 645 (17.2) | 0.28 (0.03) | 0.83 (0.66, 1.05) | 0.05 (0.05) | 0.09 (0.02, 0.17) |
| **Household size** | |  | P=0.19 |  | P=0.89 |
| 0-5 | 1001 (30.2) | 0.28 (0.02) | 1 | -0.007 (0.05) | -0.01 (-0.08, 0.05) |
| 6-7 | 1084 (32.7) | 0.31 (0.03) | 1.20 (0.97, 1.49) | -0.001 (0.05) | -0.01 (-0.08, 0.05) |
| >=8 | 1227 (37.0) | 0.34 (0.03) | 1.18 (0.95, 1.45) | 0.00 (0.04) | 0 |
| **Number of meals eaten on the previous day** | |  | P-trend=0.008 |  | P-trend=0.005 |
| Three or more | 1058 (31.9) | 0.24 (0.02) | 1 | 0.05 (0.05) | 0 |

**Formatted:** Line spacing: Double

**Formatted:** Line spacing: Double

**Formatted:** Line spacing: Double

**Formatted:** Line spacing: Double

**Formatted:** Line spacing: Double

**Formatted:** Line spacing: Double

**Formatted:** Line spacing: Double

**Formatted:** Line spacing: Double

**Formatted:** Line spacing: Double

**Formatted:** Line spacing: Double

**Formatted:** Line spacing: Double

4

| Two | 1683 (50.8) | 0.31 (0.02) | 1.20 (0.99, 1.46) | 0.001 (0.05) | -0.03 (-0.09, 0.04) |
| --- | --- | --- | --- | --- | --- |
| One or fewer | 571 (17.2) | 0.41 (0.04) | 1.53 (1.19, 1.97) | -0.08 (0.05) | -0.13 (-0.21, -0.04) |
| Trend |  |  | 1.24 (1.09, 1.40) |  | -0.06 (-0.10, -0.02) |
| **Socioeconomic status quintile** |  |  | P=0.02 |  | P=0.04 |
| Highest | 656 (19.8) | 0.24 (0.03) | 1 | 0.005 (0.06) | 0 |
| Medium-high | 684 (20.7) | 0.25 (0.03) | 0.91 (0.69, 1.19) | 0.14 (0.04) | 0.12 (0.04, 0.21) |
| Medium | 655 (19.8) | 0.29 (0.03) | 0.99 (0.75, 1.30) | 0.009 (0.06) | 0.04 (-0.05, 0.13) |
| Low-medium | 676 (20.4) | 0.30 (0.03) | 0.92 (0.69, 1.22) | -0.02 (0.05) | 0.03 (-0.06, 0.13) |
| Low | 641 (19.4) | 0.47 (0.04) | 1.35 (1.01, 1.81) | -0.06 (0.05) | 0.00 (-0.09, 0.10) |
| 1 Adjusted for all socio-demographic factors and school level clustering | | | | | |
| 43  44 | | | | | |

4

445

446

**Table 3. Menstrual characteristics associated with school absenteeism due to menstruation and examination performance at baseline**

**Formatted:** Line spacing: Double

**Formatted:** Line spacing: Double

**Formatted:** Line spacing: Double

**Formatted:** Line spacing: Double

**Formatted:** Line spacing: Double

**Formatted:** Line spacing: Double

**Formatted:** Line spacing: Double

**Formatted:** Line spacing: Double

**Formatted:** Line spacing: Double

**Formatted:** Line spacing: Double

| **Variable** | **Number**  **participants** | **of** | **Days of school missed due to**  **menstruation per month (n= 3312)** | | **Examination performance (n=2945)** | |
| --- | --- | --- | --- | --- | --- | --- |
|  | **Frequency (%)** | **Mean day (SE)** | | **aIRR (95%CI)^1^** | **Standardized**  **mean exam score (SE)** | **aSMD (95%CI)^1^** |
| **Level 2 – Menstrual support** | | | | | | |
| **Social support for menstruation** |  |  | | P=0.77 |  | P=0.28 |
| Yes | 2780 (83.9) | 0.30 (0.01) | | 1 | -0.007 (0.04) | 0 |
| No | 532 (16.1) | 0.35 (0.04) | | 1.03 (0.82, 1.31) | 0.03 (0.06) | 0.04 (-0.03, 0.12) |
| **Level 3 – Menstrual knowledge, attitudes and adequate product use** | | | | | | |
| **Adequate product use at LMP** |  |  | | P<0.001 |  | P=0.008 |
| Yes | 3038 (91.7) | 0.29 (0.02) | | 1 | 0.009 (0.04) | 0 |
| No | 274 (8.3) | 0.54 (0.07) | | 1.82 (1.37, 2.42) | -0.14 (0.06) | -0.13 (-0.23, -0.03) |
| **Attitudes towards menstruation** |  |  | | P<0.001 |  | P<0.001 |

**Formatted:** Line spacing: Double

**Formatted:** Line spacing: Double

**Formatted:** Line spacing: Double

**Formatted:** Line spacing: Double

**Formatted:** Line spacing: Double

**Formatted:** Line spacing: Double

**Formatted:** Line spacing: Double

**Formatted:** Line spacing: Double

**Formatted:** Line spacing: Double

**Formatted:** Line spacing: Double

**Formatted:** Line spacing: Double

**Formatted:** Line spacing: Double

**Formatted:** Line spacing: Double

**Formatted:** Line spacing: Double

**Formatted:** Line spacing: Double

| Positive (2-3) | 1696 (51.2) | 0.25 (0.02) | 1 | 0.10 (0.05) | 0 |
| --- | --- | --- | --- | --- | --- |
| Negative (0-1) | 1616 (48.8) | 0.37 (0.02) | 1.42 (1.21, 1.68) | -0.10 (0.05) | -0.15 (-0.21, -0.10) |
| **Knowledge of puberty and menstruation** | |  | P-trend=0.58 |  | P-trend<0.001 |
| High (7-9) | 538 (16.2) | 0.27 (0.04) | 1 | 0.34 (0.07) | 0 |
| Medium (4-6) | 2378 (71.8) | 0.31 (0.02) | 1.05 (0.83, 1.33) | -0.03 (0.04) | -0.32 (-0.40, -0.25) |
| Low (0-3) | 396 (12.0) | 0.39 (0.05) | 1.09 (0.79, 1.51) | -0.30 (0.05) | -0.55 (-0.66, -0.45) |
| Trend | |  | 1.06 (0.86, 1.30) |  | -0.28 (-0.33, -0.23) |
| **Level 4 – Menstrual pain, pain management and experience of menstrual-related teasing** | | | | | |
| **Menstrual pain at LMP** | |  | p<0.001 |  | P=0.04 |
| No | 866 (26.1) | 0.15 (0.02) | 1 | 0.02 (0.05) | 0 |
| Yes | 2446 (73.9) | 0.37 (0.02) | 2.12 (1.73-2.59) | 0.001 (0.05) | 0.06 (0.00, 0.13) |
| **Effective pain management strategy at LMP** | |  | P<0.001 |  | P=0.09 |
| No pain | 866 (26.1) | 0.15 (0.02) | 1 | 0.02 (0.05) | 0 |
| Pain and used at least one effective  management strategy | 1529 (46.2) | 0.35 (0.02) | 2.13 (1.72, 2.63) | -0.02 (0.04) | 0.08 (0.01, 0.14) |
| Pain and did not use at least one effective | 917 (27.7) | 0.39 (0.03) | 2.10 (1.66, 2.66) | -0.06 (0.04) | 0.05 (-0.03, 0.12) |

**Formatted:** Line spacing: Double

**Formatted:** Line spacing: Double

**Formatted:** Line spacing: Double

**Formatted:** Line spacing: Double

**Formatted:** Line spacing: Double

**Formatted:** Line spacing: Double

**Formatted:** Line spacing: Double

**Formatted:** Line spacing: Double

**Formatted:** Line spacing: Double

**Formatted:** Line spacing: Double

**Formatted:** Line spacing: Double

**Formatted:** Line spacing: Double

**Formatted:** Line spacing: Double

**Formatted:** Line spacing: Double

**Formatted:** Line spacing: Double

| strategy | | | | |
| --- | --- | --- | --- | --- |
| **Menstrual pain relief** |  | P<0.001 |  | P=0.13 |
| No pain 866 (26.1) | 0.15 (0.02) | 1 | 0.02 (0.05) | 0 |
| All/most pain relieved 983 (29.7) | 0.34 (0.03) | 2.00 (1.59, 2.51) | 0.01 (0.05) | 0.06 (-0.01, 0.13) |
| Some/none of pain relieved 1463 (44.2) | 0.38 (0.02) | 2.20 (1.78, 2.73) | -0.005 (0.04) | 0.07 (-0.00, 0.14) |
| **Menstrual Practice Needs Score (MPNS score)** |  | P-trend<0.001 |  | P-trend<0.001 |
| High - few unmet needs (2.38-3.00] 1116 (34.0) | 0.18 (0.02) | 1 | 0.12 (0.05) | 0 |
| Medium (1.88-2.38] 1091 (33.2) | 0.27 (0.02) | 1.41 (1.15, 1.73) | -0.03 (0.04) | -0.10 (-0.17, -0.04) |
| Low - many unmet needs (0.00-1.88] 1076 (32.8) | 0.48 (0.03) | 2.02 (1.63, 2.50) | -0.09 (0.05) | -0.14 (-0.21, -0.07) |
| Trend |  | 1.44 (1.29-1.57) |  | -0.07 (-0.11, -0.03) |
| **Experience of menstrual-related teasing by boys** |  | P=0.04 |  | P=0.18 |
| No 3061 (92.4) | 0.29 (0.01) | 1 | 0.01 (0.04) | 0 |
| Yes 251 (7.6) | 0.60 (0.08) | 1.38 (1.02, 1.88) | -0.20 (0.05) | -0.07 (-0.18, 0.03) |
| **Experience of menstrual-related teasing by girls** |  | P=0.07 |  | p<0.001 |
| No 3028 (91.4) | 0.29 (0.01) | 1 | 0.02 (0.04) | 0 |
| Yes 284 (8.6) | 0.53 (0.07) | 1.31 (0.98,1.75) | -0.30 (0.06) | -0.17 (-0.27, -0.07) |

**Formatted:** Line spacing: Double

**Formatted:** Line spacing: Double

**Formatted:** Line spacing: Double

**Formatted:** Line spacing: Double

**Formatted:** Line spacing: Double

**Formatted:** Line spacing: Double

**Formatted:** Line spacing: Double

**Formatted:** Line spacing: Double

**Formatted:** Line spacing: Double

**Formatted:** Line spacing: Double

**Formatted:** Line spacing: Double

**Formatted:** Line spacing: Double

**Formatted:** Line spacing: Double

**Formatted:** Line spacing: Double

**Formatted:** Line spacing: Double

**Formatted:** Line spacing: Double

| **Level 5 – Menstrual care confidence** | | | | | |
| --- | --- | --- | --- | --- | --- |
| **Menstrual care confidence (SAMNS score)** |  |  | P=0.01 |  | P=0.73 |
| High self-efficacy (69.62-100] | 1129 (34.1) | 0.25 (0.02) | 1 | 0.05 (0.05) | 0 |
| Medium (52.31-69.62] | 1102 (33.3) | 0.25 (0.02) | 0.88 (0.72, 1.08) | -0.02 (0.05) | -0.01 (-0.07, 0.06) |
| Low self-efficacy (0-52.31] | 1081 (32.6) | 0.43 (0.03) | 1.20 (0.98, 1.49) | -0.03 (0.05) | 0.02 (-0.05, 0.09) |
| **Menstrual preparedness sub-scale** |  |  | P-trend<0.001 |  | P=0.86 |
| High (76.47-100] | 1058 (31.9) | 0.21 (0.02) | 1 | 0.07 (0.05) | 0 |
| Medium (58.25-76.47]- | 1127 (34.0) | 0.26 (0.02) | 1.15 (0.93, 1.41) | 0.002 (0.05) | 0.01(-0.05, 0.08) |
| Low (0-58.25] | 1127 (34.0) | 0.45 (0.03) | 1.54 (1.24, 1.91) | -0.06 (0.05) | 0.00 (-0.08, 0.07) |
| Trend |  |  | 1.25 (1.12, 1.39) |  | n/a |
| **Pain management sub-scale** |  |  | P=0.49 |  | P=0.71 |
| High (82-100] | 923 (27.9) | 0.28 (0.03) | 1 | -0.001 (0.05) | 0 |
| Medium (50-82] | 1241 (37.5) | 0.28 (0.02) | 0.98 (0.80, 1.21) | 0.02 (0.05) | 0.02 (-0.04, 0.09) |
| Low (0-50] | 1148 (34.7) | 0.37 (0.03) | 1.10 (0.89, 1.36) | -0.02 (0.05) | 0.03 (-0.04, 0.10) |
| **Level 6 – Class participation and attendance at LMP** | | | | | |
| **Trouble participating in class during LMP** |  |  | P<0.001 |  | P=0.91 |

**Formatted:** Line spacing: Double

**Formatted:** Line spacing: Double

**Formatted:** Line spacing: Double

**Formatted:** Line spacing: Double

**Formatted:** Line spacing: Double

**Formatted:** Line spacing: Double

**Formatted:** Line spacing: Double

| No | 1898 (57.3%) | 0.20 (0.01) | 1 | 0.007 (0.04) | 0 |
| --- | --- | --- | --- | --- | --- |
| Yes | 1414 (42.7%) | 0.46 (0.03) | 1.80 (1.51, 2.14) | -0.01 (0.05) | 0.00 (-0.06, 0.06) |
| **School absenteeism due to menstruation** |  |  |  |  | P=0.01 |
| Missed less than one day per month | n/a | n/a | n/a | 0.14 (0.04) | 0 |
| Missed at least one day per month | n/a | n/a | n/a | -0.20 (0.05) | -0.12 (-0.22, -0.03) |
| 1 Adjusted for school level clustering, level 1 (socio-demographic) factors and variables at the same or more distal levels. SAMNS scores and  sub-scales are not adjusted for each other. Trouble participating, and school absenteeism are not adjusted for each other. | | | | | |

447

448

449

## 450 ~~3.2 Socio-demographic factors associated with menstrual-related absenteeism and~~

451 **~~examination performance at baseline~~**
452 ~~Menstrual-related absenteeism and poorer examination performance were both associated~~
453 ~~with being in a school with a poorer examination performance (aIRR=1.26, 95%CI 1.00,~~
454 ~~1.60; aMD=-0.44, 95%CI -0.57, -0.32), being a day student (aIRR=1.45, 95%CI 1.20, 1.77;~~
455 ~~aMD=-0.12, 95%CI -0.18, -0.05), older age (aIRR-trend=1.20, 95%CI 1.09, 1.33; aMD-~~
456 ~~trend=-0.10, 95%CI -0.13, -0.06) and having fewer meals the previous day (aIRR-trend=1.24,~~
457 ~~95%CI 1.09, 1.40; aMD-trend=-0.06, 95%CI -0.10, -0.02) (Table 2). In addition, poorer~~
458 ~~examination performance was associated with older age at first menses (aMD-trend=-0.05,~~
459 ~~95%CI -0.08, -0.02) and higher education of the primary caregiver (aMD=-0.12, -0.19, -0.05)~~

460

461

~~(Table 2).~~

## 462 ~~3.3 Menstrual factors associated with school absenteeism due to menstruation and~~

463 **~~poorer examination performance at baseline~~**
464 ~~Among the 991 participants reporting missing school due to menstruation, 1023 days were~~
465 ~~missed per month (mean per participant =1.03 per month (SE=0.04) (Table 4). The most~~
466 ~~commonly reported main reason was illness (back/stomach pain or cramps: n=588; 57.2% of~~
467 ~~days missed; feeling generally unwell: n=170; 17.2% of days missed). Relatively few~~
468 ~~participants reported other reasons (stigma-related, the physical environment or socio-~~
469 ~~economic) as the main reason for missing school during menstruation (Table 4).~~

34

470

471

472

**Table 4: Main reason for missing school due to menstruation, reported at baseline**

**Formatted:** Line spacing: Double

**Formatted:** Line spacing: Double

**Formatted:** Line spacing: Double

**Formatted:** Line spacing: Double

**Formatted:** Line spacing: Double

**Formatted:** Line spacing: Double

**Formatted:** Line spacing: Double

| **Main reason for missing school due to menstruation** | **Number of participants n (%)** | **Mean (SD) days per participant missed due to menstruation per month among those reporting**  **this reason** | **Total days missed due to menstruation per month with this as the main reason** | **Proportion of days missed due to menstruation per month with this as**  **the mains reason** |
| --- | --- | --- | --- | --- |
| **Total** | **991** | **1.03 (1.16)** | **1023** | **100%** |
| **Illness** | | | | |
| Back/stomach pain or cramps | 588 (59.3) | 1.00 (1.06) | 585 | 57.2% |
| Feeling generally unwell | 170 (17.2) | 0.98 (1.20) | 165 | 16.2% |
| **Stigma** | | | | |
| Scared of leaking blood on clothes | 59 (6.0) | 0.92 (0.95) | 54 | 5.3% |
| Not wanting to change pad or absorbents at school | 2 (0.2) | 2.49 (3.01) | 5 | 0.5% |

**Formatted:** Line spacing: Double

**Formatted:** Line spacing: Double

**Formatted:** Line spacing: Double

**Formatted:** Line spacing: Double

**Formatted:** Line spacing: Double

**Formatted:** Line spacing: Double

**Formatted:** Line spacing: Double

**Formatted:** Line spacing: Double

**Formatted:** Line spacing: Double

**Formatted:** Line spacing: Double

**Formatted:** Line spacing: Double

**Formatted:** Line spacing: Double

| Household members telling me not to go to school  during period | 12 (1.2) | 1.86 (2.38) | 22 | 2.2% |
| --- | --- | --- | --- | --- |
| Worried that others may know I have my periods | 18 (1.8) | 0.79 (0.53) | 14 | 1.4% |
| Scared of having to ask for help because of  periods | 6 (0.6) | 0.94 (0.72) | 6 | 0.5% |
| **Physical environment** | | | | |
| School toilets or latrines not clean | 34 (3.4) | 1.20 (1.28) | 41 | 4.0% |
| Lack of access to toilet paper at school | 4 (0.4) | 0.56 (0.24) | 2 | 0.2% |
| Lack of access to water and/or soap at school | 17 (1.7) | 1.34 (2.27) | 23 | 2.2% |
| Not enough privacy in school toilet/latrine | 34 (1.4) | 1.20 (1.28) | 41 | 4.0% |
| **Socio-economic** | | | | |
| Unable to afford pads or other absorbents | 35 | 1.50 (1.45) | 52 | 5.1% |
| Lack of knickers | 31 | 1.26 (1.35) | 39 | 4.8% |

473

474

475	participants reported other reasons (stigma-related, the physical environment or socio-

476

477

478

economic) as the main reason for missing school during menstruation (Table 4).

479 After adjusting for variables at the same hierarchical ~~or more distal~~ levels of the hierarchical
480 framework, ~~and for variables considered causally antecedent (i.e., more distal),factors at the~~
481 ~~same or more distal levels,~~ there was strong evidence that menstrual-related absenteeism and
482 poorer examination performance were both associated with not exclusively using adequate
483 menstrual ~~materials~~ products (aIRR=1.82, 95%CI 1.37, 2.42; ~~aMD~~aSMD=-0.13, 95%CI -
484 0.23, -0.03), negative menstrual attitudes (aIRR=1.42, 95%CI 1.21, 1.68, ~~aMD~~aSMD=-0.15,
485 95%CI -0.21, -0.10), menstrual pain at LMP (aIRR=2.12, 95%CI 1.73, 2.59;
486 ~~aMD~~aSMD=0.06, 95%CI 0.00, 0.13), more unmet menstrual practice needs (p-trend<0.001)
487 and reported experience of menstrual-related teasing at LMP by girls (aIRR=1.31, 95%CI
488 0.98, 1.75; ~~aMD~~aSMD=-0.17, 95%CI -0.27, -0.07) (Table 3).	In absolute terms, the
489 difference was relatively small, for example, the mean days missed per month among
490 participants with no menstrual pain at LMP was 0.15 (SE 0.02), compared with 0.37 (SE

491

492

0.02) among participants with pain at LMP.

493 In addition, m~~After adjustment, m~~enstrual-related absenteeism was associated with lack of
494 perceived pain relief at LMP (aIRR=2.20, 95%CI 1.78, 2.73 for minimal/no pain relieved vs
495 no pain), menstrual-related teasing by boys (aIRR=1.38, 95%CI 1.02, 1.88), poor menstrual

**Formatted:** Font: (Default) Times New Roman, 12 pt

496 care confidence (aIRR=1.20, 95%CI 0.98, 1.49 for lowest vs highest quartile, p-trend=0.01),
497 especially due to lack of menstrual preparedness (aIRR=1.54, 95%CI 1.24, 1.91, p-
498 trend<0.001) and with trouble participating in class during LMP (aIRR=1.80, 95%CI 1.51,
499 2.14). In absolute terms, the differences were~~as~~ relatively small, for example, the mean days
500 missed per month among participants with no menstrual pain at LMP was 0.15 (SE 0.02),
501 compared with 0.37 (SE 0.02) among participants with pain at LMP. Similar factors were
502 associated with missing school overall (i.e. for any reason) (Table ~~in Supplementary~~ S1 ~~Table~~

503

504

~~1~~).

505	Poorer examination performance was also associated with school absenteeism (aSMD=-0.12,

506	95%CI -0.22, -0.03 for >1 vs <1 day missed per month due to menstruation). ~~In absolute~~

**Formatted:** Underline

507	~~terms, the difference was relatively small, for example, the mean days missed per month~~

508	~~among participants with no menstrual pain at LMP was 0.15 (SE 0.02), compared with 0.37~~

509

510

~~(SE 0.02) among participants with pain at LMP.~~

## 511 3.4 Menstrual factors at baseline associated with menstrual-related absenteeism and

512 **poorer examination performance at endline.**
513 Among the 1652 participants in control arm schools at baseline, 1253 (75.8%) were in control
514 schools at endline and completed the endline survey (Figure 2). Of these, 1192 (95.1%)
515 reported having menstruated in the past 6 months and were included in endline analysis, and

516

517

1099 (92.2%) had examination data performance at endline (Figure 2).

518 Among these participants, 135 (11.3%) reported missing at least one day of school due to
519 menstruation, and the mean days missed was 0.31 per month (95%CI 0.27-0.37).
520 M~~menstrual~~enstrual-related absenteeism and poorer examination performance at endline were
521 both associated with using inadequate menstrual products at baseline (aIRR=1.76, 95%CI
522 0.95, 3.24; ~~aMD~~aSMD=-0.19, 95%CI -0.34, -0.03), negative menstrual attitudes (aIRR=1.76,
523 95%CI 1.26, 2.46; ~~aMD~~aSMD=-0.16, 95%CI -0.24, -0.08), more unmet menstrual practice
524 needs (aIRR=1.75, 95%CI 1.12-2.71; aSMD= -0.10, 95%CI -0.21 to 0.00) and experience of
525 menstrual-related teasing ~~about menstruation~~ by girls (aIRR=1.89, 95%CI 1.06, 2.73;

526

527

~~aMD~~aSMD=-0.33, 95%CI -0.48, -0.18) (Table 5).

528 In addition, menstrual-related absenteeism was associated with baseline menstrual pain
529 (aIRR=3.08, 95%CI 2.04, 4.65), including if there was pain management but no pain relief
530 (aIRR=3.42, 95%CI 2.18, 5.36 vs no pain), and trouble participating in class during LMP
531 (aIRR=1.69, 95%CI 1.20, 2.38). Endline examination performance was additionally
532 associated with poorer baseline menstrual knowledge (~~aMD~~aSMD=-0.44, 95%CI -0.60, -

533

534

0.28) (Table 5).

535	Similar factors were associated with school absenteeism for any reason (including

536	menstruation) (Table S1). For example, the mean number of days missed due to menstrual

537	pain was 1.36 days per month, vs 0.88 among those without pain at LMP (aIRR=1.41, 95%CI

538	1.26-1.57).

539

540	**Table 5. Baseline menstrual characteristics associated with endline school absenteeism due to menstruation and examination**

541	**performance ~~at baseline~~**

542

**Variable	Number	of**

**participants**

**Formatted:** Line spacing: Double

**Days of school missed due to menstruation at endline (n= 1192)**

**Examination performance at endline (n=1099)**

**Frequency (%)	Mean day (SE)	aIRR (95%CI)^1^	Standardized**

**mean exam score (SE)**

**Formatted:** Line spacing: Double

**Formatted:** Line spacing: Double

**Formatted:** Line spacing: Double

**aSMD (95%CI)^1^**

| **Level 2 – Menstrual support** | | | |
| --- | --- | --- | --- |
| **Social support for menstruation** | P=0.18 |  | P=0.15 |
| Yes 1010 (84.7) 0.32 (0.03) | 1 | -0.05 (0.08) | 0 |
| No 182 (15.3) 0.25 (0.04) | 0.73 (0.45, 1.16) | -0.06 (0.09) | -0.09 (-0.03, 0.20) |
| **Level 3 – Menstrual knowledge, attitudes and adequate product use** |  |  |  |

**Adequate product use**	P=0.07	P=0.01

**Formatted:** Line spacing: Double

**Formatted:** Line spacing: Double

**Formatted:** Line spacing: Double

Yes	1106 (92.8)	0.30 (0.02)	1	-0.08 (0.08)	0

**Formatted:** Line spacing: Double

No	86 (7.2)	0.48 (0.12)	1.76 (0.95, 3.24)	-0.23 (0.11)	-0.19 (-0.34, -0.03)

**Formatted:** Line spacing: Double

**Attitudes towards menstruation**	P<0.001	P<0.001

**Formatted:** Line spacing: Double

Positive (2-3)	614 (51.5)	0.23 (0.03)	1	0.01 (0.08)	0

**Formatted:** Line spacing: Double

Negative (0-1)	578 (48.5)	0.40 (0.04)	1.76 (1.26, 2.46)	-0.18 (0.08)	-0.16 (-0.24, -0.08)

**Formatted:** Line spacing: Double

**Knowledge of puberty and menstruation**	P=0.72	P-trend<0.001

**Formatted:** Line spacing: Double

High (7-9)	198 (16.6)	0.26 (0.05)	1	0.26 (0.09)	0

**Formatted:** Line spacing: Double

Medium (4-6)	839 (70.4)	0.32 (0.03)	1.13 (0.72, 1.79)	-0.06 (0.07)	-0.22 (-0.33, -0.10)

**Formatted:** Line spacing: Double

Low (0-3)	155 (13.0)	0.36 (0.08)	0.96 (0.50, 1.83)	-0.29 (0.09)	-0.44 (-0.60, -0.28)

**Formatted:** Line spacing: Double

Trend	-0.22 (-0.30, -0.14)

**Formatted:** Line spacing: Double

**Level 4 – M~~m~~enstrual pain, pain management and experience of teasing**

**Formatted:** Line spacing: Double

**Menstrual pain at LMP**	P<0.001	P=0.68

No pain	305 (25.6)	0.13 (0.04)	1	0.03 (0.09)	0

**Formatted:** Line spacing: Double

**Formatted:** Line spacing: Double

Any pain	887 (74.4)	0.37 (0.03)	3.08 (2.04, 4.65)	-0.07 (00.22, 0.09)	-0.01 (-0.11, 0.08)

**Formatted:** Line spacing: Double

**Effective pain management strategy**	P<0.001	P=0.93 No pain	305 (25.6)	0.13 (0.04)	1	0.03 (0.09)	0

**Formatted:** Line spacing: Double

**Formatted:** Line spacing: Double

Pain and used at least one effective

**Formatted:** Line spacing: Double

management strategy	538 (45.1)	0.39 (0.04)	3.07 (1.98, 4.76)	-0.06 (0.09)	-0.01 (-0.11, 0.10)

**Formatted:** Line spacing: Double

Pain and did not use at least one

effective strategy	349 (29.3)

0.36 (0.05)	3.09 (0.95, 2.15)	-0.07 (0.08)	-0.02 (-0.13, 0.09)

**Menstrual pain relief**	P<0.001	P=0.64

**Formatted:** Line spacing: Double

No pain	305 (25.6)	0.13 (0.04)	1	0.03 (0.09)	0

**Formatted:** Line spacing: Double

All/most pain relieved	371 (31.1)	0.35 (0.05)	2.72 (1.72, 4.29)	-0.11 (0.10)	-0.04 (-0.15, 0.07)

**Formatted:** Line spacing: Double

Some/none of pain relieved	516 (43.3)	0.39 (0.03)	3.42 (2.18, 5.36)	-0.04 (0.08)	0.01 (-0.10, 0.11)

**Formatted:** Line spacing: Double

**Menstrual Practice Needs Score (MPNS score)**	P=0.005	P=0.06

**Formatted:** Line spacing: Double

High - few unmet needs (2.38-3.00]	445 (37.6)	0.20 (0.03)	1	0.05 (0.08)	0

**Formatted:** Line spacing: Double

Medium (1.88-2.38]	372 (31.4)	0.35 (0.05)	1.43 (0.95, 2.15)	-0.07 (0.09)	-0.07 (-0.17, 0.04)

**Formatted:** Line spacing: Double

Low - many unmet needs (0.00-1.88]	366 (30.9)	0.42 (0.04)	1.75 (1.12, 2.71)	-0.13 (0.08)	-0.10 (-0.21, 0.00)

**Formatted:** Line spacing: Double

**Experience of menstrual-related teasing by boys**	P=0.18	P=0.25

**Formatted:** Line spacing: Double

No	1098 (92.1)	0.29 (0.02)	1	-0.04 (0.08)	0

**Formatted:** Line spacing: Double

Yes	94 (7.9)	0.59 (0.12)	1.50 (0.83, 2.73)	-0.16 (0.13)	0.07 (-0.09, 0.22)

**Formatted:** Line spacing: Double

**Experience of menstrual-related teasing by girls**	P=0.03	P<0.001

**Formatted:** Line spacing: Double

No	1087 (91.2)	0.29 (0.02)	1	-0.02 (0.08)	0

**Formatted:** Line spacing: Double

Yes	105 (8.8)	0.56 (0.09)	1.89 (1.06, 3.37)	-0.35 (0.12)	-0.33 (-0.48, -0.18)

**Formatted:** Line spacing: Double

**Formatted:** Line spacing: Double

**Formatted:** Line spacing: Double

**Formatted:** Line spacing: Double

**Formatted:** Line spacing: Double

| **Level 5 – Menstrual care confidence** | | | | |
| --- | --- | --- | --- | --- |
| **Menstrual care confidence (SAMNS score)** |  | p-trend=0.40 |  | P=0.55 |
| High self-efficacy (69.62-100) 420 (35.2) | 0.28 (0.04) | 1 | 0.02 (0.09) | 0 |
| Medium (52.31-69.62) 397 (33.3) | 0.30 (0.04) | 1.08 (0.71, 1.62) | -0.02 (0.09) | 0.02 (-0.08, 0.12) |
| Low (0-52.31) 375 (31.5) | 0.36 (0.04) | 1.20 (0.78, 1.83) | -0.13 (0.08) | -0.04 (-0.15, 0.07) |
| Trend |  | 0.91 (0.74, 1.13) |  |  |
| **Level 6 – Class participation and attendance at LMP** |  |  |  |  |

**Trouble participating in class during LMP**	P<0.001	P=0.52

**Formatted:** Line spacing: Double

**Formatted:** Line spacing: Double

**Formatted:** Line spacing: Double

**Formatted:** Line spacing: Double

No	678 (56.9)	0.20 (0.02)	1	-0.05 (0.08)	0

**Formatted:** Line spacing: Double

Yes	514 (43.1)	0.47 (0.04)	1.69 (1.20-2.38)	-0.06 (0.08)	0.03 (-0.06, 0.12)

**Formatted:** Line spacing: Double

**School	absenteeism	due	to**

**Formatted:** Line spacing: Double

**menstruation**	n/a	n/a	n/a	P=0.43

Missed less than one day per month	n/a	n/a	n/a	-0.04 (0.08)	0

Missed at least one day per month	n/a	n/a	n/a	-0.20 (0.10)	-0.06 (-0.22, 0.09)

**Formatted:** Line spacing: Double

**Formatted:** Line spacing: Double

1 Adjusted for school level clustering and baseline variables at Level 1 (socio-demographic) and at the same or more distal levels.

**Formatted:** Line spacing: Double

543

**Formatted:** Line spacing: Double

544

545

546

547

548

549	~~Similar factors were associated with school absenteeism for any reason (including~~

550	~~menstruation) (Supplementary Table in S1 Table 1). For example, the mean number of days~~

551	~~missed due to menstrual pain was 1.36 days per month, vs 0.88 among those without pain at~~

552

553

~~LMP (aIRR=1.41, 95%CI 1.26-1.57).~~

554 **4. DISCUSSION**
555 **4.1 Multiple dimensions of menstrual health are associated with school absenteeism and**
556 **poor examination performance**
557 In this setting, there is strong evidence that use of inadequate menstrual products, negative
558 attitudes towards menstruation, having many unmet menstrual needs, menstrual pain and
559 experience of menstrual-related teasing ~~multiple dimensions of menstrual health~~ were
560 associated with both increased menstrual-related absenteeism and poorer examination
561 performance. ~~These included use of inadequate products, negative attitudes towards~~
562 ~~menstruation, having many unmet menstrual needs, menstrual pain and experience of teasing.~~
563 These results add to the limited evidence quantifying both the amount of menstrual-related
564 absenteeism, and the specific dimensions of menstrual health ~~menstrual-related factors~~

565

566

associated with absenteeism and examination performance.

567 ~~Although our~~We have previously shown that a multi-component menstrual health
568 intervention ~~(MENISCUS) significantly~~ improved dimensions of ~~various~~ menstrual health
569 including~~aspects such as~~ knowledge, attitudes, self-efficacy and pain management, ~~and~~
570 ~~attitudes,~~ there was no evidence of an intervention effect on educational outcomes (~~it did not~~
571 ~~reduce~~ absenteeism and~~or improve~~ examination performance) [13] . Through this~~In contrast,~~
572 ~~our~~ observational analysis, we have shown that multiple dimensions of ~~identified specific~~
573 menstrual health are ~~factors independently and strongly~~ associated with educational
574 outcomes, but the absolute effect was small, which may help explain why the intervention
575 was not sufficient to improve these educational outcomes. ~~absenteeism and education~~

576

577

~~outcomes, highlighting critical targets for future interventions.~~

## 578 4.2 The impact of menstrual health on number of days of school missed due to

579 **menstruation**
580 Our findings that 10% of participants reported missing at least one school day per month due
581 to menstruation supports the widespread claim that one in 10 girls miss school due to
582 menstruation [3]. However, on average, participants reported missing less than half a day of
583 school per month due to menstruation (0.30 days). The prevalence of menstrual-related
584 absenteeism is at the lower end of the range seen in the systematic review of studies in Sub-
585 Saharan Africa (6-65% missing school during menstruation) [5], and slightly lower than the
586 recent multi-country study (17.7%, 95%CI 15.1-20.3%) among 15-19 year olds [6]. Reported
587 school absence may be lower in our study because i) students had missed almost two years of
588 school due to COVID-19 closures and may have felt pressure from teachers and parents to
589 make up the time; ii) reported days of school missed due to menstruation may be under-
590 reported by boarding students who interpreted being on school premises as attending school,
591 and iii) the relatively long period of reporting in our study may have led to underestimation
592 due to recall bias.
593 The variation in estimates between studies is also likely due to challenges measuring school
594 attendance accurately [8]. Measuring school attendance using self-report often underestimates
595 absenteeism compared to using school registers [23], which may be due to social desirability
596 bias [8, 24], stigma and taboos around menstruation, and recall bias. This is supported by
597 qualitative findings from a Kenyan study, in which girls rarely reported school absenteeism
598 themselves, but reported that ‘other’ girls were absent from school due to menstruation~~did~~

**Formatted:** Font: (Default) Times New Roman, 12 pt

599 [25]. However in some settings, including Uganda, school registers are incomplete and/or
600 inaccurate, due to teachers not completing the registers, and social desirability bias if school
601 funding is linked with student numbers [23]. Future studies should consider a combination of

602

603

these methods, daily diaries and spot checks to triangulate data on school attendance [23].

604 To our knowledge, this is the first study in sub-Saharan Africa to quantify days of school
605 absenteeism attributable to specific dimensions of menstrual health. ~~In our study~~We found
606 that, students reporting menstrual pain had more than twice~~double~~ the rate of menstrual-
607 related absenteeism than those with no pain, and school absenteeism was highest among
608 those without ~~in~~adequate pain relief. Further, we found that menstrual pain or feeling
609 generally unwell were responsible for about three-quarters of days of school absenteeism due
610 to menstruation. ~~To our knowledge, this is the first study to quantify days of school~~
611 ~~absenteeism attributable to specific menstrual-related factors.~~
612 At baseline, there was evidence that participants with low menstrual self-efficacy were more
613 likely to miss school due menstruation compared to those with high menstrual self-efficacy.
614 The association was strongest with the~~the~~ ~~sub-scale of lack of~~ menstrual preparedness sub-
615 scale on the menstrual self-efficacy scale (Table 1~~e.g., tracking one’s cycle, obtaining, using,~~
616 ~~cleaning, and disposing of menstrual products, and seeking assistance when needed~~). This fits
617 with findings from qualitative studies that students with low menstrual confidence might feel
618 uncomfortable to be around students or teachers during menstruation and hence choose to
619 miss school during menstruation [9, 26]. This association may also be due to girls fearing
620 leakage of blood whilst in class and hence missing school [2].
621 ~~This contrast between the MENISCUS intervention’s limited impact on absenteeism~~[14] ~~and~~
622 ~~the strong associations found in our observational analysis underscores the complexity of~~
623 ~~translating menstrual health improvements into school attendance gains. It suggests that~~
624 ~~structural, cultural, and economic barriers outside the scope of the intervention may play a~~
625 ~~role in limiting educational impacts.~~

## 626 4.3 The impact of menstrual health on examination~~ducational~~ performance

627 Qualitative studies have described how participants perceive that menstruation affects their
628 ~~academic~~ educational ~~performance~~ outcomes [2] but there are few rigorous quantitative
629 studies on this. Our findings broadly support the hypothesis that menstrual
630 health~~menstruation~~ affects ~~affects academic~~ educational ~~performance~~ outcomes through
631 multiple pathways, through menstrual pain or use of inadequate materials, but also due to
632 social stigma and lack of menstrual knowledge, and through school ~~absteneeism~~absenteeism.
633 Using a standardised assessment set by the UNEB, we found that poorer examination
634 performance was associated with use of inadequate products, negative attitudes towards
635 menstruation, having many unmet menstrual needs, menstrual pain and experience of
636 menstrual-related teasing ~~multiple dimensions of menstrual health~~. There was strong
637 evidence of associations between poorer examination scores and use of inadequate materials,
638 poorer menstrual attitudes, poorer menstrual knowledge, having more unmet menstrual
639 practice needs and experience of being teased in relation to periods. However, overall,
640 associations of dimensions of menstrual health with examination scores were weaker than
641 with school absenteeism. This is as expected given that absenteeism is more proximal to poor

642

643

menstrual health than educational performance.

644 Our results support qualitative findings from the systematic review [2] and a qualitative study
645 of 120 girls aged 14-16 years in in Kenya where girls reported that their attention in class was
646 disturbed by worries of leakage of menstrual blood and staining of clothing [27]. A further
647 study found that students reported failing to stand up and answer questions, write on the
648 board and miss exams during menstruation [28]. Missing at least one day of school due to
649 menstruation at LMP at baseline was associated with poorer baseline examination scores, but
650 not with endline examination scores. This difference may be due to chance or due to the
651 longer temporal gap between missing school at baseline and examination performance at
652 endline. Persistent school absenteeism increases the risk of school dropout, lowers girls' self-
653 esteem, and compromises future productivity [29]. A strength of our study was the use of an
654 independent examination set by UNEB. We identified one previous study in Uganda which
655 evaluated academic performance similarly, using assessments in English language,
656 Mathematics, Integrated Science, and Social Studies set by UNEB [30] . In this small
657 randomised controlled trial of 60 participants in two primary schools in Uganda, a menstrual
658 health intervention (which included telling menstrual stories and playing games related to
659 menstrual management) was associated with improved academic scores at 6 weeks after the

660

661

662

intervention [30].

663 Overall, research on the effects of menstrual health on e~~ducational~~ xamination
664 performance~~performance~~ remains limited hence scarce causal evidence [5].. But several other
665 different forms of school absenteeism due sickness, domestic violence have been are
666 associated with academic performance [31] but these maybe hard from separate from
667 menstrual related absenteeism. In our study, although we observed associations between
668 menstrual health factors and examination performance, these findings are modest and should
669 be interpreted cautiously due to potential limitations such as residual confounding, limited
670 sensitivity of educational measures.

671

672 ~~It found that absences for truancy, sickness, exceptional domestic circumstances, and family~~

**Formatted:** Font color: Red

**Formatted:** Font color: Red

673 ~~holidays are all negatively associated with academic achievement. The study suggests that the~~
674 ~~effects of absenteeism are complex, potentially involving factors beyond just lost instruction,~~
675 ~~such as behavioral, health-related, and psychosocial pathways, indicating the need for tailored~~
676 ~~interventions to improve student attendance and achievement~~

677

678 ~~Generally, despite the lack of intervention effect of exam performance~~ [14], our findings

**Formatted:** Space After: 12 pt, Line spacing: Double

679 ~~showing strong associations between menstrual health dimensions and poorer examination~~
680 ~~performance, and this emphasize the need for more tailored, multifaceted approaches that~~
681 ~~address not only menstrual health but also broader contextual barriers to enhance educational~~
682 ~~outcomes.~~

683

**Formatted:** Line spacing: Double

684

## 685 4.4 Strengths and limitations of the study

686 Strengths of our study include the measurement of multiple dimensions of menstrual health;
687 use of both cross-sectional and temporal~~longitudinal~~ analyses (to minimise reverse causality);
688 measurement of reported number of schooldays missed due to menstruation over a defined
689 period; use of a self-completed questionnaire (to minimise social desirability bias); use of
690 validated tools for menstrual care confidence and unmet practice needs; and assessment of
691 ~~educational~~ examination performance using an examination set independently by UNEB,

692

693

tailored to material that had been taught across all 60 schools.

694 Limitations include possible recall bias from retrospective, self-reported outcome of school
695 attendance, residual confounding, and challenges related to the long duration (one year)
696 between baseline menstrual experiences and endline outcomes ~~for the longitudinal analyses~~.
697 ~~Therefore~~ T~~The~~ he examination~~ducational~~ performance outcome is objective but limited,
698 especially at baseline, by the relatively sparse amount of material that had been taught
699 between schools re-opening after COVID-9 in January 2022, and the assessment in March
700 2022. This resulted in a lack of discrimination between potentially low- and high-achieving
701 students. Future research with longer follow-up, larger sample sizes, more sensitive and
702 diverse educational outcome measures, and designs that better isolate menstrual health effects
703 from other educational influences is needed to fully understand the impact of menstrual

704

705

health on ~~academic achievement~~examination performance.

## 706 5.5 Conclusion and Recommendation

707 Multiple dimensions of menstrual health such as use of inadequate products, negative

**Formatted:** Space After: 12 pt, Line spacing: Double

708 attitudes towards menstruation, having many unmet menstrual needs, menstrual pain and
709 experience of menstrual-related teasing are strongly associated with school absenteeism and
710 subsequent poor education performance among female students in Ugandan secondary
711 schools, but the absolute effect size is small as menstrual-related absenteeism is infrequent.
712 This small effect size is a plausible explanation for why ~~While~~ the MENISCUS intervention
713 improved menstrual health indicators [14], but had no impact on educational outcomes~~these~~

**Field Code Changed**

714 ~~positive changes did not translate into measurable educational benefits,~~ [13]. The~~indicating~~
715 ~~that future efforts must also address broader structural and contextual barriers in addition to~~
716 ~~menstrual health itself. Our results highlight a critical~~ gap between improving menstrual
717 health versus ~~and~~ achieving tangible gains in educational outcomes ~~absenteeism and~~
718 ~~academic performance.~~ emphasizes the need for more tailored, multifaceted approaches that
719 address not only menstrual health but also broader contextual barriers to enhance educational
720 outcomes. While the MENISCUS intervention improved menstrual health indicators [14],

**Field Code Changed**

721 these positive changes did not translate into measurable educational benefits, indicating that
722 future efforts must also address broader structural and contextual barriers in addition to
723 menstrual health itself. Nevertheless, improving menstrual health remains a critical priority
724 globally. Addressing this issue is essential not only for safeguarding physical and

725

726

727

psychological well-being but also advancing gender equality and human rights issues.

**Formatted:** Line spacing: Double

## 728 Acknowledgements

729	We would like to thank the staff, students, and communities of participating schools for their

730	engagement with the study; the stakeholders from the Uganda Ministry of Education and

731

732

733

Sports; and District Education Officers in Wakiso and Kalungu districts.

734	**Abbreviations**

735

736 MH: Menstrual health; LMP: Last menstrual period; CI: Confidence interval; LRT:

**Formatted:** Font: 12 pt

737 Likelihood ratio test; SMD: Standardized mean difference; IRR: Incidence rate; MENISCUS:
738 Menstrual health interventions, schooling, and mental health problems among Ugandan
739 students; MHM: Menstrual hygiene management; SES: Socio-economic status; WASH:
740 Water, sanitation and hygiene; SAMNS: Self-efficacy in Addressing Menstrual Needs Scale;
741 MHPM: Menstrual Hygiene Preparation and Maintenance sub-scale; MPM: Menstrual Pain
742 Management sub-scale; EST: Executing Stigmatized Tasks sub-scale

**Formatted:** Font: 12 pt

743

744

745

**Formatted:** Line spacing: Double

## 746 ~~Funding~~

747 ~~This work was supported by the Joint Global Health Scheme with funding from the UK~~
748 ~~Foreign, Commonwealth and Development Office, the UK Medical Research Council, the~~
749 ~~UK Department of Health and Social Care through the National Institute of Health Research~~
750 ~~(NIHR), and Wellcome (Grant Ref. MR/ V005634/1). The funder had no role in the design of~~
751 ~~the study; in the collection, analyses, or interpretation of the data; in the writing of the article;~~

752

753

~~or in the decision to publish the results.~~

## 754 Declarations

755 **~~Ethical approval~~**
756 ~~Ethical approval for MENISCUS protocol, the informed consent forms and draft CRFs were~~
757 ~~obtained from the Uganda Virus Research Institute Research & Ethics Committee (UVRI-~~
758 ~~REC) (reference GC/127/819) and the Uganda National Council of Science and Technology~~
759 ~~(UNCST) (reference HS1525ES) and the LSHTM Research Ethics Committee (reference~~
760 ~~22952-2).~~

## 761 ~~Consent for publication~~

762 ~~Participants consented to the research findings being published in international science~~
763 ~~journals and electronic websites, on condition that that findings cannot be traced to~~
764 ~~individuals.~~

## 765 Availability of data and materials

766 The datasets used and/or analysed during the current study are available at LSHTM Data
767 Compass ~~from~~ ~~the corresponding author~~and can be freely accessed on reasonable request.
768 Details of location are; MENISCUS Trial quantitative survey data. [Dataset]. London School
769 of Hygiene & Tropical Medicine, London, United Kingdom. 10.17037/DATA.00003865
770 ([MENISCUS Trial quantitative survey data](https://datacompass.lshtm.ac.uk/id/eprint/3865/) ).

## 771 Competing interests

772 The authors declare that they have no competing interests.

773

774

775

776

777

778

779

780

781

782

783

784

785

786

787

788

## 789 References

**Formatted:** Line spacing: Double

790 1.	Hennegan J, Winkler IT, Bobel C, Keiser D, Hampton J, Larsson G, et al. Menstrual

**Formatted:** Font: (Default) Times New Roman, 12 pt

791 health: a definition for policy, practice, and research. Sex Reprod Health Matters.
792 2021a;29(1):1911618.
793 2.	Hennegan J, Shannon AK, Rubli J, Schwab KJ, Melendez-Torres GJ. Women’s and
794 girls’ experiences of menstruation in low- and middle-income countries: A systematic review
795 and qualitative metasynthesis. PLOS Medicine. 2019;16(5):e1002803.
796 3.	Wilkinson K. No data shows 1 in 10 African girls miss school because of
797 menstruation South Africa: Africa Check; 2016 [Available from: [https://africacheck.org/fact-](https://africacheck.org/fact-checks/reports/no-data-shows-1-10-african-girls-miss-school-because-menstruation)

**Formatted:** Font: (Default) Times New Roman, 12 pt

798 [checks/reports/no-data-shows-1-10-african-girls-miss-school-because-menstruation.](https://africacheck.org/fact-checks/reports/no-data-shows-1-10-african-girls-miss-school-because-menstruation)

**Formatted:** Font: (Default) Times New Roman, 12 pt

799 4.	Bobel C. The Managed Body: Developing Girls and Menstrual Health in the Global
800 South. Cham, Switzerland.: Palgrave Macmillan / Springer Nature Switzerland AG; 2018.
801 5.	Tadesse AG. Prevalence of school absenteeism during menstruation and associated
802 factors in sub-Saharan Africa: A systematic review and meta-analysis. Research Square.
803 2023.
804 6.	Starr M, Harding R, Ataíde R, Von Dinklage N, Sinharoy SS, Jayasinghe Y, et al.
805 Epidemiology of menstrual-related absenteeism in 44 low-income and middle-income
806 countries: a cross-sectional analysis of Multiple Indicator Cluster Surveys. The Lancet Global
807 Health. 2025;13(2):e285-e97.
808 7.	Betsu BD, Medhanyie AA, Gebrehiwet TG, Wall LL. Menstrual hygiene management
809 interventions and their effects on schoolgirls' menstrual hygiene experiences in low and
810 middle countries: A systematic review. PLoS One. 2024;19(8):e0302523.
811 8.	Benshaul-Tolonen A, Zulaika G, Sommer M, Phillips-Howard PA. Measuring
812 Menstruation-Related Absenteeism Among Adolescents in Low-Income Countries. In: Bobel
813 C, Winkler IT, Fahs B, Hasson KA, Kissling EA, Roberts T-A, editors. The Palgrave
814 Handbook of Critical Menstruation Studies. Singapore: Springer Singapore; 2020. p. 705-23.

815

816

817

9.	Hennegan J, OlaOlorun FM, Oumarou S, Alzouma S, Guiella G, Omoluabi E, et al.

School and work absenteeism due to menstruation in three West African countries: findings from PMA2020 surveys. Sex Reprod Health Matters. 2021b;29(1):1915940.

**Formatted:** Font: (Default) Times New Roman, 12 pt, Portuguese (Portugal)

**Formatted:** Font: (Default) Times New Roman, 12 pt

818 10.	Munro AK, Hunter EC, Hossain SZ, Keep M. A systematic review of the menstrual
819 experiences of university students and the impacts on their education: A global perspective.
820 PLoS One. 2021;16(9):e0257333.
821 11.	Armour M, Parry K, Manohar N, Holmes K, Ferfolja T, Curry C, et al. The
822 Prevalence and Academic Impact of Dysmenorrhea in 21,573 Young Women: A Systematic
823 Review and Meta-Analysis. Journal of Women's Health. 2019;28(8):1161-71.
824 12.	Miiro G, Rutakumwa R, Nakiyingi-Miiro J, Nakuya K, Musoke S, Namakula J, et al.
825 Menstrual health and school absenteeism among adolescent girls in Uganda (MENISCUS): a
826 feasibility study. BMC Women's Health. 2018;18(1):4.
827 13.	Nelson KA, Lagony S, Kansiime C, Torondel B, Tanton C, Ndekezi D, et al. Effects
828 and costs of a multi-component menstrual health intervention (MENISCUS) on mental health
829 problems, educational performance, and menstrual health in Ugandan secondary schools: an
830 open-label, school-based, cluster-randomised controlled trial. Lancet Glob Health.
831 2025;13(5):e888-e99.
832 14.	Kansiime C, Hytti L, Nelson KA, Torondel B, Francis SC, Tanton C, et al. Menstrual
833 health interventions, schooling, and mental health problems among Ugandan students
834 (MENISCUS): study protocol for a school-based cluster-randomised trial. Trials.
835 2022;23(1):759.
836 15.	Statistics UBo. The National Population and Housing Census 2024 – Final Report -
837 Volume 1 (Main). Kampala, Uganda: Uganda Bureau of Statistics; 2024 DECEMBER 2024.
838 16.	UBOS. The national population and housing census 2014 – area specifc profle series -
839 Wakiso. Kampala: UBOS; 2017.
840 17.	UBOS. The National Population and Housing Census 2014 – Area
841 Specific Profile Series-Kalungu district. Kampala: UBOS; 2017.

**Formatted:** Font: (Default) Times New Roman, 12 pt, Portuguese (Portugal)

842 18.	Ssemata AS, Ndekezi D, Kansiime C, Bakanoma R, Tanton C, Nelson KA, et al.
843 Understanding the social and physical menstrual health environment of secondary schools in

**Formatted:** Font: (Default) Times New Roman, 12 pt

844 Uganda: A qualitative methods study. PLOS Global Public Health. 2023;3(11):e0002665.
845 19.	Nelson KA, Lagony S, Kansiime C, Torondel B, Tanton C, Ndekezi D, et al. Effects
846 and costs of a multi-component menstrual health intervention (MENISCUS) on mental health
847 problems, educational performance, and menstrual health in Ugandan secondary schools: an
848 open-label, school-based, cluster-randomised controlled trial. The Lancet Global Health.
849 2025;13(5):e888-e99.
850 20.	Hunter EC, Murray SM, Sultana F, Alam MU, Sarker S, Rahman M, et al.
851 Development and validation of the Self-Efficacy in Addressing Menstrual Needs Scale
852 (SAMNS-26) in Bangladeshi schools: A measure of girls' menstrual care confidence. PLoS
853 One. 2022;17(10):e0275736.
854 21.	Sterne JA, Davey Smith G. Sifting the evidence-what's wrong with significance tests?
855 Bmj. 2001;322(7280):226-31.
856 22.	Hennegan J, Nansubuga A, Smith C, Redshaw M, Akullo A, Schwab KJ. Measuring
857 menstrual hygiene experience: development and validation of the Menstrual Practice Needs
858 Scale (MPNS-36) in Soroti, Uganda. BMJ Open. 2020;10(2):e034461.
859 23.	Evans DK, Mendez Acosta A. How to measure student absenteeism in low- and
860 middle-income countries. Economics of Education Review. 2023;96:102454.
861 24.	Keppens G, Spruyt B, Dockx J. Measuring School Absenteeism: Administrative
862 Attendance Data Collected by Schools Differ From Self-Reports in Systematic Ways. Front
863 Psychol. 2019;10:2623.
864 25.	Mason L, Nyothach E, Alexander K, Odhiambo FO, Eleveld A, Vulule J, et al. 'We
865 keep it secret so no one should know'--a qualitative study to explore young schoolgirls
866 attitudes and experiences with menstruation in rural western Kenya. PLoS One.
867 2013;8(11):e79132.
868 26.	El-Gilany AH, Badawi K, El-Fedawy S. Menstrual hygiene among adolescent
869 schoolgirls in Mansoura, Egypt. Reprod Health Matters. 2005;13(26):147-52.
870 27.	Mason L, Nyothach E, Alexander K, Odhiambo FO, Eleveld A, Vulule J, et al. ‘We
871 Keep It Secret So No One Should Know’ – A Qualitative Study to Explore Young
872 Schoolgirls Attitudes and Experiences with Menstruation in Rural Western Kenya. PLOS
873 ONE. 2013;8(11):e79132.
874 28.	Tegegne TK, Sisay MM. Menstrual hygiene management and school absenteeism
875 among female adolescent students in Northeast Ethiopia. BMC Public Health.
876 2014;14(1):1118.
877 29.	Korpershoek H, T. CE, M. F-B, and de Boer H. The relationships between school
878 belonging and students’ motivational, social-emotional, behavioural, and academic outcomes
879 in secondary education: a meta-analytic review. Research Papers in Education.
880 2020;35(6):641-80.
881 30.	Topistar N, Dennis Zami Atibuni P, David Kani Olema P, Ujeyo MSS. Demystifying
882 Menstrual Health Management to Enhance Academic Performance among Adolescent
883 Primary School Girls in Rural Uganda. East African Journal of Education Studies.
884 2022;5(2):298-309.
885 31.	Keppens G. School absenteeism and academic achievement: Does the timing of the
886 absence matter? Learning and Instruction. 2023;86:101769.

887

888

889

890

**S1 Table. Baseline menstrual-related exposures by endline school absenteeism for any reason**

59

Response to Reviewers

Dear Editor

Thank you for reviewing the manuscript. We have revised our manuscript to address the Editor and reviewer comments. Our detailed responses are below.

1. Please ensure that your manuscript meets PLOS ONE's style requirements, including those for file naming. The PLOS ONE style templates can be found at

# Response: We have followed these style requirements

2. Please note that funding information should not appear in any section or other areas of your manuscript. We will only publish funding information present in the Funding Statement section of the online submission form. Please remove any funding-related text from the manuscript.

# Response: The funding statement has been removed from manuscript and is only in the Funding Statement.

3. When completing the data availability statement of the submission form, you indicated that you will make your data available on acceptance. We strongly recommend all authors decide on a data sharing plan before acceptance, as the process can be lengthy and hold up publication timelines. Please note that, though access restrictions are acceptable now, your entire data will need to be made freely accessible if your manuscript is accepted for publication. This policy applies to all data except where public deposition would breach compliance with the protocol approved by your research ethics board. If you are unable to adhere to our open data policy, please kindly revise your statement to explain your reasoning and we will seek the editor's input on an exemption. Please be assured that, once you have provided your new statement, the assessment of your exemption will not hold up the peer review process.

**Response: We confirm that the data are freely accessible upon request at the LSHTM Data Compass repository (**[MENISCUS Trial quantitative survey data](https://datacompass.lshtm.ac.uk/id/eprint/3865/)**) line 518-522**

4. Your ethics statement should only appear in the Methods section of your manuscript. If your ethics statement is written in any section besides the Methods, please move it to the Methods section and delete it from any other section. Please ensure that your ethics statement is included in your manuscript, as the ethics statement entered into the online submission form will not be published alongside your manuscript.

# Response: Thank you for the guidance. The ethic statement has been moved to the Methods section (lines 238-242)

5. Please include a separate caption for each figure in your manuscript.

# Response: We have two figures and provide captions in the manuscript (lines 233 and lines 258 respectively).

6. Please include captions for your Supporting Information files at the end of your manuscript, and update any in-text citations to match accordingly. Please see our Supporting Information guidelines for more information:

# Response: We have added the caption for the Supporting Information file at the end of the manuscript (line 640) and its in-text citation has been updated on lines 333 and line 357

7. Please upload a copy of your study protocol that was approved by your ethics committee/IRB as a Supporting Information file. By the study protocol, we mean the complete and detailed plan for the conduct and analysis of the trial approved by the ethics committee/IRB. Please send this in the original language. If this is in a language other than English, please also provide a translation.

# Response: The protocol has been uploaded as a supporting file

8. If the reviewer comments include a recommendation to cite specific previously published works, please review and evaluate these publications to determine whether they are relevant and should be cited. There is no requirement to cite these works unless the editor has indicated otherwise.

# Response: There were no such comments

**Reviewer Comments to the Author**

**Reviewer #1:**

The paper lacks clarity from the statistical perspective. The statistical strategy is poorly presented in this study. Section 10 of the supplemental protocol is well written with sample size justification and various analysis options per endpoints discussed.

# Response: Thank you for this comment. We have substantially revised the section on statistical analysis (Section 2.7) to clarify how we followed a conceptual framework (Figure 1) to build the multivariable models. Specifically, this first describes the two types of models used (mixed-effect negative binomial for school absenteeism, and mixed- effects linear regression for examination score) and then describes the conceptual framework and how the multivariable models were built for each level.

**As this study was nested within the MENISCUS cluster-randomised controlled trial, we did not conduct a sample size calculation for this nested analysis.**

When one starts reading the paper, the design and parallel arms not even introduced in the Abstract. One has to wait until section 2.2 and the investigators finally state that the trial was a parallel-arm, cluster-randomized controlled trial in 60 schools with schools randomized 1:1 to either immediate or delayed intervention delivery.

# Response: We apologise for the confusion with the study design. We have clarified that this manuscript reports results from a secondary observational analysis using data from the published MENISCUS trial. It has been clarified in the abstract on lines 29-30, in the introduction on lines 114-119, and the methods on lines 130-144.

1. What is the control arm mentioned in the abstract?

# Response: This is the control arm from the trial within which the current analyses are nested. We have clarified this in the abstract as follows “We conducted secondary observational analyses using data from a cluster-randomised trial of a menstrual health intervention in 60 Ugandan secondary schools. We used baseline data from trial participants in both arms, and endline data from the control arm participants. “(lines 29-30). We also now include a study flowchart (Figure 2) to help clarify this.

2. Also, the actual longitudinal input is not clear to the reader. Please relate immediate, delayed, and endline in a longitudinal context with respect to Figure 1.

# Response: We thank the reviewer for this comment, and have removed the word ‘longitudinal’ throughout the manuscript. Instead, we describe the study as a prospective study in the title, and refer to baseline and endline data analysis in the

**abstract and manuscript (e.g. lines 29-30, 43-45 in the abstract), including in Figures 1 and the new Figure 2 which is the study flowchart.**

3. In the Conclusion the authors note that among Ugandan students, multiple dimensions of menstrual health are associated with school absenteeism and educational performance. Where is the analysis of immediate or delayed intervention or is that not part of this report?

# Response: Thank you for the comment. We have clarified in the Introduction section of the manuscript that this manuscript reports secondary data analysis of the trial, and that the intervention effects have been published previously (lines 103-113). We have also clarified that the estimate of the magnitude of effects in this paper help explain the lack of intervention effect (lines 380-383) “Through this observational analysis, we have shown that dimensions of menstrual health are associated with educational outcomes, but the absolute effect was small, which may help explain why the intervention was not sufficient to improve these educational outcomes.”

4 It looks like Tables 2 to 4 are baseline and Table 5 are endline results from the longitudinal approach? Is that correct?

# Response: Yes that is correct. We apologise for an error in title of Table 5 which suggested the outcomes were at baseline (line 361).

5. Some sentences are vague. For example ,please explain line 292, ’ After adjusting for factors at the same or more distal levels, there was strong evidence that menstrual-related absenteeism and poorer examination performance were both associated…etc’. What does 'distal level' mean? Please be exact when you mention adjustment throughout the presentation.

# Response: Thank you for this comment. We have revised the manuscript throughout to improve clarity. This includes rewriting Section 2.7 on Statistical Analyses to clarify the model building strategy . This includes definition of ‘distal level’ (to say “We use the term “distal” to refer to variables hypothesized to causally precede and influence variables at more proximal levels” (lines 209-220).

6. The entire presentation has to be edited for organization and clarity.

# Response: We thank the reviewer for this comment and have edited the manuscript throughout to improve clarify.

**Reviewer #2:**

1. Abstract
   - Clearly explain how factors such as inadequate menstrual materials, negative attitudes toward menstruation, unmet menstrual practice needs, and experiences of menstrual teasing influence educational performance, not just absenteeism.

# Response: We had included this in the discussion but have moved to the introduction (lines 55-59).“Poor menstrual health is prevalent in low- and middle-income countries (LMICs), and may affect educational outcomes such as school absenteeism and examination performance through multiple pathways, including through shame, anxiety, lack of confidence, behavioural expectations, poor physical environments, and inadequate menstrual products”

Also clarify what is meant by “multiple dimensions of menstrual health” and how these are associated with both absenteeism and educational performance (may be in the body).

# Response: We have defined the dimensions of menstrual health in the introduction (lines 70-73) “These dimensions include physical, behavioural and social components such as inadequate menstrual materials, menstrual pain, poor menstrual knowledge and confidence, and inadequate infrastructure for managing menstruation in schools”

- Article type: Why is this described as a clinical trial? Since the study used secondary data and so sign of any medical test. Please justify the article type or indicate this clearly in the methods section.

# Response: Thank you for the comment. We have clarified in the manuscript that the paper is a secondary analysis using data from a cluster-randomised controlled trial (lines 123-124) and corrected this in the submission form.

2. Introduction
   - Citations: Use square brackets for in-text citations instead of parentheses, to maintain consistency with journal style.

# Response: Thank you. These have been changed to square brackets in line with journal style.

- Objective: Ensure the objective gives equal emphasis to both class absenteeism and educational performance. This balance appears lacking in the last part of the introduction. **Response: We have revised the objective to include “examination performance” (line 114-117) and have revised the manuscript throughout to improve the balance.**
- MENISCUS: Define this acronym (e.g., “MENISCUS stands for...”) before using it, to help readers unfamiliar with the term.

# Response: We have defined “MENISCUS” as “Menstrual health interventions, schooling, and mental health problems among Ugandan students (MENISCUS)” at first use on lines 112-113.

3. Methods
   - Government vs private schools: What was the purpose of sampling both government and private schools if the outcomes are not contrasted by school type? Please clarify.

# Response: We have explained reasons for sampling both government and private schools on lines 156-158: “We over-sampled government schools as these are of interest to Ministry of Education stakeholders, and to increase generalisability of findings to these schools”.

- Interventions: Specify the interventions received by the treatment schools versus what the control schools received.

# Response: We have added brief details of the intervention components for completeness on lines 137-140. We have also clarified that control schools were provided with a copy of the government guidelines on menstrual hygiene management and sexuality education and menstrual management reader.

One of the selection criteria mentioned was the presence of WASH services, suggesting there might not have been an intervention. Please clarify this point. If there were interventions, how they were managed and how frequent they were?

# Response: We have clarified that we only use baseline trial data from the intervention arm in this paper, plus endline data from the control arm (lines 131-135) so there are no

**post-intervention data from the intervention arm. The WASH services mentioned were already present at the schools at baseline, and having “a minimum WASH facility” was one of the trial eligibility criteria. We have clarified this on lines 148-155.**

- Quality control: What methods were employed to minimize recall bias in reporting class absenteeism?

# Response: We have revised the methods to clarify “To minimize recall bias, we used cognitive testing before the survey to ensure clearly worded questions anchored to specific time frames, and provided reminders of school terms and holidays during survey completion. During self-completion of the questionnaires, research assistants were present to assist participants with clarifications about the recall period and absence reasons.” Lines 192-196

- If the main results of MENISCUS have already been published, what new insights does this data analysis provide? Clearly, highlight the novel contributions/how different is this finding?

# Response: We have revised the discussion to highlight the new insights provided by this analysis (lines 376-382). “Through this observational analysis, we have shown that multiple dimensions of menstrual health are associated with educational outcomes, but the absolute effect was small, which may explain why the intervention was not sufficient to improve these educational outcomes”.

- Why did you use both cross-sectional and longitudinal designs? Are there sufficient indicators that support the longitudinal approach? It seems data were collected only at

baseline, end line, and through student self-reports, with no evidence of continuous data collection.

# Response: We chose to analyse prospective data in the control arm to address the possibility of reverse causality which could be in the baseline cross-sectional data. For example, it is possible that students with more absenteeism missed learning about how to manage their menstruation, and had poorer menstrual experiences. We chose to restrict this analysis to the control arm to assess this relationship in the absence of the MENISCUS intervention and hence make the results more generalisable. We have clarified this in the methods section (lines 227-231).

**“To mitigate against the possibility of reverse causality, and to assess temporal associations of exposures with the outcomes, we also analysed associations of baseline exposures with endline outcomes in the control arm, using analogous methods. We restricted these analyses to participants in the control arm as the MENISCUS intervention may have modified the association.”**

4.

Results

- Findings on the effects of menstrual health on educational performance are limited and not strongly demonstrated. Consider discussing this limitation.

# Response: Thank you for your comment. We have improved the discussion for example line 434-436 “However, overall, associations of dimensions of menstrual health with examination scores were weaker than with school absenteeism. This is as expected given that absenteeism is more proximal to poor menstrual health than educational performance”

**We have revised the limitations section to note that these findings are modest and should be interpreted cautiously due to potential limitations such as residual confounding and limited sensitivity of educational measures (Lines 458-462). Also future research with longer follow-up, larger sample sizes, more sensitive and diverse academic outcome measures, and designs that better isolate menstrual health effects from other educational influences is needed to fully understand the impact of menstrual health on academic achievement (lines 479-482)**

General comments

- Clearly distinguish between this study and the original MENISCUS study to avoid confusion.

# Response: Thank you for the comment. We have revised the paper throughout to clarify that this paper uses baseline data from the trial, plus endline data in the control arm, and does not estimate intervention effects on the educational outcomes as these were presented in the trial paper. This is highlighted in the introduction (lines 114-117), methods (lines 131-135), and discussion (lines 379-382).

- Explicitly state the specific menstrual-related factors analyzed, instead of repeatedly using the vague term “multiple dimensions.” This will make your findings clearer and more impactful.

# Response: Thank for the guidance. We have edited the paper to specify the menstrual- related factors throughout the manuscript.

- Follow PLOS ONE reference format

# Response: We apologise for this oversight. The citations are now in the PLoS One format.

**Reviewer #3:**

The authors have presented an intelligble fashion and the grammar is standard. The author should look at the punctuation and the tenses. The methodolgy is okay and the it is technically sound. The authour should avoid being too wordy. Some paragraphs are too long. He should make them brief and to the point.

# Response: We thank the reviewer for their comments, and have revised the manuscript throughout for clarity, including shortening sentences and paragraphs.

**Reviewer #4:**

Abstract

Overall the abstract needs a bit of work, context and clarification. Please provide a very brief (understanding the tight word count) description of the intervention in the methods (or background) to help orient the reader.

# Response: We thank the reviewer for their comment and have edited the abstract, including clarification that this is secondary analysis of observation (baseline and endline) data from cluster randomized trial (line 29-30). We prefer not to include a description of the intervention in the abstract as we are not using any post-intervention data in this analysis.

39: Do you mean “we analysed baseline and endline data”?

# Response: Thank you for this correction. We have edited the abstract as suggested (line 31-32).

The results section needs tailoring.

47: At baseline 3312 participants reported menstruating in the last 6 months. 3312 of how many? Or are you trying to say that your sample was the 3312 participants who reported menstruating in the last 6 months. Please clarify or consider revising: “Of the 3312

participants who reported menstruating in the past 6-months at baseline, 323 (9.8%) reported missing at least one day of school per month due to menstruation.”

# Response: We have edited the sentence as suggested (line 39-41)

49: 1286 (38.8%) missed at least one day a month for any reason. Is this relevant? Is this key to be reported in the abstract? Without context it lacks definition of why it is relevant.

# Response: We have removed this sentence from the abstract.

50: Of the 1192 participants in the control arm seen at endline, 135 (11.3%) missed at 51 least one day due to menstruation. Why is the control endline reported, and not the intervention arm endline? I don’t understand the relevance of reporting the control endline school absenteeism without the intervention data. What are you trying to say here? Can you add some clarifying text or otherwise provide some other data to clarify why this has been reported.

# Response: We clarified that we are reporting results for a secondary data of observation analysis (line 29-30) and clarified our rationale for not including intervention arm participants in the endline data analysis in the methods section, also this is not an evaluation analysis (lines 227-231) “To mitigate against the possibility of reverse causality, and to assess temporal associations of exposures with the outcomes, we also analysed associations of baseline exposures with endline outcomes in the control arm, using analogous methods. We restricted these analyses to participants in the control arm in the MENISCUS intervention which may have moderated the association”

53: associated with multiple dimensions of menstrual health. Multiple dimensions of poor menstrual health. The bracketed examples are the inverse of the dimensions on the conceptual model (described later). Potentially refer to the model here, saying “as per a conceptual

model described in the paper” - or something to orient a reader who is not familiar with the model. Another way might be to use the phrasing as per the introduction “aspects of

menstrual experience”. Perhaps instead of brackets, change to such as…

# Response: We thank the reviewer for this suggestion. We have removed the wording about ‘dimensions’ of menstrual health in the results section in favour of specific menstrual practices, although we retain it in the conclusion to summarise the findings (lines 43-46). “There was evidence that menstrual-related absenteeism and poorer examination performance at endline were both associated with use of inadequate menstrual materials, negative menstrual attitudes, unmet menstrual practice needs, and experience of menstrual-related teasing”.

correct (and not used consistently throughout paper). Note: Line 306: “menstrual teasing” and Line 324 should be edited for consistency.

# Response: We have corrected this throughout the manuscript.

Introduction

71: A widely-cited statistic that “one in 10 school-age girls in Africa misses school or drops out for reasons related to her period” is not evidence-based (3, 4). I get what you are trying to say here, but it reads strangely to me. Consider rephrasing. “One in ten school-age girls in

Africa missed school or drops out for reasons related to her period” is a widely –cited statistic that lacks scientific backing.

# Response: Thank you for the suggestion. We have modified the sentence to “The statement "one in 10 school-age girls in Africa misses school or drops out for reasons related to her period" is a widely-used statistic, but it is not supported by scientific research [3, 4]. “ (lines 65-66)

74: Consider: drop female – redundant. An average of 31%?

# Response: We have edited as suggested (line 68).

75: Edit: Far too long sentence. Use full-stop after longer duration, then begin sentence with Additionally, the review found menstruation also affected...

# Response : Thank you, the sentence has been revised and edited (lines 70-71).

**Response: We have revised as suggested (line 80).**

96: LMICs

# Response: We have revised as suggested (line 81).

99: Change to: ...with academic performance a reported consequence of menstruation, mainly due to menstrual pain or heavy menstrual bleeding.

**Response: We have revised this as “**A commonly-reported reason for menstrual-related absenteeism is menstrual pain, which can also contribute to reduced participation, concentration, and academic performance” **(line 89-90).**

102: Broader educational issues are written here as self-esteem, school participation, and educational performance. How is educational performance difference from academic performance? Above has been discussing academic performance, please clarify how or if educational performance is different and thus belongs to “broader educational issues.”

# Response: We have clarified the paper throughout to refer to educational outcomes as both school absenteeism and examination performance, and have replaced ‘academic performance’ and ‘educational performance’ throughout with either ‘educational outcomes’ or ‘examination performance’ as appropriate.

102/103: Should this sentence: “In the review among university students...”come above the sentence “There is relatively little and inconclusive evidence..” Im confused as to why this sentence separates the following comment on the review. Please edit or justify.

# Response: We thank the reviewer for the comment, and have edited as suggested.

109: You introduce us to: “Our recent cluster-randomised trial” is this the same cluster- randomised trail described in the methods? Aka the MENISCUS? If so, introduce it here not below. Please clarify or change the use of “our.” This is confusing.

# Response: Yes it’s the same, we have clarified this as follows: “Our recent cluster- randomised trial, the MENISCUS study (“Menstrual health interventions, schooling, and mental health problems among Ugandan students”) showed strong evidence that a multi-component menstrual health intervention improved multiple dimensions of menstruation, including knowledge about puberty and menstruation, use of effective pain management, menstrual self-efficacy, and reduced unmet menstrual needs, over a one-year period [15]. However, Lines 103-104.

111: Add: showed strong evidence of an intervention effect on multiple dimensions of menstrual health, such as....

# Response: Revised as above. Line 105-108

Please detail what dimensions were effected, considering below you say little on school absence and nothing on examination performance, or school absence overall.

# Response: Revised, as above Line 108-113 “there was little evidence of an intervention effect on school absence (measured using prospectively-collected daily diaries) during menstruation (11.2% vs 13.5% days missed; adjusted odds ratio (aOR)=0.81, 95%CI 0.62, 1.05). There was also no evidence of an intervention effect on examination

**performance (adjusted mean difference (aMD)=0.06, 95%CI -0.12, 0.24) or on school absence overall (10.5% days missed in both arms).”**

Additionally, is examination performace to be understood as different to academic performance, or educational performance. Please standardize or define.

# Response: As mentioned above, we have clarified the wording to be consistent in using examination performance as the UNEB examination, and ‘educational outcomes’ to mean both school absenteeism and examination performance. We have removed reference to academic performance and educational performance.

117- 121: I suggest moving this last introductory paragraph to the top of the introduction to orient the reader and introduce MENISCUS.

After the first sentence introducing the MENISCUS trial, please provide a brief line to describe what the trial is.

# Response: We would prefer to first introduce the reader to the literature on menstrual health and educational outcomes before focusing on the specific data source for this study, the MENISCUS trial, but will follow the Editor’s guidance on this.

The last sentence in this paragraph has a typo. Do you mean “add to the knowledge-base" or “add to the existing evidence on the impact of..” I would also advise revising the sentence to read: “based on the impact of multiple dimensions of menstrual health..” or “based on the

impact of menstruation..”

# Response: We have revised this sentence as follows : “In this paper, we provide new insights by quantifying the associations of menstrual health experiences (including pain management, social support, menstrual practices, and attitudes) with number of days of school absenteeism and examination performance“(lines 117-120).

Methods

124: Add “The MENSISCUS trial/study”

# Response: Revised (line 123)

132: Be consistent with abstract ie: baseline and endline.

# Response: We have clarified this throughout to be consistent.

132: This should not be the first time you are spelling out the acronym for MENISCUS. Please introduce in the introduction as suggested and spell out on first use.

# Response: The acronym has been included in the introduction (line 103-104).

142-143: Decapitalise Education Institutions.

# Response: This has been corrected line 148

143-148: This sentence is too long. Full-stop at 146 and then start sentence with “School enrolment was to be estimated as.	”

# Response: This has been corrected.

148: When you say “we” here, I am confused if you are talking about MENISCUS or your analysis of the trial? There needs to be clearer description of how what you are doing is

related to MENISCUS and if you referring to it as “yours” etc.

**Response: We apologise for the confusion. The sampling was done once, for the trial, by the same team that conducted the analysis for the current manuscript. We have removed the word ‘we’ here as follows:** A random sample of 60 schools were selected after being confirmed as eligible and willing to participate**. Line 155-156**

151: Provide explanation of Secondary 2 in parenthesis as above for School 1.

# Response: We have clarified this as “Secondary 2 (second year of secondary school)” (line 160)

166-167: Decapitalise subject names (Mathematics, Biology, English Language) (English remains capitalised- but not language)

# Response: We have corrected this (line 177-178)

187: Different reference style

# Response: This has been corrected (line 209).

188: Here you use the term “Menstrual-related factors”. I am wondering if you mean the same when you talk about “dimensions of menstrual health or “aspects of menstrual

experience.”

If so, could you please choose one, define it and standardize throughout the paper.

# Response: We have edited this (line 169-172) and elsewhere to be ‘dimensions of menstrual health’ which we have defined earlier (lines 61-64) as “multiple dimensions of menstrual health contribute to school absenteeism and reduced engagement [2]. These dimensions include physical, behavioural and social components such as inadequate

**menstrual materials, menstrual pain, poor menstrual knowledge and confidence, and inadequate infrastructure for managing menstruation in schools”**

190: Delete full-stop after confounders.”

# Response: We have corrected this (line 210).

198: See 188

# Response: Corrected

242: Describe/contextualise MPSN score. Is 2.11positive/negative?

# Response: We have clarified as follows (lines 270-272). “The mean MPNS score of 2.11 (SD=0.52) corresponds to an average response of “sometimes” having unmet menstrual practice needs. The possible range is 0 to 3, with 3 corresponding to no unmet needs.”

Discussion:

355: “These results add..”

# Response: We have corrected this (line 373)

365: Support – remove s

# Response: We have revised the sentence to “Our finding that 10% of participants reported missing at least one school day per month due to menstruation supports the widespread claim that one in 10 girls miss school due to menstruation” (line 386-388)

378: Sentence need clarification - “but reported that other girls were absent from school due to menstruation” or “but reported that other girls reported absenteeism”?

# Response: We have clarified this: “but reported that other girls were absent from school due to menstruation” (line 403)

381-382: Perhaps belongs in strengths and limitations

# Response: We prefer to retain this here as it is a recommendation, rather than a strength or limitation of our study

386: Suggest without adequate (not with inadequate).

# Response: We have made this change (line 412)

392: Menstrual preparedness. Where is this definition of menstrual preparedness from? I don't see obtaining, using, cleaning and disposing of menstrual products and seeking assistance when needed as menstrual preparedness. Menstrual preparedness is yes about tracking cycle and body literacy. Or pre-menarche about prior knowledge, education and support

# Response: We have clarified this by defining the menstrual preparation and Maintenance sub-scale as ‘menstrual preparedness” in Table 1, and referring to this Table in the sentence referred to by the reviewer (line 417-418).

**SCREEN-IT review**

Flow charts and attrition: We strongly recommend using flow charts because they provide an overview of the study design and more information about attrition.

# Response: We have added the flowchart as Figure 2.

Blinding: Please specify whether blinding was used at various phases of the experiment (e.g., blinding of patients, caregivers, outcome assessments, data analysis).

# Response: As this paper is not about intervention trial effects, we did not include this detail, but cite the main trial paper which includes details on blinding.

Please state how you determined the sample size for the study. If you performed a power or sample size calculation, please provide details

**Response: As this study was nested within the MENISCUS cluster-randomised controlled trial, we did not conduct a sample size calculation for this nested analysis.**
