# Supplementary material for: Associations of menstrual health with school absenteeism and examination performance among Ugandan secondary school students: A prospective study
Source: PLoS One. 2026 Jan 23;21(1):e0326549. doi: 10.1371/journal.pone.0326549 (PMC12829969; doi:10.1371/journal.pone.0326549)
Supplement: Attachment — Submitted filename: Reviewers comments.docx [file pone.0326549.s009.docx]

**Reviewer Comments on “Association of Menstrual Health with School Absenteeism and Educational Performance: Uganda”**

**1. Abstract: Comment addressed**

- Clearly explain how factors such as inadequate menstrual materials, negative attitudes toward menstruation, unmet menstrual practice needs, and experiences of menstrual teasing influence **educational performance**, not just absenteeism. Also clarify what is meant by “multiple dimensions of menstrual health” and how these are associated with both absenteeism and educational performance.
- **Article type:** Why is this described as a clinical trial? Since the study used secondary data and so sign of any medical test. Please justify the article type or indicate this clearly in the methods section.

**2. Introduction: Comment addressed!**

- **Citations:** Use square brackets for in-text citations instead of parentheses, to maintain consistency with journal style.
- **Objective:** Ensure the objective gives equal emphasis to both **class absenteeism and educational performance**. This balance appears lacking in the last part of the introduction.
- **MENISCUS:** Define this acronym (e.g., “MENISCUS stands for...”) before using it, to help readers unfamiliar with the term.

**3. Methods: Comment addressed!**

- **Government vs private schools:** What was the purpose of sampling both government and private schools if the outcomes are not contrasted by school type? Please clarify.
- **Interventions:** Specify the interventions received by the treatment schools versus what the control schools received. One of the selection criteria mentioned was the presence of WASH services, suggesting there might not have been an intervention. Please clarify this point. If there were interventions, how they were managed and how frequent they were?
- **Quality control:** What methods were employed to minimize recall bias in reporting class absenteeism?
- If the main results of MENISCUS have already been published, what new insights does this data analysis provide? Clearly, highlight the novel contributions/how different is this finding?
- Why did you use both cross-sectional and longitudinal designs? Are there sufficient indicators that support the longitudinal approach? It seems data were collected only at baseline, end line, and through student self-reports, with no evidence of continuous data collection.

**4. Results: Comment addressed!**

- Findings on the **effects of menstrual health on educational performance** are limited and not strongly demonstrated. Consider discussing this limitation.

**General comments: Comment addressed!**

- Clearly distinguish between this study and the original MENISCUS study to avoid confusion.
- Explicitly state the **specific menstrual-related factors** analyzed, instead of repeatedly using the vague term “multiple dimensions.” This will make your findings clearer and more impactful.
- Follow PLOS ONE reference format
