# Supplementary material for: Associations of menstrual health with school absenteeism and examination performance among Ugandan secondary school students: A prospective study
Source: PLoS One. 2026 Jan 23;21(1):e0326549. doi: 10.1371/journal.pone.0326549 (PMC12829969; doi:10.1371/journal.pone.0326549)
Supplement: Attachment — Submitted filename: Response_to_Reviewers_auresp_2.docx [file pone.0326549.s010.docx]

Dear Editor

Thank you for reviewing the manuscript. We have revised our manuscript to address the Editor and reviewer comments. Our detailed responses are below.

**Reviewer #3:** The grammatical errors that I sited have been addressed. The author can make the paragraphs brief and cite appropriately

We thank the reviewer for this comment.  We have made paragraphs briefer (lines 103-111; 186-187; 264-265; 311-312; 319-320).

We have also added a citation on line 111 as requested.
